# Phosphorus(III)-assisted regioselective C–H silylation of heteroarenes

**DOI:** 10.1038/s41467-020-20531-3

**Published:** 2021-01-22

**Authors:** Dingyi Wang, Xiangyang Chen, Jonathan J. Wong, Liqun Jin, Mingjie Li, Yue Zhao, K. N. Houk, Zhuangzhi Shi

**Affiliations:** 1 State Key Laboratory of Coordination Chemistry, Chemistry and Biomedicine Innovation Center (ChemBIC), School of Chemistry and Chemical Engineering, Nanjing University, Nanjing, 210093 China; 2 Department of Chemistry and Biochemistry, University of California, Los Angeles, Los Angeles, CA USA; 3 College of Chemical Engineering, Zhejiang University of Technology, Hangzhou, China

**Keywords:** Catalytic mechanisms, Homogeneous catalysis, Synthetic chemistry methodology, Coordination chemistry

## Abstract

Heteroarenes containing carbon–silicon (C–Si) bonds are important building blocks that play an important role in the construction of natural products, pharmaceuticals, and organic materials. In this context, the C–H silylation of heteroarenes is a topic of intense interest. Indole C–H silylation can preferentially occur at the nucleophilic C3 and C2 position (pyrrole core), while accessing the C4-C7 positions (benzene core) of the indole remains highly challenging. Here, we show a general strategy for the regioselective C7-H silylation of indole derivatives. Mainly, the regioselectivity is determined by strong coordination of the palladium catalyst with phosphorus (III) directing group. Using this expedient synthetic strategy, the diverse C7-silylated indoles are synthesized effectively which exhibits the broad functional group compatibility. Moreover, this protocol also been extended to other heteroarenes such as carbazoles. The obtained silylated indoles have been employed in various transformations to enable the corresponding differently functionalized indole derivatives. Significantly, a cyclopalladated intermediate is successfully synthesized to test the hypothesis about the P(III)-directed C–H metalation event. A series of mechanistic experiments and density functional theory (M06-2X) calculations has shown the preferred pathway of this directed C–H silylation process.

## Abstract (web summary)

Indole C-H silylation preferentially occurs at the C3 and C2 positions, while functionalization of the benzene core (C4-C7 sites) remains challenging. Here, the authors report a regioselective C7-H silylation of indole derivatives assisted by strong coordination of the palladium catalyst with a phosphorus directing group.

## Introduction

**Fig. 1:**
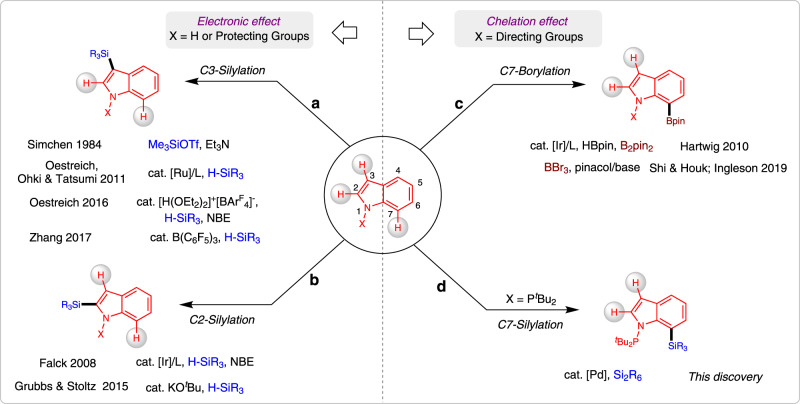
Development of a protocol to access C7-selective C–H silylation of indoles. **a** C–H Silylation of indoles at C2-position. **b** C–H Silylation of indoles at C2-position. **c** C–H Borylation of indoles at C7-position. **d** C–H Silylation of indoles at C7-position.

Silylation of the C–H bond^1–3^ is one of the most notable advances in the C–H functionalization field^4–11^ and it has been widely employed in the preparation of organosilicon compounds^12–20^. Heteroarylsilanes are considered as an important and versatile intermediates for the construction of complex molecules, since silyl groups can be easily transferred into a wide varieties of substituents^21–23^. Thus, C–H silylation of heteroarenes have extensively been employed for the preparation of highly important silylated heteroarenes^24–29^. Due to the presence of multiple C–H bonds in heteroaromatic compounds, the site-selective C–H bond functionalization is represents a key challenge in this arena. For example, the most familiar indole heteroarenes would eventually undergo electrophilic aromatic substitution (S_E_Ar) at C3-position (Fig. 1a). As early in 1984, Simchen and coworkers developed a method on regioselective electrophilic C3-silylation of indoles using Me_3_SiOTf^30^. Recently, several research groups have certainly reported C3-selective C–H silylation of indoles by merging cooperative Si-H bond activation and S_E_Ar reaction using catalytic amount of cationic Ru(II) complex^31^, Brønsted acid^32^, and B(C_6_F_5_)_3_^33^. Notably, some of the elegant protocols have also been involved for the site-selective C–H silylation of indoles at C2-position (Fig. 1b)^34^. In 2008, Falck and co-workers described an iridium-catalysed C–H silylation of *N*-unsubstituted indoles at the C2 position under mild condition using modest excess of Et_3_SiH reagent^35^. Similarly, in 2015, Stoltz and Grubbs reported a method to access C2-silylated indoles by KO^*t*^Bu-catalysed C–H silylation^36,37^. In this context, C–H silylation occurs preferentially at the nucleophilic C3 or C2 position, while accessing the benzene core of the indoles remains a great challenge^38–40^.

Usually, C7-selective C–H functionalization of indoles demands the installation of a functional group at the C2 position to block this possible site^41^. Notably, numerous research groups have recently made valuable progress on direct C–H functionalization of indoles at C7-position^42–49^. In 2010, the C7-selective C–H borylation of indole was uncovered through iridium catalysis with the assistance of *N*-silyl-directing group^50^. Our group^51,52^ and Ingleson group^53^ have reported the chelation-assisted C–H borylation mediated by BBr_3_, in which the installation of a pivaloyl group at the N1 position of indole selectively brings the boron species to the C7 position and allows subsequent C–H borylation in an efficient manner (Fig. 1c). Unlike the well-developed C–H borylation reactions, C7-silylation can only resort to C7-lithiated indoles with silicon electrophiles, this process is not compatible with many sensitive functional groups^54–57^. Herein, we report a regioselective C–H silyation of indoles at C7 position using organosilane reagents enabled by Pd(OAc)_2_ with oxidative conditions (Fig. 1d)^58–60^. The key to this high regioselectivity is the appropriate choice of N-P^*t*^Bu_2_ as a directing group and using 2,5-dimethyl-1,4-benzoquinone (DMBQ) as an external oxidant. The oxidant DMBQ can not only regenerate the catalyst, but also suppress the oxidation of the phosphorus (III) directing group.

## Results

### Reaction design

Our study commenced with the optimization of the reaction of indole **1a** and hexamethyldisilane (**I**) (Table 1). After extensive experimentation, using Pd(OAc)_2_ (10 mol%) as the catalyst and DMBQ (3.5 equiv) as the oxidant showed the best reactivity within 72 h at 120 °C without any external ligands, providing the C7-silylation product **2a** in 80% yield, in which the C2 and C3 silylation isomers **3** and **4** were not observed (based on GC; entry 1). Moreover, treatment of DMBQ with indole **1a** did not result in conversion to the oxidized by-products **5** and **6**, showing that *N*-P^*t*^Bu_2_ substituent was tolerated under the conditions. Indeed, the selection of a suitable oxidant was found to have a dramatic impact on the transformation. The reaction was conducted with 1,4-benzoquinone (BQ) as the oxidant, resulting in considerably lower yield of **2a** (entry 2). Treatment of indole **1a** either with both Ag_2_CO_3_ (entry 3) or Cu(OAc)_2_ (entry 4) showed much less efficient for C7-silylation, along with large amount of the by-product **5**. Using other palladium sources such as PdCl_2_ led to a slight decrease in yield (entry 5). Other transition metal catalysts like [Rh(cod)Cl]_2_, [RuCl_2_(p-cymene)]_2_ or [Ir(COD)Cl]_2_ were completely unsuccessful for this transformation, showing the uniqueness of palladium catalyst (entry 6-8). Changing the solvent to other nonpolar solvent like cyclohexane also provided product **2a** efficiently (entry 9), and the use of polar solvents involving DMF and THF, only led to trace amount of product **2a** (not shown in the table). The indole substrates bearing other directing groups like *N*-P(O)^*t*^Bu_2_ (**5**), *N*-Ts (**7**), *N*-Piv (**8**) and *N*-SMe (**9**)^49^ were failed to generate any C–H silylation products, and only a small amount of C2 silylation by-product was obtained with 1-(pyrimidin-2-yl)-1H-indole (**10**) (entry 10). These results confirm the importance of the *N*-P^*t*^Bu_2_ group moiety for achieving both high reactivity and selectivity. Other silane reagents including trimethyl(vinyl)silane (**II**), SiMe_2_Ph-Bpin (**III**), HSiEt_3_ (**IV**), and TfOSiMe_3_ (**V**) previously showed for the transition-metal-catalysed C–H silylations, but they completely failed in this reaction (entry 11). In addition, it was also found that the reaction temperature plays a substantial impact on this conversion (entry 12). As expected, the optimization studies clearly demonstrated that the reaction parameters including external oxidant DMBQ (entry 13) and palladium catalyst (entry 14) were optimal for the high conversion.

**Table 1:** Optimization of the reaction conditions^a^.


Entry	Variation from the “standard conditions”	Yield of 2a (%)^*b*^
1	none	80 (75)^*c*^
2	Using BQ instead of DMBQ	58
3	Using Ag_2_CO_3_ instead of DMBQ	24
4	Using Cu(OAc)_2_ instead of DMBQ	12
5	Using PdCl_2_ instead of Pd(OAc)_2_	72
6	Using [Rh(cod)Cl]_2_ instead of Pd(OAc)_2_	0
7	Using [RuCl_2_(*p*-cymene)]_2_ instead of Pd(OAc)_2_	0
8	Using [Ir(COD)Cl]_2_ instead of Pd(OAc)_2_	0
9	Using cyclohexane instead of toluene	71
10	Using **5**, **7-10** instead of **1a**	0
11	Using **II-V** instead of **I**	0
12	at 100 °C	62
13	Without DMBQ	6
14	Without Pd(OAc)_2_	0

^a^Standard conditions: **1a** (0.20 mmol), **I** (1.0 mmol), Pd(OAc)_2_ (10 mol%), DMBQ (0.7 mmol) in toluene (0.5 mL), 72 h, 120 ^o^C, under Ar. ^b^GC yield. ^c^Isolated yield.

### Scope of the methodology

**Fig. 2:**
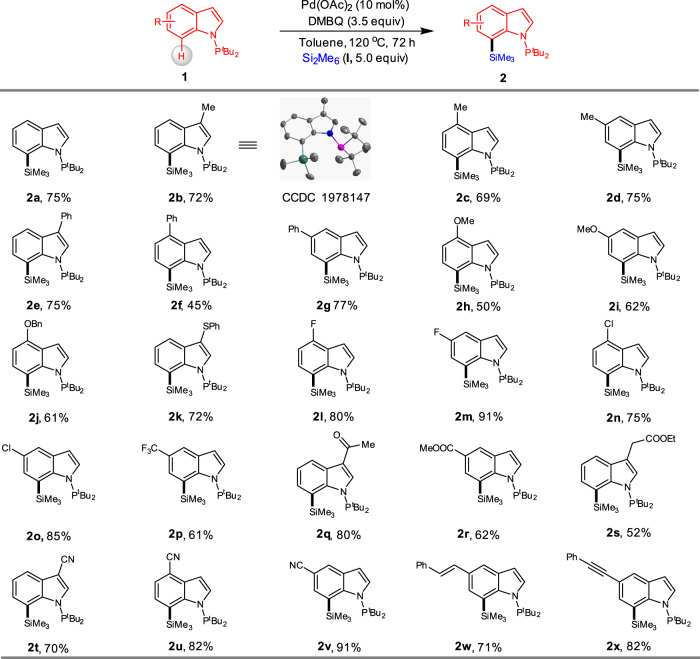
Substrate Scope. Reaction conditions: **1** (0.20 mmol), **I** (1.0 mmol), Pd(OAc)_2_ (10 mol%), DMBQ (0.7 mmol) in toluene (0.5 mL), 72 h, 120 °C, under Ar. All reported yields are isolated yields.

We then investigated the scope of the palladium-catalysed C7-selective C–H silylation of indoles (Fig. 2). Indoles bearing methyl (**2b–d**) and phenyl (**2e–g**) substituents at the C4-5 positions underwent facile C–H silylation and afforded the corresponding products in 45–77% yields. Among them, the structure of compound **2b** was confirmed by X-ray analysis (Supplementary Data 1). Indole substrates containing electron-donating groups such as OMe (**2** **h** and **2i**), OBn (**2j**) were well tolerated, and a phenylthio group (**2k**) that remained without oxidation or deactivation of the catalyst. The synthetically useful halogens such as F (**2** **l** and **2** **m**) and Cl (**2n** and **2o**) were compatible. We further observed that C7 silylation of electronically deficient CF_3_-substituted indole **2p** was also operative. Indole compounds bearing other electron-withdrawing groups like acetyl (**1q**), and ester (**1r** and **1** **s**) formed the corresponding products **2q-s** in 52–80% yields. Noticeably, the strong coordination ability of cyano group (**1t-v**) with metal catalyst didn’t inhibit the reaction outcome. In addition, indolylsilanes containing alkenyl (**2w**) or alkynyl (**2x**) substituent were also readily prepared using this methodology.

### Synthetic Applications

**Fig. 3:**
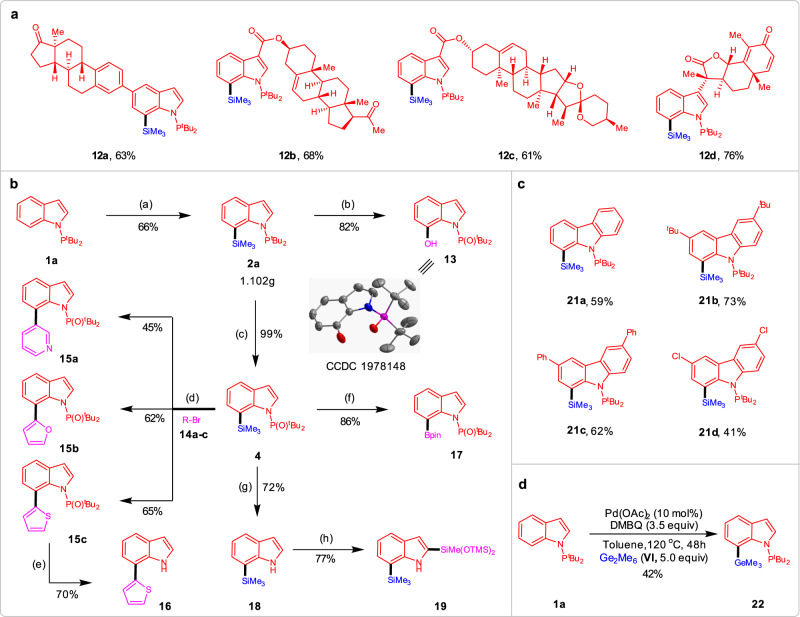
Further investigations. **a** C–H Functionalization of complex molecules. **b** Downstream transformations. **c** C–H Silylation of carbazoles. **d** C–H Germylation of indole **1a**. Reagents and conditions: (a) **1a** (5.0 mmol, 1.0 equiv.), **I** (30 mmol, 6.0 equiv.), Pd(OAc)_2_ (10 mol%), DMBQ (20 mmol, 4.0 equiv.) in toluene (10 mL), 4.5 days, 120 °C, under Ar; (**b**) **2a** (0.20 mmol), H_2_O_2_ (2.0 mL), KF (0.8 mmol), KHCO_3_ (0.8 mmol) in THF (1.0 mL), 72 h, 40 °C; **c** **2a** (0.20 mmol), H_2_O_2_ (1.0 mL) in THF (0.5 mL), 72 h, rt; **d** **2a** (0.20 mmol), BCl_3_ (0.24 mmol, 1 M in DCM) in DCM (1.0 mL), 6 h, rt, under Ar; then solvent was removed and RBr **14** (0.24 mmol), Pd(PPh_3_)_4_ (5 mol%), DME (2 mL) and 2 M Na_2_CO_3_ aqueous solution (0.5 mL), 24 h, reflux, under Ar. **e** **15c** (0.2 mmol), TBAF (1 M in THF, 0.4 mL) in THF (2.0 mL), 100 °C, 24 h. **f** **4** (0.20 mmol), BBr_3_ (0.24 mmol, 1 M in DCM) in DCM (1.0 mL), 2 h, 0 °C to rt, under Ar; then solvent was removed and pinacol (0.4 mmol) and NEt_3_ (1.0 mmol) in DCM (1.0 mL) were added, 1 h, rt; **g** **4** (0.20 mmol), iPr_2_AlH (8.0 equiv.) in THF (1.0 mL), 12 h, 100 °C, under Ar; **h** **18** (0.20 mmol), [Ir(OMe)(cod)]_2_ (5 mol% mmol), dtbpy (10 mol%), HSiMe(OTMS)_2_ (0.60 mmol), NBE (0.6 mmol) in THF (1.0 mL), 24 h, 80 °C, under Ar.

To showcase the practical utility of this C–H silylation process, further investigations were conducted (Fig. 3). First, the functionalization of complex substrates was viable for phosphorus(III)-assisted regioselective C–H silylation reaction (Fig. 3a). In order to demonstrate the potential of this transformation, the complex indole molecules **11a–d** were subjected to the C7-silylation protocol. The reactions proceeded smoothly, yielding the Si-containing desired compounds **12a–d** in 61–76% yield with excellent chemo- and regioselectivity. The ketone, ester, and olefin moieties were well tolerated without any observed side reactions. Silylation of complex molecule **11d** with a Michael accepter motif also proceeded well without affecting the sensitive dienone motif. Second, the silylation reaction can be scalable to conduct and they are suitable to undergo various subsequent transformations, which shows the great potential in synthetic utility (Fig. 3b). A gram-scale reaction was conducted with indole **1a**, and the C7-silylated product **2a** was obtained in 66%. This compound could selectively get oxidized to phenol **13** and indolylsilane **4** bearing the *N*-P(O)^*t*^Bu_2_ unit in the presence of H_2_O_2_ in excellent yields, and the former one was further confirmed by X-ray analysis (Supplementary Data 1). With the compound **4** in hand, a variety of powerful synthetic transformations are also demonstrated. For instance, the Suzuki–Miyaura cross-coupling of **4** with different heteroaryl bromides **14a–c** by Si-B exchange furnishes 7-heteroarylated indoles **15a–c** in 45–65% yields^61^. Further deprotection of thiophen-indole **15c** with TBAF under mild condition could form N-H free indole **16** in a 70% yield. The effective method to form boronate ester **17** was also demonstrated to proceed in good yield from silylated precursor **4**. In addition, the *N*-P(O)^*t*^Bu_2_ group in indole **4** could be removed to afford N-H free indole **18** in a 72% yield, which further undergo C2 selective C–H silylation by iridium catalyst, generating a bis-silylation product **19** in good yield. Third, this strategy can also be applied to site-selective C–H silylation of carbazoles (Fig. 3c). Considering the importance of carbazole and its derivatives widely used in functional materials, we further converted carbazoles **20a–d** into corresponding silylated products in 41–73% yields by palladium catalysis. Finally, the extension of the present protocol was further carried out on C7 selective C–H germanylation of indoles. Without changing the optimized condition, the reaction was conducted by employing indole **1a** with hexamethyldigermane (**VI**) gave the desired product **22** in a moderated yield (Fig. 3d).

## Discussion

**Fig. 4:**
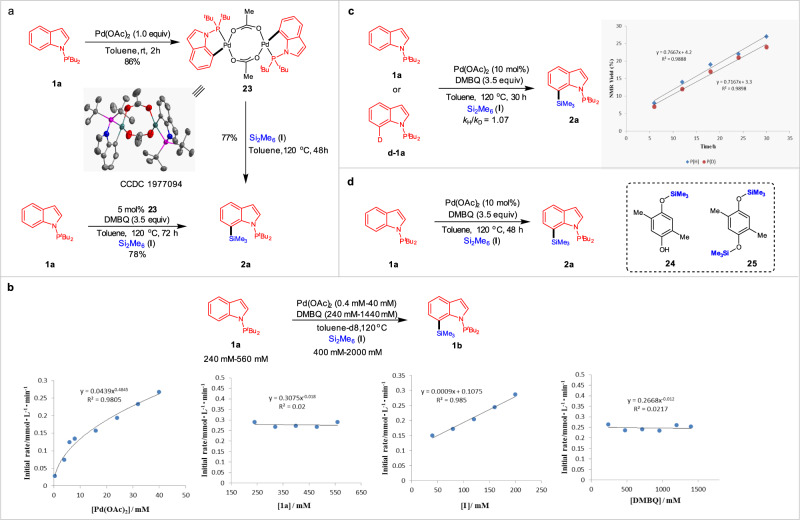
Mechanistic experiments. **a** Cyclopalladation of indole **1a** to a bimetallic Pd(II) complex **23**. **b** Kinetic profiles of Pd-catalysed C–Hsilylation between substrates **1a** and **I**. **c** KIE experiments of **1a** and **d-1a**. **d** Investigation of the byproducts from DMBQ.

To establish the mechanism for this C–H silylation process, we performed several mechanistic experiments (Fig. 4). When 1.0 equiv of indole **1a** was allowed to react with stoichiometric Pd(OAc)_2_ in toluene at room temperature for 2 h in the absence of Si_2_Me_6_ (**I**), a cyclometalated Pd^II^ dimer **23** was obtained and confirmed by X-ray analysis (Supplementary Data 1) (Fig. 4a). This complex displays square planar geometry stabilized by bridging acetate and N-P^*t*^Bu_2_ ligands. Furthermore, product **2a** could be generated by using catalytic amount of complex **23**. To gain deeper insight into the mechanism of the C–H silylation reaction, we performed kinetic investigation by using P NMR spectroscopy (Fig. 4b). Varying the concentration of Pd in the range of 0.4–40 mM, initial rates for the Pd(OAc)_2_-catalysed C–H silylation were obtained, which suggested a half-order dependence on [Pd]. By analogy to Sanford’s observation^62–64^, we proposed the resting state of the catalyst was the dimer **23**, which entered the catalytic cycle by dissociation to a monomer. Moreover, the reaction showed a zero-order dependence both on [indole **1a**] and [DMBQ] and a first order dependence on [Si_2_Me_6_ (**I**)], indicating that the coordination of N-P^*t*^Bu_2_ to Pd was very fast, DMBQ was not involved in the catalytic cycle, and the oxidative addition of Si–Si bond to catalyst center was the likely rate-limiting step. Next, we performed the intermolecular kinetic isotope effect (KIE) experiments using **1a** and **D-1a**, in which a small KIE value of 1.07 was observed (Fig. 4c)^65^. This observation further indicated that the C–H bond cleavage was not the rate-determining step of the reaction. Finally, we also sought to probe the role of DMBQ in this reaction. DMBQ-induced catalyst regeneration led to reduce DMBQ to the corresponding silylated hydroquinones, since compounds **24** and **25** could be observed in a mixture (Fig. 4d).

**Fig. 5:**
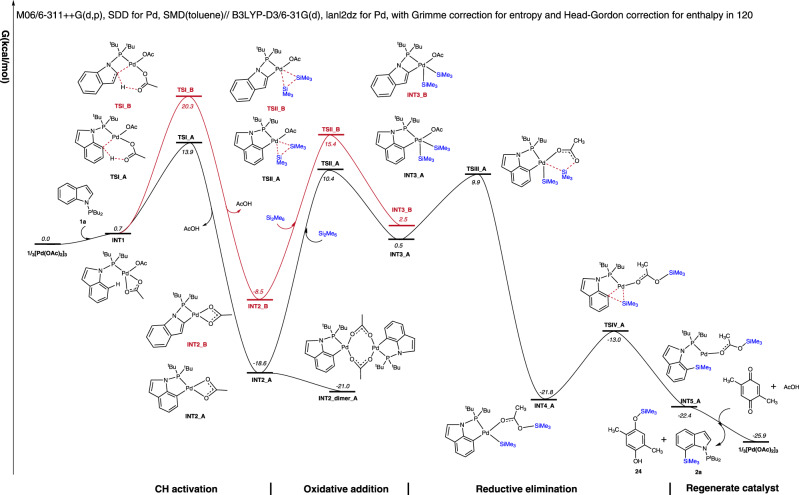
Free energy profiles for C2 (red) or C7 (black) selective C–H silylation of indole 1a. DFT method: M06/6-311 ++G(d,p), SDD for Pd, SMD(toluene)// B3LYP-D3/6-31 G(d), lanl2dz for Pd, with Grimme correction for entropy and Head-Gordon correction for enthalpy in 120°C. All energies are in kcal/mol.

Based on the above mechanistic experiments, density functional theory (DFT) calculations were then conducted on the model reaction of indole **1a** with Si_2_Me_6_ (**I**) to better understand the mechanism of this C–H silylation process and the C7 selectivity in indoles (Supplementary Data 2) (Fig. 5)^66,67^. Coordination of the Pd catalyst with **1a** to create **INT1** is endergonic by 0.7 kcal/mol due to an unfavorable decrease in entropy. **INT1** then deprotonates the C7 position of **1a** through a concerted metalation-deprotonation (CMD) transition state involving the carboxylate, **TSI_A** with an activation barrier of 13.9 kcal/mol^68–71^. This leads to a stable intermediate **INT2_A** with a relative free energy of −18.6 kcal/mol. The intermediate **INT2_A** can further dimerize to the resting state **INT2_dimer_A**, which can be isolated from the system. The subsequent oxidative addition step inserts Pd into the Si-Si bond of reagent **I** and leads to the five-coordinated **INT3_A** through **TSII_A** with a free energy of 10.4 kcal/mol. The SiMe_3_ then transfers the acetoxy group from Pd to the O of acetoxy through **TSIII_A** with a free energy of 9.9 kcal/mol to form the four-coordinated **INT4_A** (Fig. S1). The subsequent reductive elimination with the formation of C–Si bond proceeds easily with an active free energy of 8.8 kcal/mol with respect to **INT4_A**. According to the calculated free energy profile, the oxidative addition is the rate-determining step with a total energy barrier of 31.4 kcal/mol (**INT2_dimer_A** → **TSII_A**). The deprotonation from the C2 position was also studied. This leads to, however, a highly strained and unfavourable four-membered cyclic transition state **TSI_B** with a much higher free energy of 20.3 kcal/mol compared to its five-membered counterpart. This agrees with the experimental absence of the C2 silylation product **3**. A final dissociation would deliver the final product **2** while regenerating the active propagating Pd^II^ species in the presence of DMBQ and in-situ formed AcOH.

In summary, we have explored a reliable strategy on regioselective C–H silylation of heteroarenes enabled by N-P^t^Bu_2_ directing group. This strategy shows numerous advantages including the using of commercially available catalyst, avoiding the addition of the exogenous ligand, and compatibility with broad substrate scope. Because of the ubiquity of the indole and carbazole frameworks and their precursors in biologically active compounds, we hope that this strategy based on the diversity of silicon chemistry will simplify the synthesis and structural elaboration of heteroarylsilanes for advanced research in chemistry, biology, and medicine.

## Methods

### General procedures for synthesis of 2

In an oven-dried Schlenk tube, **1** (1.0 equiv, 0.20 mmol), Hexamethyldisilane **I** (5.0 equiv, 1.0 mmol), Pd(OAc)_2_ (10 mol%, 4.48 mg, 0.02 mmol), DMBQ (3.5 equiv, 95.2 mg, 0.70 mmol) were dissolved in toluene (0.5 mL). The mixture was stirred at 120 °C under argon for 72 h. Upon the completion of the reaction, the solvent was removed. The crude mixture was directly subjected to column Chromatography on silica gel using petroleum ether/ EtOAc as eluent to give the desired products **2**.

## Supplementary information

Supplementary Information

Peer Review File

Description of Additional Supplementary Files

Supplementary Data 1

Supplementary Data 2

## Data Availability

The authors declare that the data supporting the findings of this study are available within the article and its Supplementary Information Files as well as from the corresponding authors upon reasonable request. The crystallography data have been deposited at the Cambridge Crystallographic Data Center (CCDC) under accession number CCDC: 1978147 (**2b**), CCDC 1978148 (**13**), CCDC 1977094 (**23**), and can be obtained free of charge from www.ccdc.cam.ac.uk/getstructures.
