# Supplementary material for: Phosphorus(III)-assisted regioselective C–H silylation of heteroarenes
Source: Nat Commun. 2021 Jan 22;12:524. doi: 10.1038/s41467-020-20531-3 (PMC7822902; doi:10.1038/s41467-020-20531-3)
Supplement: Supplementary file 1 — Supplementary Information [file 41467_2020_20531_MOESM1_ESM.pdf]

Supplementary Information

Phosphorus(III)-Assisted Regioselective C–H Silylation of Heteroarenes

Dingyi Wang et al.

Supplementary Methods

General Information

Unless otherwise noted, all reactions were performed under an argon atmosphere using flame-dried glassware. Toluene, DCM and DCE were distilled over CaH2. All new compounds were fully characterized. NMR-spectra were recorded on Bruker AV-300, ARX-400 MHz or a ARX-600Associated. ^{1}H NMR spectra data were reported as δ values in ppm relative to chloroform (δ 7.26) if collected in $CDCl_3$. ^{13}C NMR spectra data were reported as δ values in ppm relative to chloroform (δ 77.00) if collected in $CDCl_3$. ^{1}H NMR coupling constants were reported in Hz, and multiplicity was indicated as follows: s (singlet); d (doublet); t (triplet); q (quartet); quint (quintet); m (multiplet);dd (doublet of doublets); ddd (doublet of doublet of doublets); dddd (doublet of doublet of doublet of doublets); dt (doublet of triplets); td (triplet of doublets); ddt (doublet of doublet of triplets); dq (doublet of quartets); app (apparent); br (broad). Mass spectra were conducted at Micromass Q-Tof instrument (ESI) and Agilent Technologies 5973N (EI). All reactions were carried out in flame-dried 25-mL Schlenk tubes with Teflon screw caps under argon.

General Procedure for Synthesis of Starting Materials

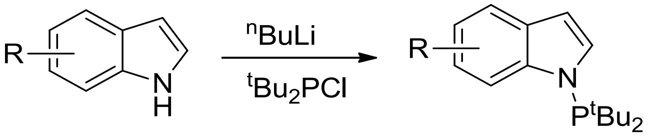

To a solution of indole (5.0 mmol, 1.0 equiv) in 20 ml anhydrous THF at 0 ℃ was added a solution of n-Butyllithium (2.5 M in hexane, 6.0 mmol, 1.2 equiv) dropwise. After stirring for 1 h, di-tert-butylchlorophosphine (6.0 mmol, 1.2 equiv) was added dropwise. The mixture was allowed to stir and warm to room temperature over several hours. After indole was consumed determined by TLC, the reaction was quenched by 2 mL MeOH. Then the solvent was removed under reduced pressure. Further purification through flash chromatography (petroleum-ether/ethyl acetate) can get the pure products **1**.

General Procedure for Selective C–H Silylation of Indoles at C7-Position

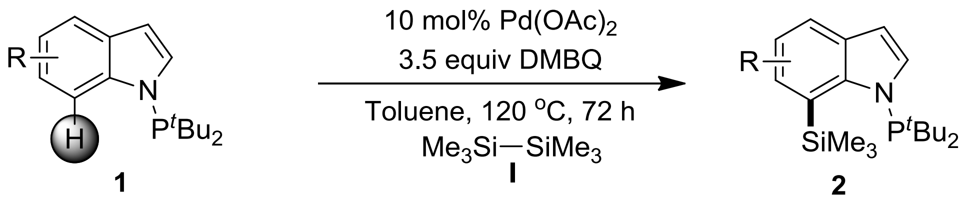

In an oven-dried Schlenk tube, **1** (1.0 equiv, 0.20 mmol), Hexamethyldisilane **I** (5.0 equiv, 1.0 mmol), $Pd(OAc)_2$ (10 mol%, 4.48 mg, 0.02 mmol), DMBQ (3.5 equiv, 95.2 mg, 0.70 mmol) were dissolved in toluene

(0.5 mL). The mixture was stirred at 120 ℃ under argon for 72 hours. Upon the completion of the reaction, the solvent was removed. The crude mixture was directly subjected to column Chromatography on silica gel using petrol ether/ EtOAc as eluent to give the desired products **2**.

1-(Di-tert-butylphosphino)-7-(trimethylsilyl)-1H-indole (2a)

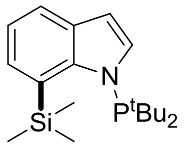

Following the general procedure, the reaction of **1a** (52.2 mg, 0.20 mmol), **I** (146 mg, 1.0 mmol), Pd(OAc)$_2$ (4.48 mg, 10 mol%), DMBQ (95.2 mg, 0.7 mmol) in toluene (0.5 mL) at 120 ℃ under Ar. After 72 h, purification by column chromatography on silica gel (petroleum-ether) yield **2a** (49.9 mg, 75%) as a colorless oil. **^{1}H NMR (500 MHz, Chloroform-*d*)** δ 7.68 (dt, *J* = 7.7, 1.3 Hz, 1H), 7.58 (dd, *J* = 7.2, 1.4 Hz, 1H), 7.47 (d, *J* = 3.4 Hz, 1H), 7.14 (t, *J* = 7.4 Hz, 1H), 6.71 (dd, *J* = 3.4, 1.3 Hz, 1H), 1.26 (d, *J* = 12.3 Hz, 18H), 0.58 (d, *J* = 2.4 Hz, 9H). **^{13}C NMR (126 MHz, Chloroform-*d*)** δ 147.8 (d, *J* = 21.9 Hz), 131.5, 131.2 (d, *J* = 4.9 Hz), 128.9 (d, *J* = 4.0 Hz), 123.3 (d, *J* = 7.0 Hz), 122.4, 119.6, 106.2 (d, *J* = 2.0 Hz), 36.2 (d, *J* = 28.8 Hz), 29.8 (d, *J* = 15.5 Hz), 3.8 (d, *J* = 13.9 Hz). **^{31}P NMR (162 MHz, CDCl$_3$)** δ 69.1. ATR-FTIR (cm^{-1}): 2956, 2895, 2864, 1514, 1259, 1148. HRMS m/z (ESI): calcd for $C_{19}H_{33}NPSi$ $(M + H)^+$ 334.2114, found 334.2115.

1-(Di-tert-butylphosphino)-3-methyl-7-(trimethylsilyl)-1H-indole (2b)

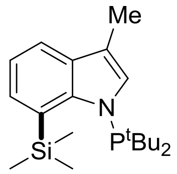

Following the general procedure, the reaction of **1b** (55.0 mg, 0.20 mmol), **I** (146 mg, 1.0 mmol), Pd(OAc)$_2$ (4.48 mg, 10 mol%), DMBQ (95.2 mg, 0.7 mmol) in toluene (0.5 mL) at 120 ℃ under Ar. After 72 h, purification by column chromatography on silica gel (petroleum-ether) yield **2b** (50.0 mg, 72%) as a colorless solid. **^{1}H NMR (500 MHz, Chloroform-*d*)** δ 7.56 (dt, *J* = 7.7, 1.3 Hz, 1H), 7.54 (dd, *J* = 7.3, 1.4 Hz, 1H), 7.17 (s, 1H), 7.11 (t, *J* = 7.5 Hz, 1H), 2.32 (s, 3H), 1.21 (d, *J* = 12.3 Hz, 18H), 0.53 (d, *J* = 2.4 Hz, 9H). **^{13}C NMR (126 MHz, Chloroform-*d*)** δ 148.3 (d, *J* = 22.3 Hz), 131.4, 129.3 (d, *J* = 4.0 Hz), 128.4 (d, *J* = 5.3 Hz), 123.2 (d, *J* = 7.4 Hz), 120.2, 119.1, 114.5 (d, *J* = 1.9 Hz), 36.1 (d, *J* = 28.4 Hz), 29.9 (d, *J* = 15.5 Hz), 3.8 (d, *J* = 13.9 Hz). **^{31}P NMR (162 MHz, CDCl$_3$)** δ 70.0. ATR-FTIR (cm^{-1}): 2965, 1678, 1268, 1540, 1105, 683. HRMS m/z (ESI): calcd for $C_{20}H_{35}NPSi$ $(M + H)^+$ 348.2271, found 348.2265.

1-(Di-tert-butylphosphino)-4-methyl-7-(trimethylsilyl)-1H-indole (2c)

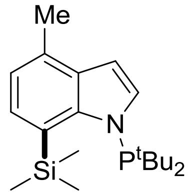

Following the general procedure, the reaction of **1c** (55.0 mg, 0.20 mmol), **I** (146 mg, 1.0 mmol), Pd(OAc)$_2$ (4.48 mg, 10 mol%), DMBQ (95.2 mg, 0.7 mmol) in toluene (0.5 mL) at 120 ℃ under Ar. After 72 h, purification by column chromatography on silica gel (petroleum-ether) yield **2c** (47.8 mg, 69%) as a colorless solid. **^{1}H NMR (500 MHz, Chloroform-*d*)** δ 7.46 – 7.42 (m, 2H), 6.92 (dd, *J* = 7.2, 1.0 Hz, 1H), 6.71 (dd, *J* = 3.4, 1.3 Hz, 1H), 2.53 (s, 3H), 1.21 (d, *J* = 12.4 Hz, 18H), 0.52 (d, *J* = 2.4 Hz, 9H). **^{13}C NMR (126 MHz, Chloroform-*d*)** δ 147.6 (d, *J* = 21.9 Hz), 131.8, 131.7, 130.7 (d, *J* = 5.1 Hz), 128.7 (d, *J* = 3.8 Hz), 120.4 (d, *J*

= 7.3 Hz), 120.2, 104.3 (d, J = 1.9 Hz), 36.3 (d, J = 28.5 Hz), 29.9 (d, J = 15.2 Hz), 18.8, 3.9 (d, J = 13.9 Hz). **^{31}P NMR (162 MHz, CDCl$_3$)** δ 69.3. ATR-FTIR (cm^{-1}): 2895, 1473, 1238, 1515, 1103, 939. $C_{20}H_{35}NPSi$ (M + H)$^+$ 348.2271, found 348.2264.

1-(Di-tert-butylphosphino)-5-methyl-7-(trimethylsilyl)-1H-indole (2d)

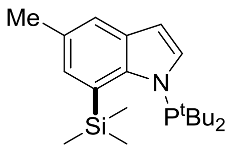

Following the general procedure, the reaction of **1d** (55.0 mg, 0.20 mmol), **I** (146 mg, 1.0 mmol), Pd(OAc)$_2$ (4.48 mg, 10 mol%), DMBQ (95.2 mg, 0.7 mmol) in toluene (0.5 mL) at 120 ℃ under Ar. After 72 h, purification by column chromatography on silica gel (petroleum-ether) yield **2d** (52.1 mg, 75%) as a colorless solid. **^{1}H NMR (500 MHz, Chloroform-*d*)** δ 7.53 – 7.51 (m, 1H), 7.49 (d, J = 3.4 Hz, 1H), 7.45 (d, J = 2.0 Hz, 1H), 6.69 (dd, J = 3.3, 1.3 Hz, 1H), 2.52 (s, 3H), 1.31 (d, J = 12.3 Hz, 18H), 0.64 (d, J = 2.3 Hz, 9H). **^{13}C NMR (126 MHz, Chloroform-*d*)** δ 146.1 (d, J = 22.2 Hz), 133.3, 131.3 (d, J = 5.2 Hz), 129.3 (d, J = 3.5 Hz), 128.4, 123.0 (d, J = 6.8 Hz), 122.3, 105.8 (d, J = 2.2 Hz), 36.3 (d, J = 28.5 Hz), 29.9 (d, J = 15.4 Hz), 21.3, 3.9 (d, J = 13.9 Hz). **^{31}P NMR (162 MHz, CDCl$_3$)** δ 68.8. ATR-FTIR (cm^{-1}): 2798, 1562, 1255, 1033, 658, 579. $C_{20}H_{35}NPSi$ (M + H)$^+$ 348.2271, found 348.2269.

1-(Di-tert-butylphosphino)-3-phenyl-7-(trimethylsilyl)-1H-indole (2e)

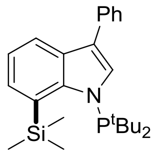

Following the general procedure, the reaction of **1e** (67.4 mg, 0.20 mmol), **I** (146 mg, 1.0 mmol), Pd(OAc)$_2$ (4.48 mg, 10 mol%), DMBQ (95.2 mg, 0.7 mmol) in toluene (0.5 mL) at 120 ^{o}C under Ar. After 72 h, purification by column chromatography on silica gel (petroleum-ether: ethyl acetate = 500:1) yield **2e** (61.3 mg, 75%) as a white solid. **^{1}H NMR (500 MHz, Chloroform-*d*)** δ 7.95 (dt, J = 7.9, 1.3 Hz, 1H), 7.65 (ddd, J = 12.6, 7.8, 1.4 Hz, 3H), 7.57 (s, 1H), 7.49 (dd, J = 8.4, 7.0 Hz, 2H), 7.38 – 7.32 (m, 1H), 7.20 (t, J = 7.5 Hz, 1H), 1.29 (d, J = 12.3 Hz, 18H), 0.60 (d, J = 2.4 Hz, 9H). **^{13}C NMR (126 MHz, Chloroform-*d*)** δ 148.6 (d, J = 21.5 Hz), 135.3, 132.0, 128.9 (d, J = 5.2 Hz), 128.8, 128.1, 127.0 (d, J = 3.5 Hz), 126.3, 123.8 (d, J = 7.1 Hz), 121.12 , 121.06 (d, J = 2.2 Hz), 120.0, 36.3 (d, J = 29.1 Hz), 29.9 (d, J = 15.4 Hz), 3.9 (d, J = 14.0 Hz). **^{31}P NMR (162 MHz, CDCl$_3$)** δ 70.1. ATR-FTIR (cm^{-1}): 2957, 1605, 1473, 1374, 1365, 655. HRMS m/z (ESI): calcd for $C_{25}H_{37}NPSi$ (M + H)$^+$ 410.2427, found 410.2432.

1-(Di-tert-butylphosphino)-4-phenyl-7-(trimethylsilyl)-1H-indole (2f)

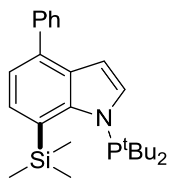

Following the general procedure, the reaction of **1f** (67.4 mg, 0.20 mmol), **I** (146 mg, 1.0 mmol), Pd(OAc)$_2$ (4.48 mg, 10 mol%), DMBQ (95.2 mg, 0.7 mmol) in toluene (0.5 mL) at 120 ℃ under Ar. After 72 h, purification by column chromatography on silica gel (petroleum-ether: ethyl acetate = 500:1) yield **2f** (36.8 mg, 45%) as a white solid. **^{1}H NMR (500 MHz, Chloroform-*d*)** δ 7.68 – 7.65 (m, 2H), 7.62 (d, J = 7.4 Hz, 1H), 7.49 – 7.44 (m, 3H), 7.39 – 7.34 (m, 1H), 7.16 (d, J = 7.4 Hz, 1H), 6.85 (dd, J = 3.5, 1.2 Hz, 1H), 1.24 (d, J = 12.3 Hz, 18H),

0.57 (d, *J* = 2.5 Hz, 9H). **^{13}C NMR (126 MHz, Chloroform-*d*)** δ 148.3 (d, *J* = 22.0 Hz), 141.1, 135.8, 131.8, 131.5 (d, *J* = 4.9 Hz), 129.0, 128.4, 127.2 (d, *J* = 3.5 Hz), 126.9, 122.2 (d, *J* = 7.5 Hz), 119.6, 105.3 (d, *J* = 2.2 Hz), 36.3 (d, *J* = 28.9 Hz), 29.8 (d, *J* = 15.5 Hz), 3.9 (d, *J* = 13.9 Hz). **^{31}P NMR (162 MHz, $CDCl_3$)** δ 64.4. ATR-FTIR (cm^{-1}): 2957, 1507, 1558, 1479, 1365, 752. HRMS m/z (ESI): calcd for $C_{25}H_{37}NPSi$ $(M + H)^+$ 410.2427, found 410.2426.

1-(Di-tert-butylphosphino)-5-phenyl-7-(trimethylsilyl)-1H-indole (2g)

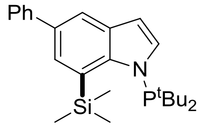

Following the general procedure, the reaction of **1g** (67.4 mg, 0.20 mmol), **I** (146 mg, 1.0 mmol), $Pd(OAc)_2$ (4.48 mg, 10 mol%), DMBQ (95.2 mg, 0.7 mmol) in toluene (0.5 mL) at 120 ℃ under Ar. After 72 h, purification by column chromatography on silica gel (petroleum-ether: ethyl acetate = 500:1) yield **2g** (63.0 mg, 77%) as a white solid. **^{1}H NMR (500 MHz, Chloroform-*d*)** δ 7.93 – 7.90 (m, 1H), 7.88 (d, *J* = 2.0 Hz, 1H), 7.72 (dd, *J* = 8.2, 1.1 Hz, 2H), 7.54 – 7.48 (m, 3H), 7.37 (t, *J* = 7.4 Hz, 1H), 6.80 (dd, *J* = 3.3, 1.3 Hz, 1H), 1.31 (d, *J* = 12.4 Hz, 18H), 0.66 (d, *J* = 2.3 Hz, 9H). **^{13}C NMR (126 MHz, Chloroform-*d*)** δ 147.4 (d, *J* = 21.9 Hz), 142.5, 132.5, 131.9 (d, *J* = 5.1 Hz), 131.5, 129.5, 128.7, 127.3, 126.3, 123.6 (d, *J* = 6.9 Hz), 120.7, 106.6 (d, *J* = 2.2 Hz), 36.3 (d, *J* = 28.7 Hz), 29.8 (d, *J* = 15.3 Hz), 3.9 (d, *J* = 13.6 Hz). **^{31}P NMR (162 MHz, $CDCl_3$)** δ 69.6. ATR-FTIR (cm^{-1}): 2863, 1465, 1140, 951, 753, 636. HRMS m/z (ESI): calcd for $C_{25}H_{37}NPSi$ $(M + H)^+$ 410.2427, found 410.2422.

1-(Di-tert-butylphosphino)-4-methoxy-7-(trimethylsilyl)-1H-indole (2h)

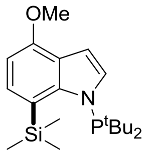

Following the general procedure, the reaction of **1h** (58.2 mg, 0.20 mmol), **I** (146 mg, 1.0 mmol), $Pd(OAc)_2$ (4.48 mg, 10 mol%), DMBQ (95.2 mg, 0.7 mmol) in toluene (0.5 mL) at 120 ℃. After 48 h, purification by column chromatography on silica gel (petroleum-ether: ethyl acetate = 100:1) yield **2h** (36.3 mg, 50%) as a white solid. **^{1}H NMR (500 MHz, Chloroform-*d*)** δ 7.47 (d, *J* = 8.0 Hz, 1H), 7.35 (d, *J* = 3.4 Hz, 1H), 6.81 (dd, *J* = 3.4, 1.3 Hz, 1H), 6.55 (d, *J* = 8.0 Hz, 1H), 3.94 (s, 3H), 1.21 (d, *J* = 12.3 Hz, 18H), 0.51 (d, *J* = 2.3 Hz, 9H). **^{13}C NMR (126 MHz, Chloroform-*d*)** δ 154.5, 149.2 (d, *J* = 22.6 Hz), 132.8, 129.8 (d, *J* = 5.2 Hz), 119.5 (d, *J* = 4.2 Hz), 115.5 (d, *J* = 7.3 Hz), 102.8 (d, *J* = 2.0 Hz), 99.7, 55.1, 36.2 (d, *J* = 28.8 Hz), 29.8 (d, *J* = 15.4 Hz), 3.9 (d, *J* = 13.7 Hz). **^{31}P NMR (162 MHz, $CDCl_3$)** δ 69.7. ATR-FTIR (cm^{-1}): 2987, 1308, 1236, 1181, 757, 699. HRMS m/z (ESI): calcd for $C_{20}H_{35}NOPSi$ $(M + H)^+$ 364.2220, found 364.2223.

1-(Di-tert-butylphosphino)-5-methoxy-7-(trimethylsilyl)-1H-indole (2i)

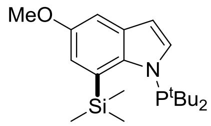

Following the general procedure, the reaction of **1i** (58.2 mg, 0.20 mmol), **I** (146 mg, 1.0 mmol), $Pd(OAc)_2$ (4.48 mg, 10 mol%), DMBQ (95.2 mg, 0.7 mmol) in toluene (0.5 mL) at 120 ℃ under Ar. After 72 h, purification by column chromatography on silica gel (petroleum-ether: ethyl acetate = 100:1) yield **3ai** (45.0 mg, 62%) as a white solid. **^{1}H NMR (500 MHz,**

Chloroform-*d*) δ 7.41 (d, *J* = 3.3 Hz, 1H), 7.18 (d, *J* = 2.7 Hz, 1H), 7.08 (dd, *J* = 2.8, 1.2 Hz, 1H), 6.61 (dd, *J* = 3.3, 1.3 Hz, 1H), 3.84 (s, 3H), 1.21 (d, *J* = 12.3 Hz, 18H), 0.53 (d, *J* = 2.3 Hz, 9H). **^{13}C NMR (126 MHz, Chloroform-*d*)** δ 153.2, 142.5, 131.9 (d, *J* = 5.2 Hz), 129.6, 124.86 (d, *J* = 7.0 Hz), 121.6, 105.9 (d, *J* = 2.1 Hz), 103.3, 55.4, 36.2 (d, *J* = 28.4 Hz), 29.7 (d, *J* = 15.2 Hz), 3.6 (d, *J* = 13.8 Hz). **^{31}P NMR (162 MHz, $CDCl_3$)** δ 72.0. ATR-FTIR (cm^{-1}): 2887, 1589, 1456, 1236, 769, 604. HRMS m/z (ESI): calcd for $C_{20}H_{35}NOPSi$ $(M + H)^+$ 364.2220, found 364.2218.

4-(Benzyloxy)-1-(di-tert-butylphosphino)-7-(trimethylsilyl)-1H-indole (2j)

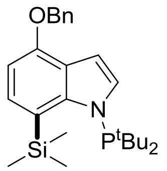

Following the general procedure, the reaction of **1j** (73.4 mg, 0.20 mmol), **I** (146 mg, 1.0 mmol), $Pd(OAc)_2$ (4.48 mg, 10 mol%), DMBQ (95.2 mg, 0.7 mmol) in toluene (0.5 mL) at 120 ℃ under Ar. After 72 h, purification by column chromatography on silica gel (petroleum-ether: ethyl acetate = 100:1) yield **2j** (53.6 mg, 61%) as a white solid. **^{1}H NMR (500 MHz, Chloroform-*d*)** δ 7.51 (d, *J* = 7.2 Hz, 2H), 7.44 – 7.40 (m, 3H), 7.35 (t, *J* = 7.3 Hz, 1H), 7.28 (s, 1H), 7.18 (d, *J* = 1.5 Hz, 1H), 6.62 (dd, *J* = 3.3, 1.3 Hz, 1H), 5.11 (s, 2H), 1.24 (d, *J* = 12.3 Hz, 18H), 0.55 (s, 9H). **^{13}C NMR (101 MHz, Chloroform-*d*)** δ 152.6, 142.8 (d, *J* = 21.9 Hz), 137.8, 131.9 (d, *J* = 5.2 Hz), 129.5 (d, *J* = 3.5 Hz), 128.5, 127.8, 127.6, 124.9 (d, *J* = 7.0 Hz), 122.2, 106.0 (d, *J* = 1.9 Hz), 104.7, 70.4, 36.2 (d, *J* = 28.8 Hz), 29.8 (d, *J* = 15.2 Hz), 3.7 (d, *J* = 13.7 Hz). **^{31}P NMR (162 MHz, $CDCl_3$)** δ 69.7. ATR-FTIR (cm^{-1}): 2678, 1488, 1365, 1289, 1120, 639. HRMS m/z (ESI): calcd for $C_{26}H_{39}NOPSi$ $(M + H)^+$ 440.2533, found 440.2532.

1-(Di-tert-butylphosphino)-3-(phenylthio)-7-(trimethylsilyl)-1H-indole (2k)

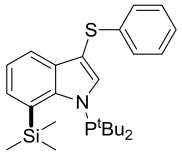

Following the general procedure, the reaction of **1k** (73.8 mg, 0.20 mmol), **I** (146 mg, 1.0 mmol), $Pd(OAc)_2$ (4.48 mg, 10 mol%), DMBQ (95.2 mg, 0.7 mmol) in toluene (0.5 mL) at 120 ℃ under Ar. After 72 h, purification by column chromatography on silica gel (petroleum-ether) yield **3aj** (63.5 mg, 72%) as a white solid. **^{1}H NMR (500 MHz, Chloroform-*d*)** δ 7.72 (s, 1H), 7.62 (t, *J* = 8.0 Hz, 2H), 7.14 (q, *J* = 7.7 Hz, 3H), 7.08 – 7.01 (m, 3H), 1.25 (d, *J* = 12.5 Hz, 18H), 0.56 (d, *J* = 2.4 Hz, 9H). **^{13}C NMR (126 MHz, Chloroform-*d*)** δ 148.5 (d, *J* = 21.5 Hz), 138.9, 137.4 (d, *J* = 5.3 Hz), 132.7, 129.7 (d, *J* = 3.5 Hz), 128.8, 125.8, 124.8, 124.1 (d, *J* = 6.6 Hz), 121.3, 120.6, 105.7 (d, *J* = 3.9 Hz), 36.3 (d, *J* = 29.4 Hz), 29.8 (d, *J* = 15.2 Hz), 3.8 (d, *J* = 13.6 Hz). **^{31}P NMR (162 MHz, $CDCl_3$)** δ 71.2. ATR-FTIR (cm^{-1}): 2998, 2856, 1154, 1028, 654, 389. HRMS m/z (ESI): calcd for $C_{25}H_{37}NPSSi$ $(M + H)^+$ 442.2148, found 442.2145.

1-(Di-tert-butylphosphino)-4-fluoro-7-(trimethylsilyl)-1H-indole (2l)

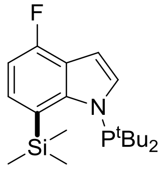

Following the general procedure, the reaction of **1l** (55.8 mg, 0.20 mmol), **I** (146 mg, 1.0 mmol), $Pd(OAc)_2$ (4.48 mg, 10 mol%), DMBQ (95.2 mg, 0.7 mmol) in toluene (0.5 mL) at

120 °C under Ar. After 72 h, purification by column chromatography on silica gel (petroleum-ether) yield **2l** (56.2 mg, 80%) as a white solid. **^{1}H NMR (500 MHz, Chloroform-*d*)** δ 7.45 (dd, *J* = 8.1, 6.5 Hz, 1H), 7.40 (d, *J* = 3.4 Hz, 1H), 6.80 – 6.70 (m, 2H), 1.22 (d, *J* = 12.4 Hz, 18H), 0.52 (d, *J* = 2.3 Hz, 9H). **^{13}C NMR (101 MHz, Chloroform-*d*) δ** 157.7 (d, *J* = 249.3 Hz), 150.4 (d, *J* = 9.2 Hz), 132.3 (d, *J* = 6.8 Hz), 119.3 (d, *J* = 4.3 Hz), 119.2 (d, *J* = 4.2 Hz), 118.0 (d, *J* = 20.4 Hz), 104.4 (d, *J* = 16.2 Hz), 101.5 (d, *J* = 2.2 Hz), 36.3 (d, *J* = 28.8 Hz), 29.7 (d, *J* = 15.3 Hz), 3.8 (d, *J* = 13.7 Hz).**^{31}P NMR (162 MHz, $CDCl_3$)** δ 71.0. **^{19}F NMR (471 MHz, Chloroform-*d*) δ** -121.9 (d, *J* = 10.4 Hz). ATR-FTIR (cm^{-1}): 2879, 1789, 1653, 1178, 1085, 341. HRMS m/z (ESI): calcd for $C_{19}H_{32}FNPSi$ $(M + H)^+$ 352.2020, found 352.2025.

1-(Di-tert-butylphosphino)-5-fluoro-7-(trimethylsilyl)-1H-indole (2m)

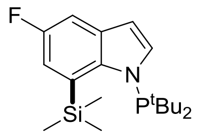

Following the general procedure, the reaction of **1m** (55.8 mg, 0.20 mmol), **I** (146 mg, 1.0 mmol), Pd(OAc)$_2$ (4.48 mg, 10 mol%), DMBQ (95.2 mg, 0.7 mmol) in toluene (0.5 mL) at 120 ℃ under Ar. After 72 h, purification by column chromatography on silica gel (petroleum-ether) yield **2m** (63.9mg, 91%) as a white solid. **^{1}H NMR (500 MHz, Chloroform-*d*)** δ 7.46 (d, *J* = 3.3 Hz, 1H), 7.32 – 7.18 (m, 2H), 6.63 (dd, *J* = 3.4, 1.3 Hz, 1H), 1.21 (d, *J* = 12.4 Hz, 18H), 0.53 (d, *J* = 2.4 Hz, 9H). **^{13}C NMR (126 MHz, Chloroform-*d*)** δ 157.3 (d, *J* = 236.7 Hz), 144.1 (d, *J* = 21.9 Hz), 132.8 (d, *J* = 4.9 Hz), 129.7 (d, *J* = 12.6 Hz), 125.4, 119.1 (d, *J* = 23.7 Hz), 106.7 (d, *J* = 22.4 Hz), 106.1 (d, *J* = 2.4 Hz), 36.3 (d, *J* = 28.7 Hz), 29.7 (d, *J* = 15.1 Hz), 3.6 (d, *J* = 13.9 Hz). **^{31}P NMR (162 MHz, $CDCl_3$)** δ 70.2. **^{19}F NMR (471 MHz, Chloroform-*d*)** δ -126.0 (d, *J* = 10.4 Hz). ATR-FTIR (cm^{-1}): 2959, 1317, 1141, 1111, 777, 702. HRMS m/z (ESI): calcd for $C_{19}H_{32}FNPSi$ $(M + H)^+$ 352.2020, found 352.2020.

4-Chloro-1-(di-tert-butylphosphino)-7-(trimethylsilyl)-1H-indole (2n)

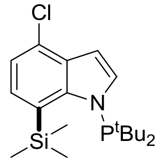

Following the general procedure, the reaction of **1n** (59.0 mg, 0.20 mmol), **I** (146 mg, 1.0 mmol), Pd(OAc)$_2$ (4.48 mg, 10 mol%), DMBQ (95.2 mg, 0.7 mmol) in toluene (0.5 mL) at 120 ℃ under Ar. After 72 h, purification by column chromatography on silica gel (petroleum-ether) yield **2n** (55.1 mg, 75%) as a white solid. **^{1}H NMR (500 MHz, Chloroform-*d*)** δ 7.50 (d, *J* = 3.5 Hz, 1H), 7.47 (d, *J* = 7.8 Hz, 1H), 7.14 (d, *J* = 7.8 Hz, 1H), 6.86 (dd, *J* = 3.4, 1.3 Hz, 1H), 1.25 (d, *J* = 12.5 Hz, 18H), 0.56 (d, *J* = 2.5 Hz, 9H). **^{13}C NMR (126 MHz, Chloroform-*d*)** δ 148.5 (d, *J* = 22.0 Hz), 132.0, 131.8 (d, *J* = 5.1 Hz), 128.0, 127.6 (d, *J* = 3.5 Hz), 122.1 (d, *J* = 6.9 Hz), 119.4, 104.5 (d, *J* = 2.0 Hz), 36.3 (d, *J* = 28.6 Hz), 29.7 (d, *J* = 15.3 Hz), 3.8 (d, *J* = 13.6 Hz). **^{31}P NMR (162 MHz, $CDCl_3$)** δ 71.3. ATR-FTIR (cm^{-1}): 2956, 1549, 1148, 989, 728, 541. HRMS m/z (ESI): calcd for $C_{19}H_{32}ClNPSi$ $(M + H)^+$ 368.1725, found 368.1727.

5-Chloro-1-(di-tert-butylphosphino)-7-(trimethylsilyl)-1H-indole (2o)

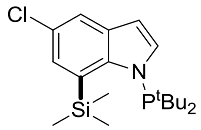

Following the general procedure, the reaction of **1o** (59.0 mg, 0.20 mmol), **I** (146 mg, 1.0 mmol), Pd(OAc)$_2$ (4.48 mg, 10 mol%), DMBQ (95.2 mg, 0.7 mmol) in toluene (0.5

mL) at 120 ℃ under Ar. After 72 h, purification by column chromatography on silica gel (petroleum-ether) yield **2o** (62.4 mg, 85%) as a white solid. **^{1}H NMR (500 MHz, Chloroform-*d*)** δ 7.57 (dd, *J* = 2.3, 1.2 Hz, 1H), 7.44 – 7.43 (m, 2H), 6.60 (dd, *J* = 3.4, 1.3 Hz, 1H), 1.20 (d, *J* = 12.4 Hz, 18H), 0.54 (d, *J* = 2.3 Hz, 9H). **^{13}C NMR (126 MHz, Chloroform-*d*)** δ 146.1 (d, *J* = 21.8 Hz), 132.5 (d, *J* = 5.3 Hz), 131.2, 130.3 (d, *J* = 3.5 Hz), 125.7, 125.6, 121.4, 105.7 (d, *J* = 2.2 Hz), 36.3 (d, *J* = 28.8 Hz), 29.7 (d, *J* = 15.2 Hz), 3.7 (d, *J* = 13.8 Hz). **^{31}P NMR (162 MHz, $CDCl_3$)** δ 70.5. ATR-FTIR (cm^{-1}): 2897, 2220, 1594, 1344, 1211, 1127. HRMS m/z (ESI): calcd for $C_{19}H_{32}ClNPSi$ $(M + H)^+$ 368.1725, found 368.1718.

1-(Di-tert-butylphosphino)-5-(trifluoromethyl)-7-(trimethylsilyl)-1H-indole (2p)

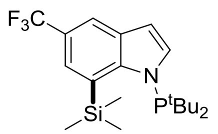

Following the general procedure, the reaction of **1p** (65.8 mg, 0.20 mmol), **I** (146 mg, 1.0 mmol), $Pd(OAc)_2$ (4.48 mg, 10 mol%), DMBQ (95.2 mg, 0.7 mmol) in toluene (0.5 mL) at 120 ℃ under Ar. After 72 h, purification by column chromatography on silica gel (petroleum-ether) yield **2p** (48.9 mg, 61%) as a white solid. **^{1}H NMR (500 MHz, Chloroform-*d*)** δ 7.96 – 7.92 (m, 1H), 7.78 (d, *J* = 2.1 Hz, 1H), 7.56 (d, *J* = 3.4 Hz, 1H), 6.79 (dd, *J* = 3.4, 1.3 Hz, 1H), 1.23-1.25 (d, *J* = 12.5 Hz, 18H), 0.60 (d, *J* = 2.4 Hz, 9H). **^{13}C NMR (126 MHz, Chloroform-*d*)** δ 149.2 (d, *J* = 21.7 Hz), 132.8 (d, *J* = 5.1 Hz), 128.4 (d, *J* = 3.5 Hz), 127.9 (q, *J* = 3.5 Hz), 125.5 (q, *J* = 3.5 Hz), 124.3 (d, *J* = 7.1 Hz), 119.7 (q, *J* = 4.1 Hz), 106.8 (d, *J* = 2.3 Hz), 36.3 (d, *J* = 28.8 Hz), 29.7 (d, *J* = 15.4 Hz), 3.6 (d, *J* = 13.9 Hz). **^{31}P NMR (162 MHz, $CDCl_3$)** δ 71.1. **^{19}F NMR (471 MHz, Chloroform-*d*)** δ -60.5. ATR-FTIR (cm^{-1}): 2957, 1469, 1361, 1149, 890, 831. HRMS m/z (ESI): calcd for $C_{20}H_{32}F_3NPSi$ $(M + H)^+$ 402.1988, found 402.1987.

1-(1-(Di-tert-butylphosphino)-7-(trimethylsilyl)-1H-indol-3-yl)ethanone (2q)

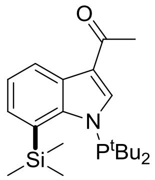

Following the general procedure, the reaction of **1q** (60.6 mg, 0.20 mmol), **I** (146 mg, 1.0 mmol), $Pd(OAc)_2$ (4.48 mg, 10 mol%), DMBQ (95.2 mg, 0.7 mmol) in toluene (0.5 mL) at 120 ℃ under Ar. After 72 h, purification by column chromatography on silica gel (petroleum-ether: ethyl acetate = 20:1) yield **2q** (60.0 mg, 80%) as a white solid. **^{1}H NMR (500 MHz, Chloroform-*d*)** δ 8.50 (dd, *J* = 7.8, 1.3 Hz, 1H), 8.08 (s, 1H), 7.62 (dd, *J* = 7.3, 1.4 Hz, 1H), 7.28 – 7.24 (m, 1H), 2.57 (s, 3H), 1.26 (d, *J* = 12.5 Hz, 18H), 0.54 (d, *J* = 2.4 Hz, 9H). **^{13}C NMR (126 MHz, Chloroform-*d*)** δ 192.7, 148.1 (d, *J* = 20.6 Hz), 138.4 (d, *J* = 5.1 Hz), 132.7, 125.7 (d, *J* = 2.8 Hz), 123.4, 121.7, 119.9 (d, *J* = 2.6 Hz), 116.8, 35.9 (d, *J* = 30.0 Hz), 29.2 (d, *J* = 15.4 Hz), 27.3, 3.3 (d, *J* = 13.7 Hz). **^{31}P NMR (162 MHz, $CDCl_3$)** δ 74.0. ATR-FTIR (cm^{-1}): 2958, 1758, 1526, 1209, 1148, 1062. HRMS m/z (ESI): calcd for $C_{21}H_{35}NOPSi$ $(M + H)^+$ 376.2220, found 376.2221.

Methyl 1-(di-tert-butylphosphino)-7-(trimethylsilyl)-1H-indole-5-carboxylate (2r)

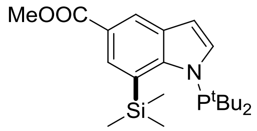

Following the general procedure, the reaction of **1r** (63.8 mg, 0.20 mmol), **I** (146 mg, 1.0 mmol), $Pd(OAc)_2$ (4.48 mg, 10 mol%), DMBQ (95.2 mg, 0.7 mmol) in

toluene (0.5 mL) at 120 ℃ under Ar. After 72 h, purification by column chromatography on silica gel (petroleum-ether: ethyl acetate = 20:1) yield **2r** (48.5 mg, 62%) as a white solid. **^{1}H NMR (500 MHz, Chloroform-*d*)** δ 8.35 (t, *J* = 1.6 Hz, 1H), 8.26 (d, *J* = 1.9 Hz, 1H), 7.48 (d, *J* = 3.4 Hz, 1H), 6.76 (dd, *J* = 3.4, 1.3 Hz, 1H), 3.92 (s, 3H), 1.22 (d, *J* = 12.4 Hz, 18H), 0.56 (d, *J* = 2.4 Hz, 9H). **^{13}C NMR (126 MHz, Chloroform-*d*)** δ 168.3, 150.5 (d, *J* = 21.4 Hz), 132.9, 132.5 (d, *J* = 5.1 Hz), 128.7 (d, *J* = 3.5 Hz), 124.8, 123.4 (d, *J* = 7.2 Hz), 121.3, 107.2 (d, *J* = 2.3 Hz), 51.8, 36.3 (d, *J* = 29.0 Hz), 29.7 (d, *J* = 15.4 Hz), 3.6 (d, *J* = 13.7 Hz). **^{31}P NMR (162 MHz, $CDCl_3$)** δ 71.6. ATR-FTIR (cm^{-1}): 2879, 2875, 1718, 1256, 1084, 895. HRMS m/z (ESI): calcd for $C_{21}H_{35}NO_2PSi$ $(M + H)^+$ 392.2169, found 392.2169.

Ethyl 2-(1-(di-tert-butylphosphino)-7-(trimethylsilyl)-1H-indol-3-yl) acetate (2s)

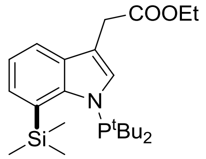

Following the general procedure, the reaction of **1s** (69.4 mg, 0.20 mmol), **I** (146 mg, 1.0 mmol), $Pd(OAc)_2$ (4.48 mg, 10 mol%), DMBQ (95.2 mg, 0.7 mmol) in toluene (0.5 mL) at 120 ℃ under Ar. After 72 h, purification by column chromatography on silica gel (petroleum-ether: ethyl acetate = 20:1) yield **2s** (43.6 mg, 52%) as a white solid. **^{1}H NMR (500 MHz, Chloroform-*d*)** δ 7.60 (dt, *J* = 7.7, 1.3 Hz, 1H), 7.55 (dd, *J* = 7.3, 1.3 Hz, 1H), 7.44 (s, 1H), 7.13 (t, *J* = 7.5 Hz, 1H), 4.15 (q, *J* = 7.1 Hz, 2H), 3.76 (d, *J* = 0.9 Hz, 2H), 1.23 – 1.20 (m, 21H), 0.55 – 0.51 (m, 9H). **^{13}C NMR (126 MHz, Chloroform-*d*)** δ 171.8, 148.0 (d, *J* = 21.5 Hz), 131.7, 130.0 (d, *J* = 5.3 Hz), 128.3, 123.5 (d, *J* = 6.9 Hz), 120.1, 119.5, 111.4 (d, *J* = 2.3 Hz), 60.7, 36.2 (d, *J* = 28.8 Hz), 31.4, 29.8 (d, *J* = 15.5 Hz), 14.2, 3.7 (d, *J* = 13.9 Hz). **^{31}P NMR (162 MHz, $CDCl_3$)** δ 73.1. ATR-FTIR (cm^{-1}): 2987, 1756, 1458, 1165, 1097, 650. HRMS m/z (ESI): calcd for $C_{23}H_{39}NO_2PSi$ $(M + H)^+$ 420.2482, found 420.2474.

1-(Di-tert-butylphosphino)-7-(trimethylsilyl)-1H-indole-3-carbonitrile (2t)

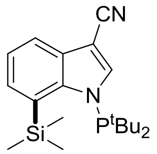

Following the general procedure, the reaction of **1t** (57.2 mg, 0.20 mmol), **I** (146 mg, 1.0 mmol), $Pd(OAc)_2$ (4.48 mg, 10 mol%), DMBQ (95.2 mg, 0.7 mmol) in toluene (0.5 mL) at 120 ℃ under Ar. After 72 h, purification by column chromatography on silica gel (petroleum-ether: ethyl acetate = 50:1) yield **2t** (50.1 mg, 70%) as a white solid. **^{1}H NMR (500 MHz, Chloroform-*d*)** 7.95 (s, 1H), 7.79 (d, *J* = 7.7 Hz, 1H), 7.66 (dd, *J* = 7.3, 1.4 Hz, 1H), 7.26 (t, *J* = 7.5 Hz, 1H), 1.23 (d, *J* = 12.6 Hz, 18H), 0.54 (d, *J* = 2.3 Hz, 9H). **^{13}C NMR (126 MHz, Chloroform-*d*)** δ 146.8 (d, *J* = 20.5 Hz), 138.5 (d, *J* = 5.4 Hz), 133.8, 127.6 (d, *J* = 2.7 Hz), 125.0 (d, *J* = 6.5 Hz), 121.9, 121.2, 115.6, 90.7 (d, *J* = 3.1 Hz), 36.3 (d, *J* = 30.0 Hz), 29.5 (d, *J* = 15.4 Hz), 3.7 (d, *J* = 13.5 Hz). **^{31}P NMR (162 MHz, $CDCl_3$)** δ 75.6. ATR-FTIR (cm^{-1}): 2956, 2650, 1589, 1441, 1289, 855. HRMS m/z (ESI): calcd for $C_{20}H_{32}N_2PSi$ $(M + H)^+$ 359.2067, found 359.2061.

1-(Di-tert-butylphosphino)-7-(trimethylsilyl)-1H-indole-4-carbonitrile (2u)

Following the general procedure, the reaction of **1u** (57.2 mg, 0.20 mmol), **I** (146 mg, 1.0 mmol), $Pd(OAc)_2$ (4.48 mg, 10 mol%), DMBQ (95.2 mg, 0.7 mmol) in toluene (0.5 mL) at 120 ℃ under Ar. After 72 h, purification by column chromatography on silica gel (petroleum-ether: ethyl acetate = 50:1) yield **2u** (58.7 mg, 82%) as a white solid.

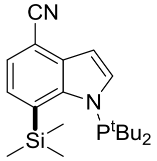

^{1}H NMR (500 MHz, Chloroform-*d*) δ 7.60 (d, *J* = 3.4 Hz, 1H), 7.56 (d, *J* = 7.5 Hz, 1H), 7.42 (d, *J* = 7.5 Hz, 1H), 6.93 (dd, *J* = 3.4, 1.3 Hz, 1H), 1.22 (d, *J* = 12.5 Hz, 18H), 0.55 (d, *J* = 2.4 Hz, 9H). **^{13}C NMR (126 MHz, Chloroform-*d*)** δ 147.4 (d, *J* = 21.9 Hz), 133.8 (d, *J* = 5.0 Hz), 130.9, 130.3 (d, *J* = 6.9 Hz), 130.0 (d, *J* = 3.5 Hz), 124.1, 118.7, 104.8 (d, *J* = 2.3 Hz), 104.4, 36.3 (d, *J* = 28.7 Hz), 29.7 (d, *J* = 15.5 Hz), 3.5 (d, *J* = 13.8 Hz). **^{31}P NMR (162 MHz, $CDCl_3$)** δ 78.9. ATR-FTIR (cm^{-1}): 2959, 2222, 1473, 1152, 1008, 926. HRMS m/z (ESI): calcd for $C_{20}H_{32}N_2PSi$ $(M + H)^+$ 359.2067, found 359.2064.

1-(Di-tert-butylphosphino)-7-(trimethylsilyl)-1H-indole-5-carbonitrile (2v)

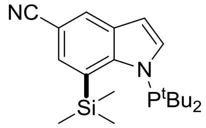

Following the general procedure, the reaction of **1v** (57.2 mg, 0.20 mmol), **I** (146 mg, 1.0 mmol), $Pd(OAc)_2$ (4.48 mg, 10 mol%), DMBQ (95.2 mg, 0.7 mmol) in toluene (0.5 mL) at 120 ℃ under Ar. After 72 h, purification by column chromatography on silica gel (petroleum-ether: ethyl acetate = 50:1) yield **2v** (65.2 mg, 91%) as a white solid. **^{1}H NMR (500 MHz, Chloroform-*d*)** δ 7.98 (t, *J* = 1.5 Hz, 1H), 7.76 (d, *J* = 1.8 Hz, 1H), 7.56 (d, *J* = 3.4 Hz, 1H), 6.77 (dd, *J* = 3.4, 1.3 Hz, 1H), 1.24 (d, *J* = 12.6 Hz, 18H), 0.58 (d, *J* = 2.5 Hz, 9H). **^{13}C NMR (126 MHz, Chloroform-*d*)** δ 149.4 (d, *J* = 21.6 Hz), 134.3, 133.3 (d, *J* = 4.9 Hz), 128.7, 127.1, 125.4 (d, *J* = 7.3 Hz), 121.0, 106.6 (d, *J* = 2.3 Hz), 103.1, 36.3 (d, *J* = 29.0 Hz), 29.6 (d, *J* = 15.3 Hz), 3.5 (d, *J* = 13.9 Hz). **^{31}P NMR (162 MHz, $CDCl_3$)** δ 72.1. ATR-FTIR (cm^{-1}): 2957, 2220, 1594, 1270, 875, 829. HRMS m/z (ESI): calcd for $C_{20}H_{32}N_2PSi$ $(M + H)^+$ 359.2067, found 359.2066.

(E)-1-(Di-tert-butylphosphino)-5-styryl-7-(trimethylsilyl)-1H-indole (2w)

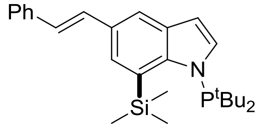

Following the general procedure, the reaction of **1w** (72.6 mg, 0.20 mmol), **I** (146 mg, 1.0 mmol), $Pd(OAc)_2$ (4.48 mg, 10 mol%), DMBQ (95.2 mg, 0.7 mmol) in toluene (0.5 mL) at 120 ℃ under Ar. After 72 h, purification by column chromatography on silica gel (petroleum-ether) yield **2w** (61.8 mg, 71%) as a white solid. **^{1}H NMR (500 MHz, Chloroform-*d*)** δ 7.75 (d, *J* = 23.1 Hz, 2H), 7.53 (d, *J* = 7.4 Hz, 2H), 7.42 (d, *J* = 3.3 Hz, 1H), 7.34 (t, *J* = 7.7 Hz, 2H), 7.23 (d, *J* = 16.0 Hz, 2H), 7.07 (d, *J* = 16.3 Hz, 1H), 6.68 (dd, *J* = 3.3, 1.3 Hz, 1H), 1.22 (d, *J* = 12.4 Hz, 18H), 0.58 (d, *J* = 2.4 Hz, 9H). **^{13}C NMR (126 MHz, Chloroform-*d*)** δ 147.7 (d, *J* = 21.6 Hz), 138.2, 131.9 (d, *J* = 5.0 Hz), 131.0, 130.0, 129.5 (d, *J* = 3.5 Hz), 128.8, 128.6, 127.0, 126.3, 126.2, 123.6 (d, *J* = 7.0 Hz), 120.3, 106.5 (d, *J* = 2.3 Hz), 36.3 (d, *J* = 28.9 Hz), 29.8 (d, *J* = 15.2 Hz), 3.8 (d, *J* = 13.8 Hz). **^{31}P NMR (162 MHz, $CDCl_3$)** δ 70.0. ATR-FTIR (cm^{-1}): 2899, 2489, 2370, 2059, 1152, 568. HRMS m/z (ESI): calcd for $C_{27}H_{39}NPSi$ $(M + H)^+$ 436.2584, found 436.2586.

1-(Di-tert-butylphosphino)-5-(phenylethynyl)-7-(trimethylsilyl)-1H-indole (2x)

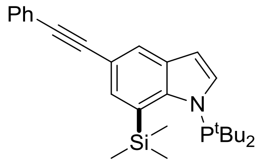

Following the general procedure, the reaction of **1x** (72.2 mg, 0.20 mmol), **I** (146 mg, 1.0 mmol), $Pd(OAc)_2$ (4.48 mg, 10 mol%), DMBQ (95.2 mg, 0.7 mmol) in toluene (0.5 mL) at 120 ℃ under Ar. After 72 h, purification by column chromatography on silica gel (petroleum-ether) yield **2x** (71.0 mg, 82%) as a white solid. **^{1}H NMR (500 MHz, Chloroform-*d*)** δ 7.83 (s, 1H), 7.69 (d, *J* = 1.4 Hz, 1H), 7.57 – 7.52 (m, 2H), 7.44 (d, *J* = 3.4 Hz, 1H), 7.36 – 7.27 (m, 3H), 6.66 (dd, *J* = 3.4, 1.3 Hz, 1H), 1.21 (d, *J* = 12.4 Hz, 18H), 0.56 (d, *J* = 2.4 Hz, 9H). **^{13}C NMR (126 MHz, Chloroform-*d*)** δ 147.5 (d, *J* = 21.7 Hz), 135.1, 132.1 (d, *J* = 5.0 Hz), 131.6, 129.0 (d, *J* = 3.4 Hz), 128.3, 127.7, 125.8, 124.1, 123.9 (d, *J* = 7.0 Hz), 114.2, 106.3 (d, *J* = 2.3 Hz), 91.1, 87.3, 36.3 (d, *J* = 28.9 Hz), 29.8 (d, *J* = 15.3 Hz), 3.7 (d, *J* = 13.9 Hz). **^{31}P NMR (162 MHz, $CDCl_3$)** δ 70.6. ATR-FTIR (cm^{-1}): 2997, 2564, 1588, 1463, 997, 541. HRMS m/z (ESI): calcd for $C_{27}H_{37}NPSi$ $(M + H)^+$ 434.2427, found 434.2426.

Late-Stage Modification

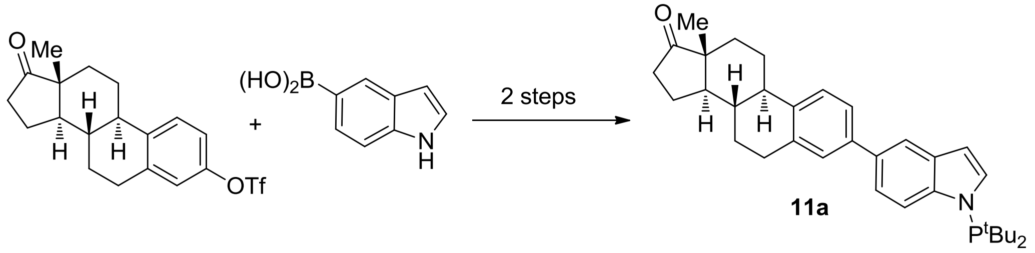

Step 1: Trifluoromethanesulfonate (5.0 mmol), 5-indoleboronic acid (6.0 mmol), Pd $(PPh_3)_4$ (5.0 mol%), K_3PO_4 (3.0 equiv.) and 1,2-dimethoxyethane (5.0 ml) was stirred in a nitrogen atmosphere at 85 ℃ for 24 hours. After left cooled, the reaction mixture was diluted with ethyl acetate, filtered through Celite, and the filtrate was concentrated under reduced pressure. The residue was purified through silica gel column chromatography (petroleum-ether: ethyl acetate = 20: 1) to obtain the title compound as a colorless solid.

Step 2: To a solution of indole (2.0 mmol, 1.0 equiv) in 10 ml anhydrous THF at -40 ℃ was added a solution of LHMDS (solution in THF, 3.0 mmol, 1.5 equiv) dropwise. After stirring for 1 h, di-tert-butylchlorophosphine (3.0 mmol, 1.5 equiv) was added dropwise. The mixture was allowed to stir and warm to room temperature over several hours. After indole was consumed determined by TLC, the reaction was quenched by 2 mL MeOH. Then the solvent was removed under reduced pressure. Further purification through flash chromatography (petroleum-ether/ethyl acetate) can provide the pure product **11a**.

(8R,9S,13S,14S)-3-(1-(di-tert-butylphosphino)-1H-indol-5-yl)-13-methyl-7,8,9,11,12,13,15,16-octahydro-6H-cyclopenta[a]phenanthren-17(14H)-one (11a)

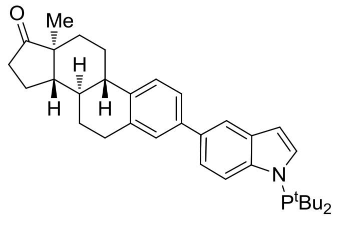

The crude product **11a** was purified using flash column chromatography on silica gel (petroleum-ether: ethyl acetate = 20:1) yield **11a** (554 mg, 54%) as a

white solid (the total yield of the multiple steps). **^{1}H NMR (500 MHz, Chloroform-*d*)** δ 7.89 (dd, *J* = 8.6, 2.3 Hz, 1H), 7.77 (s, 1H), 7.48 – 7.42 (m, 3H), 7.39 – 7.32 (m, 2H), 6.69 – 6.66 (m, 1H), 2.98 (dd, *J* = 8.8, 3.9 Hz, 2H), 2.53 – 2.31 (m, 3H), 2.20 – 1.96 (m, 4H), 1.67 – 1.45 (m, 6H), 1.22 (d, *J* = 12.7 Hz, 18H), 0.91 (s, 3H). **^{13}C NMR (126 MHz, Chloroform-*d*)** δ 220.9, 143.3 (d, *J* = 21.2 Hz), 140.1, 137.8, 136.6, 133.4, 131.3 (d, *J* = 8.7 Hz), 129.3 (d, *J* = 3.1 Hz), 128.0, 125.7, 124.9, 121.6 (d, *J* = 1.8 Hz), 118.5, 113.1 (d, *J* = 19.9 Hz), 105.7 (d, *J* = 2.0 Hz), 50.6, 48.1, 44.4, 38.3, 35.9, 35.3 (d, *J* = 25.3 Hz), 31.7, 29.6, 29.2 (d, *J* = 16.3 Hz), 26.7, 25.8, 21.7, 13.9. **^{31}P NMR (162 MHz, $CDCl_3$)** δ 72.2. ATR-FTIR (cm^{-1}): 2876, 2470, 1720, 1514, 1340, 1157. HRMS m/z (ESI): calcd for $C_{34}H_{45}NOP$ $(M + H)^+$ 514.3233, found 514.3238.

(8S,9R,13R,14R)-3-(1-(di-tert-butylphosphino)-7-(trimethylsilyl)-1H-indol-5-yl)-13-methyl-7,8,9,11,12,13,15,16-octahydro-6H-cyclopenta[a]phenanthren-17(14H)-one (12a)

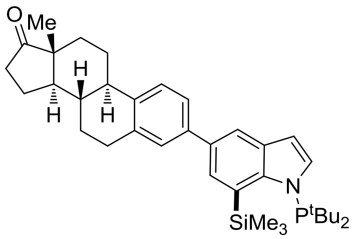

Following the general procedure, the reaction of **11a** (102.6 mg, 0.20 mmol), **I** (146 mg, 1.0 mmol), $Pd(OAc)_2$ (4.48 mg, 10 mol%), DMBQ (95.2 mg, 0.7 mmol) in toluene (0.5 mL) at 120 ℃ under Ar. After 72 h, purification by column chromatography on silica gel (petroleum-ether: ethyl acetate = 10:1) yield **12a** (73.7mg, 63%) as a colorless oil. **^{1}H NMR (500 MHz, Chloroform-*d*)** δ 7.79 (d, *J* = 16.9 Hz, 2H), 7.47 – 7.42 (m, 2H), 7.37 (d, *J* = 8.0 Hz, 2H), 6.72 (dt, *J* = 3.3, 1.6 Hz, 1H), 3.01 (dd, *J* = 8.6, 3.8 Hz, 2H), 2.54 – 2.34 (m, 3H), 2.18 – 1.97 (m, 4H), 1.66 – 1.48 (m, 6H), 1.24 (d, *J* = 12.3 Hz, 18H), 0.93 (s, 3H), 0.57 (d, *J* = 2.2 Hz, 9H). **^{13}C NMR (126 MHz, Chloroform-*d*)** δ 221.0, 147.3 (d, *J* = 22.0 Hz), 140.2, 137.8, 136.6, 132.4, 131.8 (d, *J* = 5.1 Hz), 131.4, 129.5 (d, *J* = 3.7 Hz), 128.0, 125.7, 124.8, 123.5 (d, *J* = 6.9 Hz), 120.6, 106.5 (d, *J* = 2.2 Hz), 50.6, 48.1, 44.4, 38.3, 36.3 (d, *J* = 28.8 Hz), 35.9, 31.7, 29.8 (d, *J* = 15.3 Hz), 29.7, 26.7, 25.8, 21.7 13.9, 3.8 (d, *J* = 13.8 Hz). **^{31}P NMR (162 MHz, $CDCl_3$)** δ 72.2. ATR-FTIR (cm^{-1}): 2876, 2470, 1745, 1502, 1340, 651. HRMS m/z (ESI): calcd for $C_{37}H_{53}NOPSi$ $(M + H)^+$ 586.3629, found 586.3628.

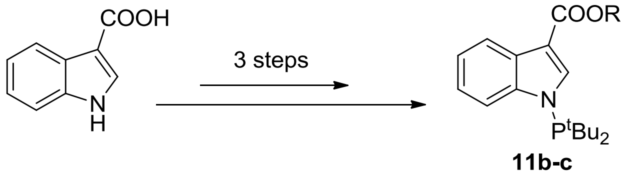

11b-c

Step 1: Thionyl chloride (2 equiv.) was added to a suspension 3-Indoleformic acid (10 mmol) in anhydrous CH_2Cl_2 (30 mL), followed by a catalytic amount of anhydrous DMF (2 drops). The suspension was stirred at reflux for 6 h. Removal of the solvent in vacuo gave the crude acid chloride as yellow oil.

Step 2: Alcohols (10 mmol), NEt_3 (2 equiv.) and DMAP (0.2 equiv.) was dissolved in DCM (20 mL). The solution was cooled to 0 ℃. Then the crude acid chloride in DCM (20 mL) was added. The solution was stirred at room temperature for 12 h. The solvent was removed under reduced pressure and the crude product

was purified using flash column chromatography on silica gel (petroleum-ether/ethyl acetate).

Step 3: To a solution of indole (2.0 mmol, 1.0 equiv) in 10 ml anhydrous THF at -40 ℃ was added a solution of LHMDS (solution in THF, 3.0 mmol, 1.5 equiv) dropwise. After stirring for 1 h, di-tert-butylchlorophosphine (3.0 mmol, 1.5 equiv) was added dropwise. The mixture was allowed to stir and warm to room temperature over several hours. After indole was consumed determined by TLC, the reaction was quenched by 2 mL MeOH. Then the solvent was removed under reduced pressure. Further purification through flash chromatography (petroleum-ether/ethyl acetate) can get the pure products **11b-c**.

(3S,8S,9S,10R,13S,14S,17S)-17-acetyl-10,13-dimethyl-2,3,4,7,8,9,10,11,12,13,14,15,16,17-tetradecahydro-1H-cyclopenta[a]phenanthren-3-yl-1-(di-tert-butylphosphino)-1H-indole-3-carboxylate (11b)

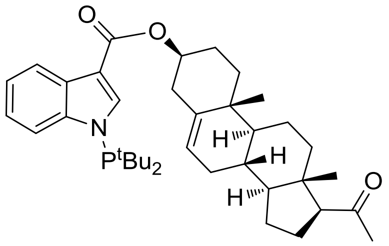

Following the general procedure, the crude product **11b** was purified using flash column chromatography on silica gel (petroleum-ether: ethyl acetate = 20:1) yield **11b** (663.3 mg, 55%) as a white solid (the total yield of the multiple steps). **^{1}H NMR (500 MHz, Chloroform-*d*)** δ 8.17 – 8.10 (m, 2H), 7.88 (dt, *J* = 7.3, 2.2 Hz, 1H), 7.26 (t, *J* = 4.2 Hz, 2H), 5.47 – 5.40 (m, 1H), 4.91 (dd, *J* = 11.2, 5.6 Hz, 1H), 2.54 (t, *J* = 7.7 Hz, 3H), 2.19 (d, *J* = 9.7 Hz, 1H), 2.13 (s, 3H), 2.09 – 1.81 (m, 5H), 1.69 – 1.43 (m, 8H), 1.22 (d, *J* = 12.9 Hz, 20H), 1.10 (s, 3H), 0.95 – 0.87 (m, 1H), 0.65 (s, 3H). **^{13}C NMR (126 MHz, Chloroform-*d*)** δ 209.5, 164.7, 144.2 (d, *J* = 20.3 Hz), 140.0, 137.5 (d, *J* = 9.1 Hz), 126.3 (d, *J* = 2.6 Hz), 122.9 (d, *J* = 1.8 Hz), 122.3, 122.1, 121.2, 113.5 (d, *J* = 20.3 Hz), 111.8 (d, *J* = 2.4 Hz), 73.5, 63.7, 56.9, 49.9, 44.0, 38.6, 37.2, 36.7, 35.2, 35.0, 31.9 (d, *J* = 3.9 Hz), 31.6, 29.0 (d, *J* = 16.5 Hz), 28.2, 24.5, 22.9, 21.1, 19.5, 13.3. **^{31}P NMR (162 MHz, $CDCl_3$)** δ 76.2. ATR-FTIR (cm^{-1}): 2879, 2486, 1746, 1565, 1360, 960. HRMS m/z (ESI): calcd for $C_{38}H_{55}NO_3P$ $(M + H)^+$ 604.3914, found 604.3920.

(3S,8S,9S,10R,13S,17S)-17-acetyl-10,13-dimethyl-2,3,4,7,8,9,10,11,12,13,14,15,16,17-tetradecahydro-1H-cyclopenta[a]phenanthren-3-yl-1-(di-tert-butylphosphino)-7-(trimethylsilyl)-1H-indole-3-carboxylate (12b)

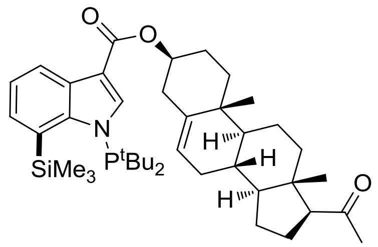

Following the general procedure, the reaction of **11b** (120.6 mg, 0.20 mmol), **I** (146 mg, 1.0 mmol), $Pd(OAc)_2$ (4.48 mg, 10 mol%), DMBQ (95.2 mg, 0.7 mmol) in toluene (0.5 mL) at 120 ℃ under Ar. After 72 h, purification by column chromatography on silica gel (petroleum-ether: ethyl acetate = 10:1) yield **12b** (91.8 mg, 68%) as a colorless oil. **^{1}H NMR (500 MHz, Chloroform-*d*)** δ 8.27 (d, *J* = 7.8 Hz, 1H), 8.14 (s, 1H), 7.60 (dd, *J* = 7.3, 1.2 Hz, 1H), 7.24 (d, *J* = 7.6 Hz, 1H), 5.43 (d, *J* = 5.0 Hz, 1H), 4.96 – 4.84 (m, 1H), 2.59 – 2.48 (m, 3H), 2.24 – 2.14 (m, 1H), 2.13 (s, 3H), 2.09 – 1.91 (m, 4H), 1.81 – 1.45 (m, 9H), 1.24 (d, *J* = 12.5 Hz, 18H), 1.09 (d, *J* = 5.0 Hz, 4H), 0.99 – 0.76 (m, 2H), 0.65 (s, 3H), 0.53 (d, *J* = 2.4 Hz, 9H). **^{13}C NMR (126**

MHz, Chloroform-*d*) δ 209.6, 164.5, 140.0, 138.2 (d, *J* = 5.1 Hz), 132.6, 126.4 (d, *J* = 2.8 Hz), 124.1, 124.0, 123.1, 122.2, 121.4, 111.7, 73.4, 63.7, 56.9, 50.0, 44.0, 38.8, 38.6, 37.2, 36.7, 36.3, 36.0, 31.9, 31.7 (d, *J* = 25.9 Hz), 29.6 (d, *J* = 15.4 Hz), 28.2, 24.5, 22.9, 21.1, 19.5, 13.3, 3.8 (d, *J* = 13.9 Hz). **^{31}P NMR (162 MHz, $CDCl_3$)** δ 73.7. ATR-FTIR (cm^{-1}): 2877, 2504, 1700, 1563, 1025, 886. HRMS m/z (ESI): calcd for $C_{41}H_{63}NO_3PSi$ $(M + H)^+$ 676.4309, found 676.4296.

(2'R,4S,5'R,6aR,6bS,8aS,8bR,9S,11aS,12aS,12bS)-5',6a,8a,9-tetramethyl-1,3,3',4,4',5,5',6,6a,6b,6',7,8,8a, 8b,9,11a,12,12a,12b-icosahydrospiro[naphtho[2',1':4,5]indeno[2,1-b]furan-10,2'-pyran]-4-yl 1-(di-tert-butylphosphino)-1H-indole-3-carboxylate (11c)

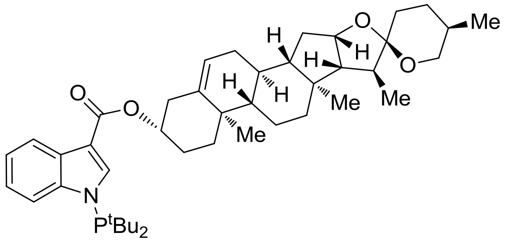

Following the general procedure, the crude product **11c** was purified using flash column chromatography on silica gel (petroleum-ether: ethyl acetate = 50:1) yield **11c** (630.9 mg, 45%) as a white solid (the total yield of the multiple steps). **^{1}H NMR (500 MHz, Chloroform-*d*)** δ 8.19 – 8.13 (m, 2H), 7.91 (dt, *J* = 6.9, 2.2 Hz, 1H), 7.30 – 7.27 (m, 2H), 5.48 – 5.45 (m, 1H), 4.98 – 4.88 (m, 1H), 4.46 (q, *J* = 7.4 Hz, 1H), 3.54 – 3.47 (m, 1H), 3.41 (t, *J* = 10.9 Hz, 1H), 2.58 (q, *J* = 9.5, 8.6 Hz, 2H), 2.11 – 1.50 (m, 18H), 1.37 – 1.30 (m, 2H), 1.25 (d, *J* = 12.9 Hz, 18H), 1.15 (s, 3H), 1.11 – 1.06 (m, 1H), 1.01 (d, *J* = 7.0 Hz, 3H), 0.88 (d, *J* = 1.1 Hz, 1H), 0.84 – 0.80 (m, 6H). **^{13}C NMR (126 MHz, Chloroform-*d*)** δ 164.7, 144.3 (d, *J* = 20.2 Hz), 140.0, 137.5 (d, *J* = 9.1 Hz), 126.3 (d, *J* = 2.4 Hz), 122.9 (d, *J* = 1.8 Hz), 122.3, 122.1, 121.2, 113.4 (d, *J* = 20.3 Hz), 111.9 (d, *J* = 2.4 Hz), 109.3, 80.8, 73.6, 66.9, 62.1, 56.5, 50.0, 41.6, 40.3, 39.8, 38.6, 37.2, 36.9, 35.2, 35.0, 32.1, 31.9, 31.5 (d, *J* = 6.0 Hz), 30.3, 29.1 (d, *J* = 16.5 Hz), 28.9, 28.2, 26.9, 20.9, 19.5, 17.2, 16.3, 14.6. **^{31}P NMR (162 MHz, $CDCl_3$)** δ 76.2. ATR-FTIR (cm^{-1}): 2765, 2256, 1987, 1687, 1540, 978. HRMS m/z (ESI): calcd for $C_{44}H_{65}NO_4P$ $(M + H)^+$ 702.4646, found 702.4657.

(2'R,4S,5'R,6aR,6bS,8aS,8bR,9S,11aS,12aS,12bS)-5',6a,8a,9-tetramethyl-1,3,3',4,4',5,5',6,6a,6b,6',7,8,8a, 8b,9,11a,12,12a,12b-icosahydrospiro[naphtho[2',1':4,5]indeno[2,1-b]furan-10,2'-pyran]-4-yl 1-(di-tert-butylphosphino)-7-(trimethylsilyl)-1H-indole-3-carboxylate (12c)

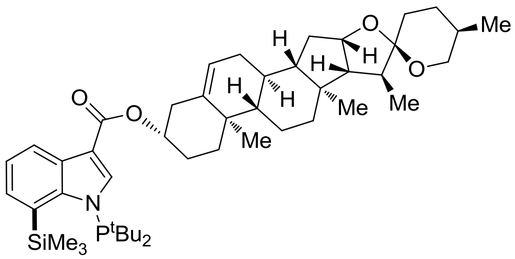

Following the general procedure, the reaction of **11c** (140.2 mg, 0.20 mmol), **I** (146 mg, 1.0 mmol), $Pd(OAc)_2$ (4.48 mg, 10 mol%), DMBQ (95.2 mg, 0.7 mmol) in toluene (0.5 mL) at 120 ℃ under Ar. After 72 h, purification by column chromatography on silica gel (petroleum-ether: ethyl acetate = 50:1) yield **12c** (94.3 mg, 61%) as a colorless oil. **^{1}H NMR (500 MHz, Chloroform-*d*)** δ 8.27 (d, *J* = 7.8 Hz, 1H), 8.14 (s, 1H), 7.59 (d, *J* = 6.4 Hz, 1H), 7.23 (t, *J* = 7.6 Hz, 1H), 5.43 (d, *J* = 4.8 Hz, 1H), 4.89 (dt, *J* = 10.9, 5.6 Hz, 1H), 4.46 – 4.40 (m, 1H), 3.52 – 3.37 (m, 2H), 2.57 – 2.48 (m, 2H), 2.08 – 1.49 (m, 20H), 1.36 – 1.30 (m, 1H), 1.23 (d, *J* = 12.5 Hz, 18H), 1.12 (s, 3H), 0.98

(d, J = 6.8 Hz, 3H), 0.83 – 0.77 (m, 7H), 0.53 (d, J = 2.4 Hz, 9H). **^{13}C NMR (126 MHz, Chloroform-*d*)** δ 164.5, 148.4 (d, J = 20.7 Hz), 140.0, 138.2 (d, J = 5.2 Hz), 132.6, 126.4 (d, J = 3.2 Hz), 124.0 (d, J = 6.8 Hz), 123.1, 122.3, 121.4, 111.8 (d, J = 2.5 Hz), 109.3, 80.8, 73.5, 66.9, 62.1, 56.5, 50.0, 41.6, 40.3, 39.8, 38.6, 37.1, 36.9, 36.3, 36.0, 32.1, 31.9, 31.5, 31.4, 30.3, 29.6 (d, J = 15.4 Hz), 28.8, 28.2, 20.9, 19.5, 17.2, 16.3, 14.6, 3.8 (d, J = 14.0 Hz). **^{31}P NMR (162 MHz, $CDCl_3$)** δ 73.6. ATR-FTIR (cm^{-1}): 2860, 2250, 1707, 1687, 1536, 560. HRMS m/z (ESI): calcd for $C_{47}H_{73}NO_4PSi$ $(M + H)^+$ 774.5041, found 774.5022.

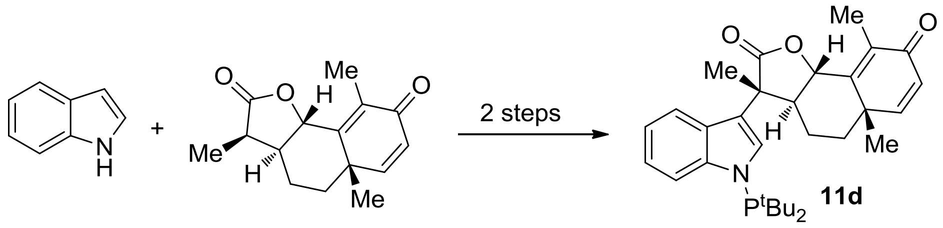

Step 1: According to the literature,[21] to a solution of carbonyl compound (1 equiv.) and indole (2 equiv.) in THF (1.0 M) at –78 ℃ in a flame-dried flask under a nitrogen atmosphere was added a 1.0 M solution of LHMDS (3.3 equiv.). After stirring for 30 minutes at –78 ℃, the septum was removed, solid copper (II)2-ethylhexanoate (1.5 equiv.) was rapidly added in one portion, and the septum quickly replaced. The flask was then removed from the cooling bath and allowed to warm to room temperature. Once the flask reached ambient temperature, the reaction was partitioned between 1 N HCl and EtOAc. The organic layer was further washed with 1 N NaOH, water, then brine and dried (MgSO4). The solvent was removed in vacuo and the crude product purified by flash column chromatography (silica gel).

Step 2: To a solution of indole (2.0 mmol, 1.0 equiv) in 10 ml anhydrous THF at -40 ℃ was added a solution of LHMDS (solution in THF, 3.0 mmol, 1.5 equiv) dropwise. After stirring for 1 h, di-tert-butylchlorophosphine (3.0 mmol, 1.5 equiv) was added dropwise. The mixture was allowed to stir and warm to room temperature over several hours. After indole was consumed determined by TLC, the reaction was quenched by 2 mL MeOH. Then the solvent was removed under reduced pressure. Further purification through flash chromatography (petroleum-ether/ethyl acetate) can generate the pure product **11d**.

(3R,3aS,5aS,9bS)-3-(1-(di-tert-butylphosphino)-1H-indol-3-yl)-3,5a,9-trimethyl-3a,4,5,5a-tetrahydronaphtho[1,2-b] furan-2,8(3H,9bH)-dione (11d)

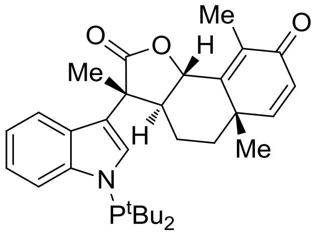

Following the general procedure, the crude product **11d** was purified using flash column chromatography on silica gel (petroleum-ether: ethyl acetate = 20:1) yield **11d** (323.2 mg, 32%) as a white solid (the total yield of the multiple steps). **^{1}H NMR (500 MHz, Chloroform-*d*) δ** 7.93 (dd, J = 8.3, 2.7 Hz, 1H), 7.64 (d, J = 8.0 Hz, 1H), 7.25 (t, J = 7.3 Hz, 1H), 7.20 (s,1H), 7.14 (t, J = 7.2 Hz, 1H), 6.60 (d, J = 9.8 Hz, 1H), 6.20 (d, J = 9.8 Hz, 1H), 4.85 (d, J = 12.8 Hz, 1H), 2.30 – 2.25 (m, 1H), 2.22 (s, 3H), 2.08 (d, J = 14.9 Hz, 1H), 1.83 (s, 3H), 1.76 (d, J = 10.1 Hz, 1H), 1.35 (d, J =

10.7 Hz, 1H), 1.19 (dd, J = 20.2, 12.8 Hz, 18H), 0.94 (s, 3H), 0.87 – 0.76 (m, 1H). **^{13}C NMR (126 MHz, Chloroform-*d*) δ** 186.2, 179.0, 154.8, 151.6, 144.8 (d, J = 20.5 Hz), 129.1, 129.0, 126.8 (d, J = 2.8 Hz), 125.9, 122.4 (d, J = 1.9 Hz), 120.3 (d, J = 5.0 Hz), 114.0, 113.8, 113.3 (d, J = 2.5 Hz), 79.5, 58.2, 47.8, 41.2, 37.7, 35.4 (d, J = 9.4 Hz), 35.1 (d, J = 8.2 Hz), 29.2 (d, J = 3.2 Hz), 29.0 (d, J = 3.2 Hz), 24.5, 21.9, 21.6, 11.1. **^{31}P NMR (162 MHz, $CDCl_3$)** δ 72.2. ATR-FTIR (cm^{-1}): 2798, 2658, 1720, 1025, 654, 530. HRMS m/z (ESI): calcd for $C_{31}H_{41}NO_3P$ $(M + H)^+$ 506.2819, found 506.2835.

(3R,3aS,5aS,9bS)-3-(1-(di-tert-butylphosphino)-7-(trimethylsilyl)-1H-indol-3-yl)-3,5a,9-trimethyl-3a,4,5,5a-tetrahydronaphtho[1,2-b] furan-2,8(3H,9bH)-dione (12d)

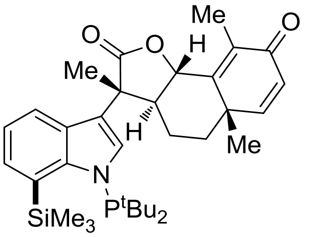

Following the general procedure, the reaction of **11d** (101 mg, 0.20 mmol), **I** (146 mg, 1.0 mmol), $Pd(OAc)_2$ (4.48 mg, 10 mol%), DMBQ (95.2 mg, 0.7 mmol) in toluene (0.5 mL) at 120 ^{o}C under Ar. After 72 h, purification by column chromatography on silica gel (petroleum-ether: ethyl acetate = 10:1) yield **12d** (87.7 mg, 76%) as a colorless oil. **^{1}H NMR (500 MHz, Chloroform-*d*)** δ 7.74 (d, J = 8.0 Hz, 1H), 7.64 (d, J = 7.2 Hz, 1H), 7.24 (s, 1H), 7.17 (t, J = 7.6 Hz, 1H), 6.63 (d, J = 9.8 Hz, 1H), 6.25 (d, J = 9.8 Hz, 1H), 4.87 (dq, J = 11.7, 1.5 Hz, 1H), 2.31 (dd, J = 11.9, 3.5 Hz, 1H), 2.25 (s, 3H), 2.16 – 2.10 (m, 1H), 1.86 (s, 3H), 1.78 (s, 1H), 1.53 – 1.40 (m, 2H), 1.27 – 1.18 (m, 18H), 0.99 (s, 3H), 0.58 (d, J = 2.4 Hz, 9H). **^{13}C NMR (126 MHz, Chloroform-*d*)** δ 186.2, 179.1, 154.9, 151.7, 148.9 (d, J = 21.1 Hz), 132.1, 129.8 (d, J = 5.0 Hz), 129.1, 126.8 (d, J = 3.3 Hz), 125.9, 122.2, 119.6, 117.3, 113.3 (d, J = 2.7 Hz), 79.5, 58.2, 47.9, 41.2, 37.8, 36.7, 29.8 (dd, J = 15.3, 12.9 Hz), 24.5, 21.9, 21.6，11.1, 3.9 (d, J = 14.0 Hz). **^{31}P NMR (162 MHz, $CDCl_3$)** δ 69.5. ATR-FTIR (cm^{-1}): 2798, 2658, 1748, 1055, 756, 630. HRMS m/z (ESI): calcd for $C_{34}H_{49}NO_3PSi$ $(M + H)^+$ 578.3214, found 578.3219.

Downstream Transformations

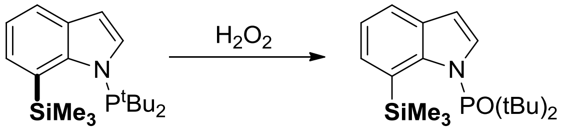

2a **4**

1-(Di-tert-butylphosphino)-7-(trimethylsilyl)-1H-indole (4)

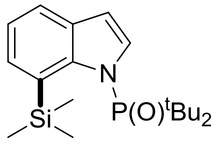

The reaction of **2a** (66.6 mg, 0.20 mmol), H_2O_2 (1 mL) in THF (0.5 mL) at room temperature under air. After 72 h, purification by column chromatography on silica gel (petroleum-ether) yield **4** (69.1 mg, 99%) as a white solid. **^{1}H NMR (500 MHz, Chloroform-*d*)** δ 7.66 (dd, J = 7.3, 1.4 Hz, 1H), 7.61 (dt, J = 7.6, 1.3 Hz, 1H), 7.32 – 7.28 (m, 1H), 7.20 (t, J = 7.5 Hz, 1H), 6.74 (dd, J = 3.7, 1.3 Hz, 1H), 1.27 (d, J = 14.5 Hz, 18H), 0.49 (s, 9H). **^{13}C NMR (126 MHz, Chloroform-*d*)** δ 145.8, 133.6, 130.3 (d, J = 4.9 Hz), 128.2, 127.4 (d, J = 6.0 Hz), 121.8,

121.5, 108.5 (d, J = 6.2 Hz), 39.5 (d, J = 67.5 Hz), 27.8, 4.3. **^{31}P NMR (162 MHz, $CDCl_3$)** δ 61.4.

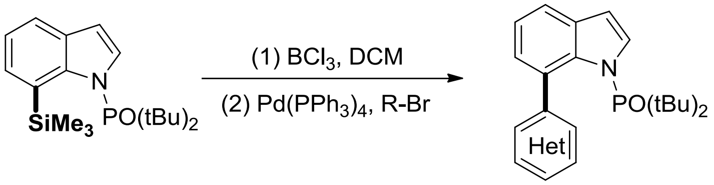

According to the literature,[22] a solution of BCl_3 (1.0 M, 0.48 mL, 0.24 mmol) in CH_2Cl_2 was added by syringe under N_2 to a stirred solution of indolesilane **4** (70.6 mg, 0.2 mmol) in CH_2Cl_2 (1.0 mL) at 0 ℃. The mixture was stirred at room temperature for 3 h, after which time the solvent was removed *in vacuo*. After the residue was dried under high vacuum for 2 h, **14a-c** (0.24 mmol, 1.2 equiv.), $Pd(PPh_3)_4$ (11.55 mg, 5 mol%), DME (2 mL, degassed) and 2M Na_2CO_3 aqueous solution (0.5 mL, degassed) were added and the mixture was stirred under reflux for 24 h. Then the reaction mixture was cooled to room temperature and water (10 mL) was added. The mixture was extracted with Et_2O (3 x 15 mL), the combined organic extracts were washed with brine, dried over Na_2SO_4 and concentrated. The desired product was obtained after purification by silica gel flash chromatography (petroleum-ether /ethyl acetate).

Di-tert-butyl(7-(pyridin-3-yl)-1H-indol-1-yl) phosphine oxide (15a)

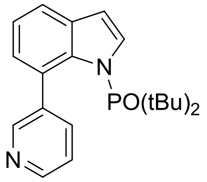

Following the above procedure, The desired product (31.8 mg, 45% yield) was obtained as a white solid after purification by silica gel flash chromatography (petroleum-ether: ethyl acetate = 1:1). **^{1}H NMR (500 MHz, Chloroform-*d*)** δ 8.20 – 8.15 (m, 2H), 7.67 (d, J = 7.8 Hz, 1H), 7.41 – 7.39 (m, 1H), 7.34 (d, J = 7.9 Hz, 1H), 7.31 – 7.27 (m, 2H), 7.08 (dd, J = 7.3, 1.0 Hz, 1H), 6.84 (d, J = 3.6 Hz, 1H), 1.20 (s, 18H). **^{13}C NMR (126 MHz, Chloroform-*d*)** δ 143.3, 139.1, 136.7, 129.3 (d, J = 5.3 Hz), 127.5, 126.9, 125.1, 124.71, 122.1, 121.6, 108.8 (d, J = 5.2 Hz), 27.1. **^{31}P NMR (162 MHz, $CDCl_3$)** δ 64.0. ATR-FTIR (cm^{-1}): 2997, 2450, 1453, 1365, 1025, 883. HRMS m/z (ESI): calcd for $C_{21}H_{28}N_2OP$ $(M + H)^+$ 355.1934, found 355.1931.

Di-tert-butyl(7-(furan-2-yl)-1H-indol-1-yl) phosphine oxide (15b)

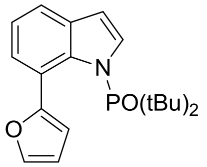

Following the above procedure, the desired product (42.5 mg, 62% yield) was obtained as a white solid after purification by silica gel flash chromatography (petroleum-ether: ethyl acetate = 10:1). **^{1}H NMR (400 MHz, Chloroform-*d*)** δ 7.57 (dt, J = 7.6, 1.2 Hz, 1H), 7.40 – 7.38 (m, 2H), 7.30 (dd, J = 7.3, 1.4 Hz, 1H), 7.20 (t, J = 7.5 Hz, 1H), 6.79 (dd, J = 3.6, 1.0 Hz, 1H), 6.51 (dd, J = 3.2, 1.8 Hz, 1H), 6.42 (dd, J = 3.3, 0.9 Hz, 1H), 1.23 (d, J = 14.6 Hz, 18H). **^{13}C NMR (126 MHz, Chloroform-*d*)** δ 156.8, 140.3, 139.1, 132.3 (d, J = 3.5 Hz), 129.4 (d, J = 4.8 Hz), 127.7, 122.0, 121.2, 121.0, 111.4, 108.8 (d, J = 5.4 Hz), 104.5, 40.1 (d, J = 67.7 Hz), 27.3. **^{31}P NMR (162 MHz, $CDCl_3$)** δ 63.1. ATR-FTIR (cm^{-1}): 2995, 2568, 1533, 1456, 1369, 892. HRMS m/z (ESI): calcd for

$C_{20}H_{27}NO_2P(M + H)^+$ 344.1774, found 344.1774

Di-tert-butyl(7-(thiophen-2-yl)-1H-indol-1-yl) phosphine oxide (15c)

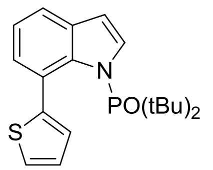

Following the above procedure, the desired product (46.7 mg, 65% yield) was obtained as a white solid after purification by silica gel flash chromatography (petroleum-ether: ethyl acetate = 10:1). **^{1}H NMR (400 MHz, Chloroform-*d*)** δ 7.57 (dt, *J* = 7.6, 1.1 Hz, 1H), 7.40 – 7.37 (m, 1H), 7.30 (dd, *J* = 5.1, 1.2 Hz, 1H), 7.26 (d, *J* = 1.4 Hz, 1H), 7.19 (t, *J* = 7.5 Hz, 1H), 7.01 (dd, *J* = 5.1, 3.5 Hz, 1H), 6.95 (dd, *J* = 3.5, 1.2 Hz, 1H), 6.78 (dd, *J* = 3.6, 0.9 Hz, 1H), 1.19 (d, *J* = 14.6 Hz, 18H). **^{13}C NMR (126 MHz, Chloroform-*d*)** δ 145.4, 140.3, 132.2 (d, *J* = 3.6 Hz), 129.4 (d, *J* = 5.6 Hz), 129.1, 126.0, 125.6, 124.1, 123.9, 121.7, 120.5, 108.5 (d, *J* = 5.5 Hz), 40.1 (d, *J* = 67.5 Hz), 27.4. **^{31}P NMR (162 MHz, $CDCl_3$)** δ 62.3. ATR-FTIR (cm^{-1}): 2985, 2650, 2345, 1675, 1265, 984. HRMS m/z (ESI): calcd for $C_{20}H_{27}NOPS$ $(M + H)^+$ 360.1545, found 360.1546.

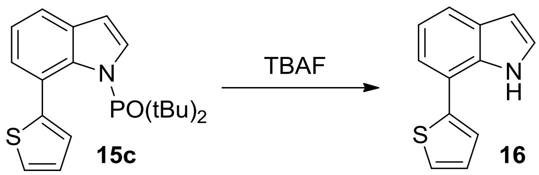

Di-tert-butyl(7-(thiophen-2-yl)-1H-indol-1-yl) phosphine oxide **15c** (0.2 mmol), TBAF (1 M in THF, 0.4 mL) and THF (2.0 mL) were added in a test tube under air atmosphere. After this, the mixture was stirred at 100 ^{o}C for 24 h. After the reaction was completed (monitored **16** by TLC), the resulting mixture was extracted with ethyl acetate. The combined organic layers were evaporated under vacuum. The desired product **16** was obtained in 70% (27.9 mg) yield after purified by column chromatography on silica gel with a mixture of petroleum ether/ethyl acetate (v/v = 20/1). **^{1}H NMR (500 MHz, Chloroform-*d*)** δ 8.61 (s, 1H), 7.62 (dt, *J* = 7.9, 1.0 Hz, 1H), 7.37 – 7.31 (m, 3H), 7.21 (d, *J* = 2.7 Hz, 1H), 7.18 – 7.13 (m, 2H), 6.61 (dd, *J* = 3.2, 2.1 Hz, 1H). **^{13}C NMR (126 MHz, Chloroform-*d*)** δ 141.3, 133.2, 128.6, 128.0, 124.9, 124.6, 124.5, 121.9, 120.6, 120.2, 118.4, 103.4.

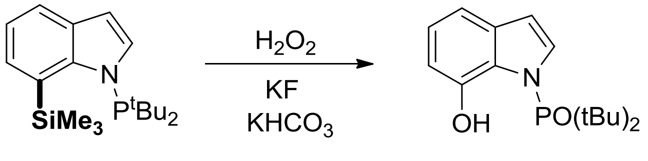

According to the literature,[23] the reaction of **2a** (66.6 mg, 0.20 mmol), H_2O_2 (2.0 mL), KF (46.4 mg, 0.8 mmol), $KHCO_3$ (80.0 mg, 0.8 mmol) in THF (1.0 mL) at 40°C. After 72 h, purification by column chromatography on silica gel (petroleum-ether: ethyl acetate = 1:1) yield **13** (48.0 mg, 82%) as a colorless oil. **^{1}H NMR (400 MHz, DMSO-*d*$_6$)** δ 11.74 (d, *J* = 1.4 Hz, 1H), 7.55 (dd, *J* = 3.6, 2.3 Hz, 1H), 7.08 – 6.99 (m,

2H), 6.80 (dd, J = 3.6, 2.1 Hz, 1H), 6.64 (dd, J = 7.5, 1.5 Hz, 1H), 1.29 (d, J = 15.3 Hz, 18H). **^{13}C NMR (126 MHz, DMSO-d_6)** δ 145.0, 132.8 (d, J = 4.7 Hz), 129.4, 128.1 (d, J = 5.9 Hz), 123.6, 111.5, 111.3, 109.0 (d, J = 5.8 Hz), 38.6 (d, J = 66.9 Hz), 26.4. **^{31}P NMR (162 MHz, CDCl$_3$)** δ 68.8. ATR-FTIR (cm^{-1}): 3050, 2958, 2256, 1798, 1569, 1287, 556. HRMS m/z (ESI): calcd for $C_{16}H_{25}NO_2P$ (M + H)$^+$ 294.1617, found 294.1618.

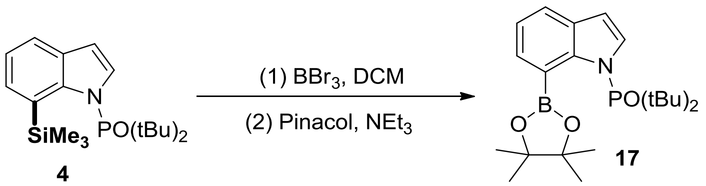

4 → 17

According to the literature,[24] a solution of BBr_3 (1.0 M, 0.48 mL, 0.24 mmol) in CH_2Cl_2 was added by syringe under N_2 to a stirred solution of indolesilane **4** (70.6 mg, 0.2 mmol) in CH_2Cl_2 (1.0 mL) at 0 ℃. The mixture was stirred at room temperature for 2 h. And Pinacol (47.2 mg, 0.4 mmol) and NEt_3 (101 mg, 1.0 mmol) were added and the mixture was stirred for 1 h. It was then concentrated and purified by silica gel chromatography (petroleum-ether : ethyl acetate = 1:1) to afford the desired product **17** (46.3 mg, 79% yield). **^{1}H NMR (400 MHz, Chloroform-*d*)** δ 7.49 – 7.41 (m, 2H), 7.19 (dd, J = 7.8, 7.1 Hz, 1H), 7.08 – 7.04 (m, 1H), 6.72 (t, J = 3.1 Hz, 1H), 1.35 (d, J = 15.8 Hz, 30H). **^{13}C NMR (101 MHz, Chloroform-*d*)** δ 144.4, 128.6, 126.8 (d, J = 6.4 Hz), 123.2, 123.1, 123.0, 119.1, 110.1 (d, J = 5.6 Hz), 80.3, 38.6 (d, J = 66.6 Hz), 26.6, 25.8. **^{31}P NMR (162 MHz, CDCl$_3$)** δ 69.4. ATR-FTIR (cm^{-1}): 2986, 2665, 1547, 1498, 1029, 673. HRMS m/z (ESI): calcd for $C_{22}H_{36}BNO_3P$ (M + H)$^+$ 404.2520, found 404.2528.

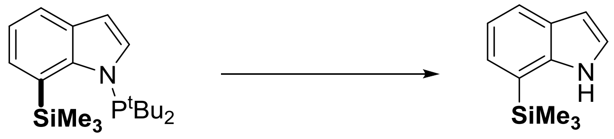

2a → 18

7-(Trimethylsilyl)-1H-indole (3aa)

The reaction of **2a** (66.6 mg, 0.20 mmol), diisobutylaluminium hydride (8 equiv., 1.5 M/in toluene) in THF (2.0 mL) at 100 ℃ under Ar. After 12 h, purification by column chromatography on silica gel (petroleum-ether: ethyl acetate = 50:1) yield **18** (27.2 mg, 72%) as a colorless oil. **^{1}H NMR (500 MHz, Chloroform-*d*)** δ 8.18 (s, 1H), 7.68 (d, J = 8.7 Hz, 1H), 7.33 – 7.29 (m, 1H), 7.23 (d, J = 3.5 Hz, 1H), 7.14 (td, J = 7.8, 2.7 Hz, 1H), 6.59 – 6.56 (m, 1H), 0.42 (d, J = 2.4 Hz, 9H). **^{13}C NMR (126 MHz, Chloroform-*d*)** δ 138.8, 127.0, 125.6, 122.9, 121.0, 119.6, 118.6, 101.6, -1.5. ATR-FTIR (cm^{-1}): 3102, 2798, 2054, 1779, 1026, 945. HRMS m/z (ESI): calcd for $C_{11}H_{16}NSi$ (M + H)$^+$ 190.1047, found 190.1046.

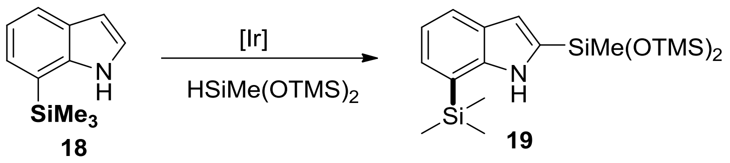

According to the literature,[25] the reaction of **18** (37.8 mg, 0.20 mmol), HSiMe(OTMS)$_2$ (133.2 mg, 0.60 mmol), [{Ir(OMe)(cod)}$_2$] (6.62 mg, 5 mol%), 4,4‐di‐*tert*‐butyl‐2,2‐bipyridine (5.36 mg, 10 mol%), norbornene (55.2 mg, 0.60 mmol) in THF (0.5 mL) at 80 ℃. After 24 h, purification by column chromatography on silica gel (petroleum-ether: ethyl acetate = 50:1) yield **19** (63.0 mg, 77%) as a colorless oil. **^{1}H NMR (500 MHz, Chloroform-*d*)** δ 8.25 (s, 1H), 7.67 (d, *J* = 7.9 Hz, 1H), 7.31 (dd, *J* = 6.9, 1.2 Hz, 1H), 7.12 – 7.08 (m, 1H), 6.75 (d, *J* = 2.0 Hz, 1H), 0.43 (s, 9H), 0.35 (s, 3H), 0.14 (s, 18H). **^{13}C NMR (126 MHz, Chloroform-*d*)** δ 140.6, 134.6, 127.3, 126.1, 121.0, 119.4, 118.2, 109.9, 0.6, -0.7, -1.7. ATR-FTIR (cm^{-1}): 3072, 2561, 2133, 1659, 1068 995. HRMS m/z (ESI): calcd for $C_{18}H_{36}NO_2Si_4$ (M + H)$^+$ 410.1818, found 410.1819.

C-H Silylation of Carbazoles

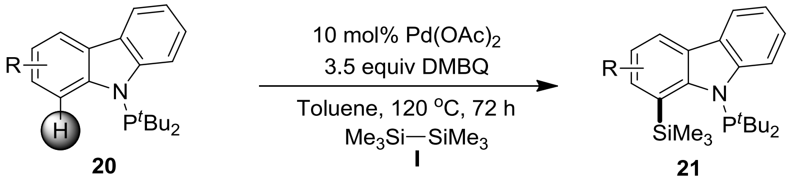

The reaction of **20** (0.20 mmol), **I** (146 mg, 1.0 mmol), Pd(OAc)$_2$ (4.48 mg, 10 mol%), DMBQ (95.2 mg, 0.7 mmol) in toluene (0.5 mL) at 120 ^{o}C under Ar. After 72 h, purification by column chromatography on silica gel (petroleum-ether) yield **21.**

9-(Di-tert-butylphosphino)-1-(trimethylsilyl)-9H-carbazole (21a)

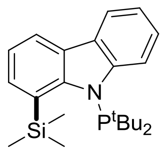

Following the general procedure, the reaction of **20a** (62.2 mg, 0.20 mmol), **I** (146 mg, 1.0 mmol), Pd(OAc)$_2$ (4.48 mg, 10 mol%), DMBQ (95.2 mg, 0.7 mmol) in toluene (0.5 mL) at 120 ℃ under Ar. After 72 h, purification by column chromatography on silica gel (petroleum-ether) yield **21a** (45.2 mg, 59%) as a white solid. **^{1}H NMR (500 MHz, Chloroform-*d*)** δ 8.05 (td, *J* = 7.6, 1.2 Hz, 2H), 7.87 (d, *J* = 8.3 Hz, 1H), 7.75 (dt, *J* = 7.2, 1.2 Hz, 1H), 7.37 (ddt, *J* = 8.2, 7.0, 1.1 Hz, 1H), 7.25 – 7.18 (m, 2H), 1.28 (d, *J* = 12.8 Hz, 18H), 0.57 (d, *J* = 2.6 Hz, 9H). **^{13}C NMR (126 MHz, Chloroform-*d*)** δ 153.5, 143.2 (d, *J* = 9.3 Hz), 135.2 (d, *J* = 1.9 Hz), 127.0 (d, *J* = 2.4 Hz), 124.8, 124.7, 124.6, 121.0, 120.5, 119.7, 119.5, 115.9, 37.3 (d, *J* = 34.3 Hz), 30.5 (d, *J* = 16.1 Hz), 4.2 (d, *J* = 15.2 Hz). **^{31}P NMR (162 MHz, $CDCl_3$)** δ 78.9. ATR-FTIR (cm^{-1}): 2952, 1580, 1389, 1175, 1118, 959. HRMS m/z (ESI): calcd for $C_{23}H_{35}NPSi$ (M + H)$^+$ 384.2271, found 384.2276.

3,6-Di-tert-butyl-9-(di-tert-butylphosphino)-1-(trimethylsilyl)-9H-carbazole (21b)

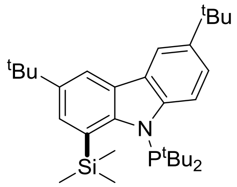

Following the general procedure, the reaction of **20b** (84.6 mg, 0.20 mmol), **I** (146 mg, 1.0 mmol), $Pd(OAc)_2$ (4.48 mg, 10 mol%), DMBQ (95.2 mg, 0.7 mmol) in toluene (0.5 mL) at 120 ℃ under Ar. After 72 h, purification by column chromatography on silica gel (petroleum-ether) yield **21b** (72.3 mg, 73%) as a white solid. **^{1}H NMR (500 MHz, Chloroform-*d*)** δ 8.02 (q, *J* = 2.5, 2.0 Hz, 2H), 7.80 (t, *J* = 2.3 Hz, 1H), 7.75 (dd, *J* = 8.8, 1.9 Hz, 1H), 7.39 (dt, *J* = 8.8, 2.1 Hz, 1H), 1.44 (s, 18H), 1.28 (dd, *J* = 12.8, 1.9 Hz, 18H), 0.57 (t, *J* = 2.8 Hz, 9H). **^{13}C NMR (126 MHz, Chloroform-*d*)** δ 151.8 (d, *J* = 27.4 Hz), 143.2, 141.6, 141.4 (d, *J* = 9.8 Hz), 133.3, 126.8 (d, *J* = 2.5 Hz), 124.5 (d, *J* = 4.3 Hz), 123.6 (d, *J* = 12.3 Hz), 122.3, 116.9, 115.4, 115.4, 37.2 (d, *J* = 34.1 Hz), 34.7 (d, *J* = 5.9 Hz), 32.0 (d, *J* = 3.8 Hz), 30.6 (d, *J* = 16.2 Hz), 4.3 (d, *J* = 15.4 Hz). **^{31}P NMR (162 MHz, $CDCl_3$)** δ 77.1. ATR-FTIR (cm^{-1}): 2960, 1544, 1405, 1256, 1178, 965. HRMS m/z (ESI): calcd for $C_{31}H_{51}NPSi$ $(M + H)^+$ 496.3523, found 496.3523.

9-(Di-tert-butylphosphino)-3,6-diphenyl-1-(trimethylsilyl)-9H-carbazole (21c)

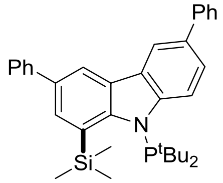

Following the general procedure, the reaction of **20c** (92.6 mg, 0.20 mmol), **1** (146 mg, 1.0 mmol), $Pd(OAc)_2$ (4.48 mg, 10 mol%), DMBQ (95.2 mg, 0.7 mmol) in toluene (0.5 mL) at 120 ℃ under Ar. After 72 h, purification by column chromatography on silica gel (petroleum-ether) yield **21c** (66.3 mg, 62%) as a white solid. **^{1}H NMR (500 MHz, Chloroform-*d*)** δ 8.40 – 8.36 (m, 2H), 8.11 (d, *J* = 1.9 Hz, 1H), 8.00 (d, *J* = 8.6 Hz, 1H), 7.80 (d, *J* = 8.0 Hz, 4H), 7.72 (dd, *J* = 8.6, 1.8 Hz, 1H), 7.54 (t, *J* = 7.6 Hz, 4H), 7.40 (t, *J* = 7.3 Hz, 2H), 1.40 (d, *J* = 12.9 Hz, 18H), 0.69 (d, *J* = 3.1 Hz, 9H). **^{13}C NMR (126 MHz, Chloroform-*d*)** δ 153.5 (d, *J* = 27.1 Hz), 143.2 (d, *J* = 9.6 Hz), 141.9, 141.6, 135.0 (d, *J* = 1.8 Hz), 133.9, 132.6, 128.8, 127.6 (d, *J* = 2.3 Hz), 127.2, 127.2, 126.7, 126.6, 125.4 (d, *J* = 4.0 Hz), 125.3, 125.1, 124.2, 119.3, 117.9, 116.2, 37.4 (d, *J* = 34.4 Hz), 30.6 (d, *J* = 16.0 Hz), 4.3 (d, *J* = 15.3 Hz). **^{31}P NMR (162 MHz, $CDCl_3$)** δ 79.4. ATR-FTIR (cm^{-1}): 2978, 1586, 1562, 1355, 1077, 955. HRMS m/z (ESI): calcd for $C_{35}H_{43}NPSi$ $(M + H)^+$ 536.2897, found 536.2896.

3,6-Dichloro-9-(di-tert-butylphosphino)-1-(trimethylsilyl)-9H-carbazole (21d)

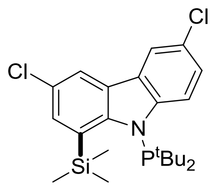

Following the general procedure, the reaction of **20d** (75.8 mg, 0.20 mmol), **1** (146 mg, 1.0 mmol), $Pd(OAc)_2$ (4.48 mg, 10 mol%), DMBQ (95.2 mg, 0.7 mmol) in toluene (0.5 mL) at 120 ℃ under Ar. After 72 h, purification by column chromatography on silica gel (petroleum-ether) yield **21d** (36.9 mg, 41%) as a white solid. **^{1}H NMR (500 MHz, Chloroform-*d*)** δ 7.93 (dd, *J* = 4.1, 1.8 Hz, 2H), 7.76 (d, *J* = 8.9 Hz, 1H), 7.66 (d, *J* = 2.3 Hz, 1H), 7.35 (dd, *J* = 8.8, 2.2 Hz, 1H), 1.26 (d, *J* = 12.9 Hz, 18H), 0.56 (d, *J* = 3.1 Hz, 9H). **^{13}C NMR (126 MHz, Chloroform-*d*)** δ 152.2 (d, *J* = 27.1 Hz), 142.0 (d, *J* = 9.8 Hz), 135.5 (d, *J* = 1.8 Hz), 127.7 (d, *J* = 12.6 Hz), 127.3 (d, *J* = 2.8 Hz), 126.5, 125.9 (d, *J* = 1.7 Hz), 125.1 (d, *J* = 3.7 Hz), 120.4, 119.4, 117.0, 100.0, 37.4 (d, *J* = 34.6 Hz), 30.4 (d, *J* = 16.1 Hz), 4.0 (d, *J* = 15.1 Hz). **^{31}P NMR (162 MHz, $CDCl_3$)** δ 81.1.

ATR-FTIR (cm^{-1}): 2960, 1566, 1460, 1255, 1189, 688. HRMS m/z (ESI): calcd for $C_{23}H_{33}Cl_2NPSi$ $(M + H)^+$ 452.1491, found 452.1529.

C-H Germylation of Indole 1a

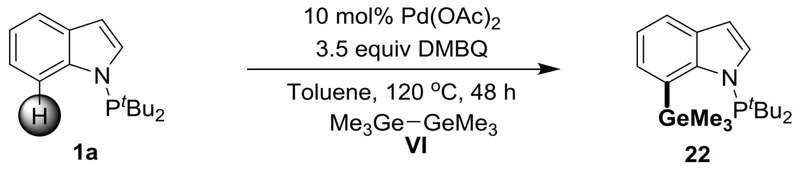

The reaction of **1a** (52.2 mg, 0.20 mmol), **VI** (235 mg, 1.0 mmol), $Pd(OAc)_2$ (4.48 mg, 10 mol%), DMBQ (95.2 mg, 0.7 mmol) in toluene (0.5 mL) at 120 ℃ under Ar. After 48 h, purification by column chromatography on silica gel (petroleum-ether) yield **22** (31.8 mg, 42%) as a colorless oil. **^{1}H NMR (500 MHz, Chloroform-*d*)** δ 7.61 (d, J = 7.7 Hz, 1H), 7.46 – 7.42 (m, 2H), 7.09 (t, J = 7.4 Hz, 1H), 6.67 (dd, J = 3.4, 1.3 Hz, 1H), 1.22 (d, J = 12.3 Hz, 18H), 0.62 (d, J = 2.0 Hz, 9H). **^{13}C NMR (126 MHz, Chloroform-*d*)** δ 147.3 (d, J = 20.8 Hz), 131.1 (d, J = 5.4 Hz), 130.2, 129.1 (d, J = 3.3 Hz), 124.7 (d, J = 9.4 Hz), 121.6, 119.7, 106.1 (d, J = 2.2 Hz), 36.1 (d, J = 28.3 Hz), 29.8 (d, J = 15.6 Hz), 3.7 (d, J = 13.9 Hz). **^{31}P NMR (162 MHz, $CDCl_3$)** δ 67.4. ATR-FTIR (cm^{-1}): 2965, 2798, 2589, 1545 1336, 1068. HRMS m/z (ESI): calcd for $C_{19}H_{33}GeNP^+$ 380.1557, found 380.1558.

Kinetic Studies

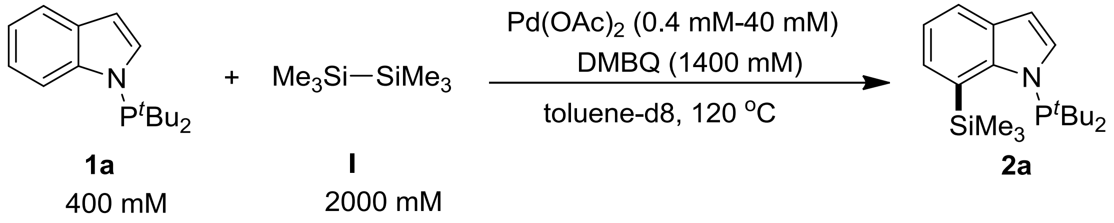

A 0.02 M stock solution of $Pd(OAc)_2$ was used (44.8 mg $Pd(OAc)_2$ dissolved in 10.0 mL toluene-d8) to dispense desired amounts of $Pd(OAc)_2$ to each Schlenk tube; **1a** (52.2 mg, 0.2 mmol), **I** (146 mg, 1.0 mmol), and DMBQ (95.2mg, 0.7 mmol) were dissolved in toluene-d8 (0.5 mL). The mixture was stirred at 120 ℃ under argon for desired time. The reaction mixture was analyzed by ^{31}P NMR analysis immediately (Triphenyl phosphite as the internal standard). For amounts of $Pd(OAc)_2$ used, see Supplementary Table 1.

Supplementary Table 1. Amounts of $Pd(OAc)_2$ and results used to determine the order in $Pd(OAc)_2$

Entry	$[Pd(OAc)_2]$ (mM)	Reaction time (min)	Yield (%)	Initial rate (mmol•L^{-1} •min^{-1})
1	0.4	1440	10.0	2.78×10^{-2}
2	4	480	8.8	7.33×10^{-2}
3	6	390	12.1	1.24×10^{-1}
4	8	300	10.1	1.34×10^{-1}
5	16	180	7.1	1.57×10^{-1}
6	24	180	8.7	1.93×10^{-1}
7	32	120	7.0	2.33×10^{-1}
8	40	120	8.0	2.67×10^{-1}

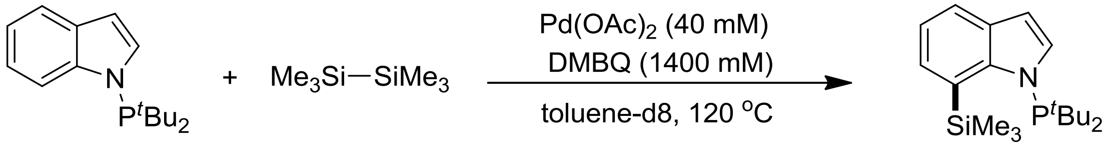

1a 240 mM-560 mM **I** 2000 mM **2a**

In an oven-dried Schlenk tube, **1a** (from 0.12 mmol to 0.28 mmol), **I** (146 mg, 1.0 mmol), $Pd(OAc)_2$ (4.48 mg, 0.02 mmol), DMBQ (95.2 mg, 0.7 mmol) were dissolved in toluene-d8 (0.5 mL). The mixture was stirred at 120 ℃ under argon for 90 min. The reaction mixture was analyzed by ^{31}P NMR analysis immediately (Triphenyl phosphite as the internal standard). For amounts of **1a** used, see Supplementary Table 2.

Supplementary Table 2. Amounts of 1a and results used to determine the order in 1a

Entry	[**1a**] (mM)	Reaction time (min)	Yield (%)	Initial rate (mmol•L^{-1} •min^{-1})
1	240	90	6.6	2.93×10^{-1}
2	320	90	6.0	2.67×10^{-1}
3	400	90	6.1	2.71×10^{-1}
4	480	90	6.0	2.67×10^{-1}
5	560	90	6.5	2.89×10^{-1}

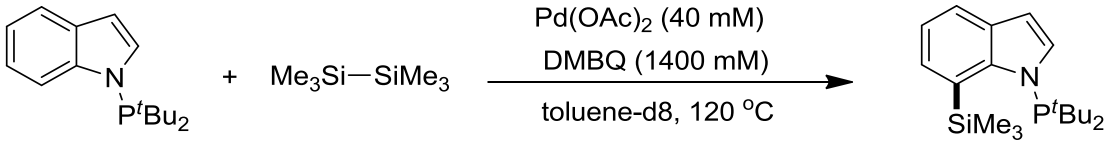

1a 400 mM **I** 400 mM-2000 mM **2a**

In an oven-dried Schlenk tube, **1a** (52.2 mg, 0.2 mmol), **I** (from 0.2 mmol to 1.0 mmol), $Pd(OAc)_2$ (4.48

mg, 0.02 mmol), DMBQ (95.2 mg, 0.7 mmol) were dissolved in toluene-d8 (0.5 mL). The mixture was stirred at 120 ℃ under argon for 210 min. The reaction mixture was analyzed by ^{31}P NMR analysis immediately (Triphenyl phosphite as the internal standard). For amounts of **I** used, see Supplementary Table 3.

Supplementary Table 3. Amounts of I and results used to determine the order in I

Entry	[**I**] (mM)	Reaction time (min)	Yield (%)	Initial rate (mmol•L^{-1} •min^{-1})
1	400	210	7.9	1.50×10^{-1}
2	800	210	9.0	1.71×10^{-1}
3	1200	210	10.7	2.04×10^{-1}
4	1600	210	12.8	2.44×10^{-1}
5	2000	210	15.0	2.86×10^{-1}

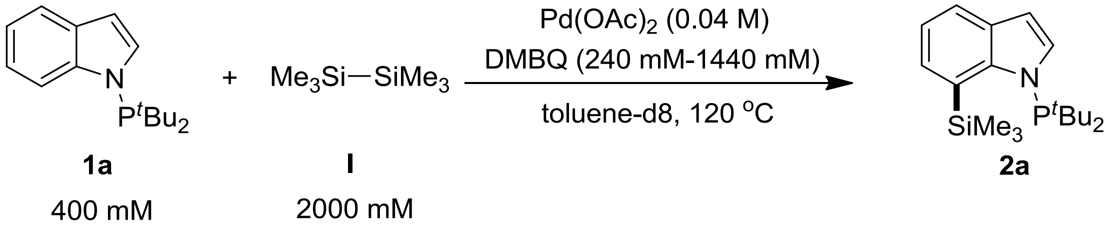

In an oven-dried Schlenk tube, **1a** (52.2 mg, 0.2 mmol), **I** (146 mg, 1.0 mmol), $Pd(OAc)_2$ (4.48 mg, 0.02 mmol), DMBQ (from 0.12 mmol to 0.72 mmol) were dissolved in toluene-d8 (0.5 mL). The mixture was stirred at 120 ℃ under argon for 120 min. The reaction mixture was analyzed by ^{31}P NMR analysis immediately (Triphenyl phosphite as the internal standard). For amounts of DMBQ used, see Supplementary Table 4.

Supplementary Table 4. Amounts of DMBQ and results used to determine the order in DMBQ

Entry	[**DMBQ**] (mM)	Reaction time (min)	Yield (%)	Initial rate (mmol•L^{-1} •min^{-1})
1	240	120	7.9	2.63×10^{-1}
2	480	120	7.1	2.36×10^{-1}
3	720	120	7.2	2.40×10^{-1}
4	960	120	7.0	2.33×10^{-1}
5	1200	120	7.8	2.6×10^{-1}
6	1440	120	7.6	2.53×10^{-1}

KIE Experiments

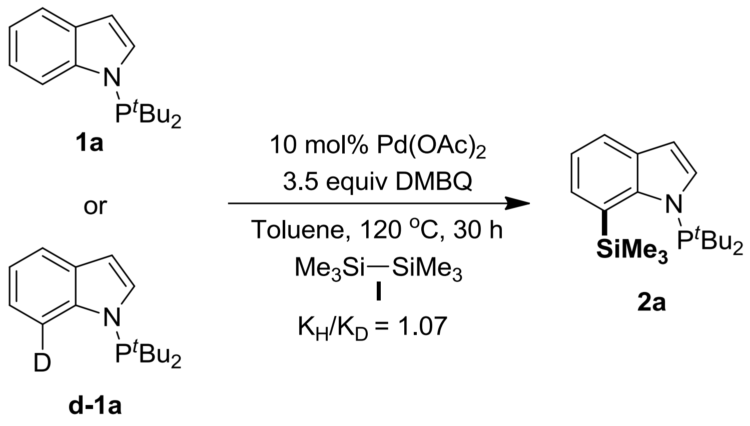

To a flame dried Schlenk tube was added indole substrates **1a** or **d-1a** (0.2 mmol), $Pd(OAc)_2$ (10 mol%) and DMBQ (3.5 equiv.) and toluene (0.5 mL) was added to the Schlenk tube under Ar atmosphere. Then **I** (5.0 equiv) was added to the reaction mixture and stirred at 120°C for the indicated time (five parallel runs). After cooled to room temperature it was passed through a short pad of silica and the filtrate was concentrated in vacuo. The yield was determined by ^{31}P NMR analysis of the crude product using triphenyl phosphite as the internal standard. For **1a**, y = 0.7667x+4.2, R^2 = 0.9888; for **d-1a**, y = 0.7167x+3.3, R^2 = 0.9898. KIE value (1.07) was determined by comparing the relative initial rates (Supplementary Figure 1).

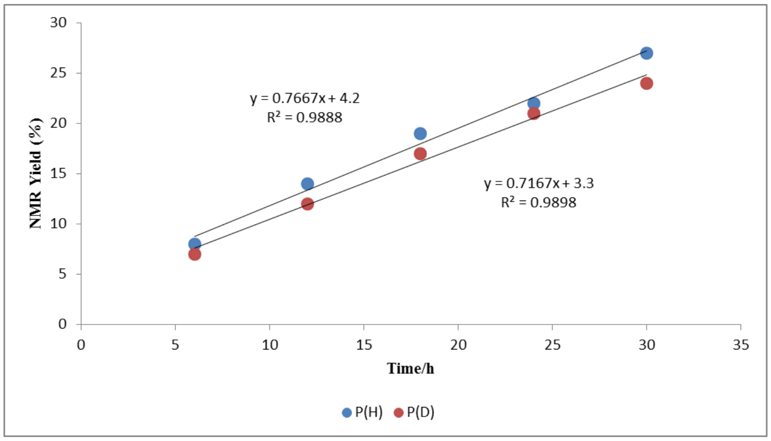

Supplementary Figure 1. KIE experiments of **1a** and **d-1a**.

Synthesis of Cyclopalladated Complex 23

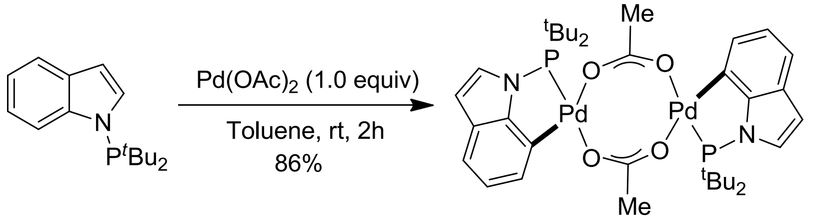

1a **23**

$Pd(OAc)_2$ (44.8 mg, 0.2 mol, 1.0 equiv) and **1a** (52.2 mg, 0.2 mol, 1.0 equiv.) were placed in a Schlenk tube under a nitrogen atmosphere followed by the addition of toluene (1 mL). The reaction mixture was stirred at room temperature for 2 h. Then the solvent was removed. The solid was washed with hexane and dried under vacuum to give **23** as a yellow-green solid (73.1 mg, 86%). The single crystals of **23** was grown by slow evaporation of the DCM (Dichloromethane)/ Hexane.

Computational Details and Discussion

Computational methods

All DFT calculations were performed with the Gaussian 09 package.[1] Geometry optimizations were performed in the gas phase B3LYP[2-4] functional with D3 version of Grimme's dispersion[5], and the 6-31G(d)[6-8] basis set for all nonmetal atoms and the LANL2DZ[9-10] basis set with effective core potential (ECP) for Pd. Frequency analysis was conducted at the same level of theory to verify the stationary points to be minima or saddle points and to obtain zero-point vibrational energy (ZPVE) and thermal energy corrections under 298.15 K and 1 atm pressure. All transition states were confirmed to connect reactants and products by intrinsic reaction coordinate (IRC) calculations. Single-point solvation energies were calculated with M06[11] functional with the 6-311++G(d,p)[12] for all nonmetal atoms and the SDD[13-14] for Pd by using SMD[15-16] solvation model (solvent = toluene). Grimme[17] correction (frequency cut-off is 100 cm^{-1}) for entropy and Head-Gordon[18] correction for enthalpy are performed under 120℃ using GoodVibes v3.0.1[19]. Unless otherwise noted, the relative energies reported in the text are Gibbs free energies with the solvent effect corrections. Computed structures are illustrated using CYLView (Supplementary Figure 2).[20]

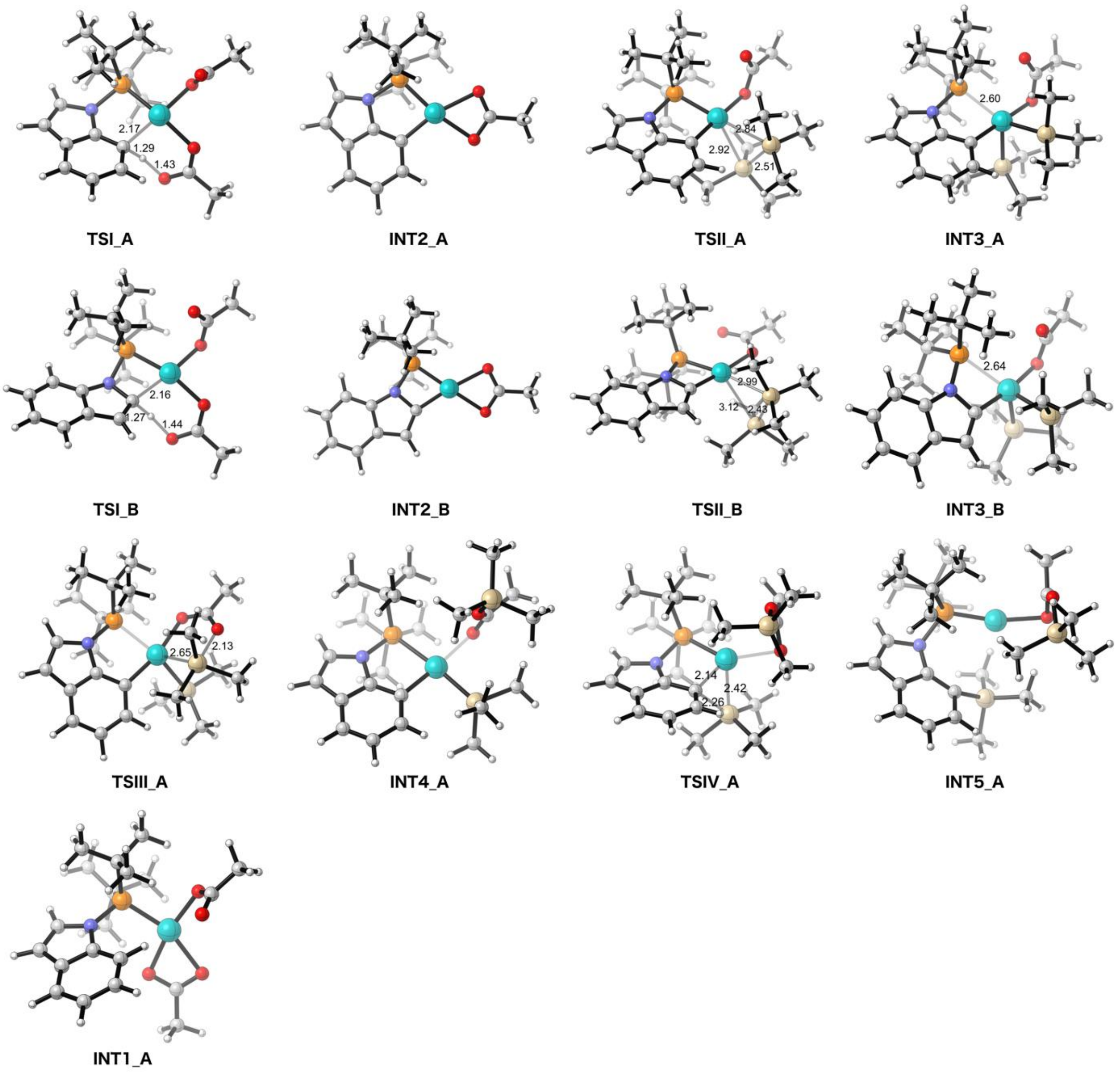

Supplementary Figure 2. Optimized **TS** and key intermediates structures.

Based on the DFT calculations shown in Supplementary Figure 3, the P^tBu_2 is the best directing group to active the C7 position with a stable five member formed.

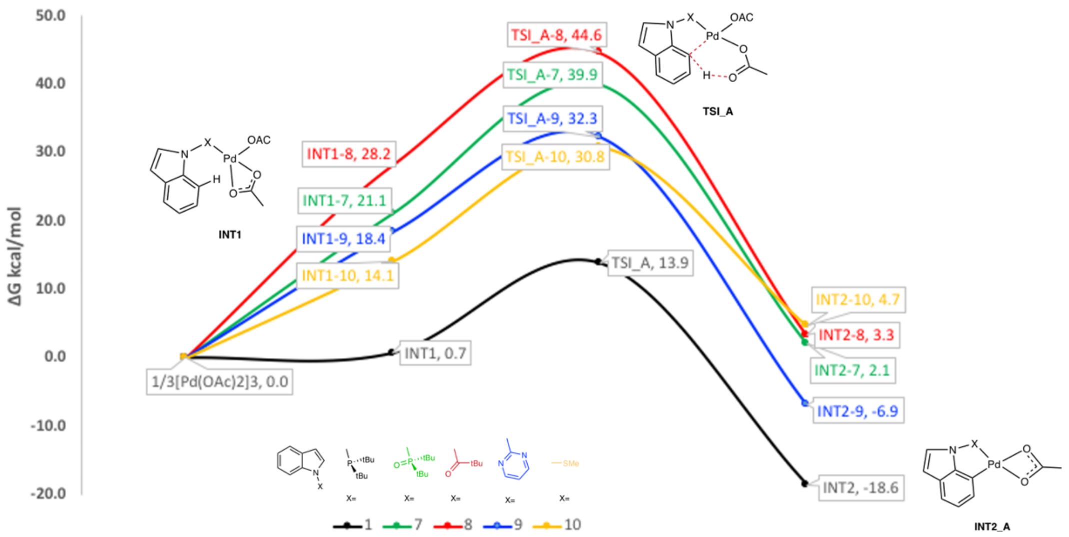

Supplementary Figure 3. C-H activation step using 7, 8, 9 and 10.

Based on the DFT calculations shown in Supplementary Figure 4, the DMBQ as external base for C-H activation is feasible with a lower free energy barrier of 26.1 kcal/mol.

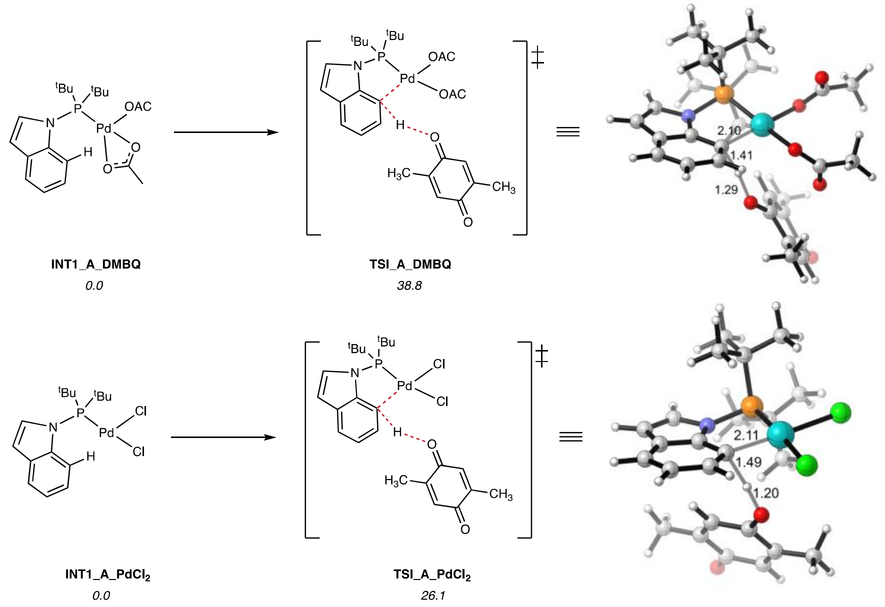

Supplementary Figure 4. C-H activation step using DMBQ.

Supplementary Table 5. Energies, and vibrational frequencies

Structure	E_SPC	E	ZPE	H_SPC	qh-H_SPC	G(T)_SPC	qh-G(T)_SPC
**1a**	-1019.925691	-1020.317485	0.368359	-1019.523991	-1019.526670	-1019.625426	-1019.624743
**2a**	-1428.519904	-1428.998402	0.471335	-1428.003091	-1428.007248	-1428.129762	-1428.129309
**Cat_3Pd**	-1754.454286	-1751.367846	0.316124	-1754.083314	-1754.093807	-1754.247296	-1754.242382
**DMBQ**	-459.920599	-460.110113	0.141296	-459.762554	-459.763567	-459.828415	-459.828621
**24**	-869.784327	-870.060151	0.266587	-869.487373	-869.490662	-869.587455	-869.586066
**HOAC**	-229.037044	-229.084787	0.062032	-228.966807	-228.967307	-229.012998	-229.012919
**INT1_A**	-1604.752441	-1604.100753	0.474435	-1604.225565	-1604.232686	-1604.373373	-1604.371080
**INT2_A**	-1375.717479	-1374.997929	0.410636	-1375.263364	-1375.268570	-1375.391521	-1375.388904
**INT2_B**	-1375.700918	-1374.979873	0.410358	-1375.246983	-1375.252254	-1375.375145	-1375.372898
**INT2_dimmer_A**	-2751.481300	-2750.070608	0.824602	-2750.569151	-2750.580852	-2750.789907	-2750.785316
**INT3_A**	-2194.110951	-2193.567506	0.639401	-2193.402557	-2193.410328	-2193.579865	-2193.579229
**INT3_B**	-2194.104262	-2193.559583	0.638035	-2193.396541	-2193.405080	-2193.577515	-2193.576119
**INT4_A**	-2194.141043	-2193.589556	0.637281	-2193.433900	-2193.442811	-2193.617201	-2193.614830
**INT5_A**	-2194.142473	-2193.592389	0.637875	-2193.435039	-2193.444013	-2193.617870	-2193.615677
**Si_2Me_6**	-818.382112	-818.553451	0.223607	-818.132380	-818.135001	-818.221313	-818.220804
**TSI_A**	-1604.728622	-1604.069840	0.469521	-1604.207683	-1604.214336	-1604.352055	-1604.350122
**TSI_B**	-1604.715531	-1604.058421	0.468522	-1604.195050	-1604.202206	-1604.342314	-1604.339766
**TSII_A**	-2194.093418	-2193.543064	0.638068	-2193.386716	-2193.394848	-2193.564804	-2193.563557
**TSII_B**	-2194.081802	-2193.531692	0.636834	-2193.375663	-2193.384738	-2193.558093	-2193.555607
**TSIII_A**	-2194.097195	-2193.549613	0.639135	-2193.390251	-2193.398021	-2193.565585	-2193.564180
**TSIV_A**	-2194.128848	-2193.574745	0.636951	-2193.423191	-2193.431618	-2193.602379	-2193.600747

E_SPC= Single-point solvation energies; E= Electronic energies; ZPE = zero-point vibrational energy; H_SPC = enthalpy; qh-H_SPC = enthalpy with Head-Gordon correction; G(T)_SPC = Gibbs free energy; qh-G(T)_SPC = Gibbs free energy with Head-Gordon correction.

.

Supplementary Figures

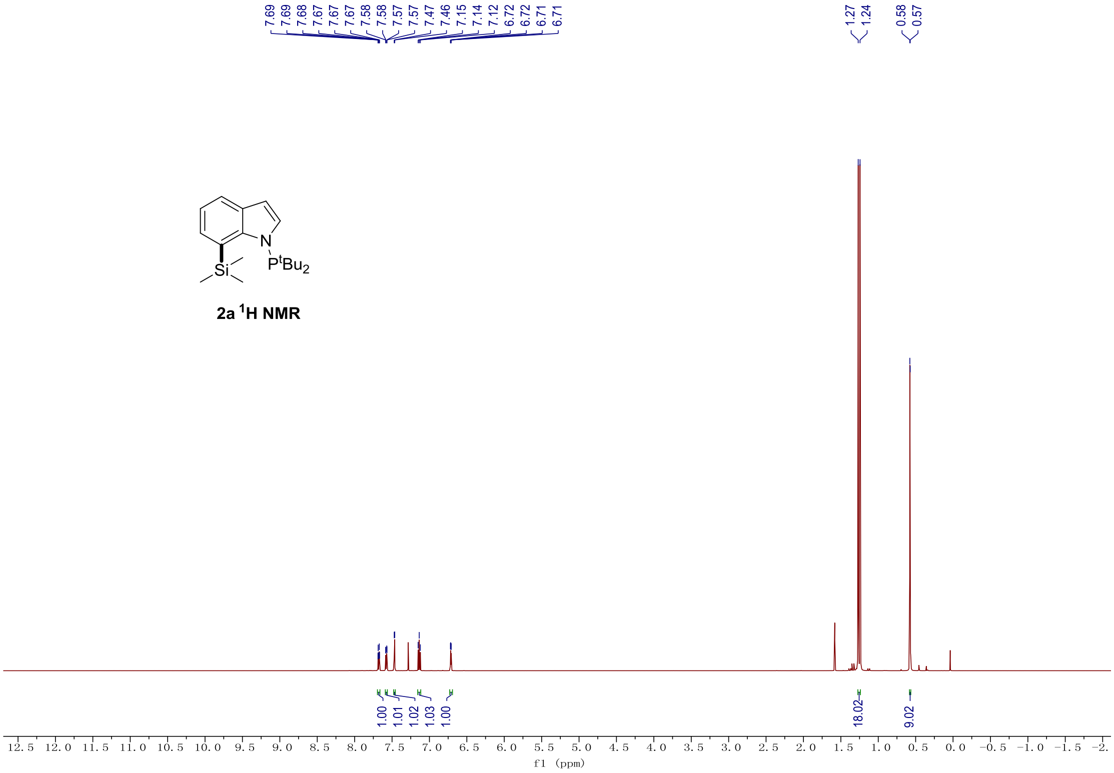

Supplementary Figure 5. ^{1}H NMR of compound 2a.

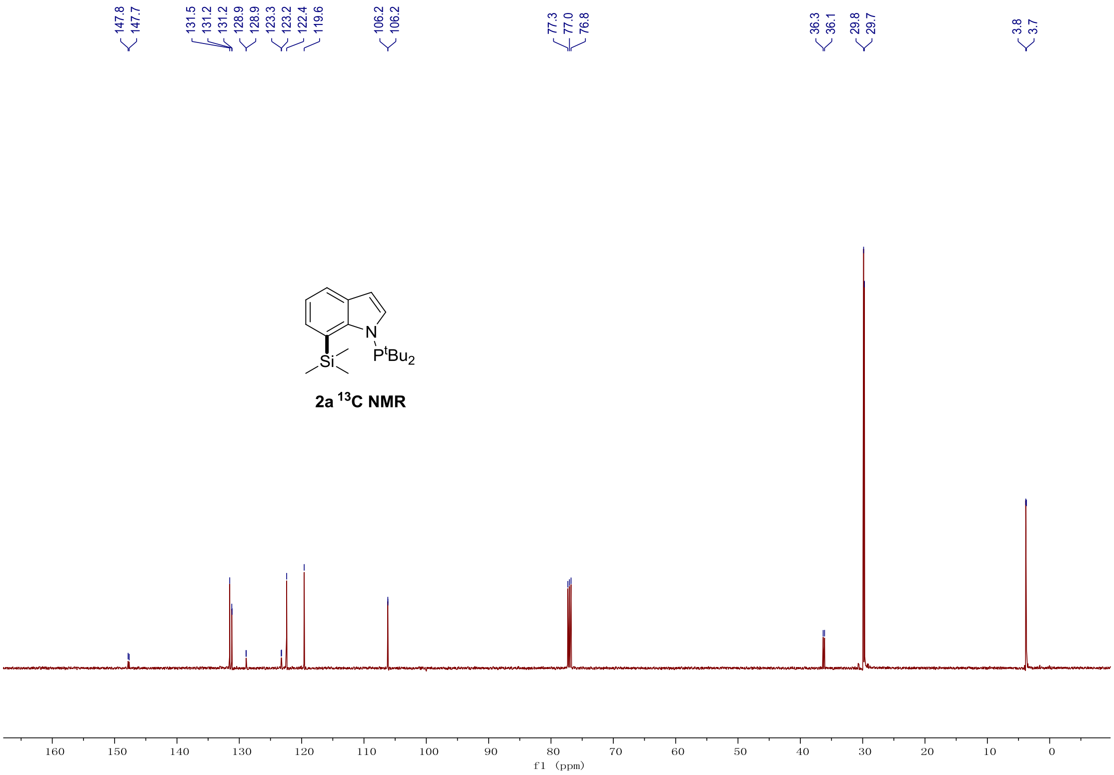

Supplementary Figure 6. ^{13}C NMR of compound 2a.

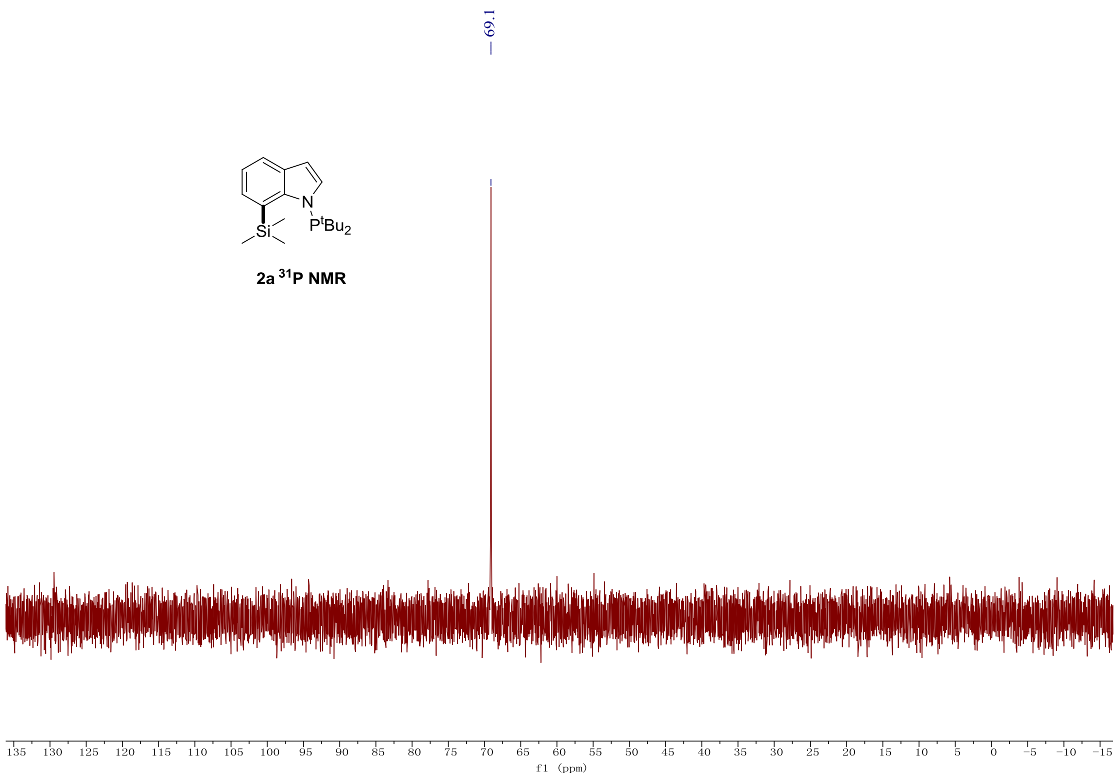

Supplementary Figure 7. ^{31}P NMR of compound 2a.

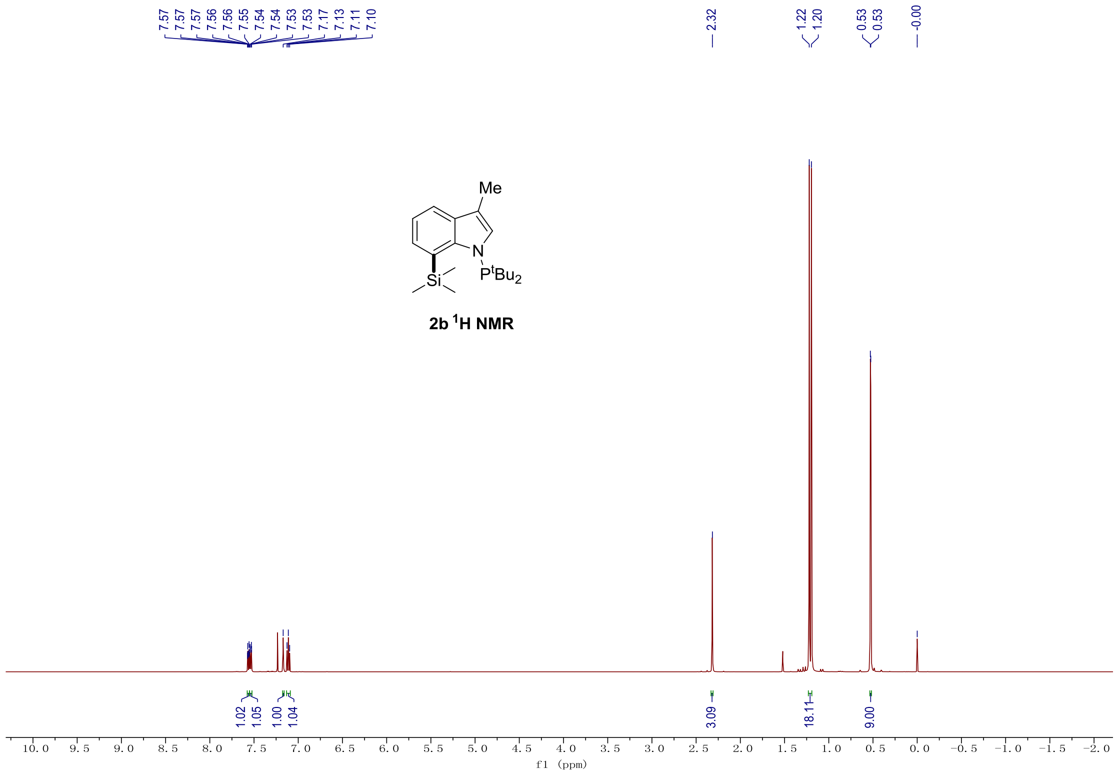

Supplementary Figure 8. ^{1}H NMR of compound 2b.

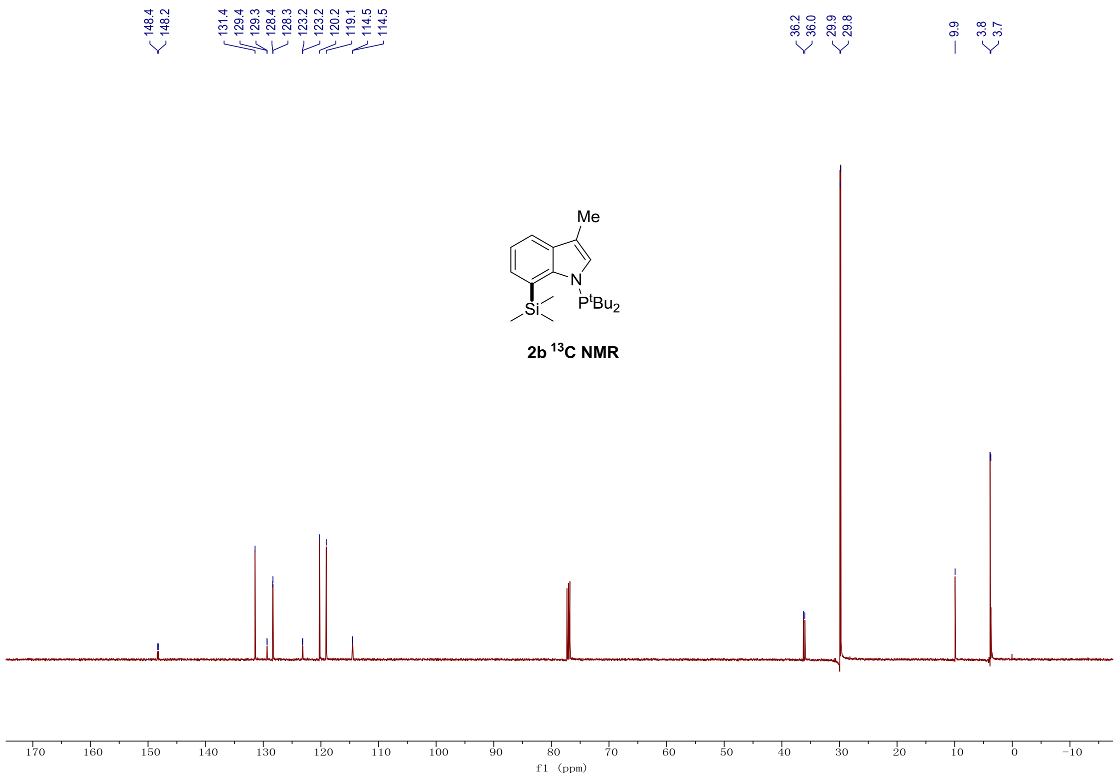

Supplementary Figure 9. ^{13}C NMR of compound 2b.

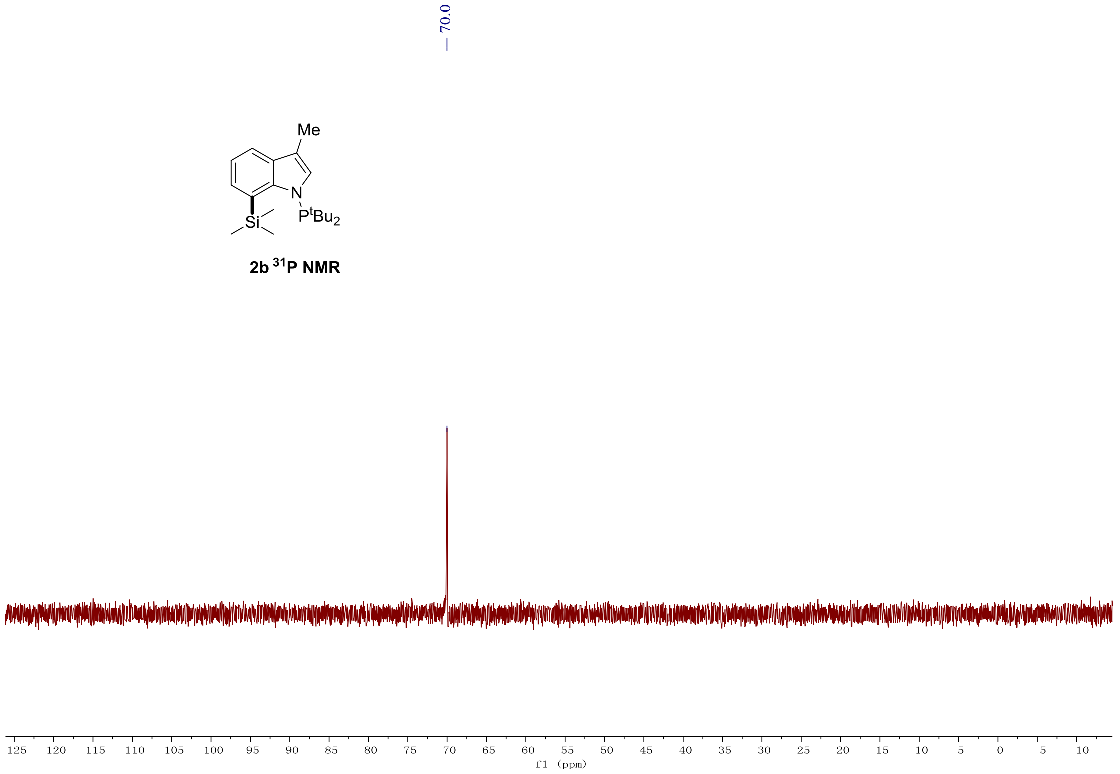

Supplementary Figure 10. ^{31}P NMR of compound 2b.

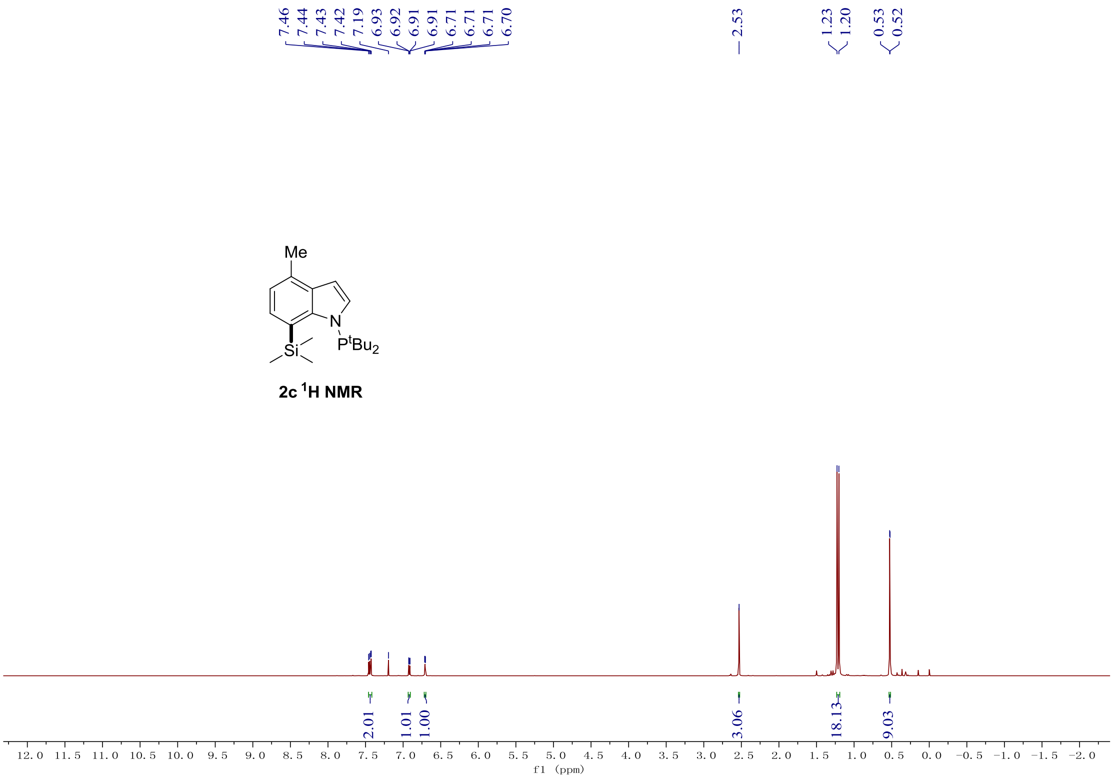

Supplementary Figure 11. ^{1}H NMR of compound 2c.

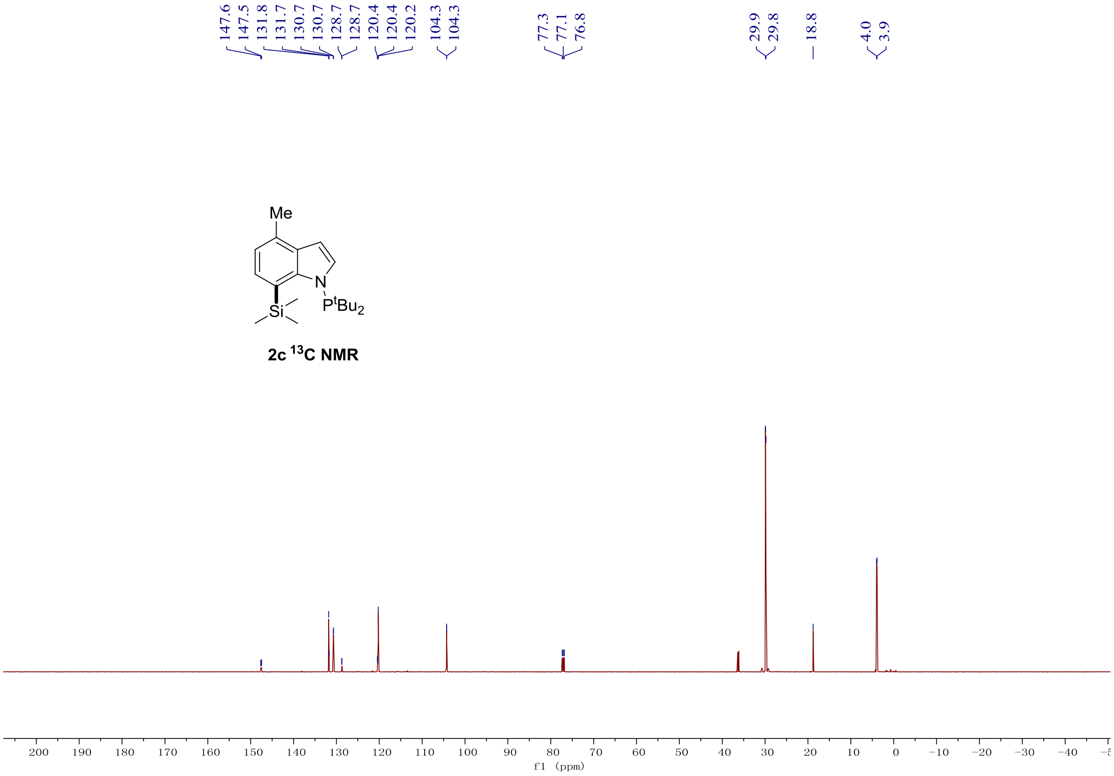

Supplementary Figure 12. ^{13}C NMR of compound 2c.

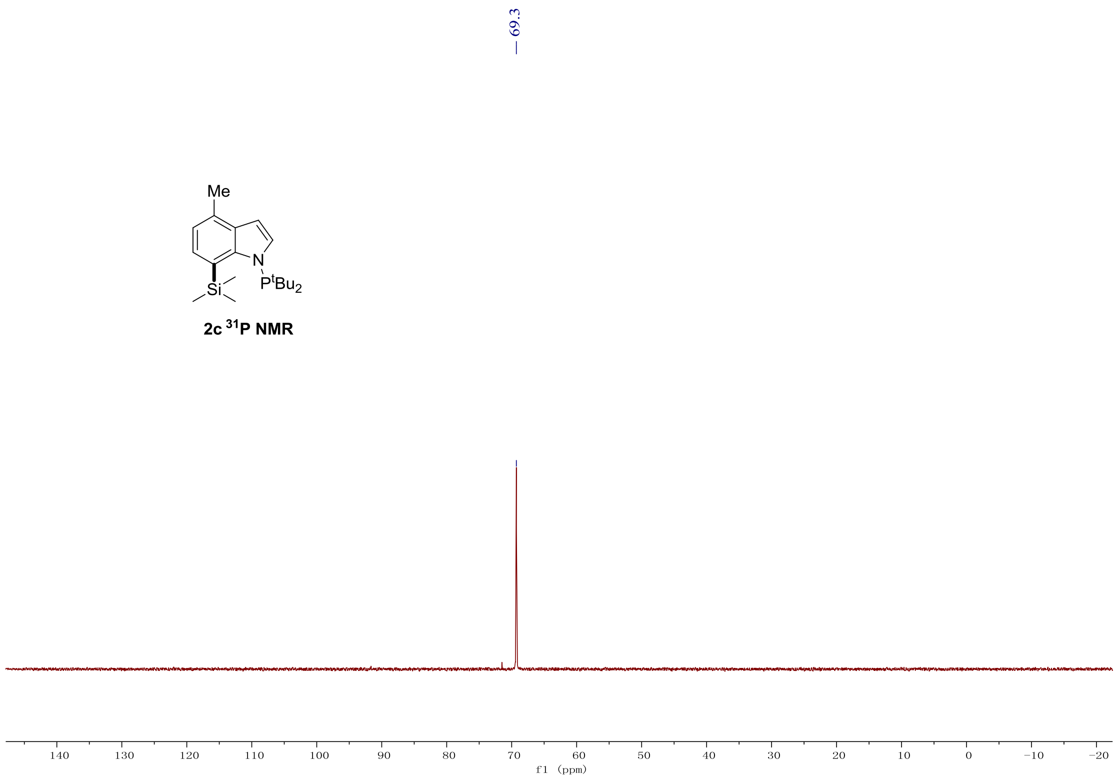

Supplementary Figure 13. ^{31}P NMR of compound 2c.

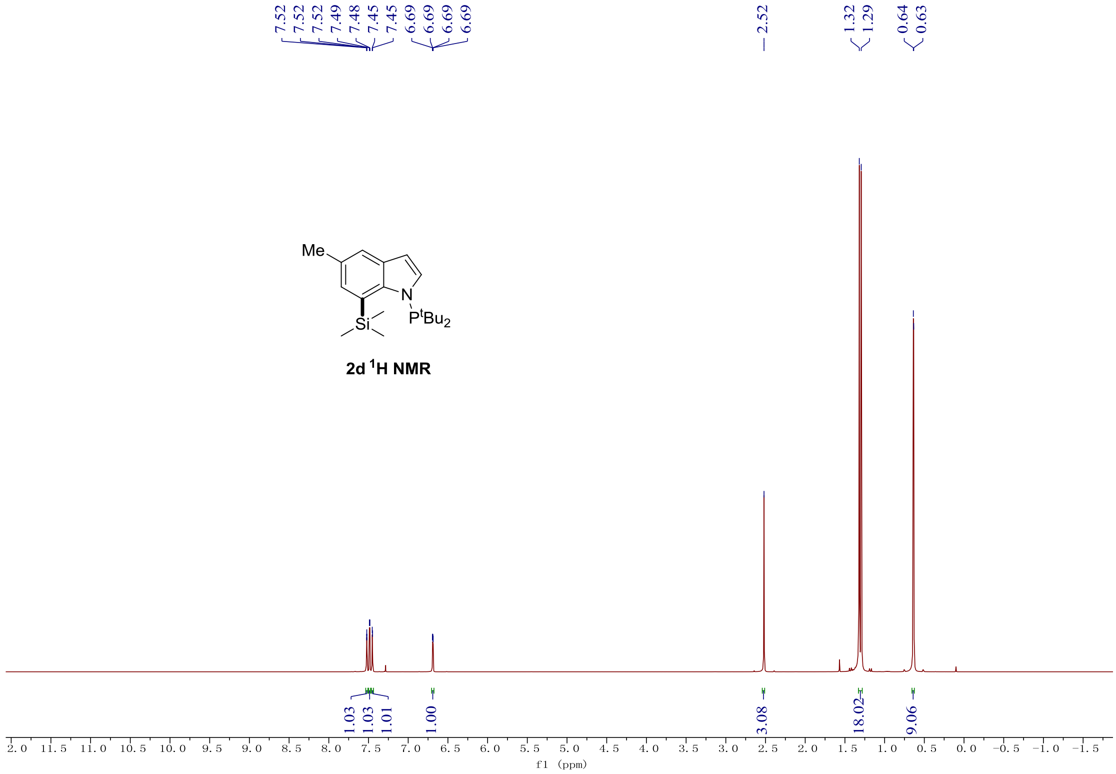

Supplementary Figure 14. ^{1}H NMR of compound 2d.

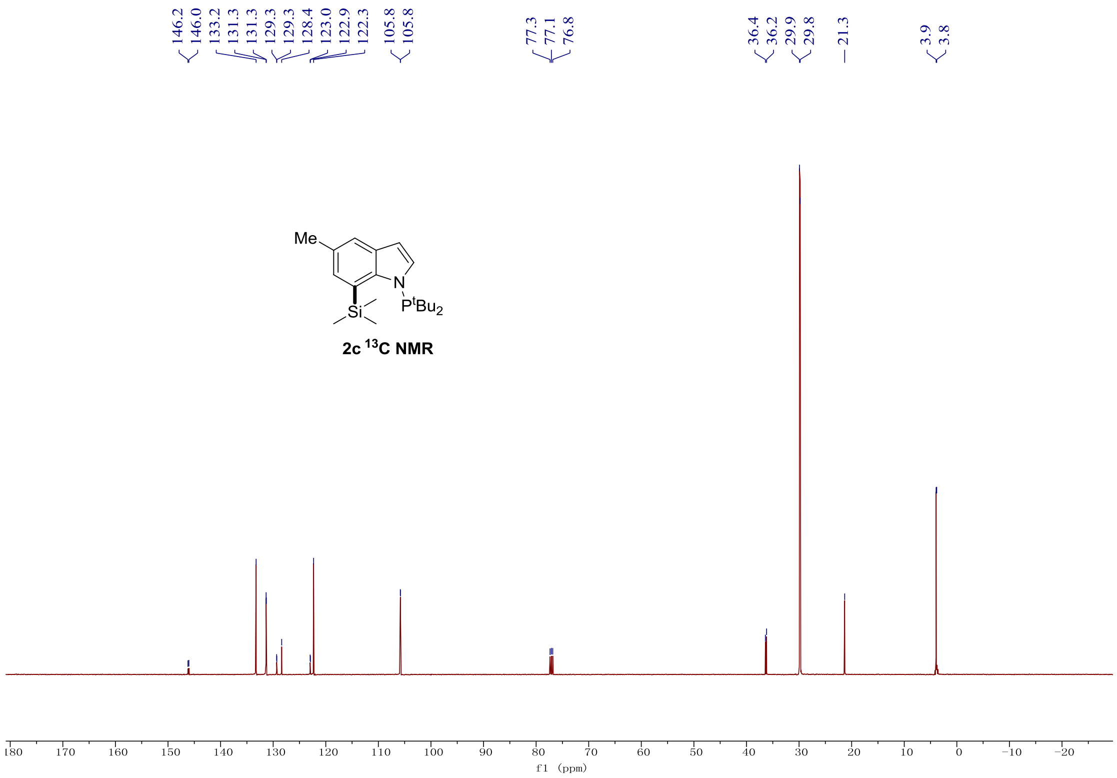

Supplementary Figure 15. ^{13}C NMR of compound 2d.

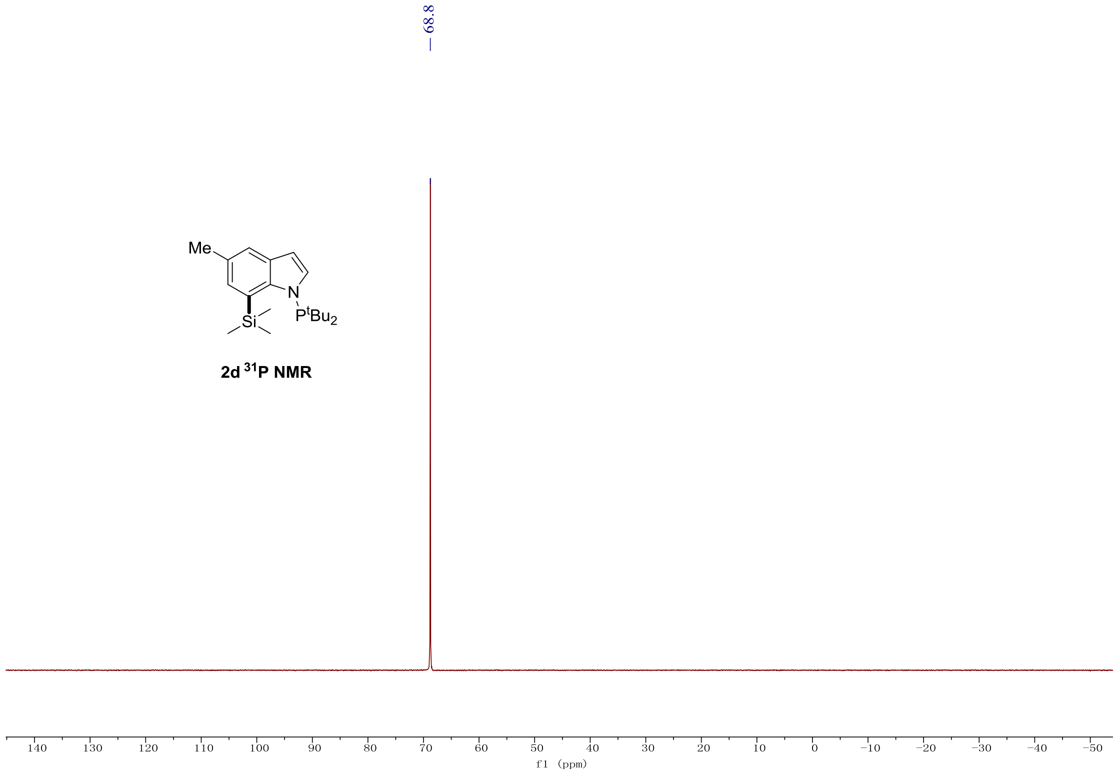

Supplementary Figure 16. ^{31}P NMR of compound 2d.

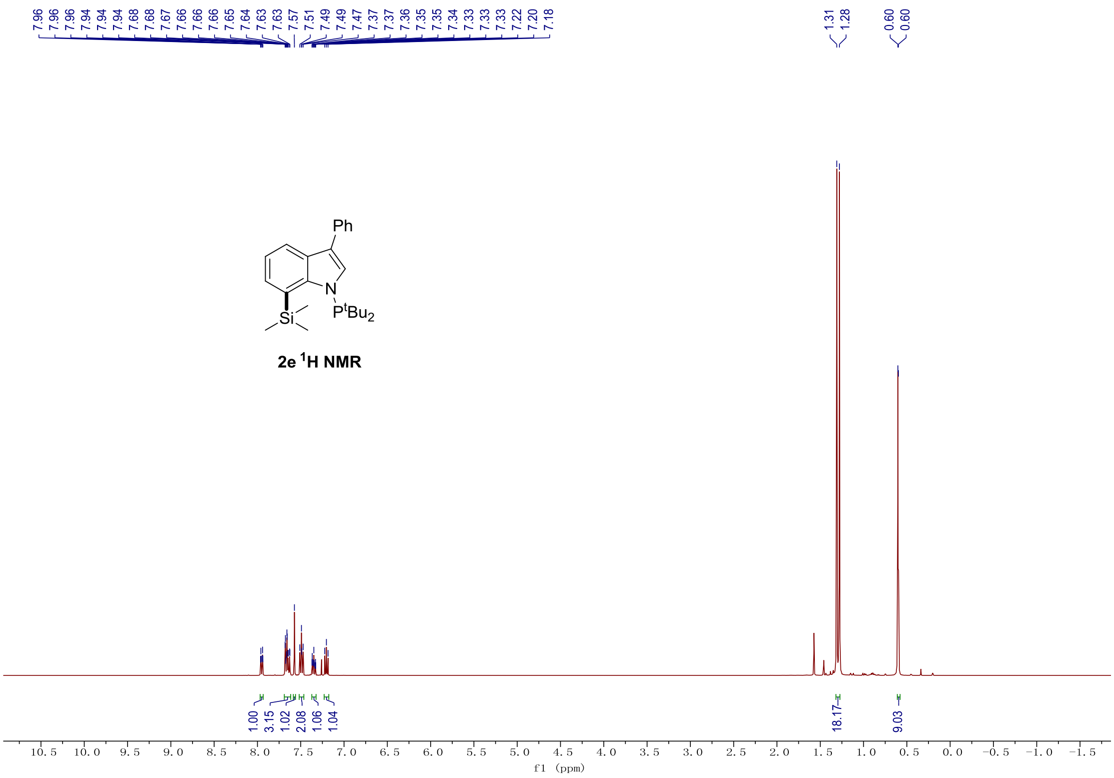

Supplementary Figure 17. ^{1}H NMR of compound 2e.

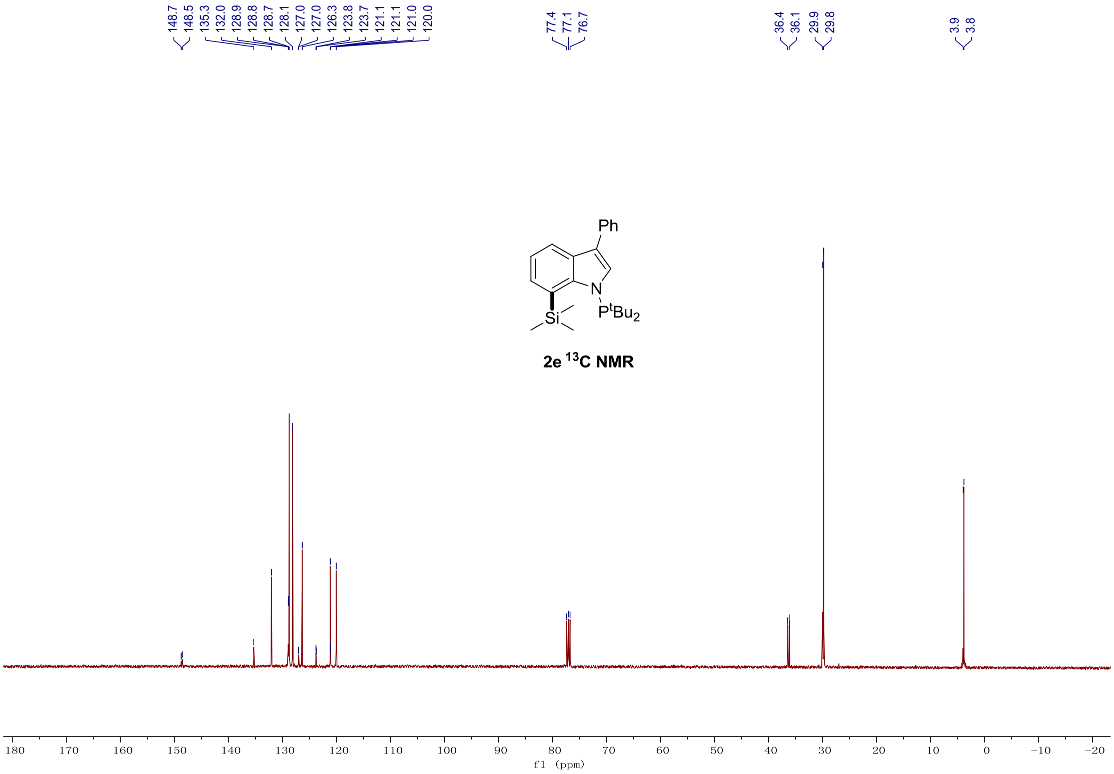

Supplementary Figure 18. ^{13}C NMR of compound 2e.

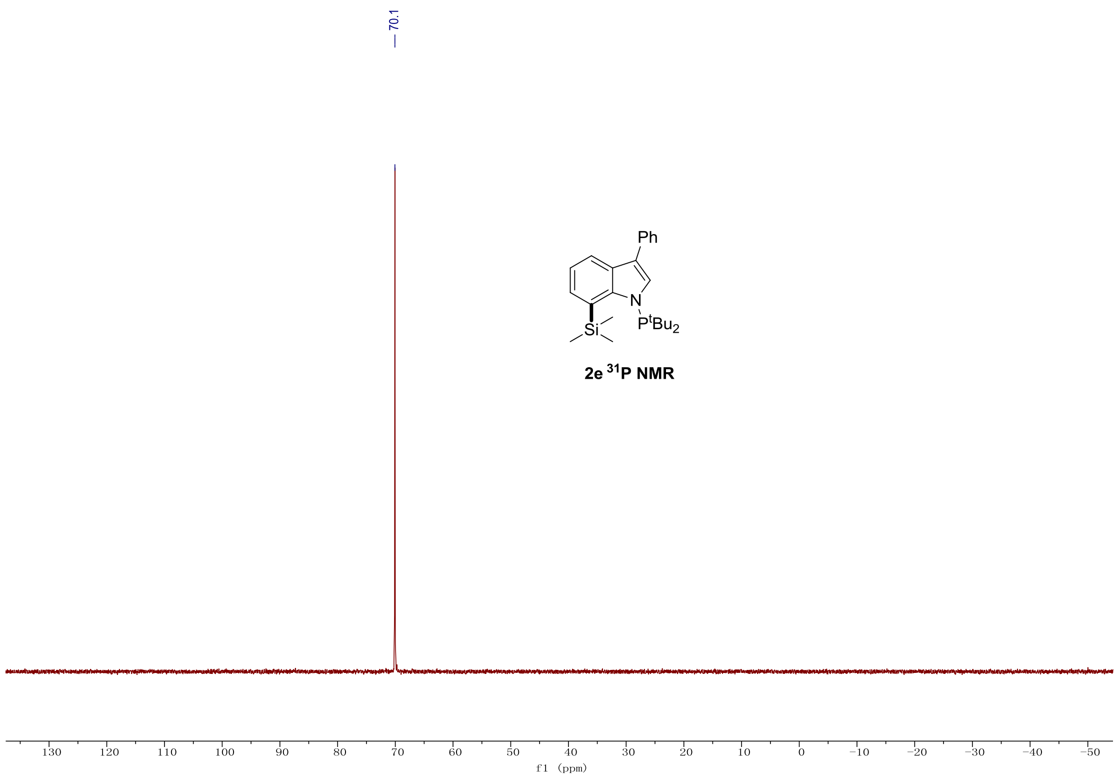

Supplementary Figure 19. ^{31}P NMR of compound 2e.

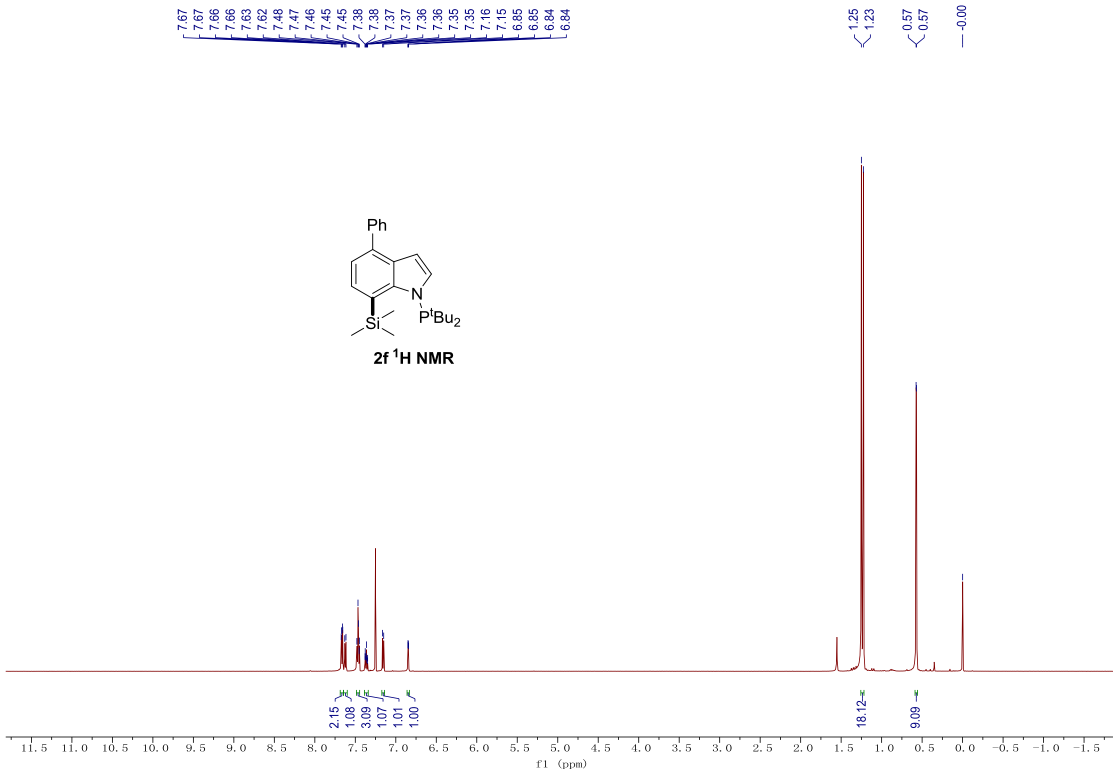

Supplementary Figure 20. ^{1}H NMR of compound 2f.

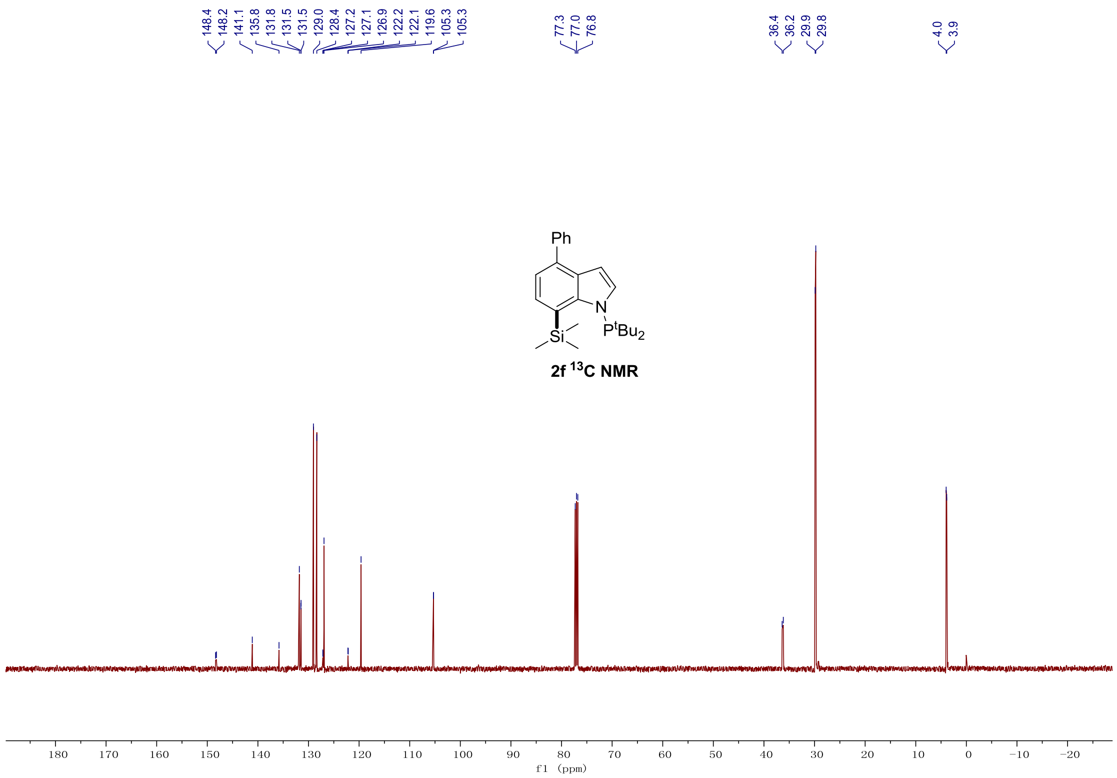

Supplementary Figure 21. ^{13}C NMR of compound 2f.

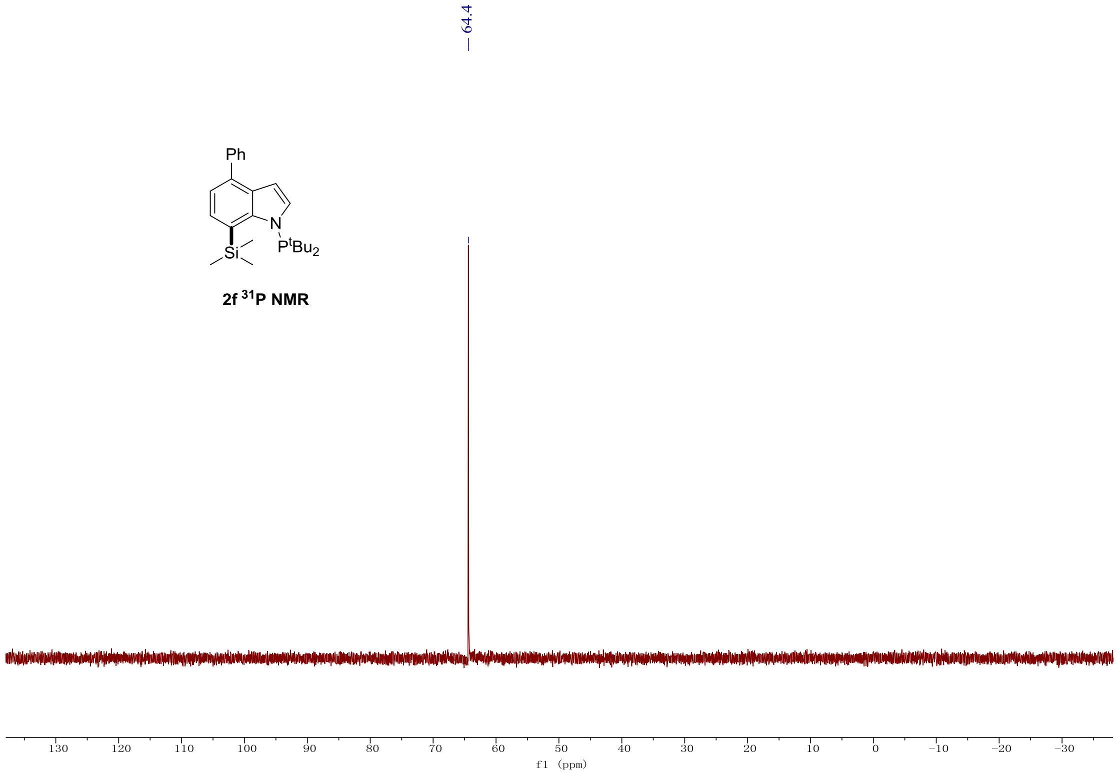

Supplementary Figure 22. ^{31}P NMR of compound 2f.

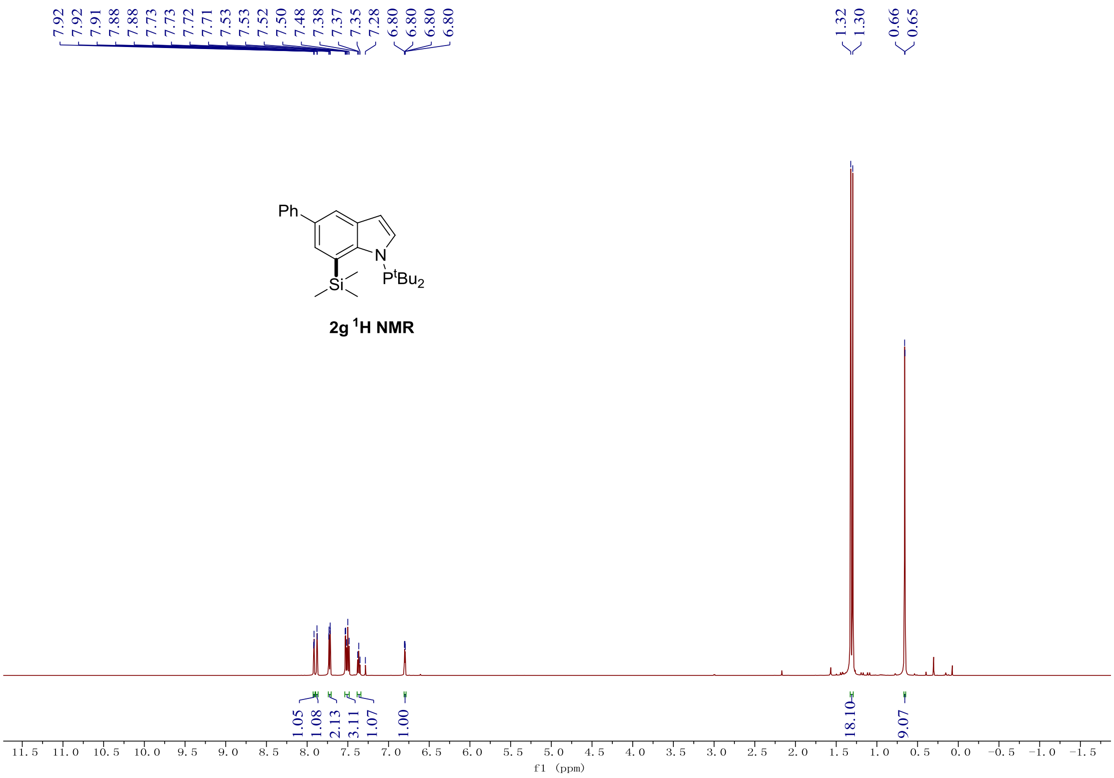

Supplementary Figure 23. ^{1}H NMR of compound 2g.

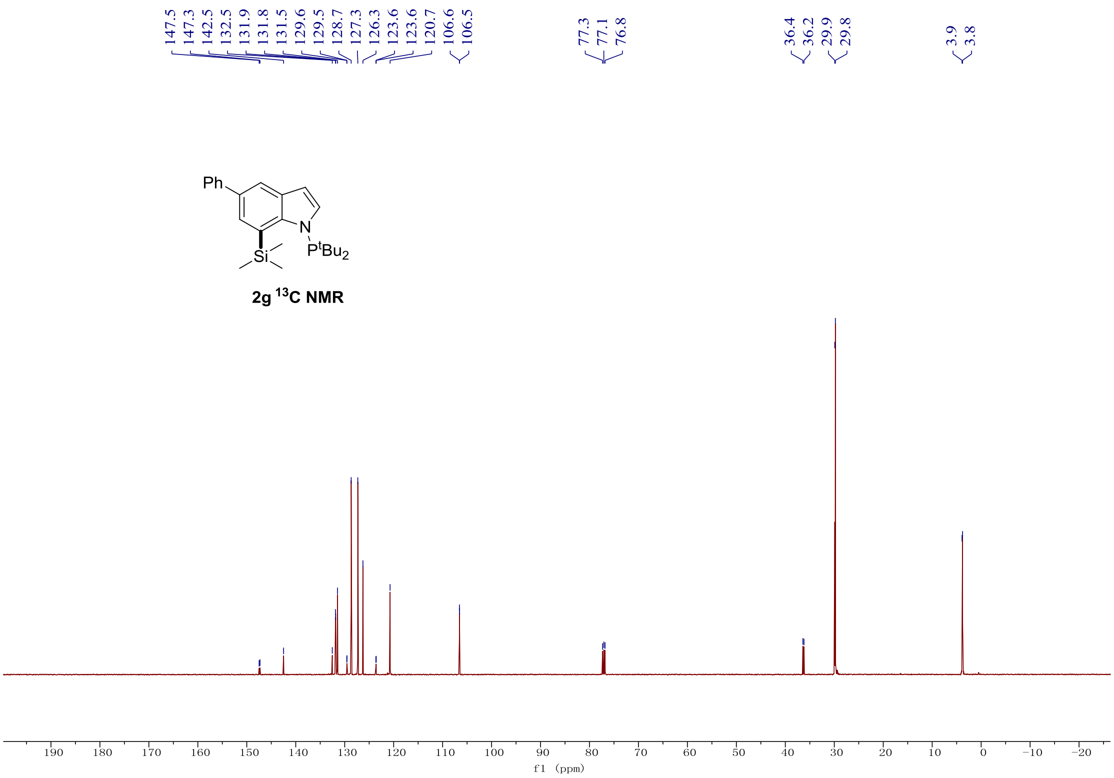

Supplementary Figure 24. ^{13}C NMR of compound 2g.

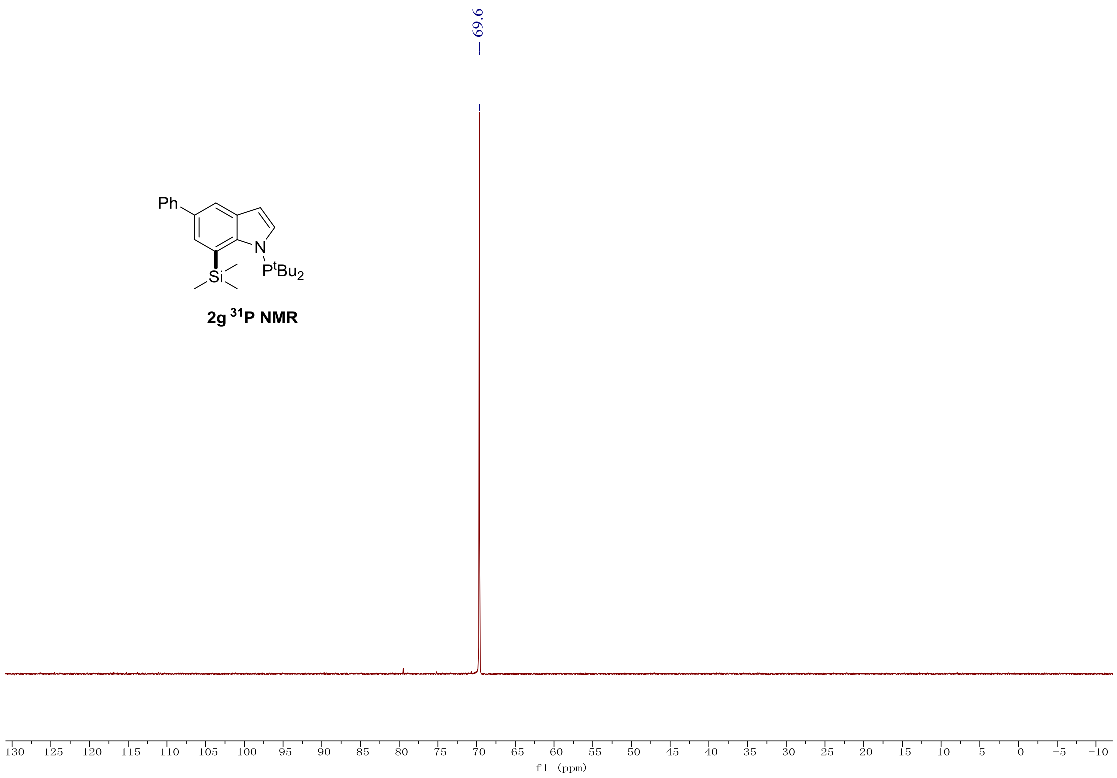

Supplementary Figure 25. ^{31}P NMR of compound 2g.

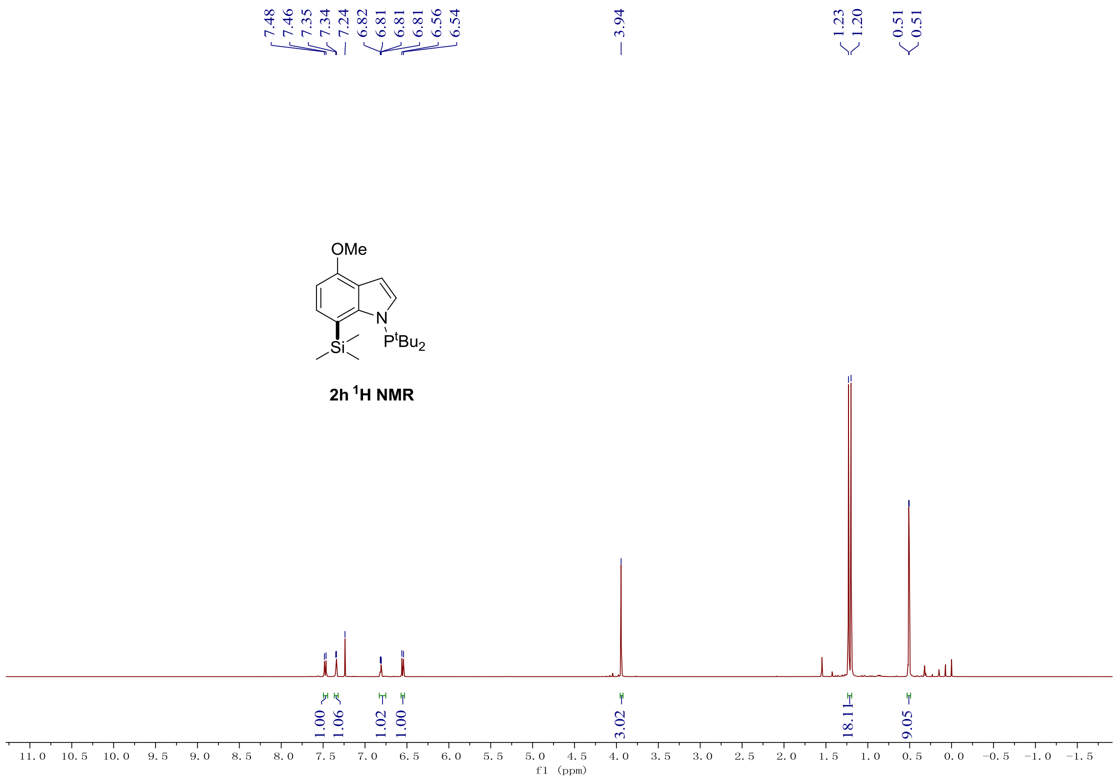

Supplementary Figure 26. ^{1}H NMR of compound 2h.

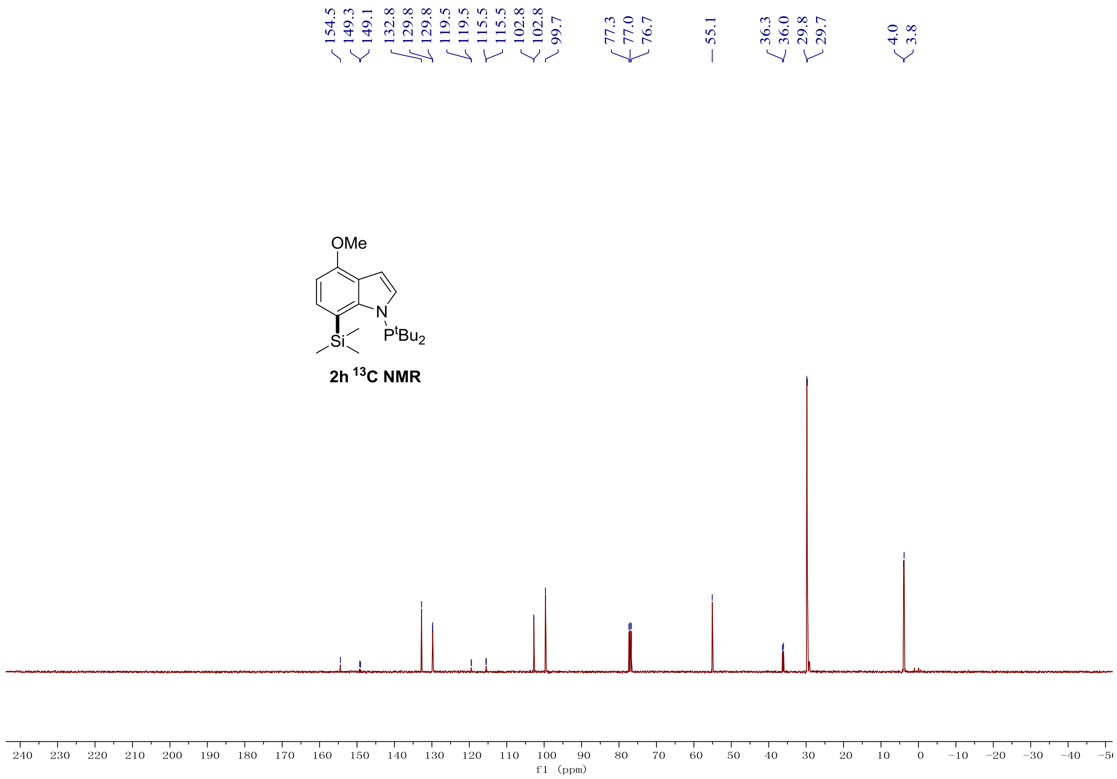

Supplementary Figure 27. ^{13}C NMR of compound 2h.

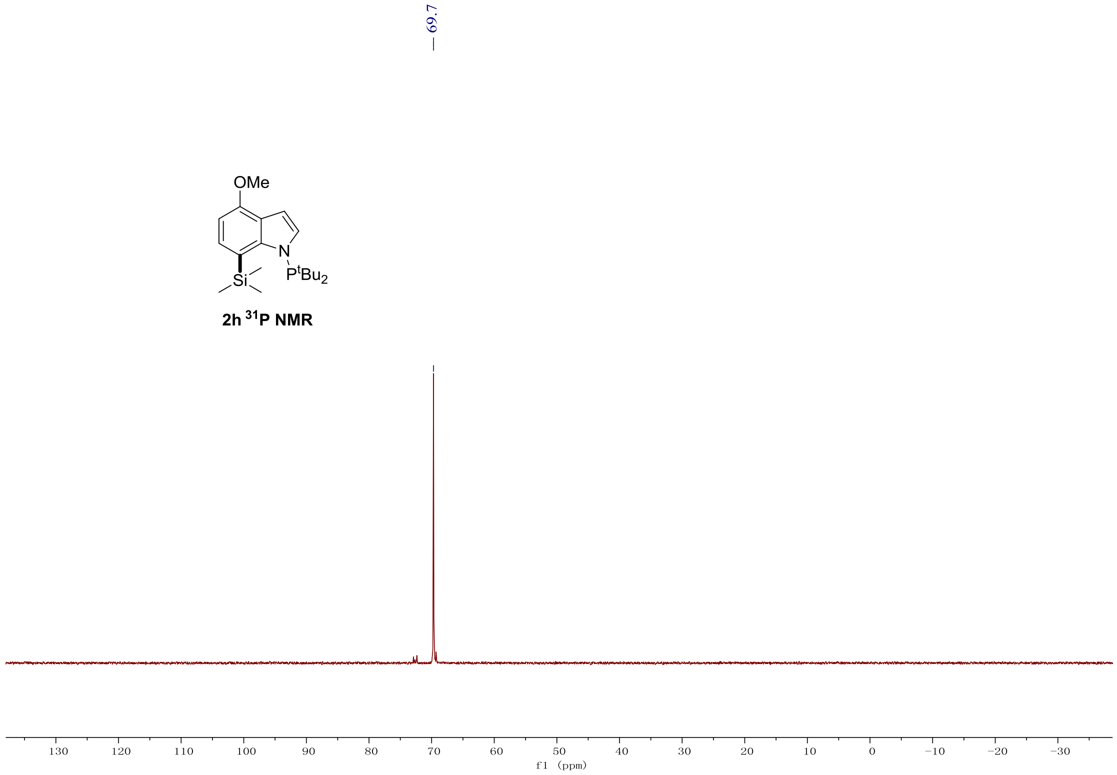

Supplementary Figure 28. ^{31}P NMR of compound 2h.

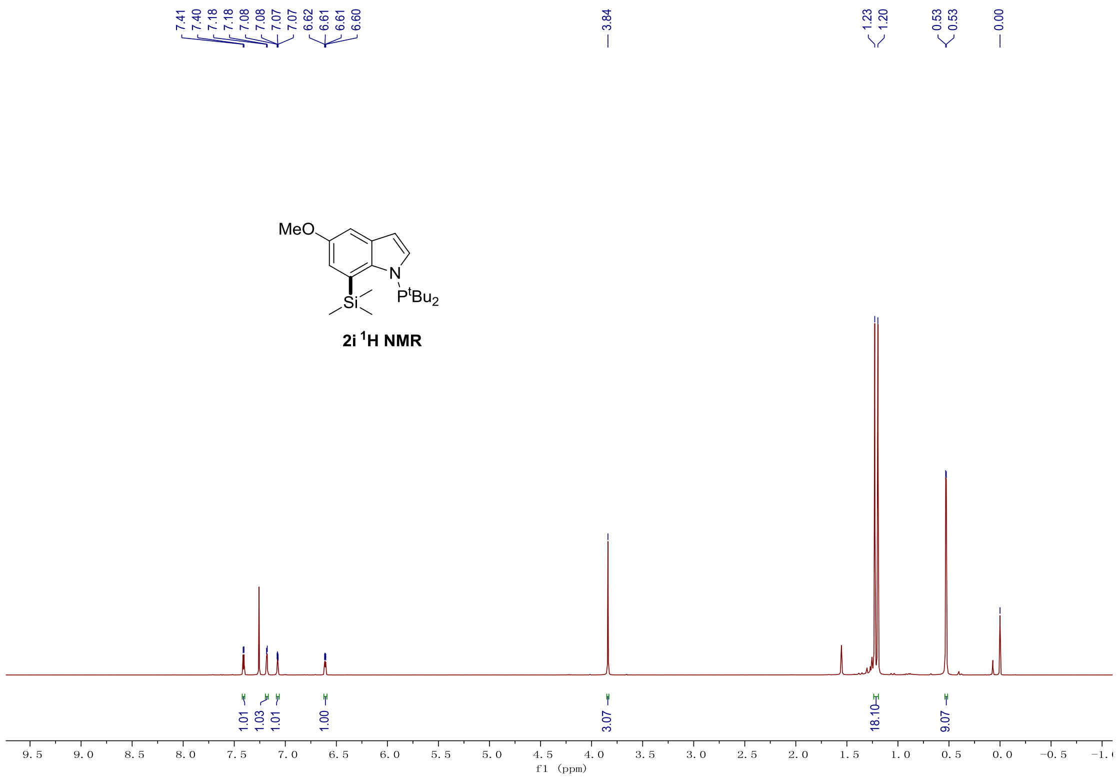

Supplementary Figure 29. ^{1}H NMR of compound 2i.

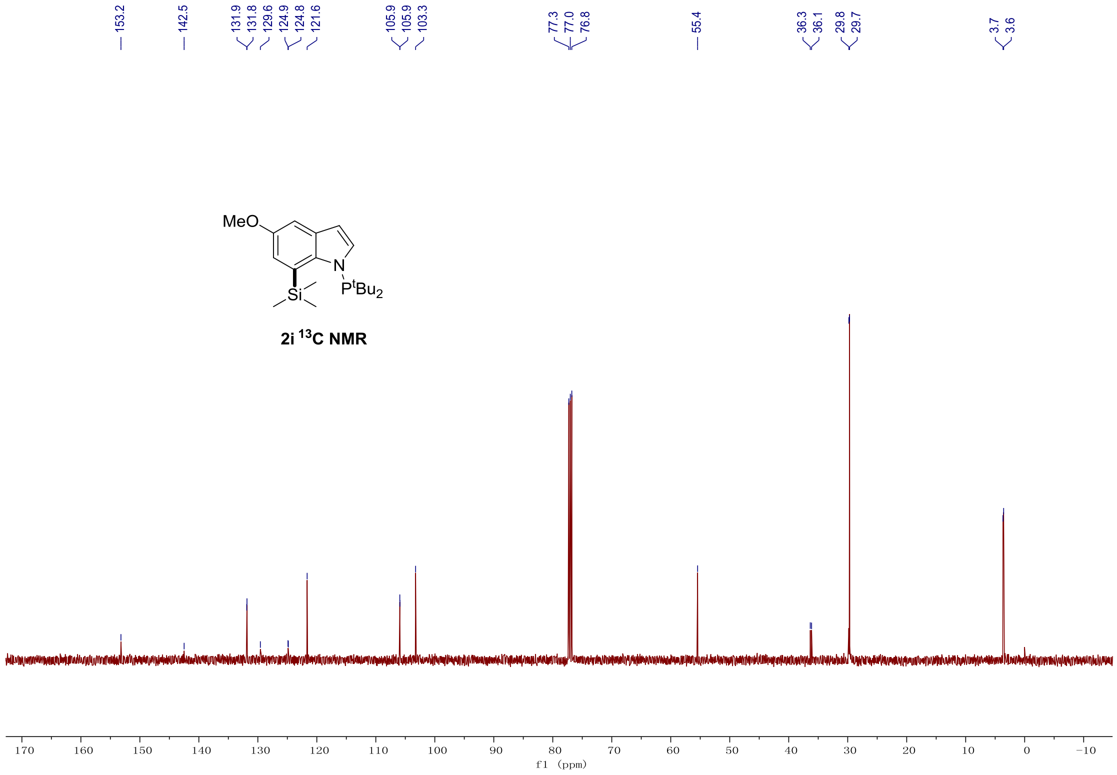

Supplementary Figure 30. ^{13}C NMR of compound 2i.

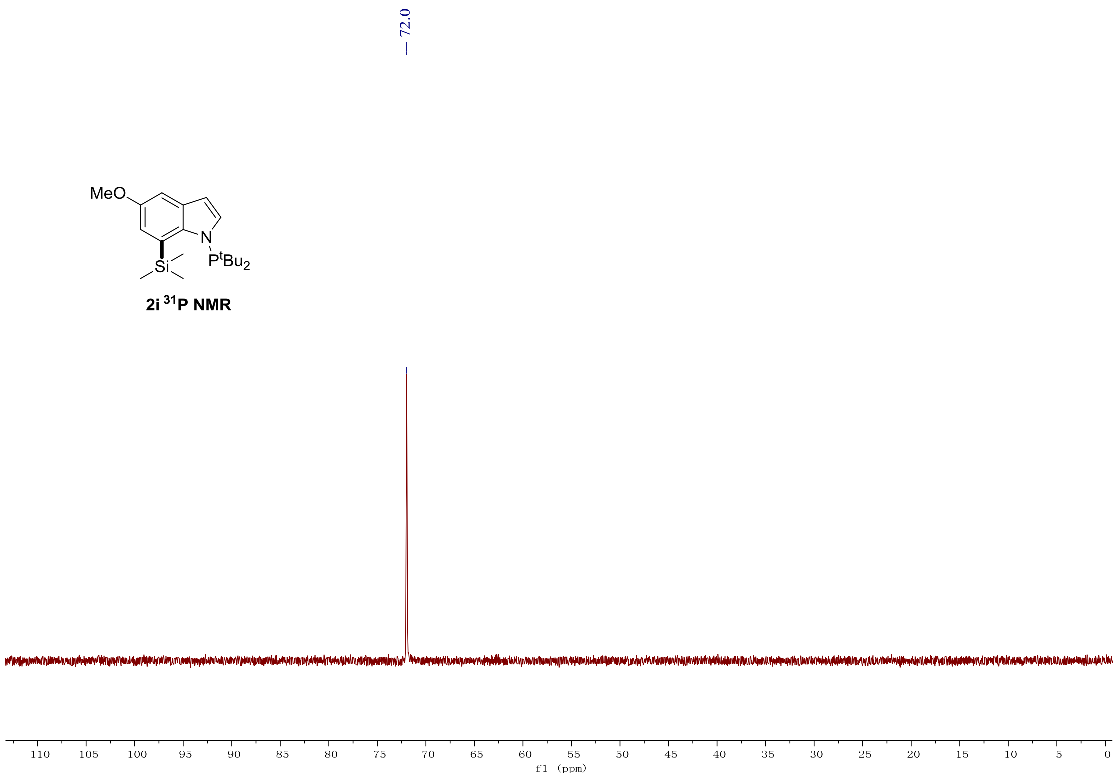

Supplementary Figure 31. ^{31}P NMR of compound 2i.

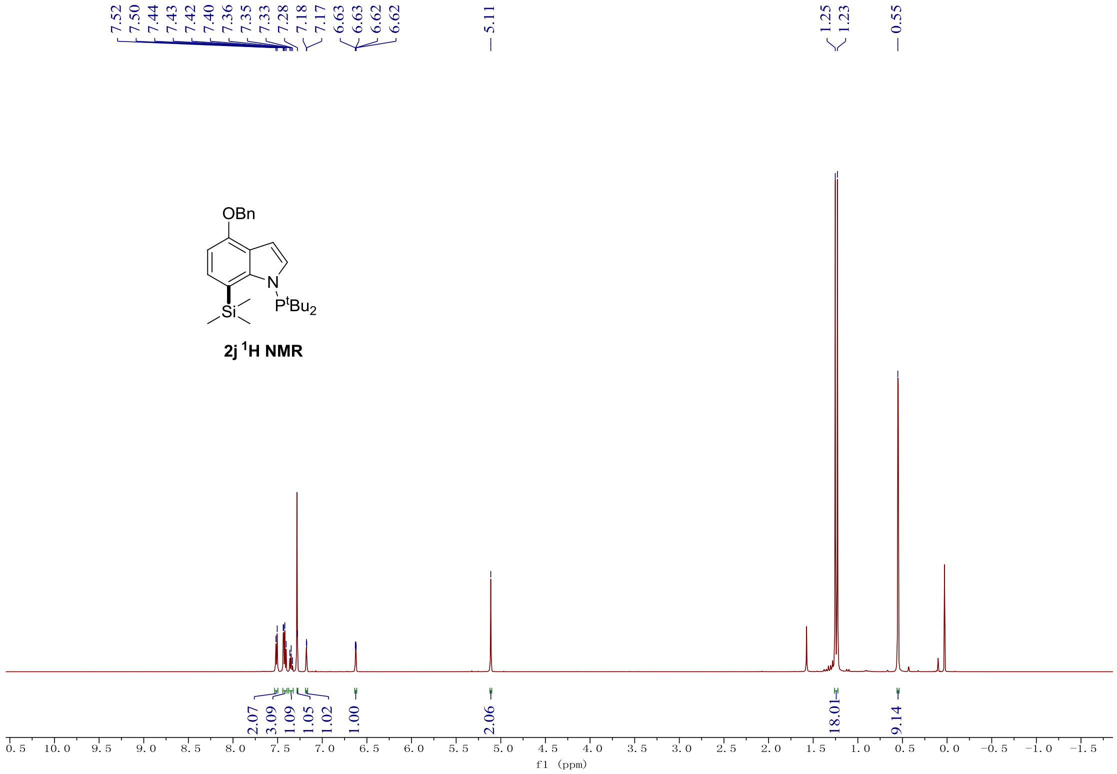

Supplementary Figure 32. ^{1}H NMR of compound 2j.

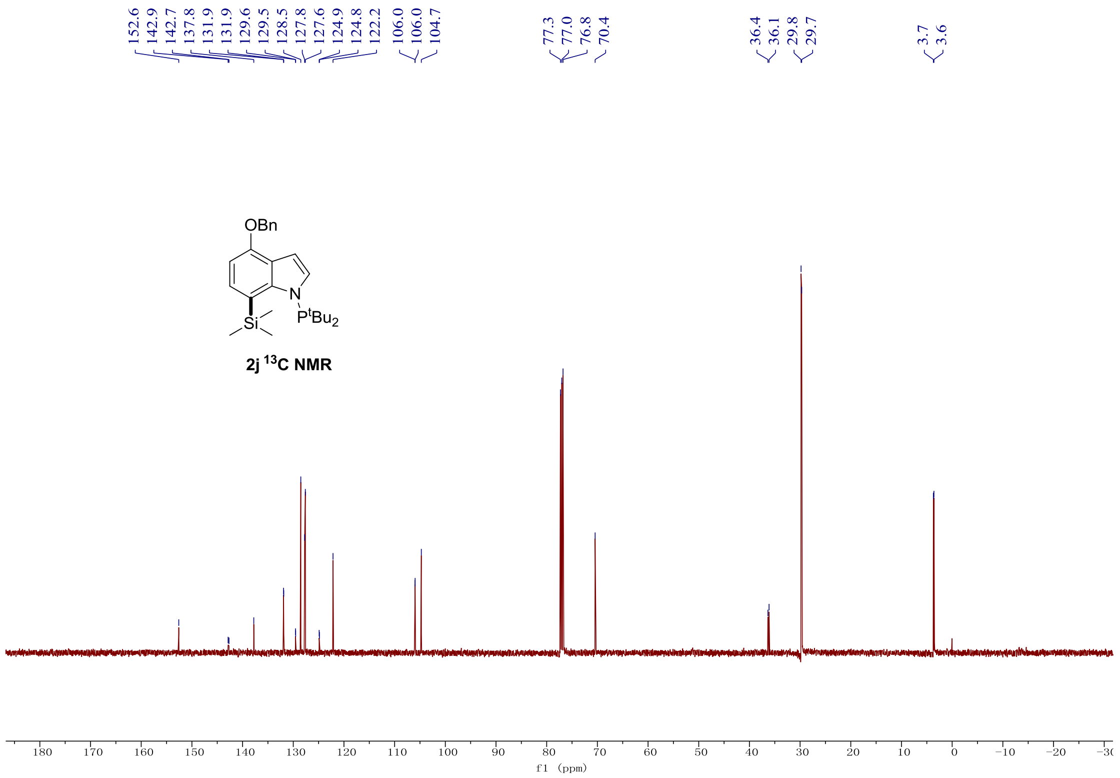

Supplementary Figure 33. ^{13}C NMR of compound 2j.

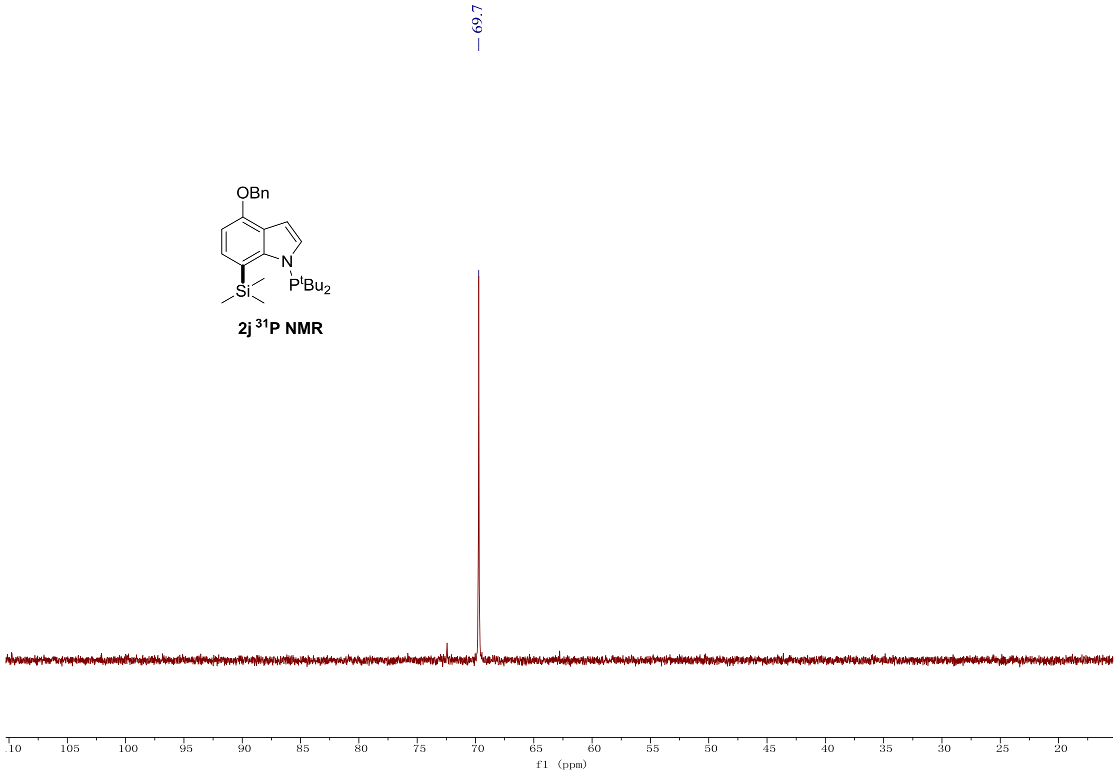

Supplementary Figure 34. ^{31}P NMR of compound 2j.

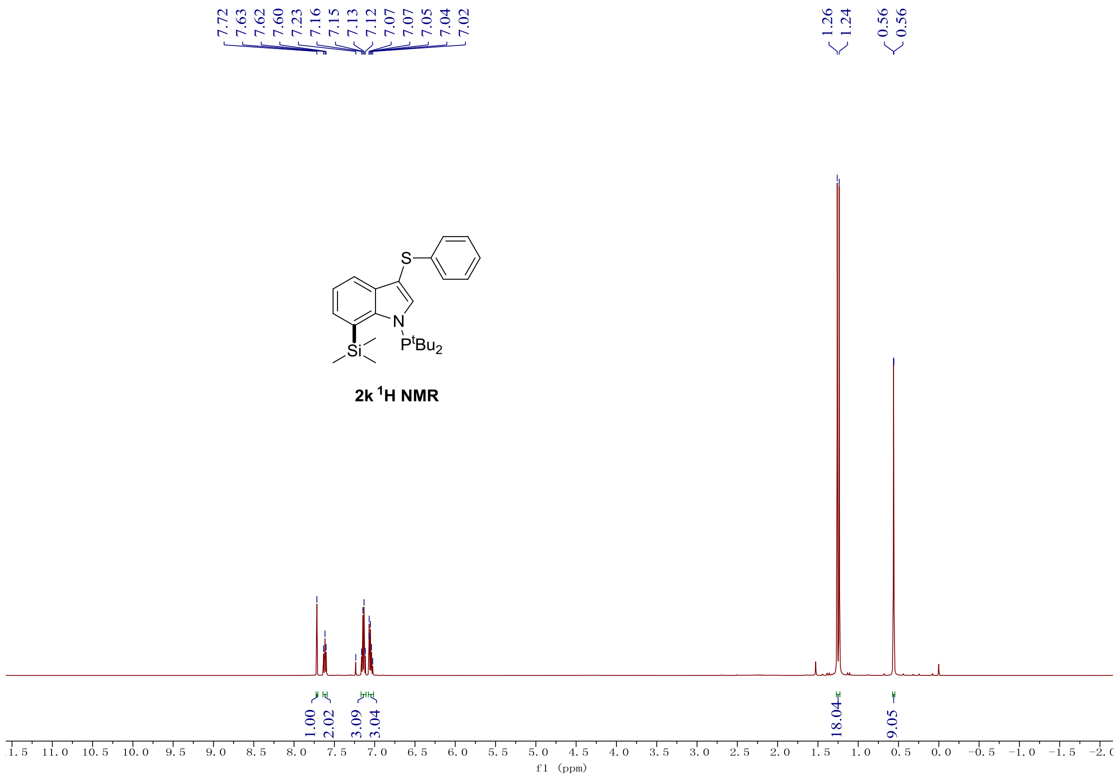

Supplementary Figure 35. ^{1}H NMR of compound 2k.

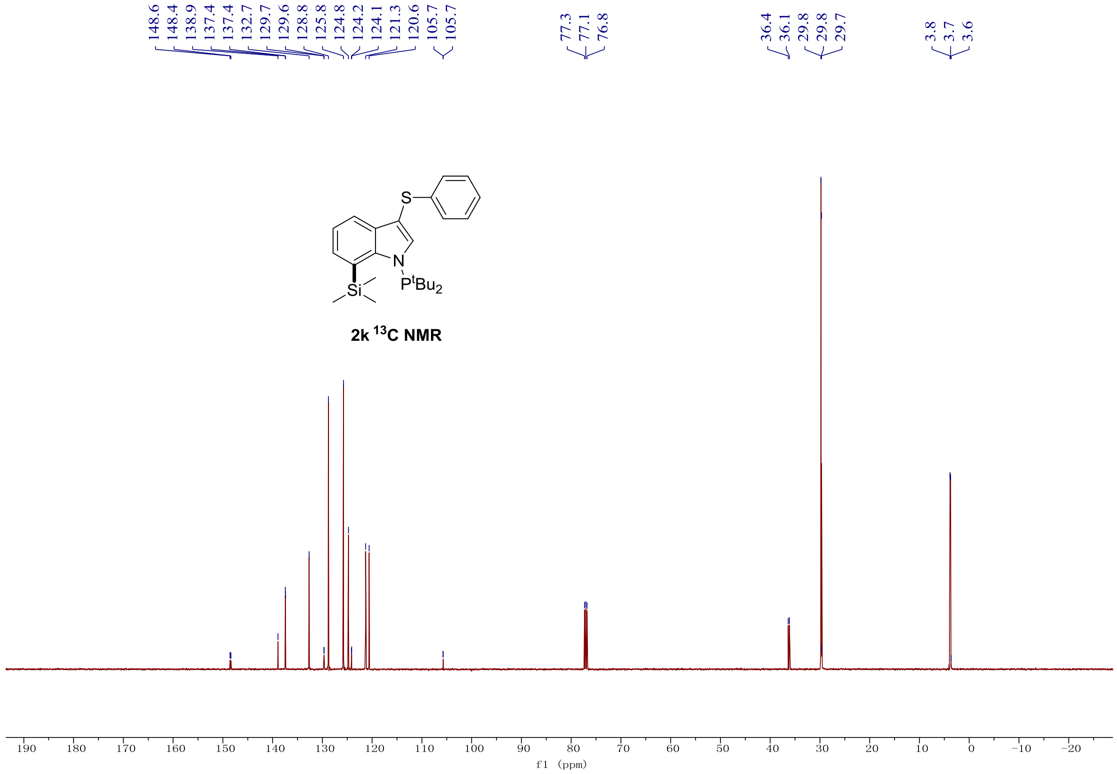

Supplementary Figure 36. ^{13}C NMR of compound 2k.

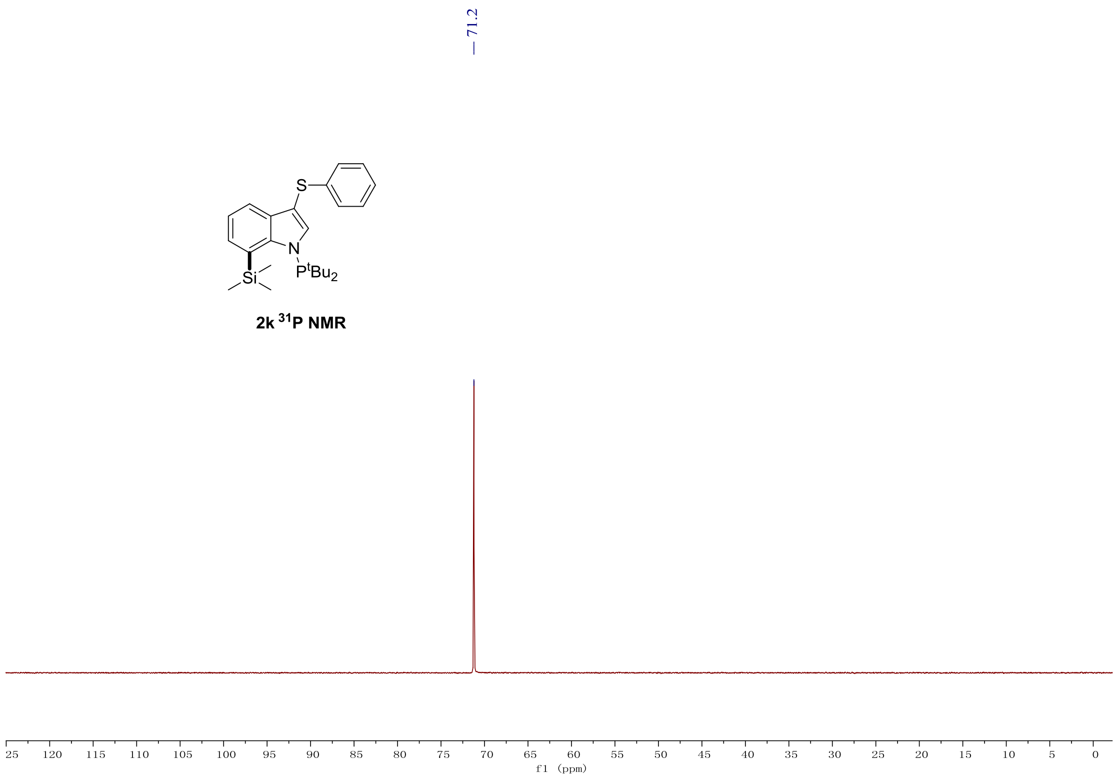

Supplementary Figure 37. ^{31}P NMR of compound 2k.

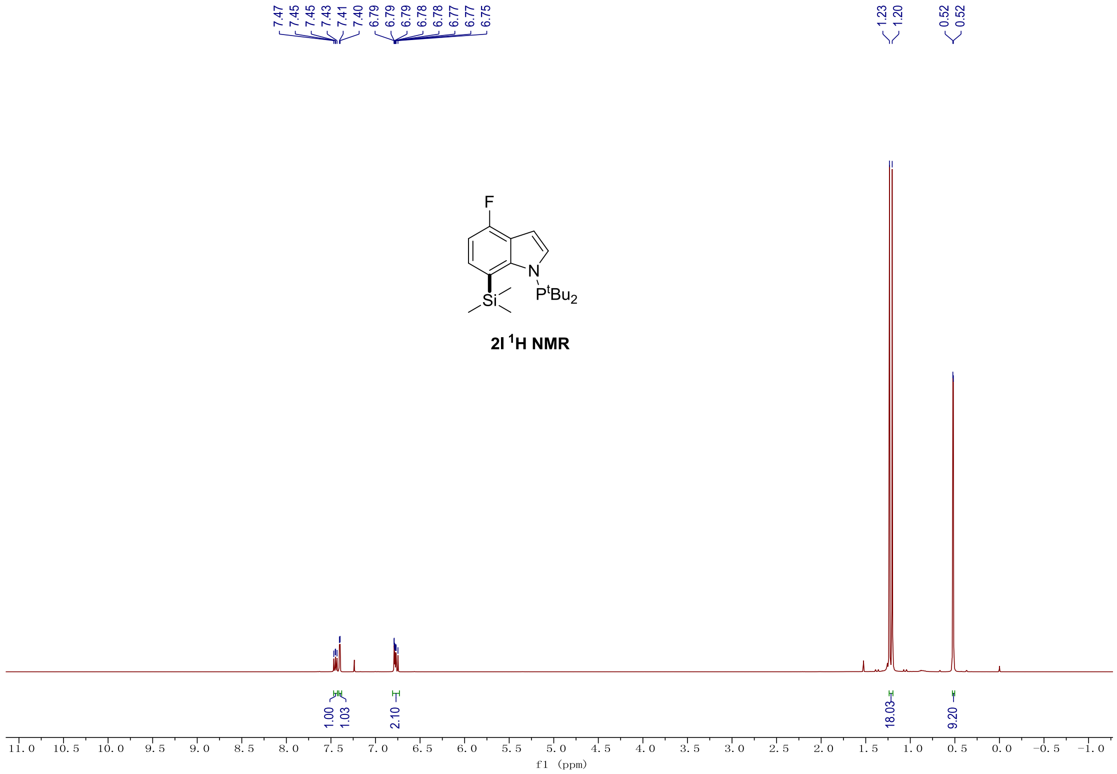

Supplementary Figure 38. ^{1}H NMR of compound 2l.

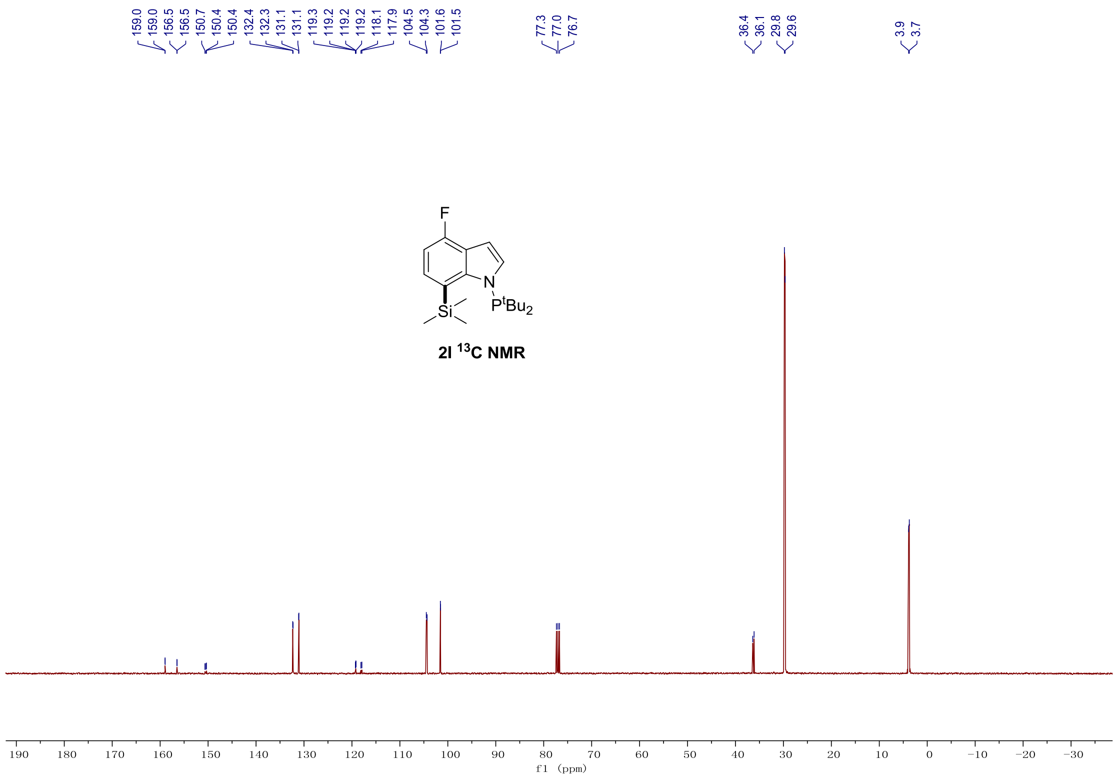

Supplementary Figure 39. ^{13}C NMR of compound 2l.

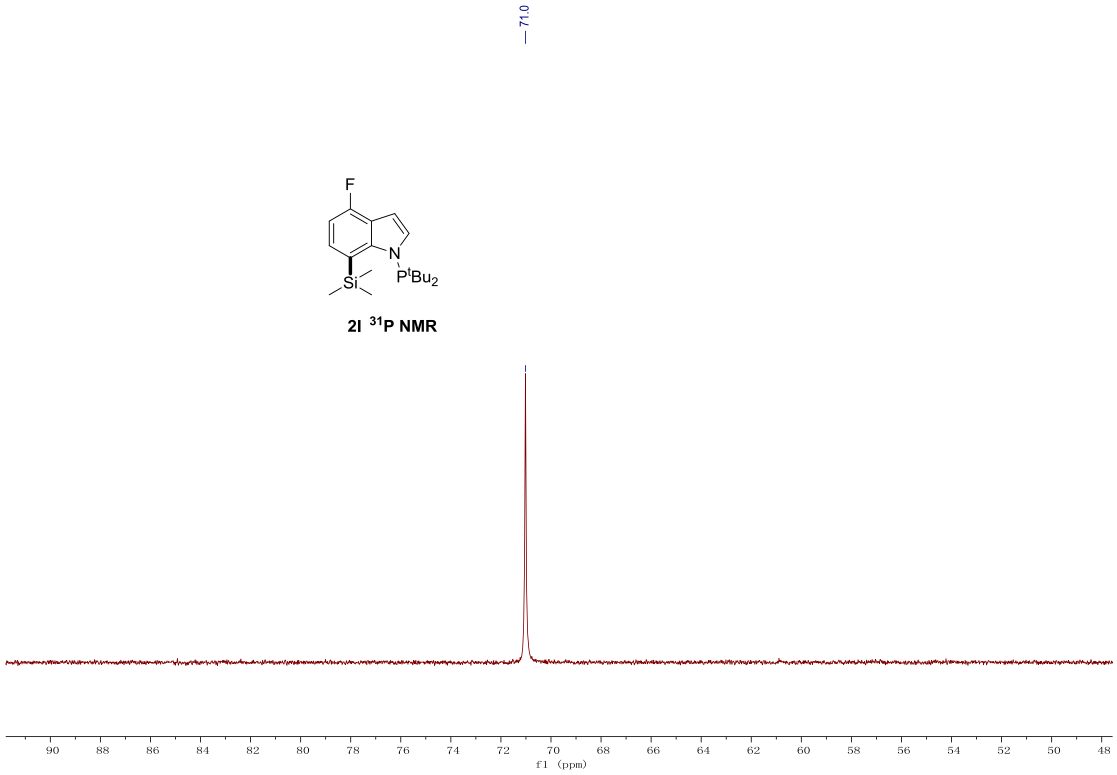

Supplementary Figure 40. ^{31}P NMR of compound 2l.

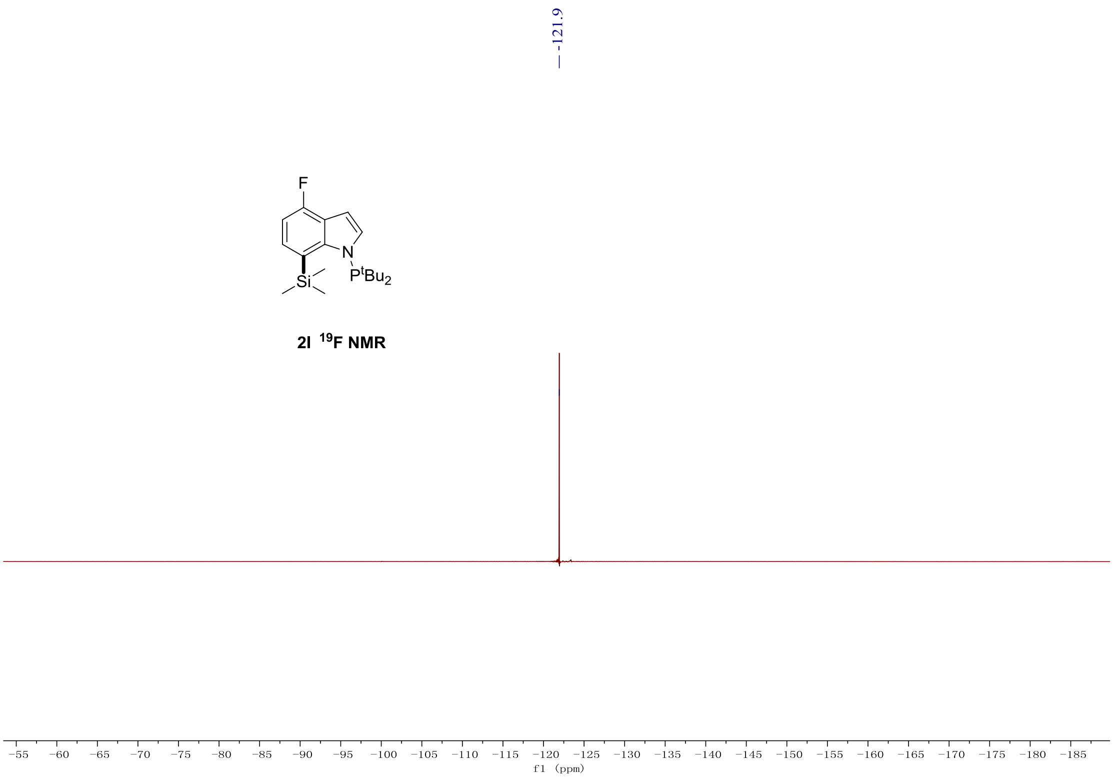

Supplementary Figure 41. ^{19}F NMR of compound 2l.

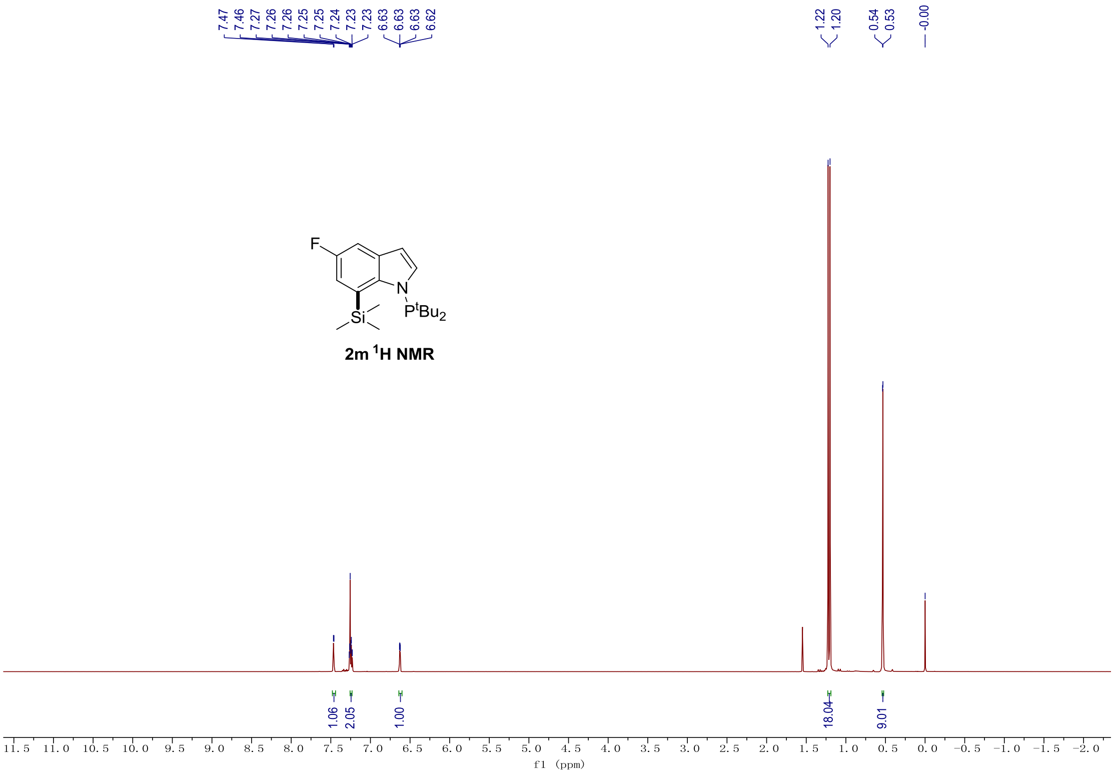

Supplementary Figure 42. ^{1}H NMR of compound 2m.

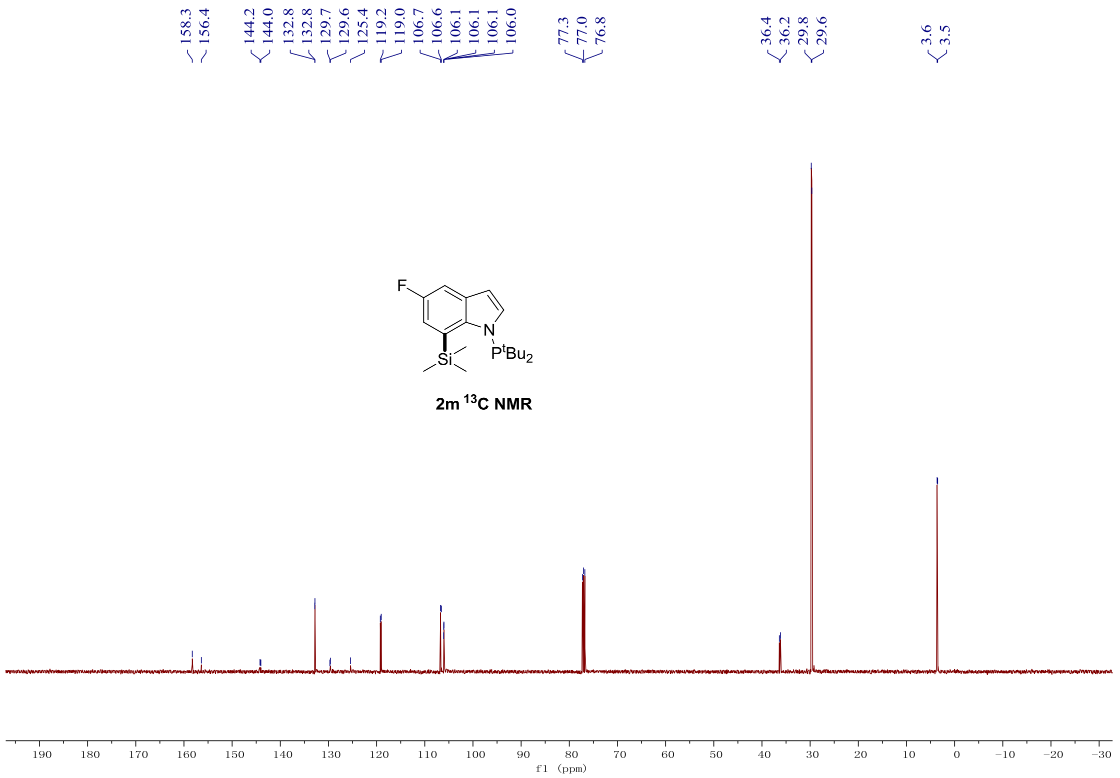

Supplementary Figure 43. ^{13}C NMR of compound 2m.

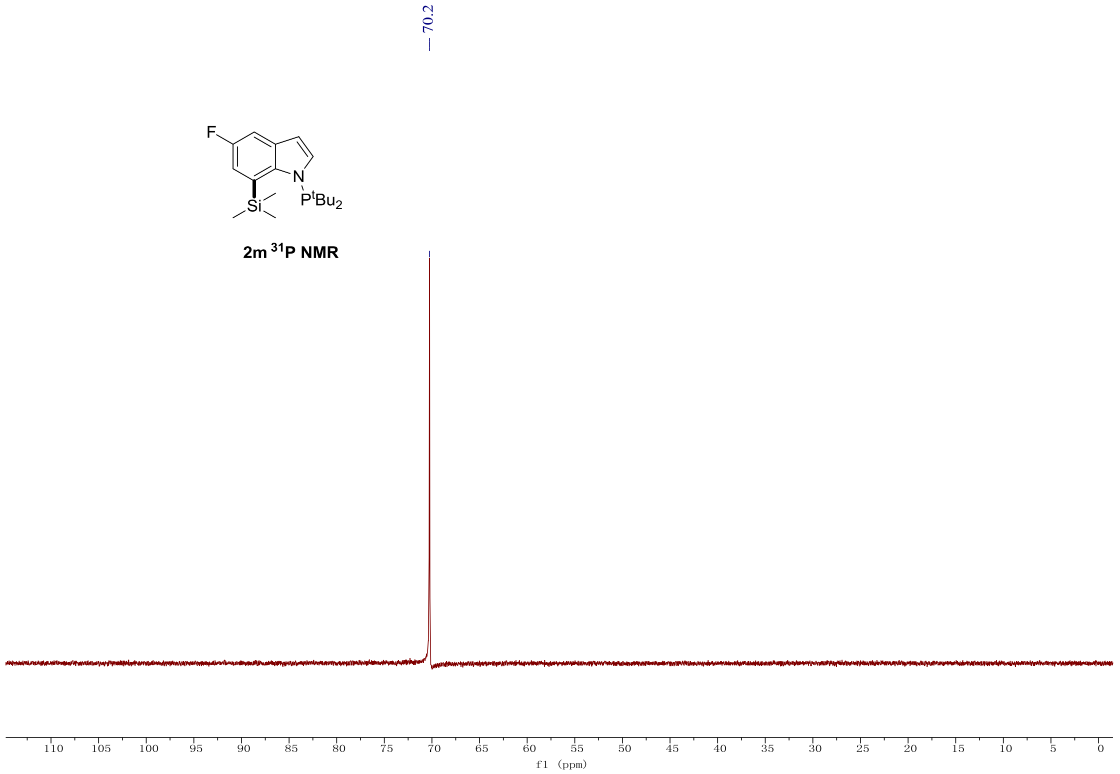

Supplementary Figure 44. ^{31}P NMR of compound 2m.

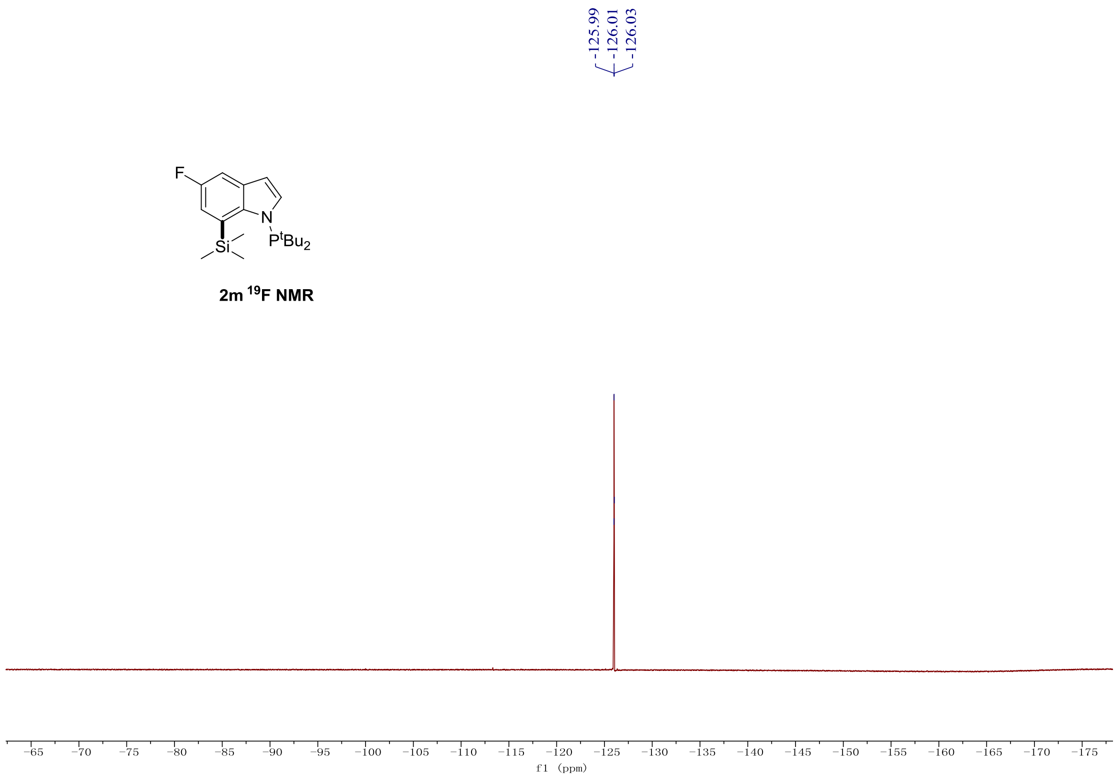

Supplementary Figure 45. ^{19}F NMR of compound 2m.

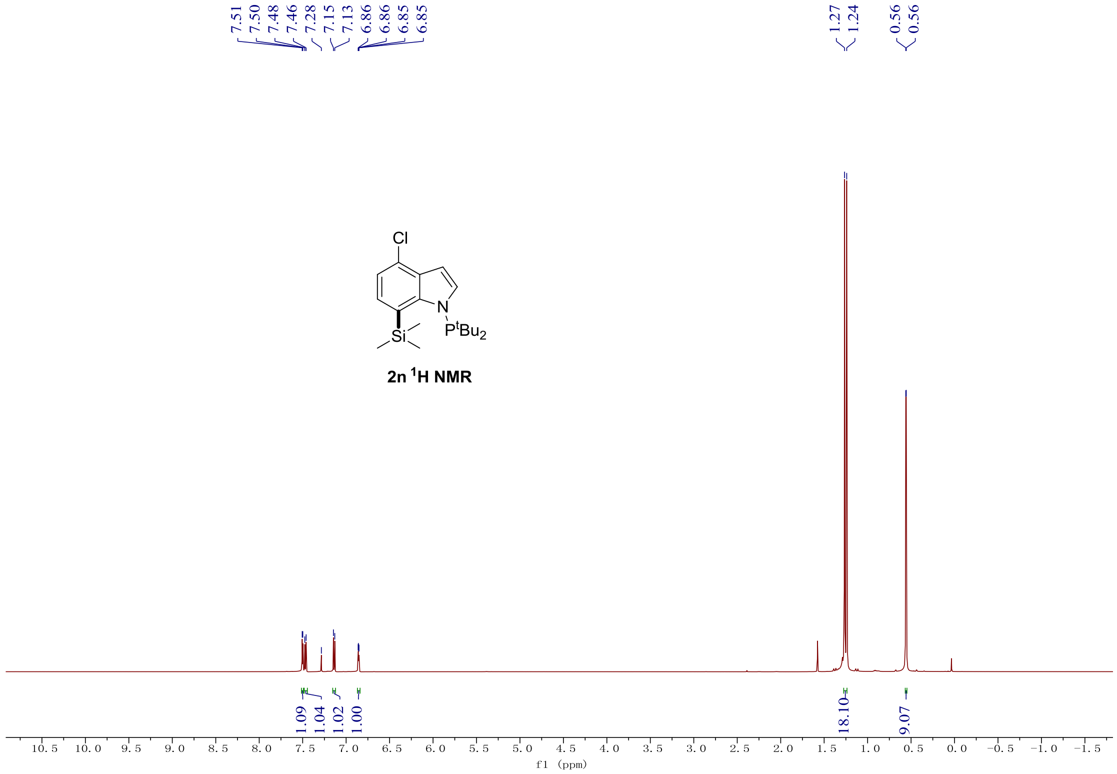

Supplementary Figure 46. ^{1}H NMR of compound 2n.

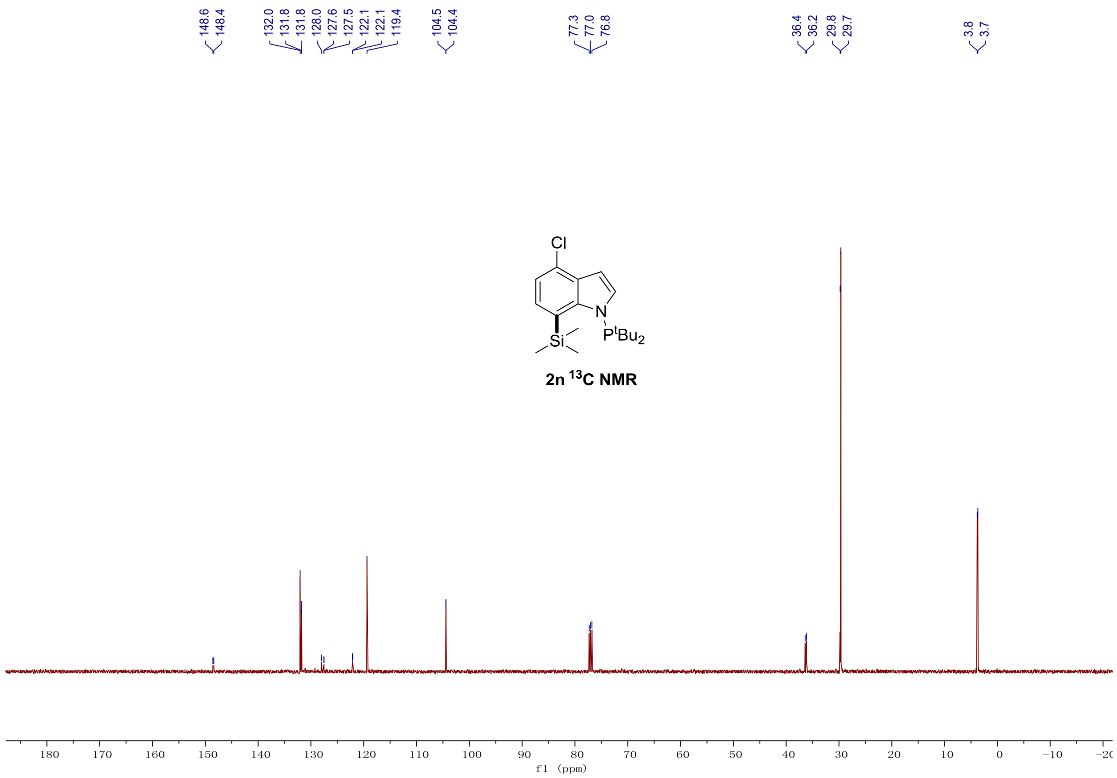

Supplementary Figure 47. ^{13}C NMR of compound 2n.

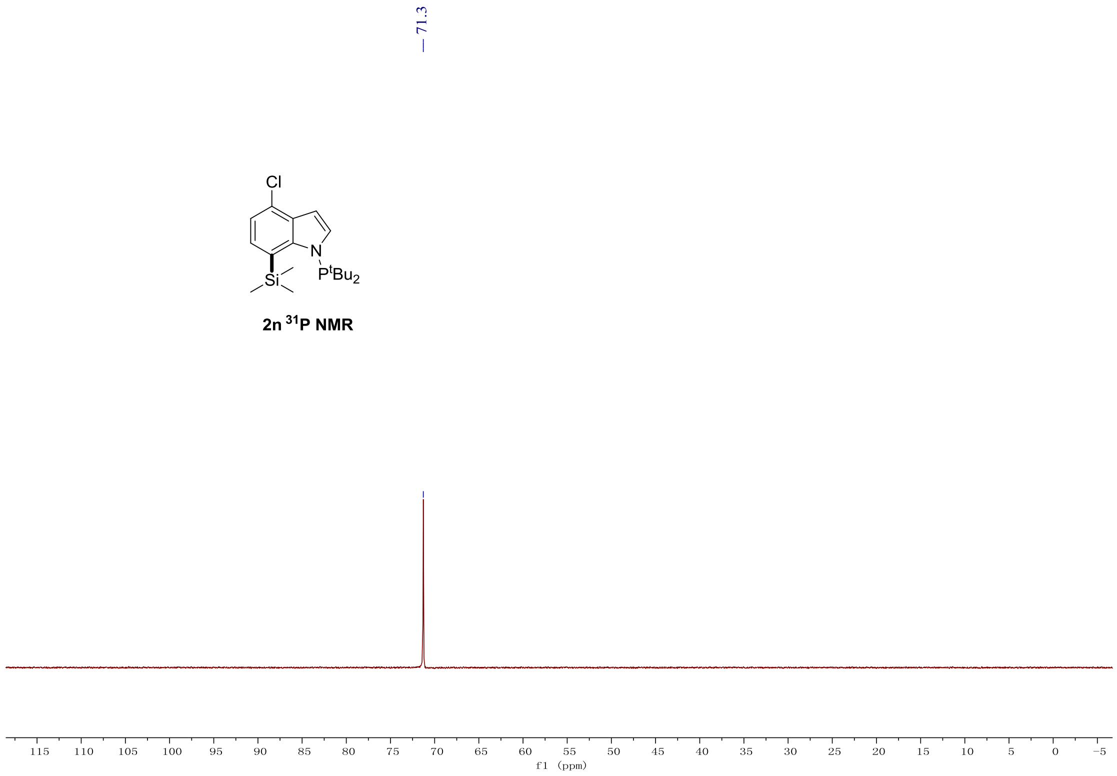

Supplementary Figure 48. ^{31}P NMR of compound 2n.

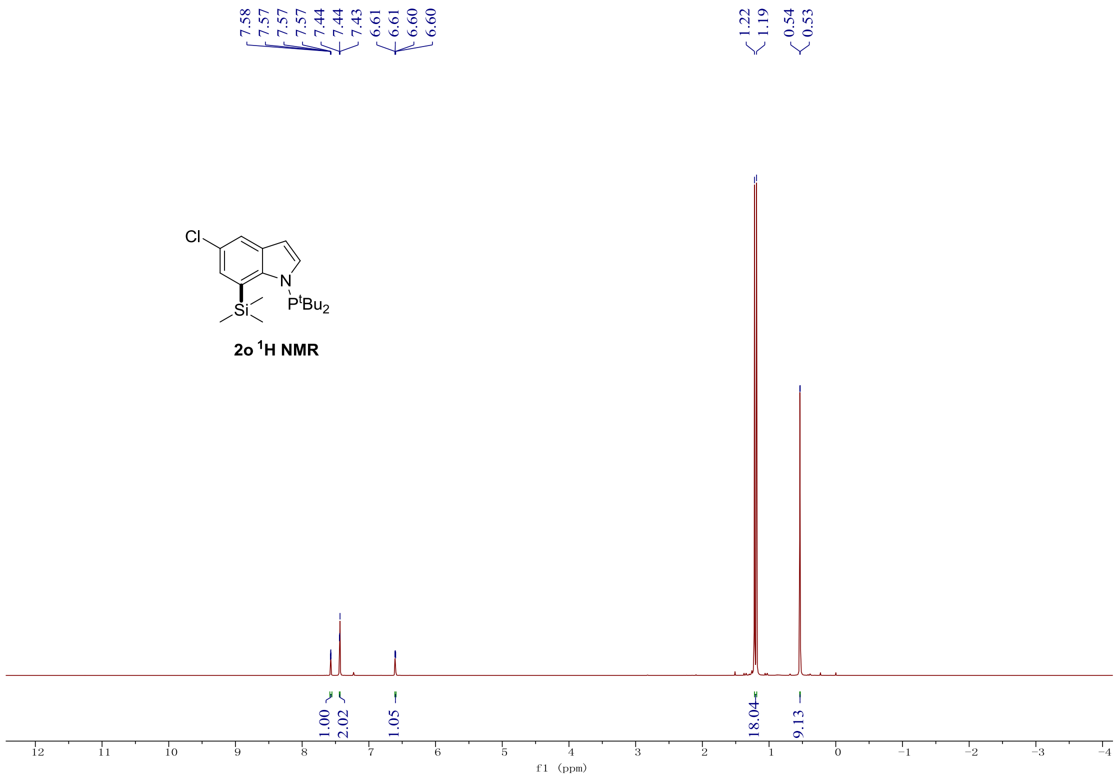

Supplementary Figure 49. ^{1}H NMR of compound 2o.

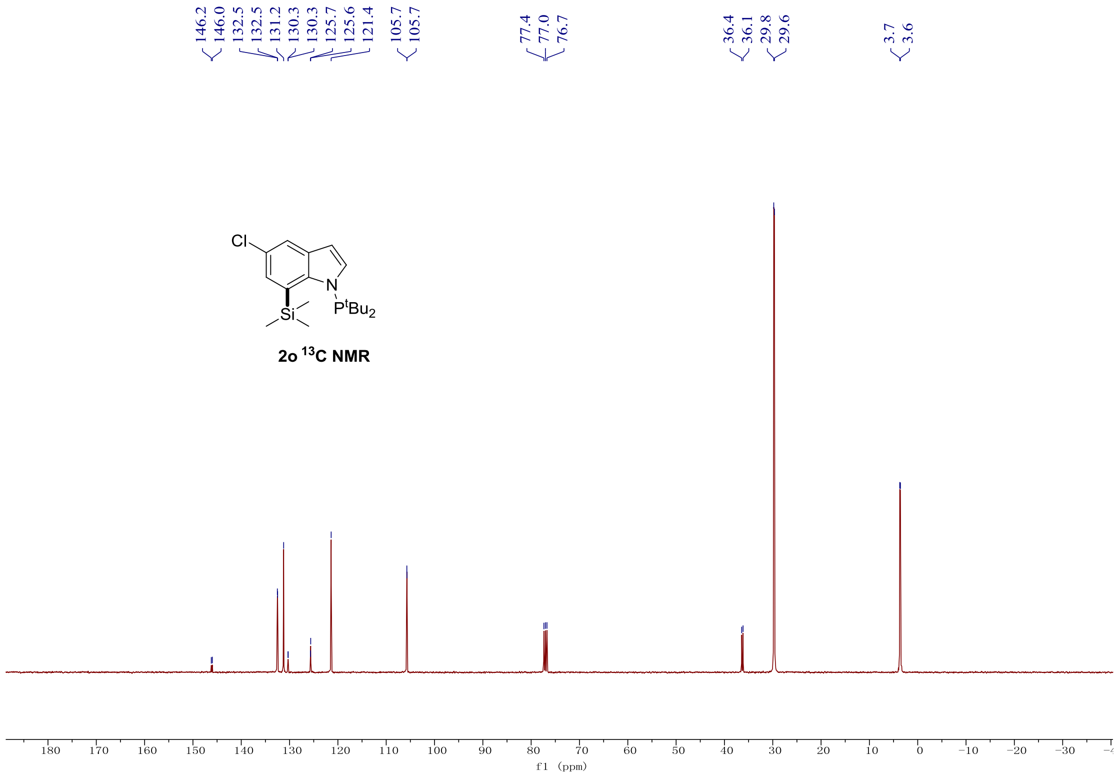

Supplementary Figure 50. ^{13}C NMR of compound 2o.

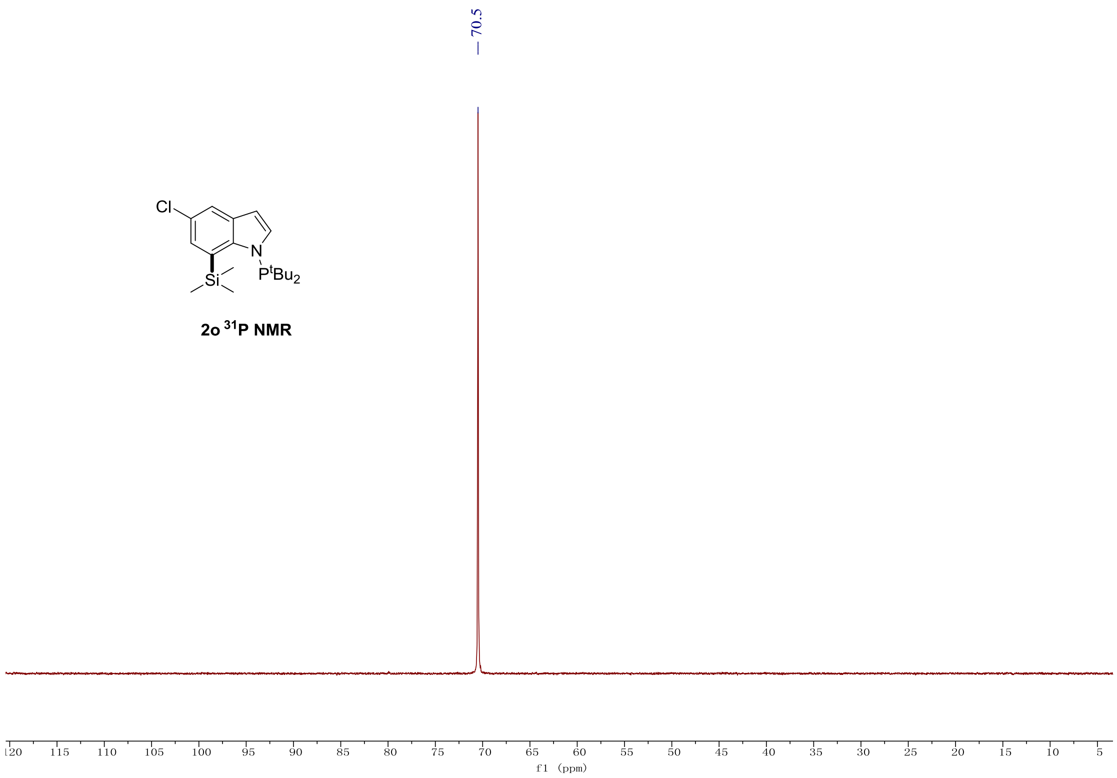

Supplementary Figure 51. ^{31}P NMR of compound 2o.

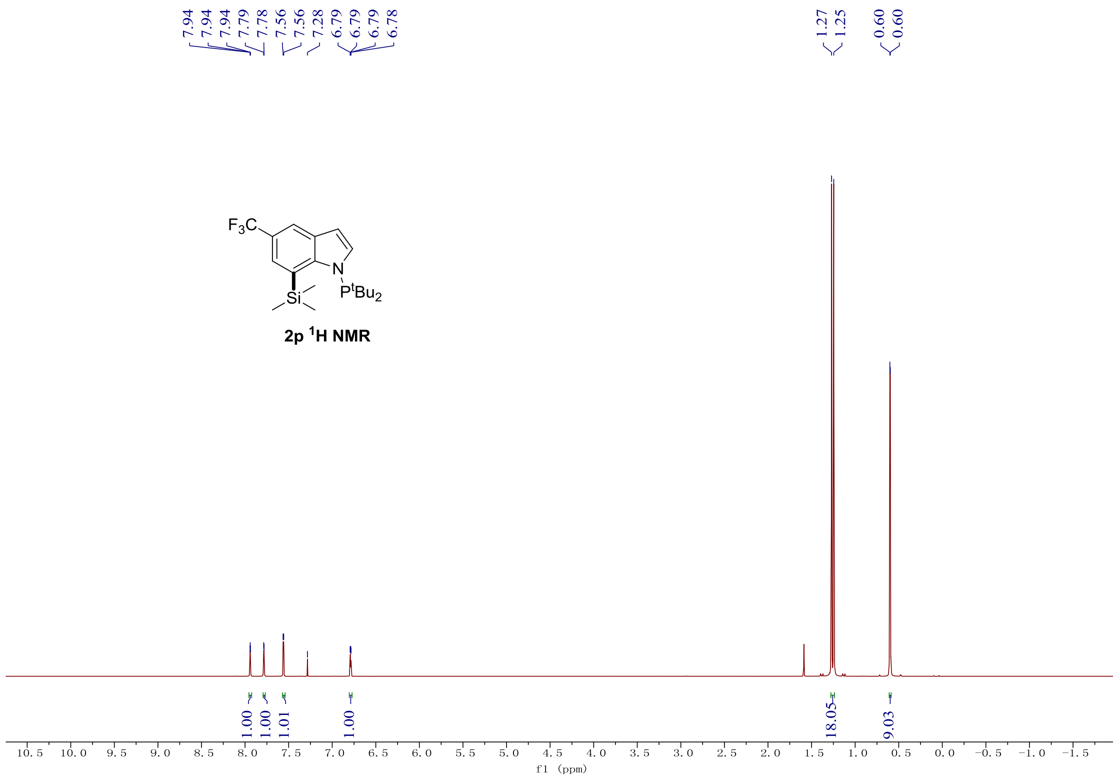

Supplementary Figure 52. ^{1}H NMR of compound 2p.

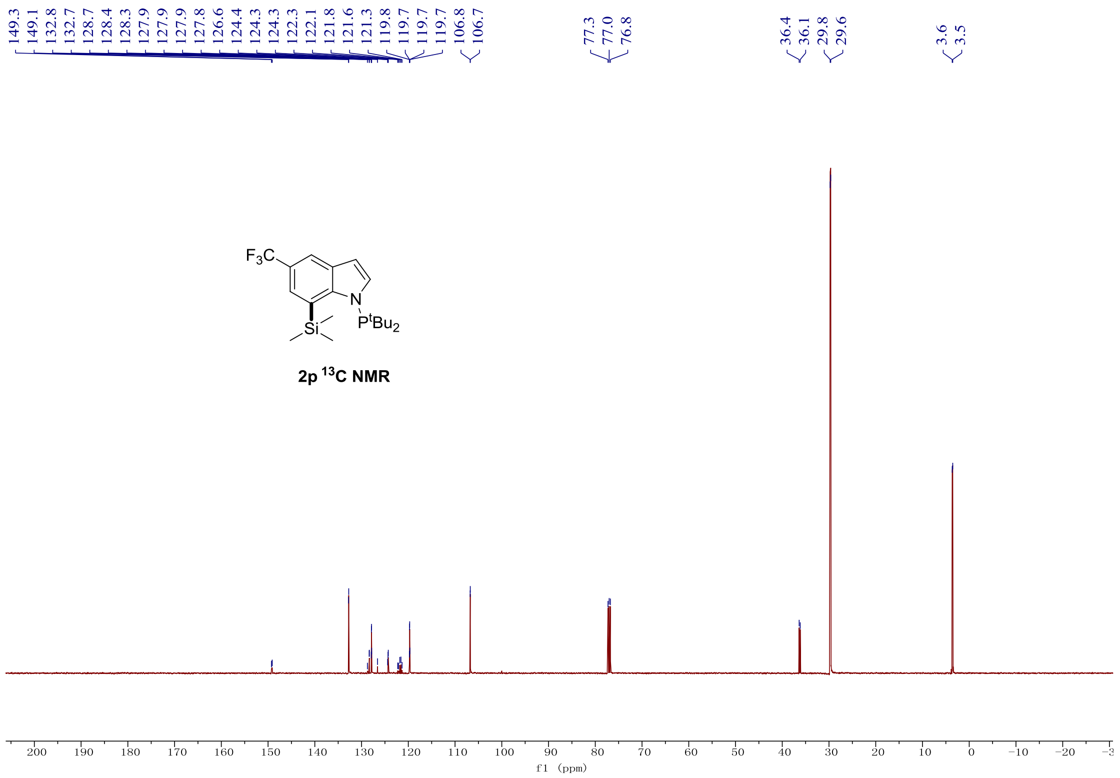

Supplementary Figure 53. ^{13}C NMR of compound 2p.

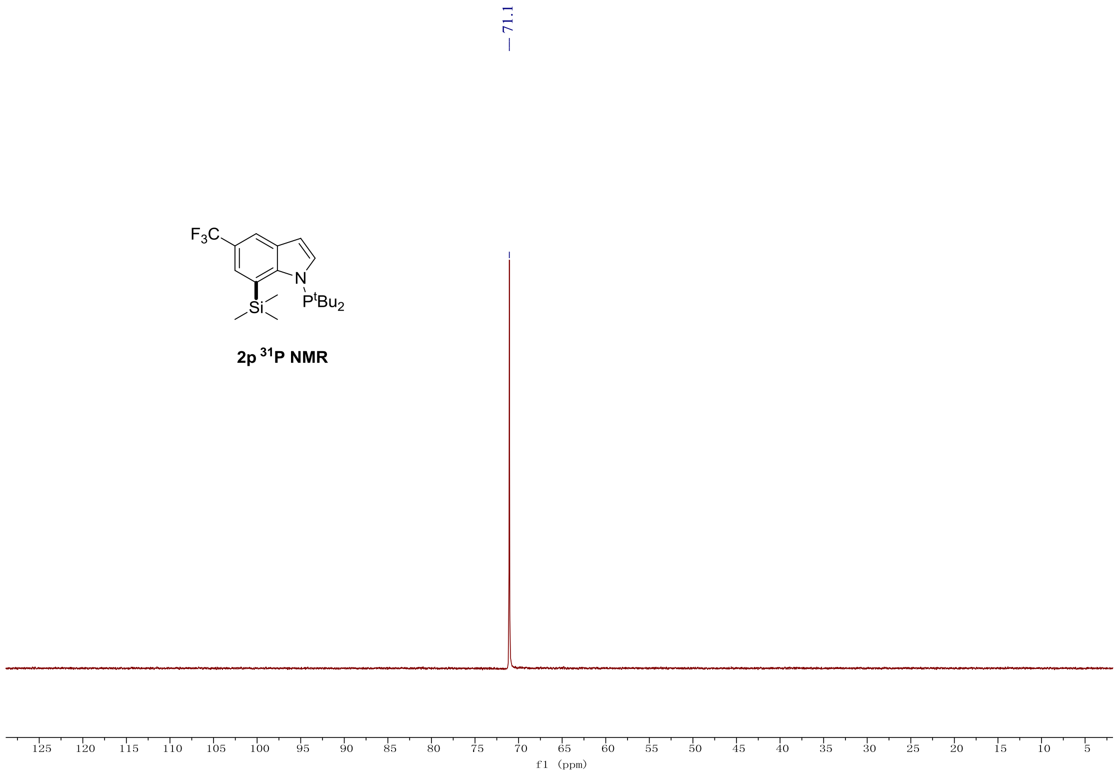

Supplementary Figure 54. ^{19}F NMR of compound 2p.

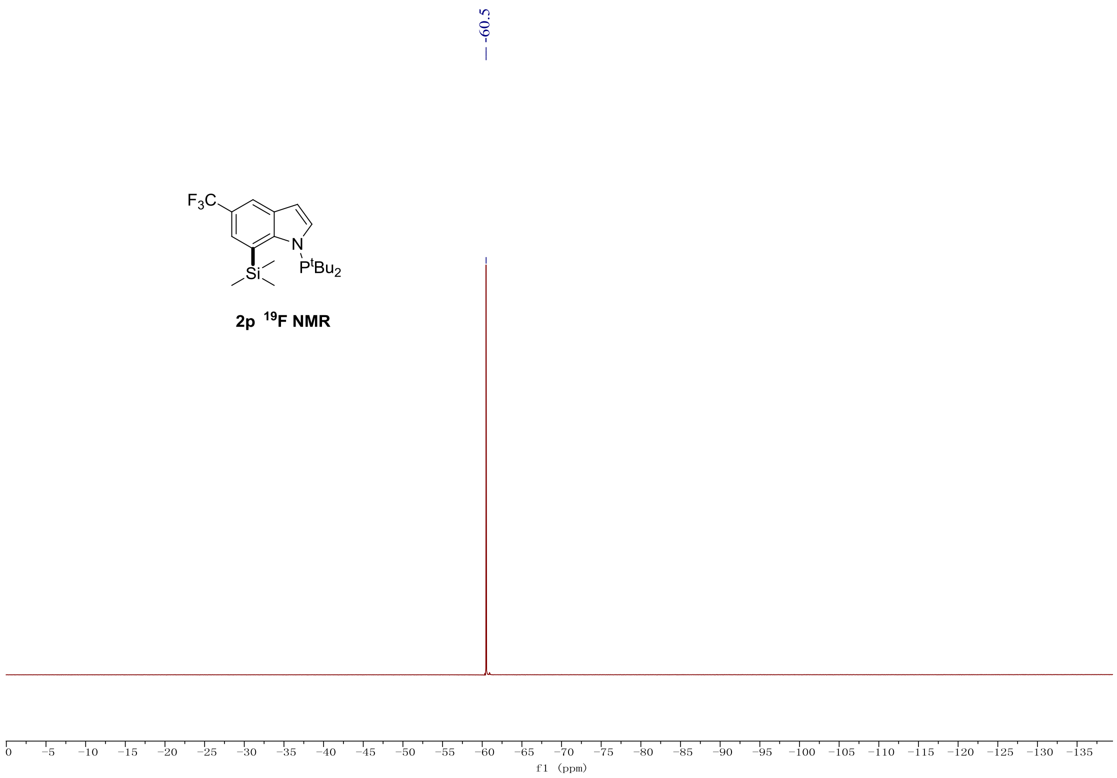

Supplementary Figure 55. ^{1}H NMR of compound 2p.

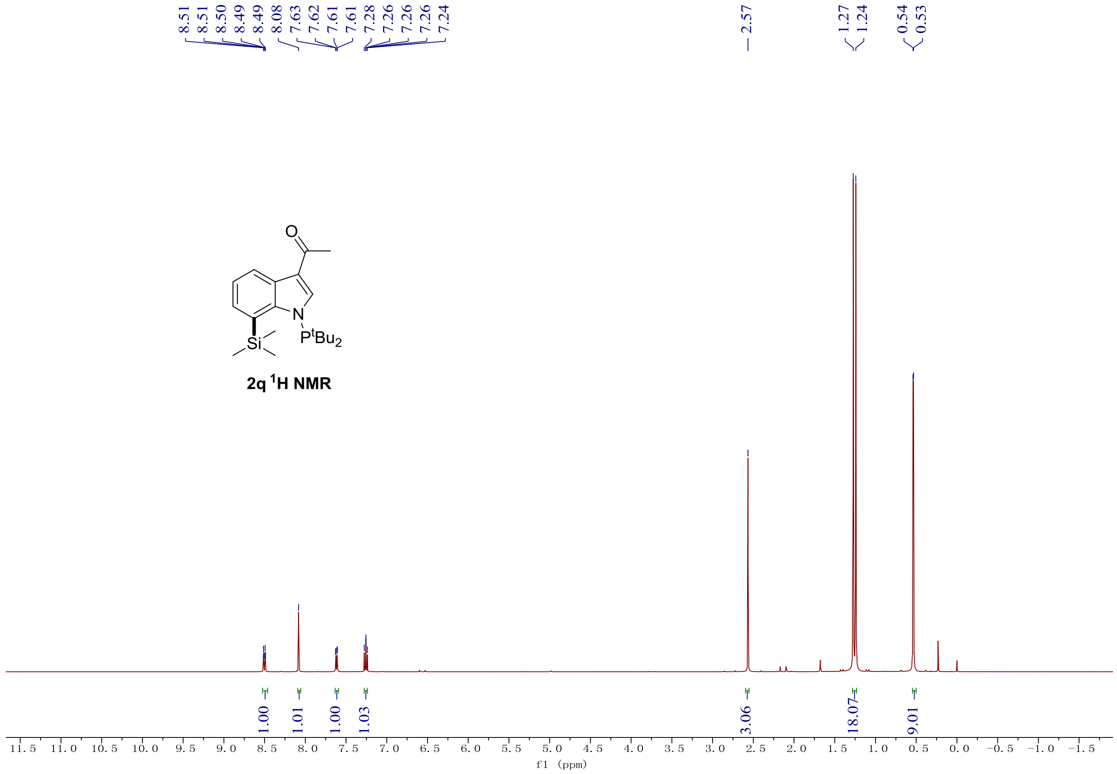

Supplementary Figure 56. ^{1}H NMR of compound 2q.

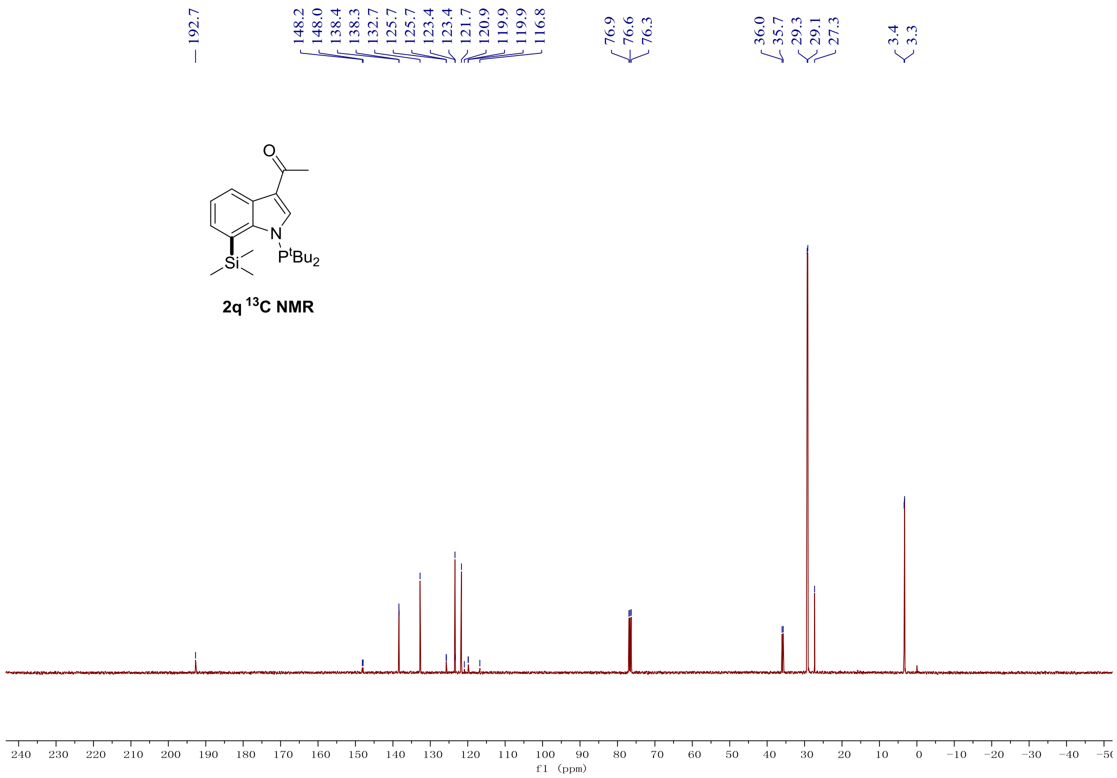

Supplementary Figure 57. ^{13}C NMR of compound 2q.

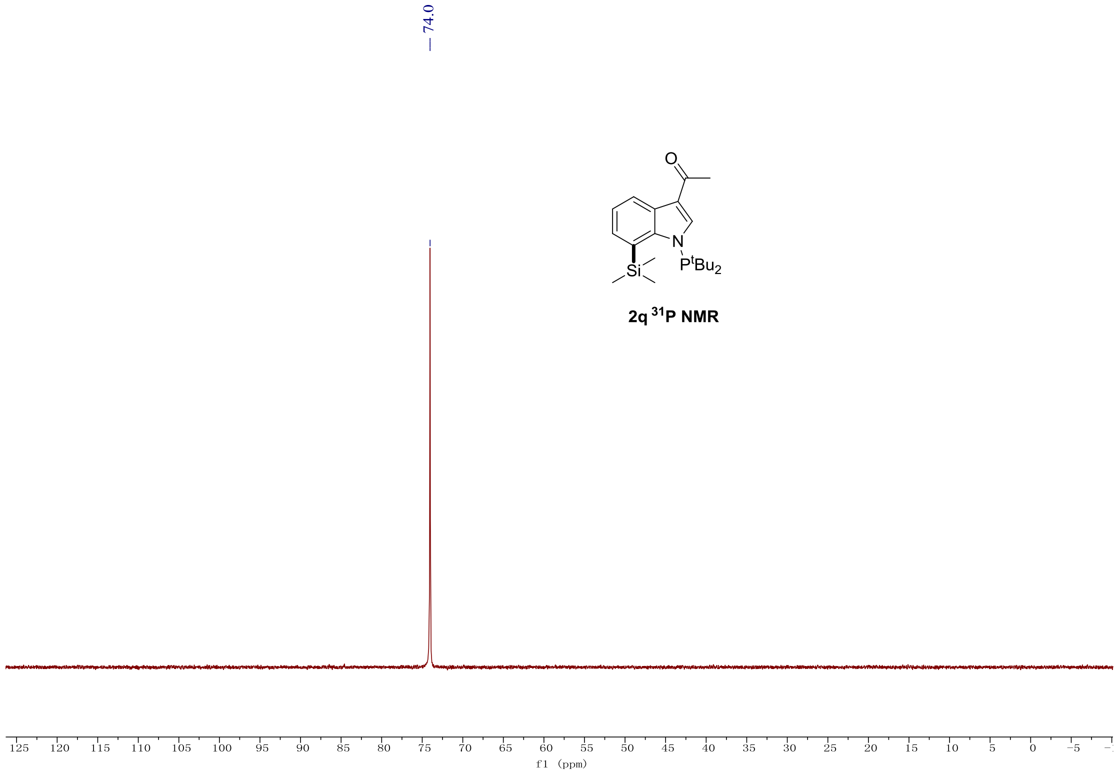

Supplementary Figure 58. ^{31}P NMR of compound 2q.

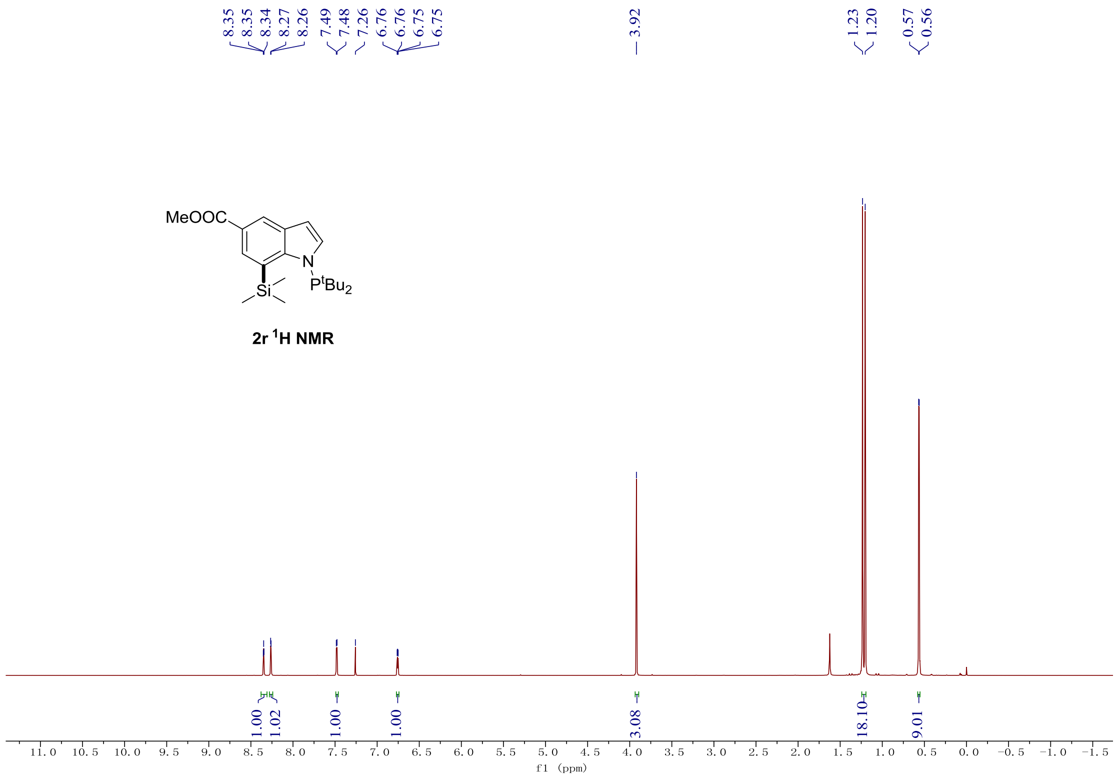

Supplementary Figure 59. ^{1}H NMR of compound 2r.

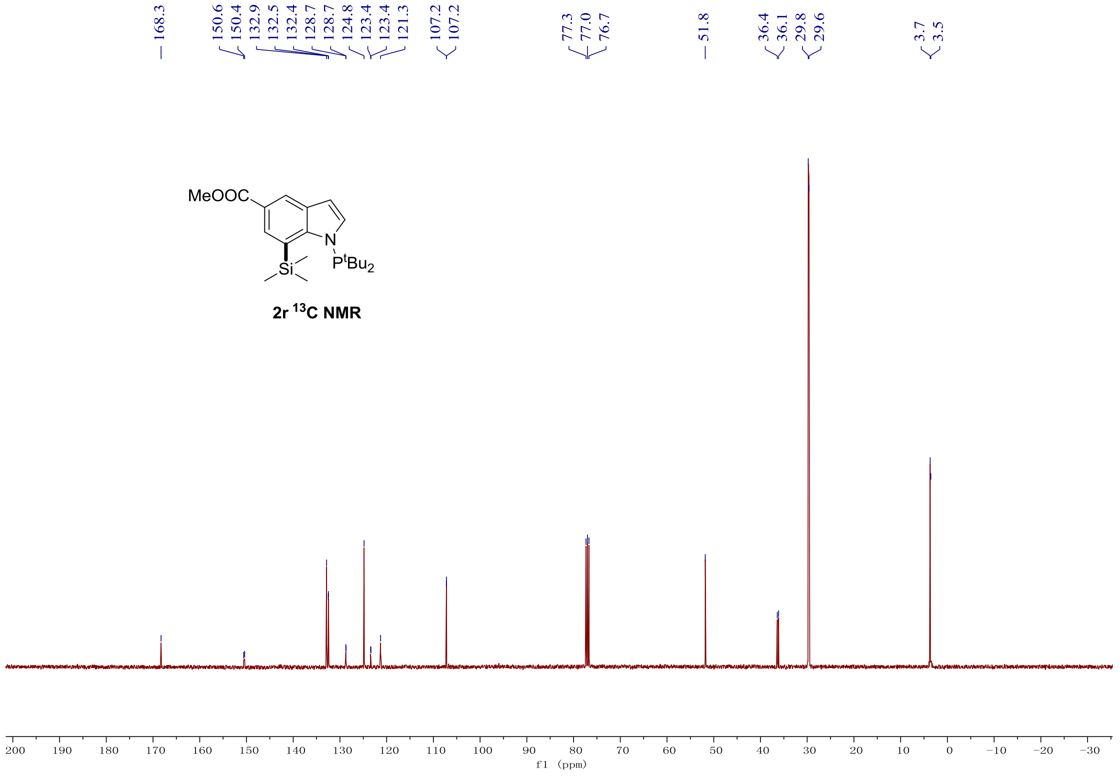

Supplementary Figure 60. ^{13}C NMR of compound 2r.

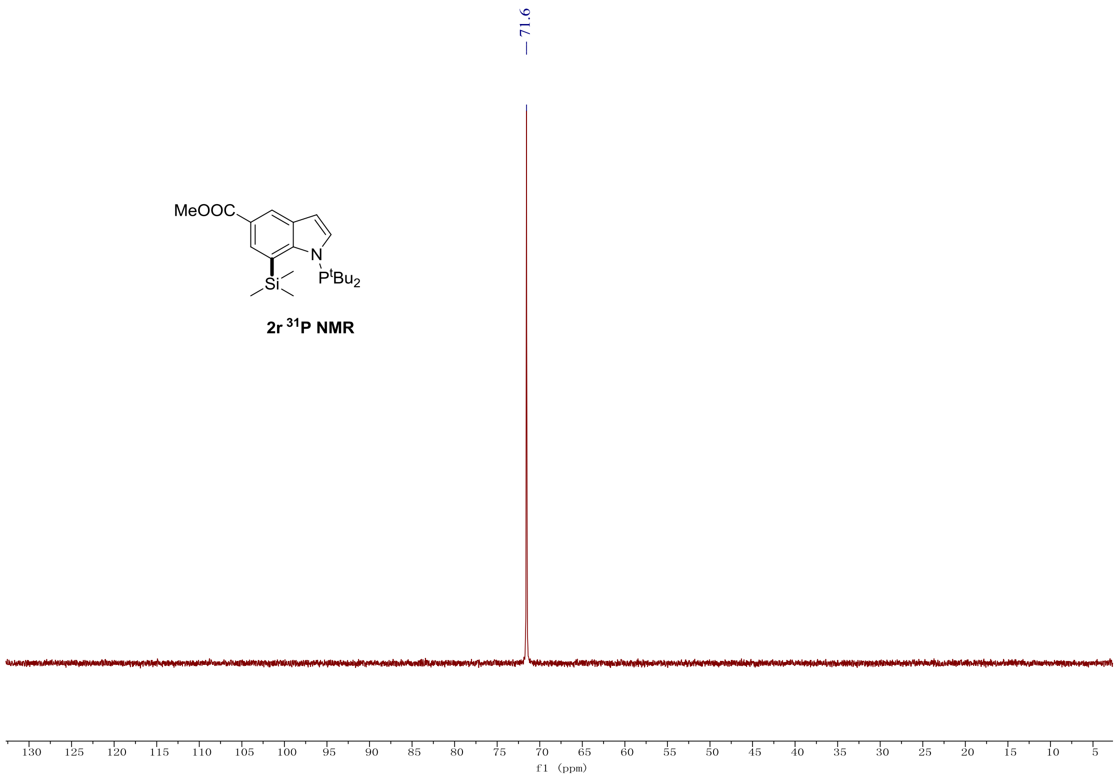

Supplementary Figure 61. ^{31}P NMR of compound 2r.

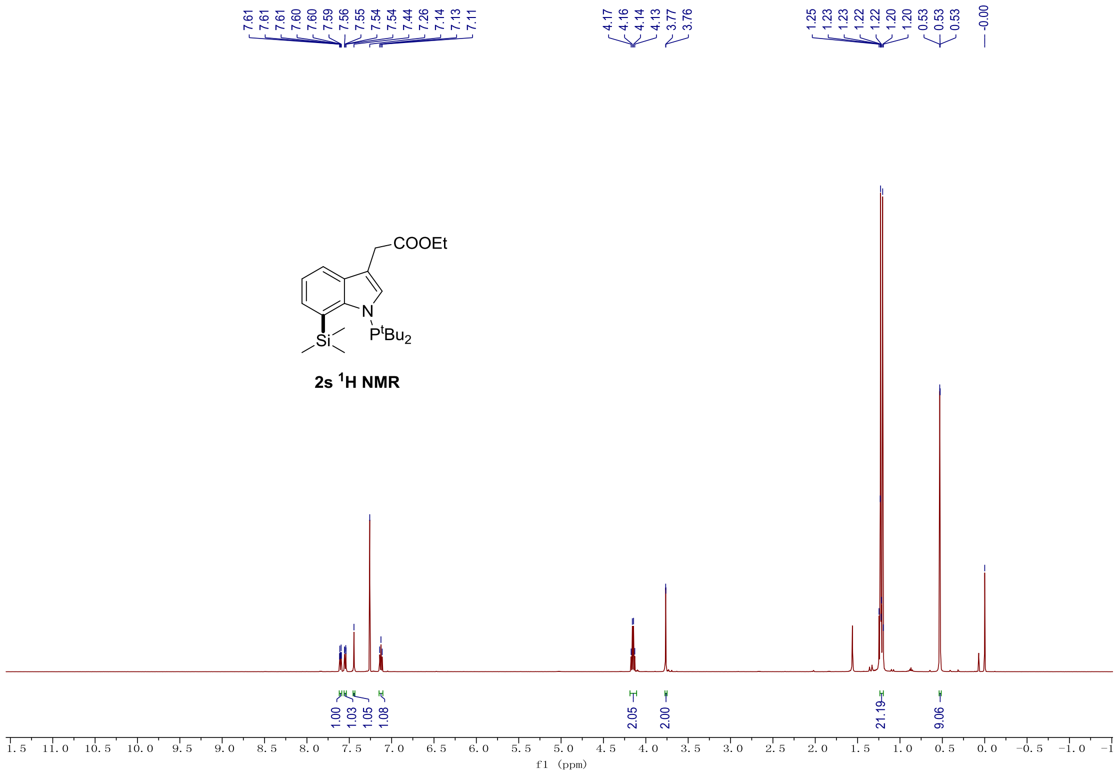

Supplementary Figure 62. ^{1}H NMR of compound 2s.

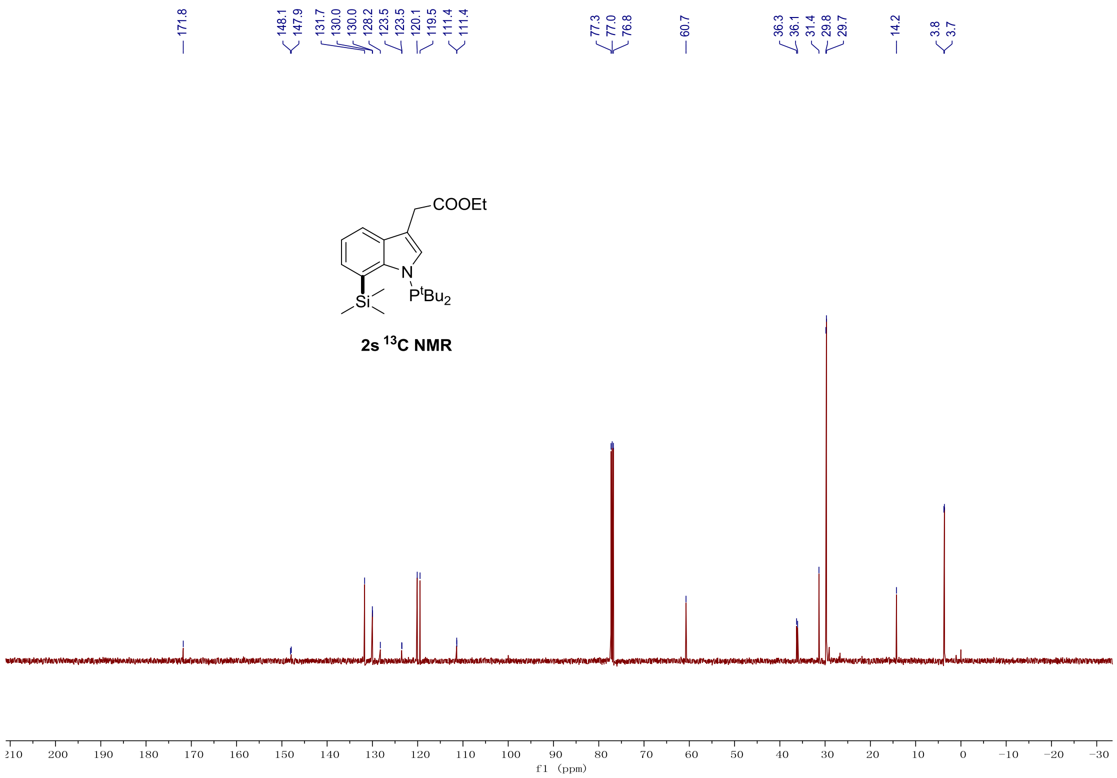

Supplementary Figure 63. ^{13}C NMR of compound 2s.

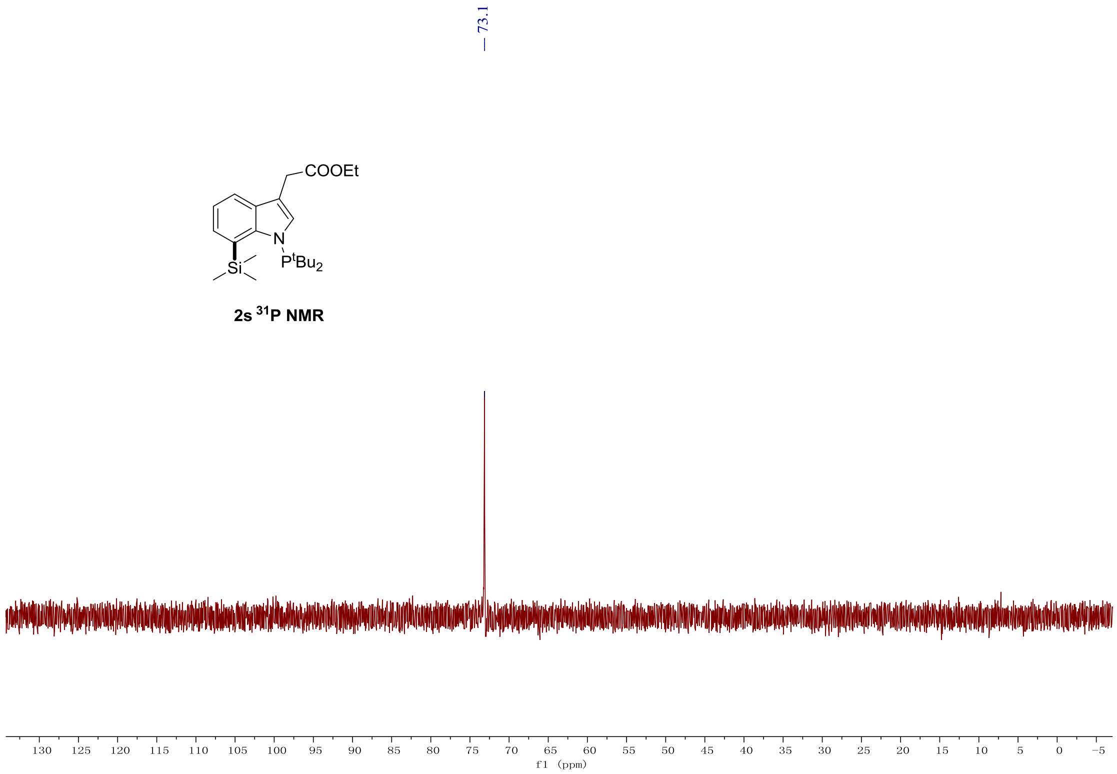

Supplementary Figure 64. ^{31}P NMR of compound 2s.

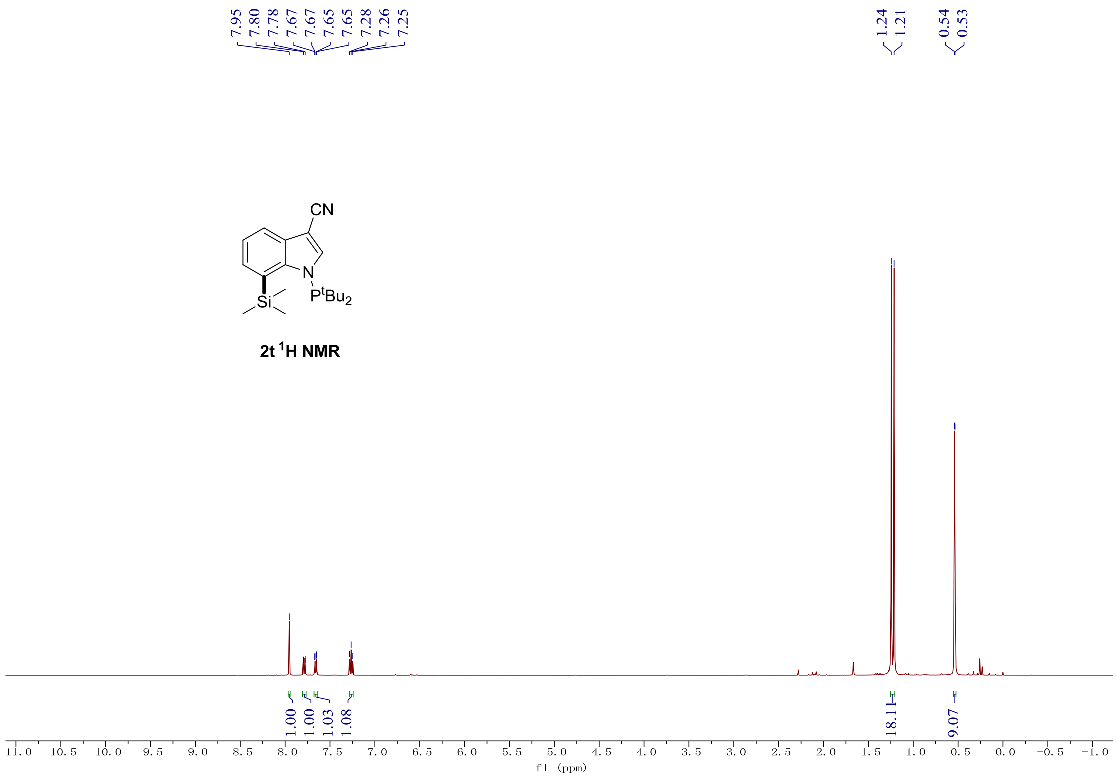

Supplementary Figure 65. ^{1}H NMR of compound 2t.

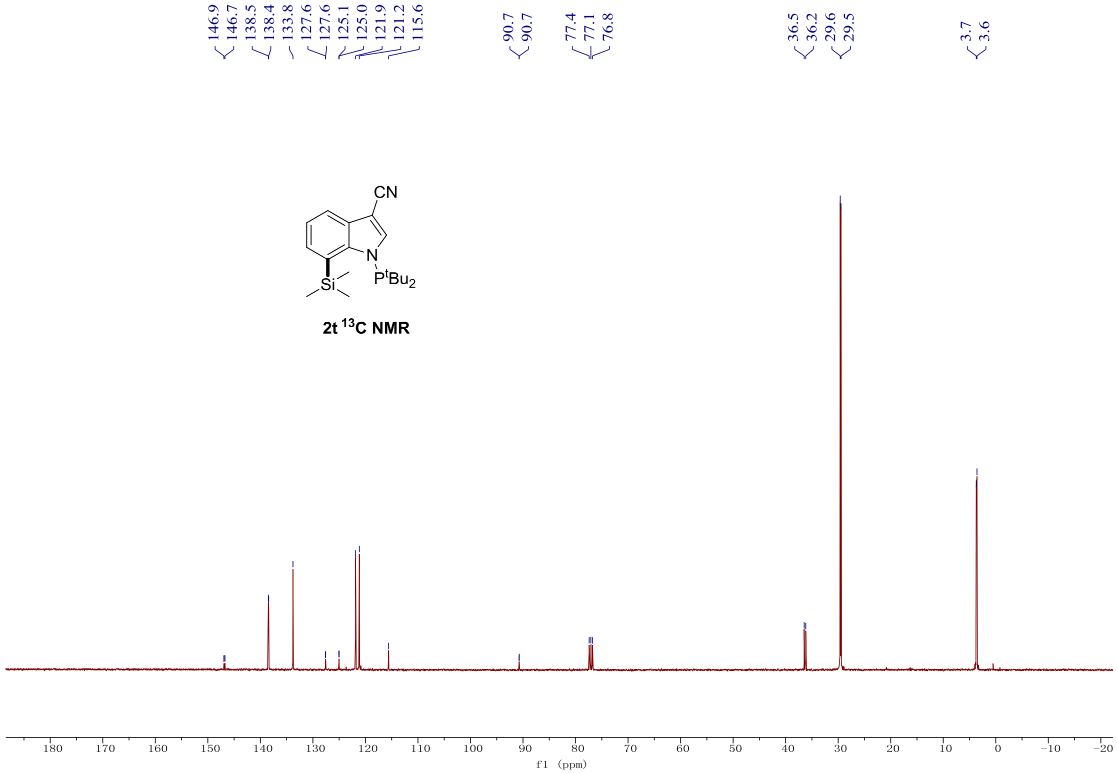

Supplementary Figure 66. ^{13}C NMR of compound 2t.

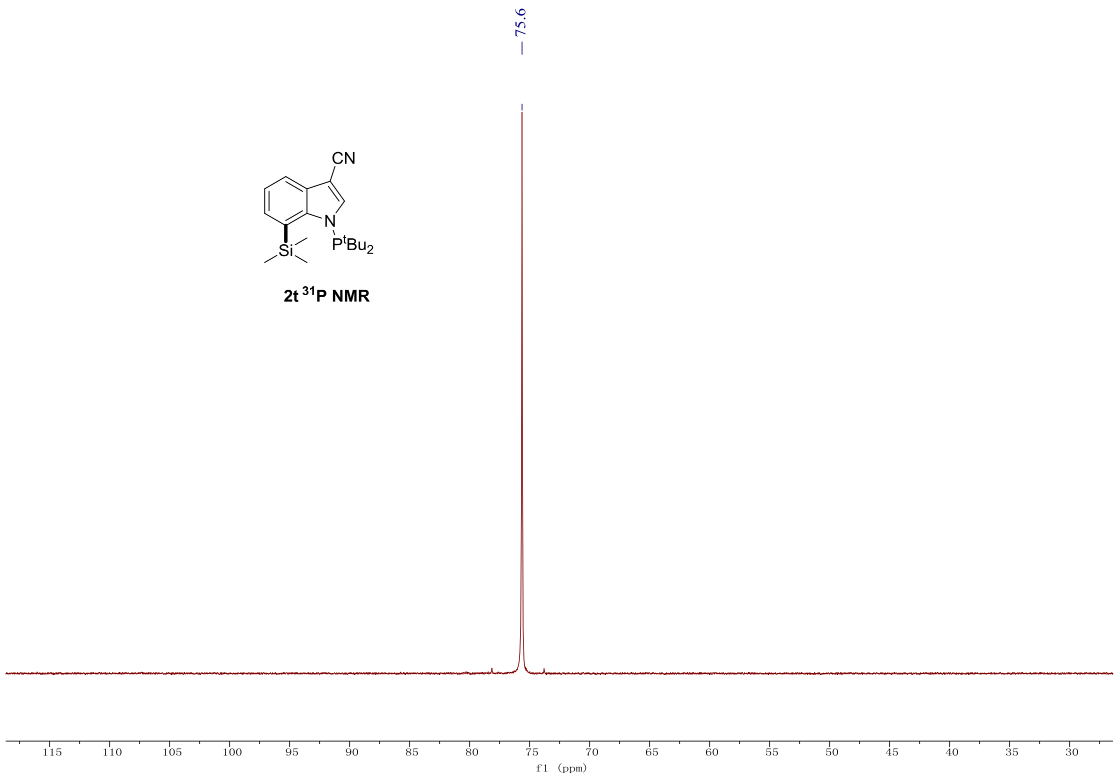

Supplementary Figure 67. ^{31}P NMR of compound 2t.

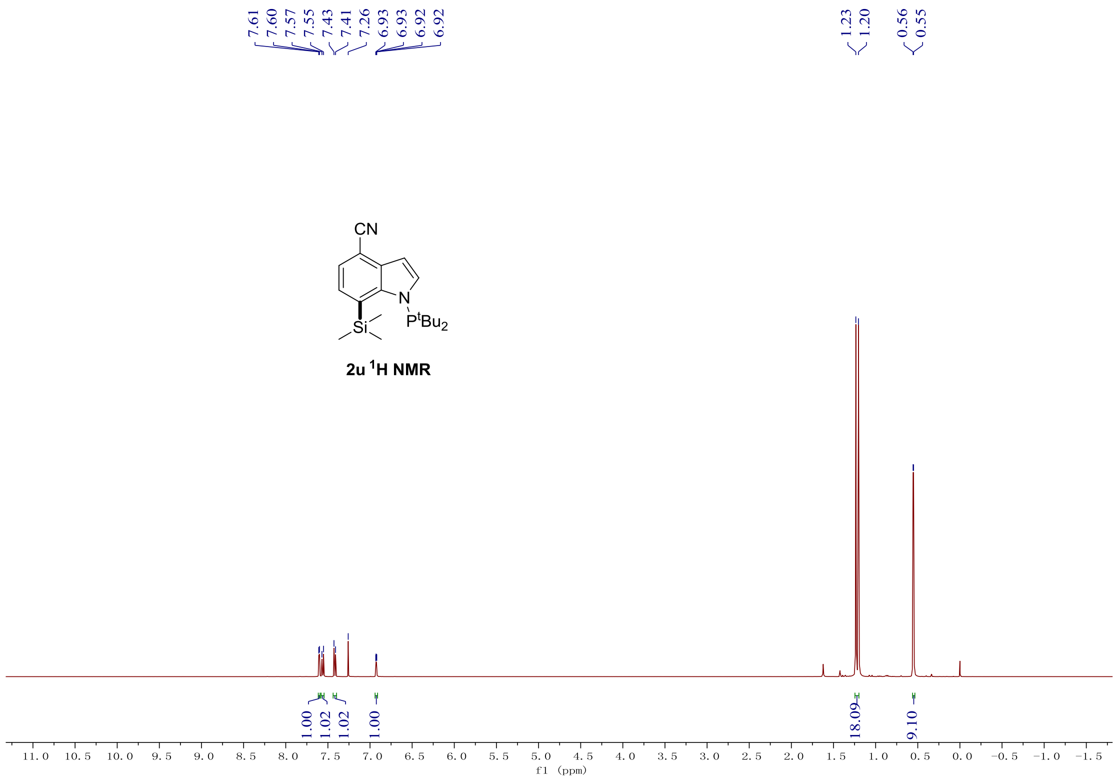

Supplementary Figure 68. ^{1}H NMR of compound 2u.

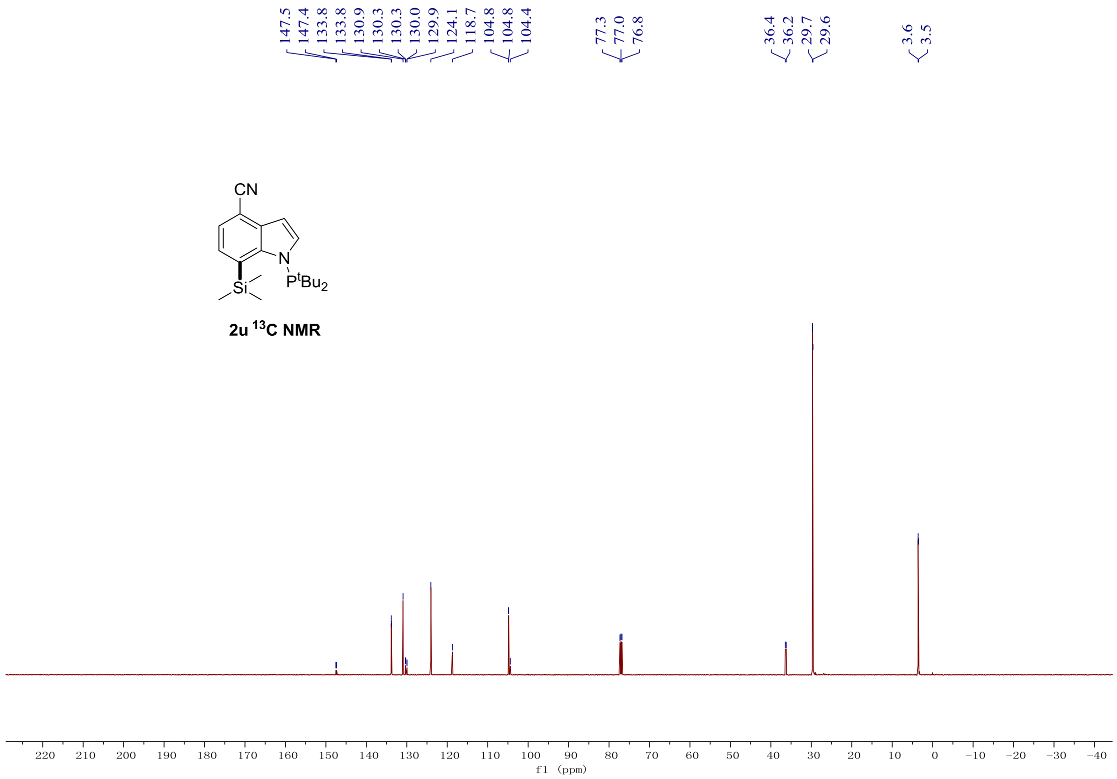

Supplementary Figure 69. ^{13}C NMR of compound 2u.

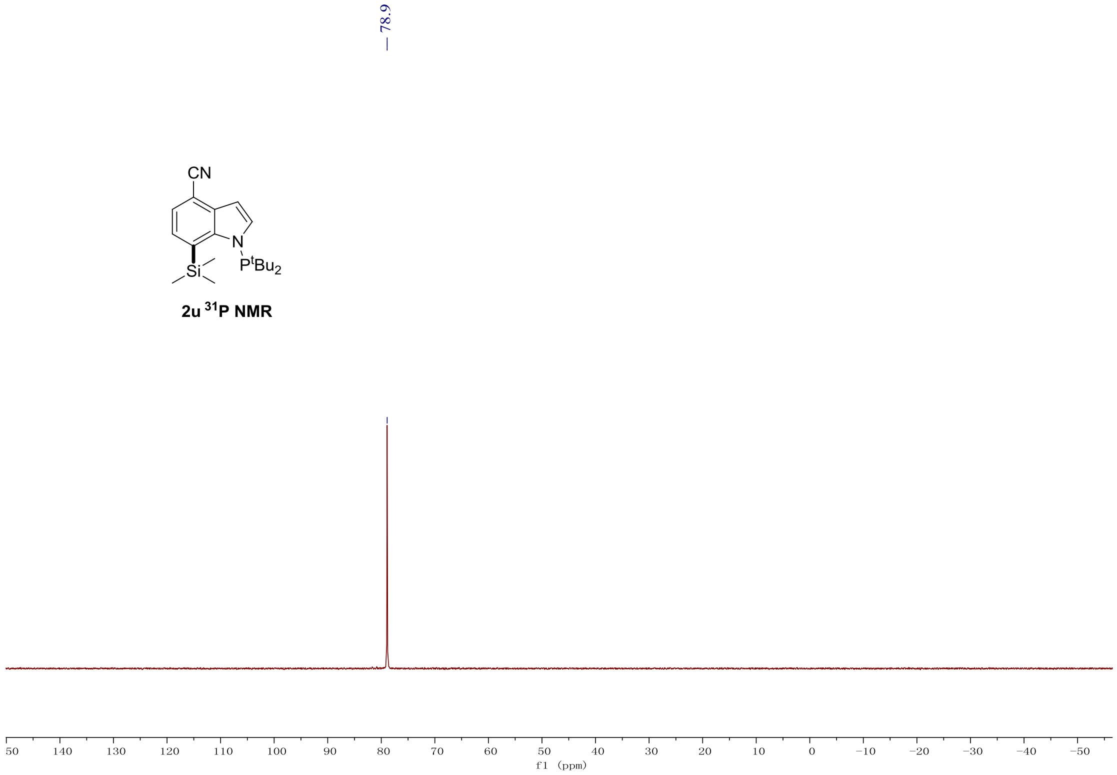

Supplementary Figure 70. ^{31}P NMR of compound 2u.

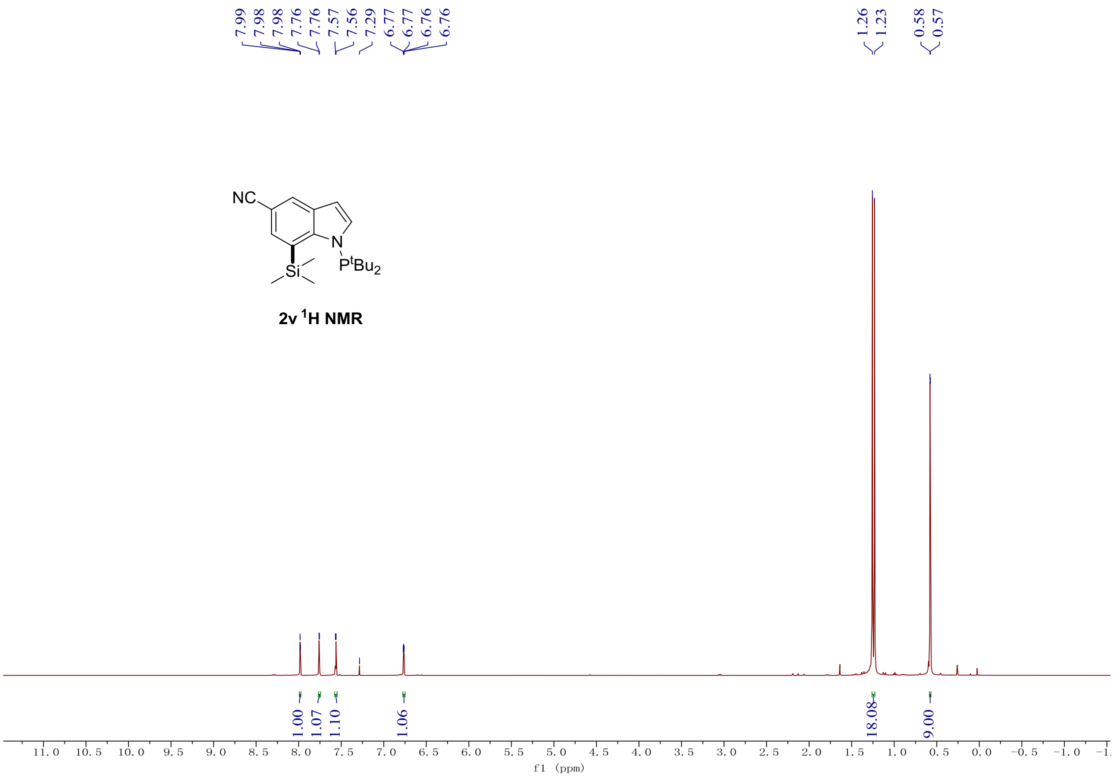

Supplementary Figure 71. ^{1}H NMR of compound 2v.

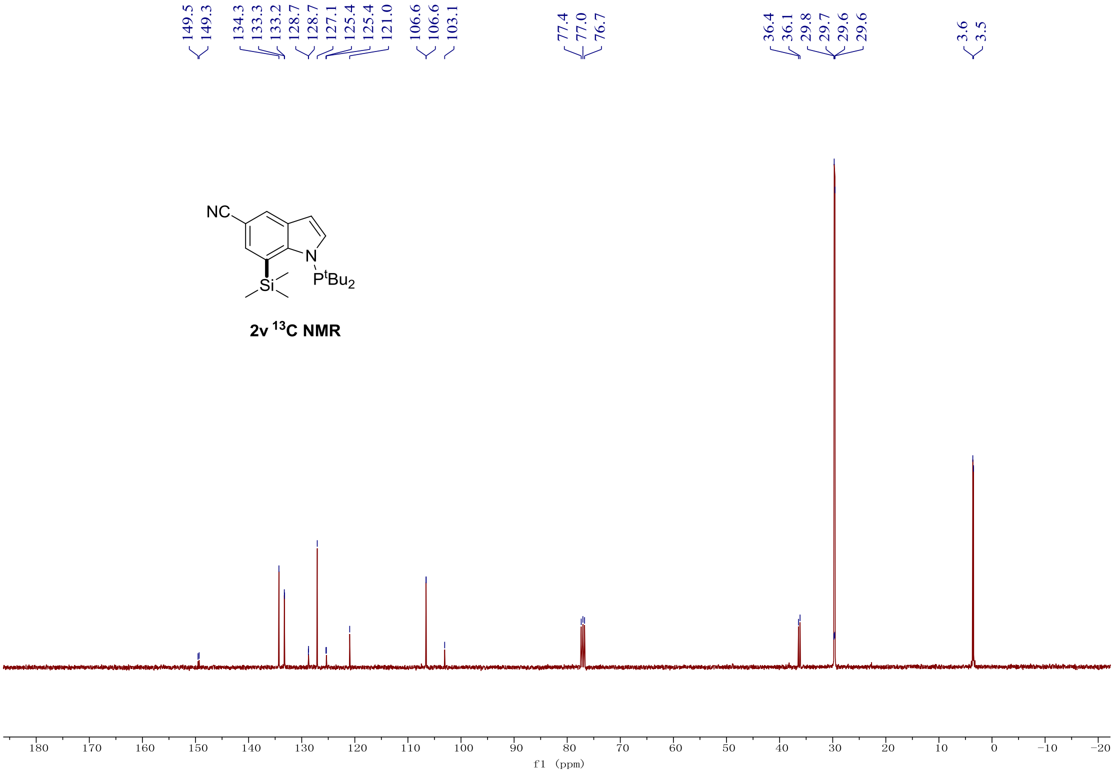

Supplementary Figure 72. ^{13}C NMR of compound 2v.

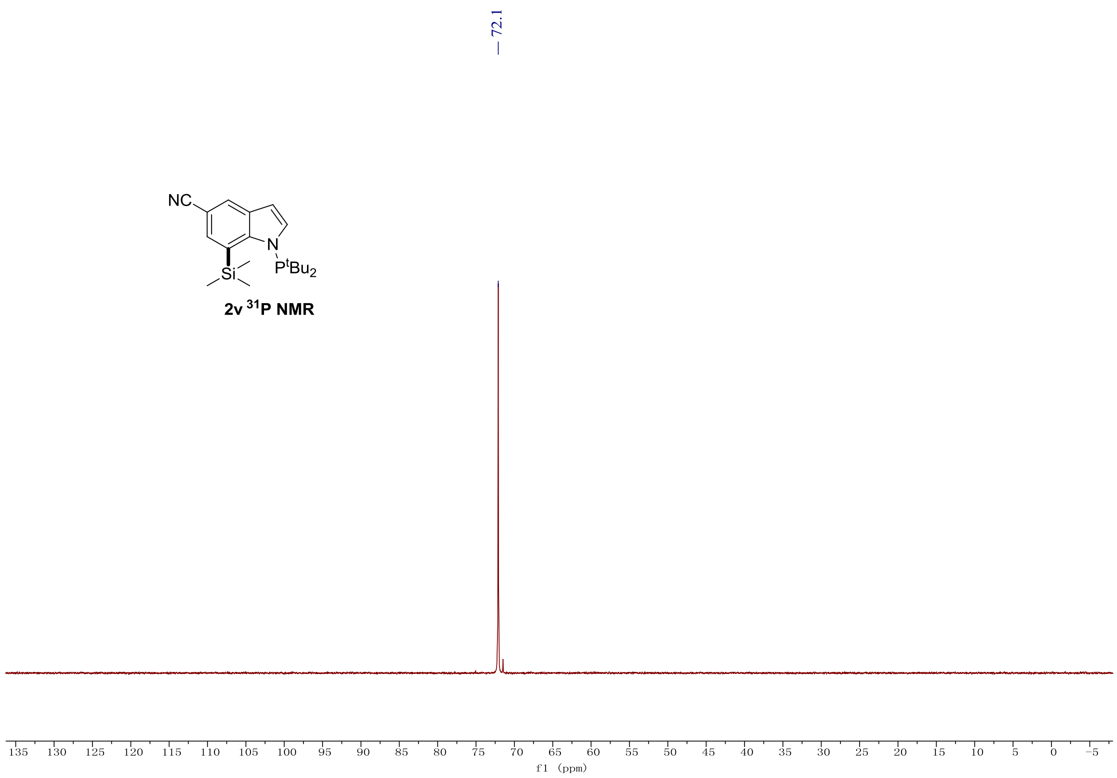

Supplementary Figure 73. ^{31}P NMR of compound 2v.

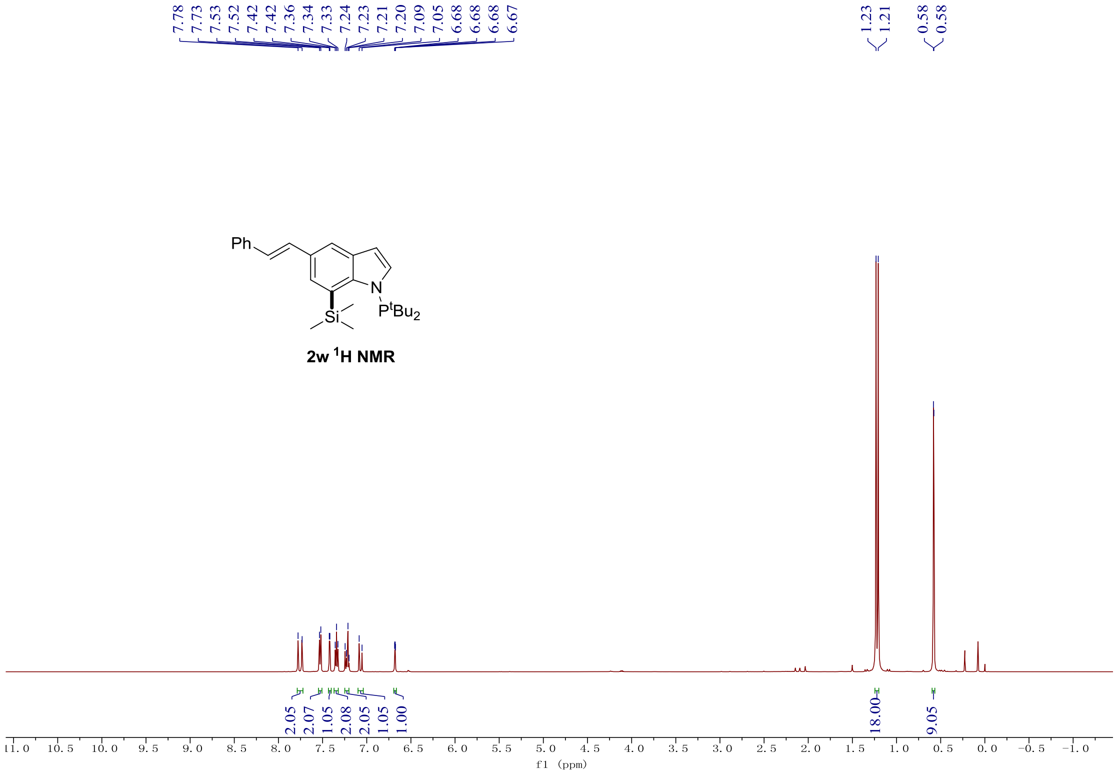

Supplementary Figure 74. ^{1}H NMR of compound 2w.

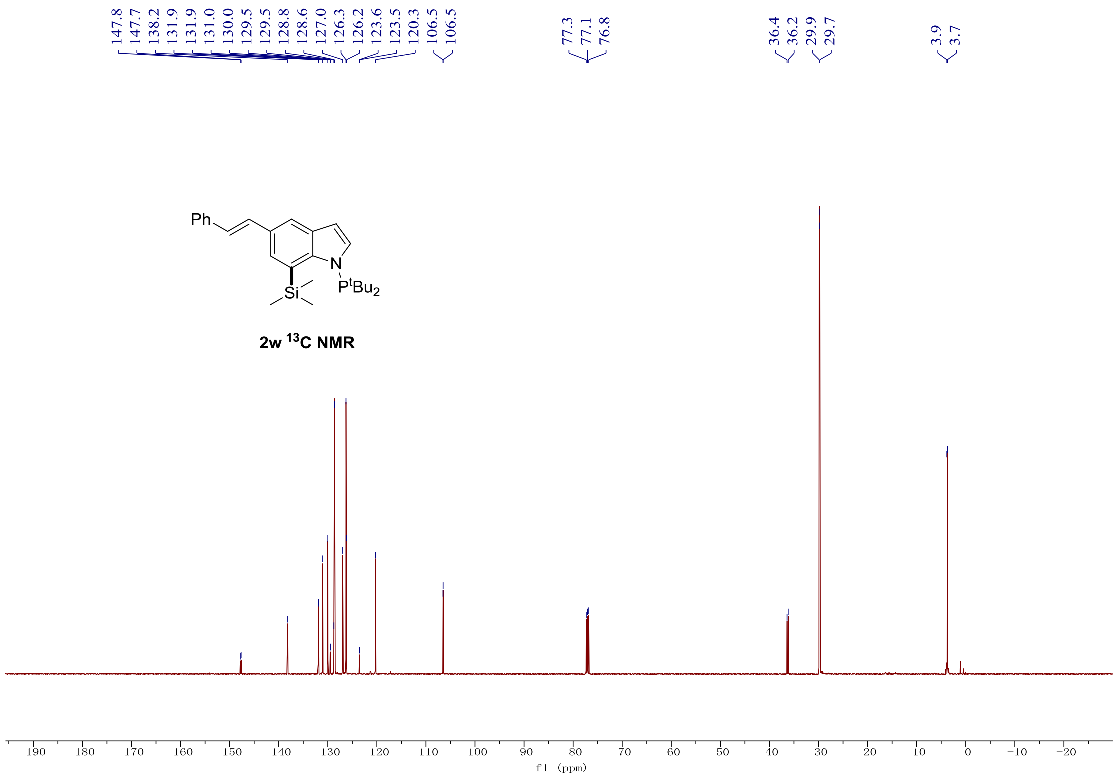

Supplementary Figure 75. ^{13}C NMR of compound 2w.

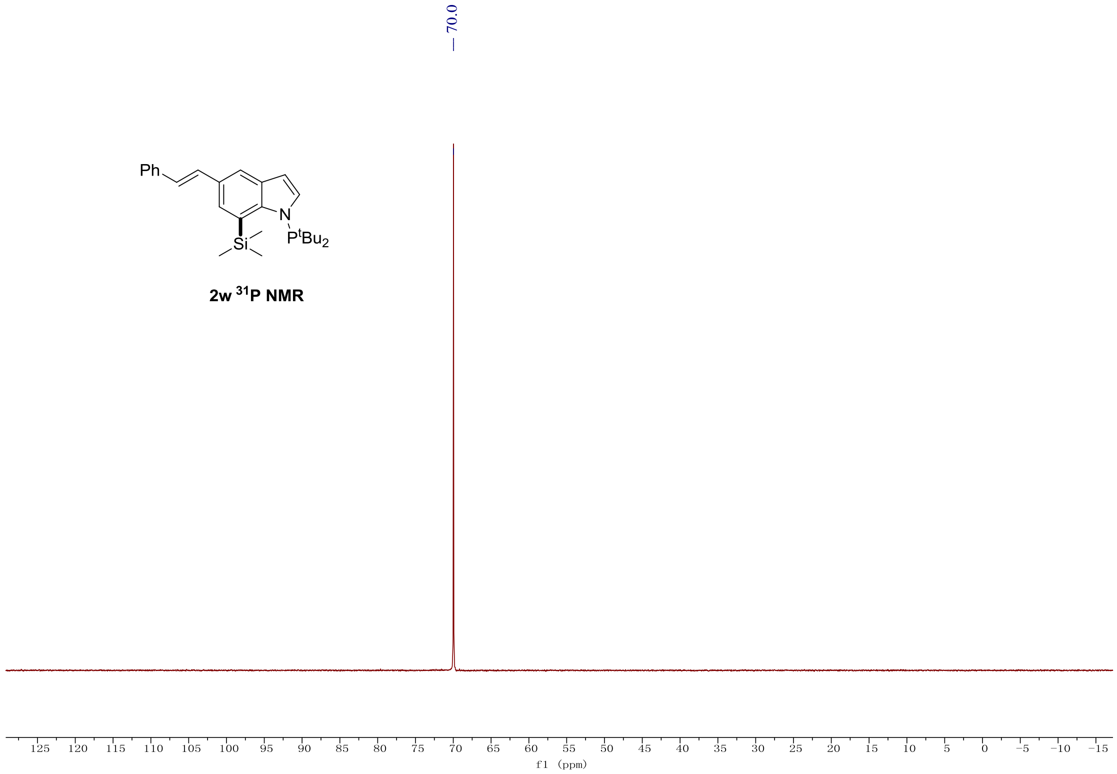

Supplementary Figure 76. ^{31}P NMR of compound 2w.

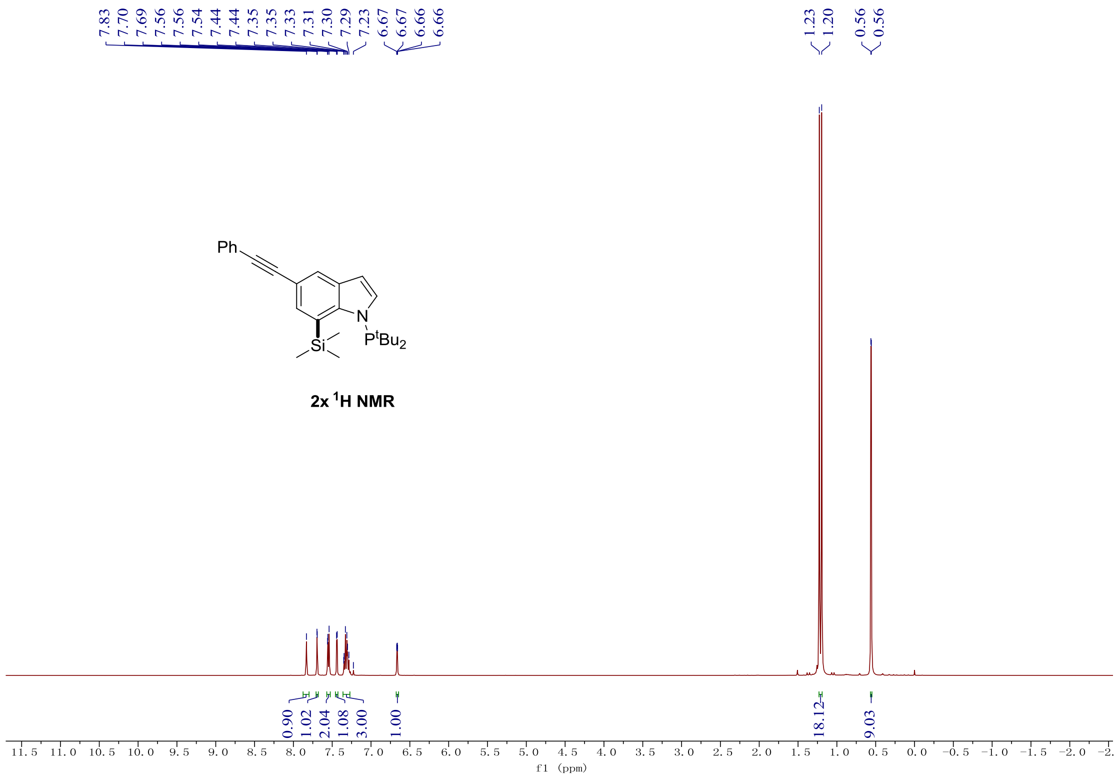

Supplementary Figure 77. ^{1}H NMR of compound 2x.

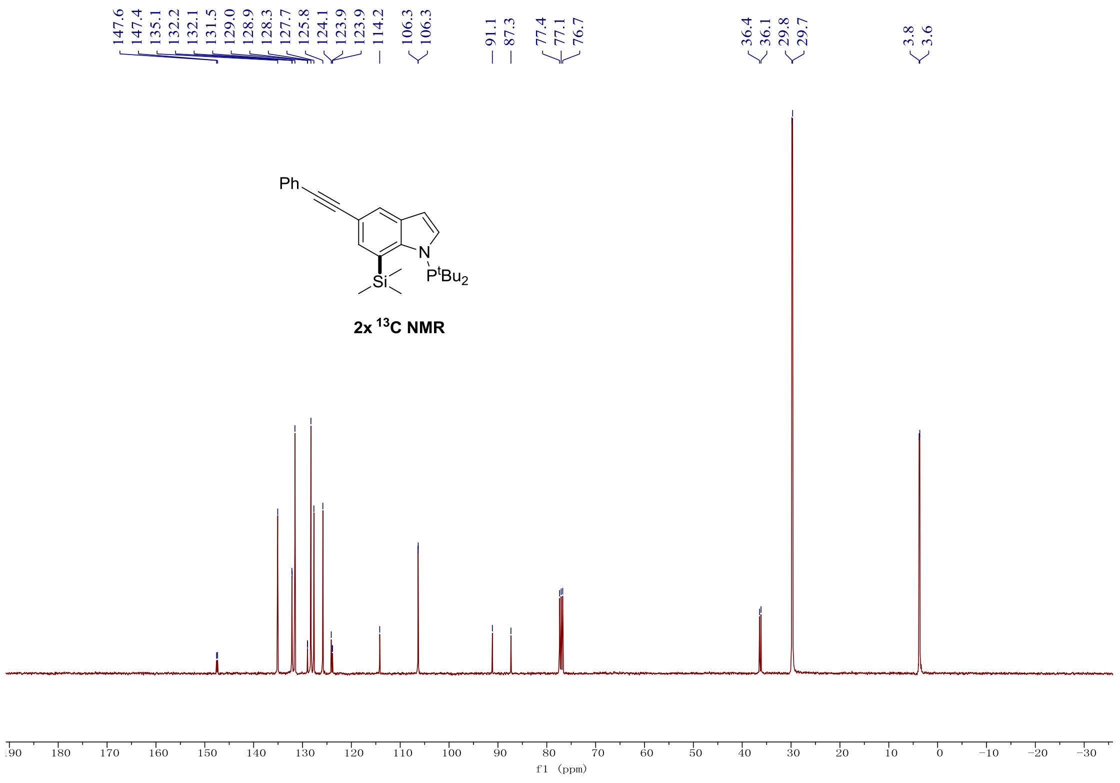

Supplementary Figure 78. ^{13}C NMR of compound 2x.

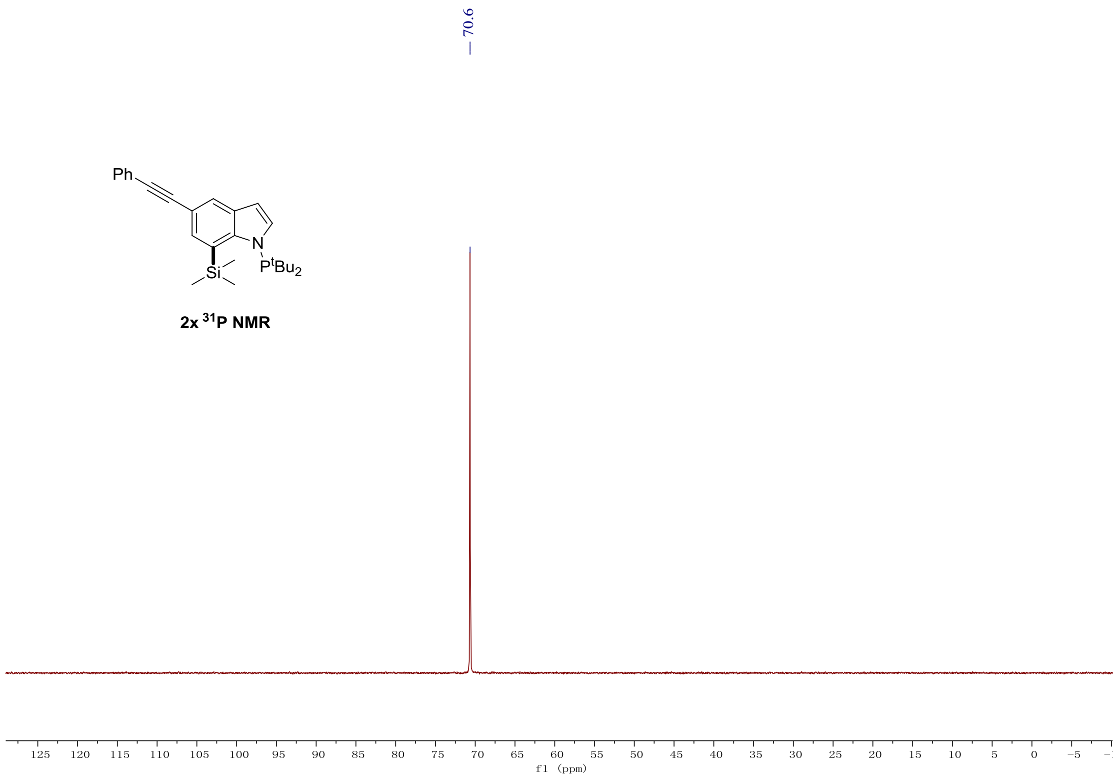

Supplementary Figure 79. ^{31}P NMR of compound 2x.

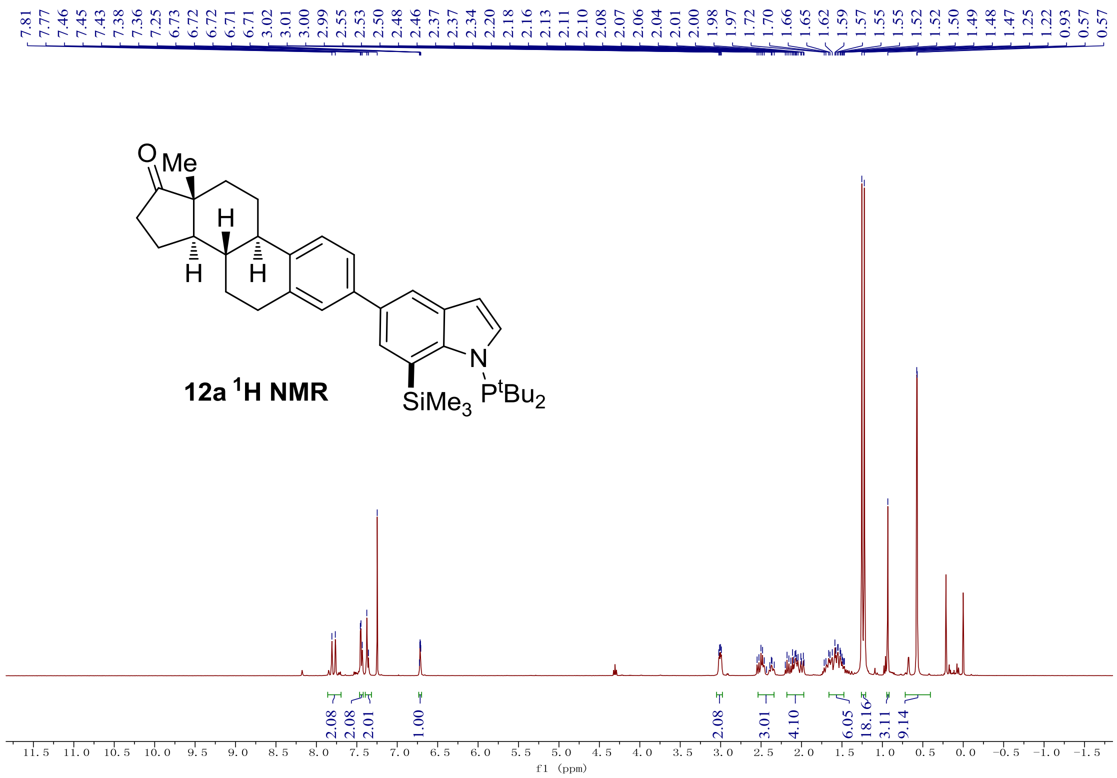

Supplementary Figure 80. ^{1}H NMR of compound 12a.

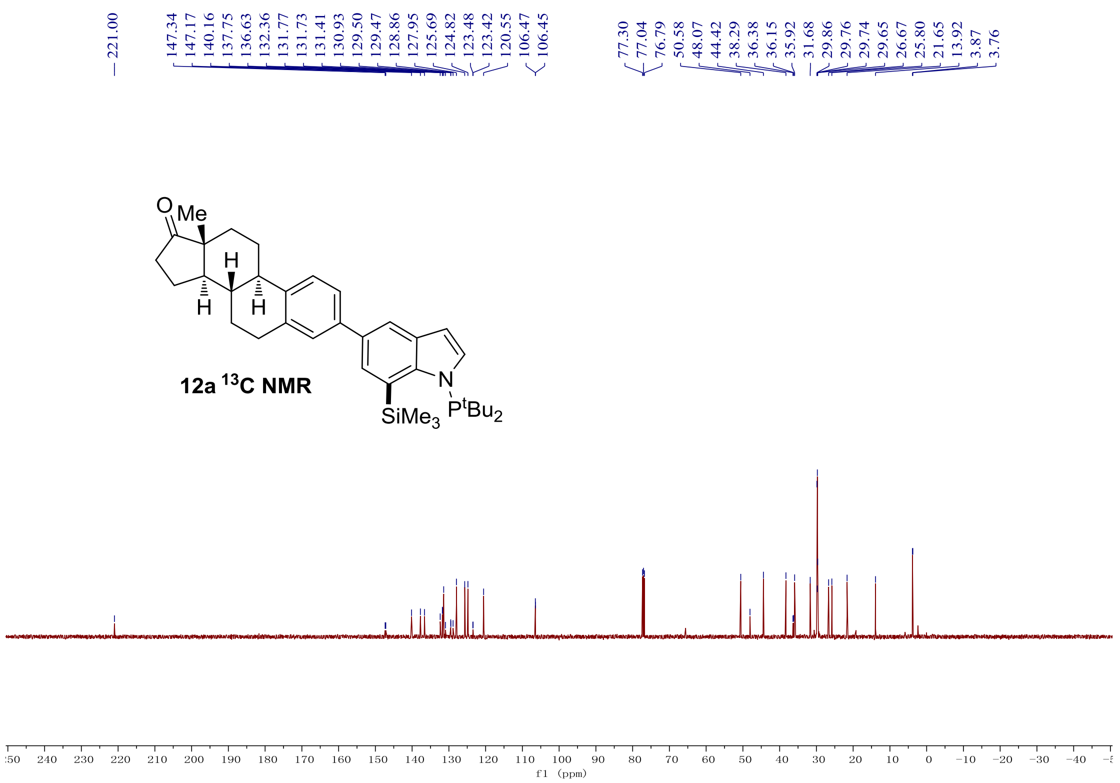

Supplementary Figure 81. ^{13}C NMR of compound 12a.

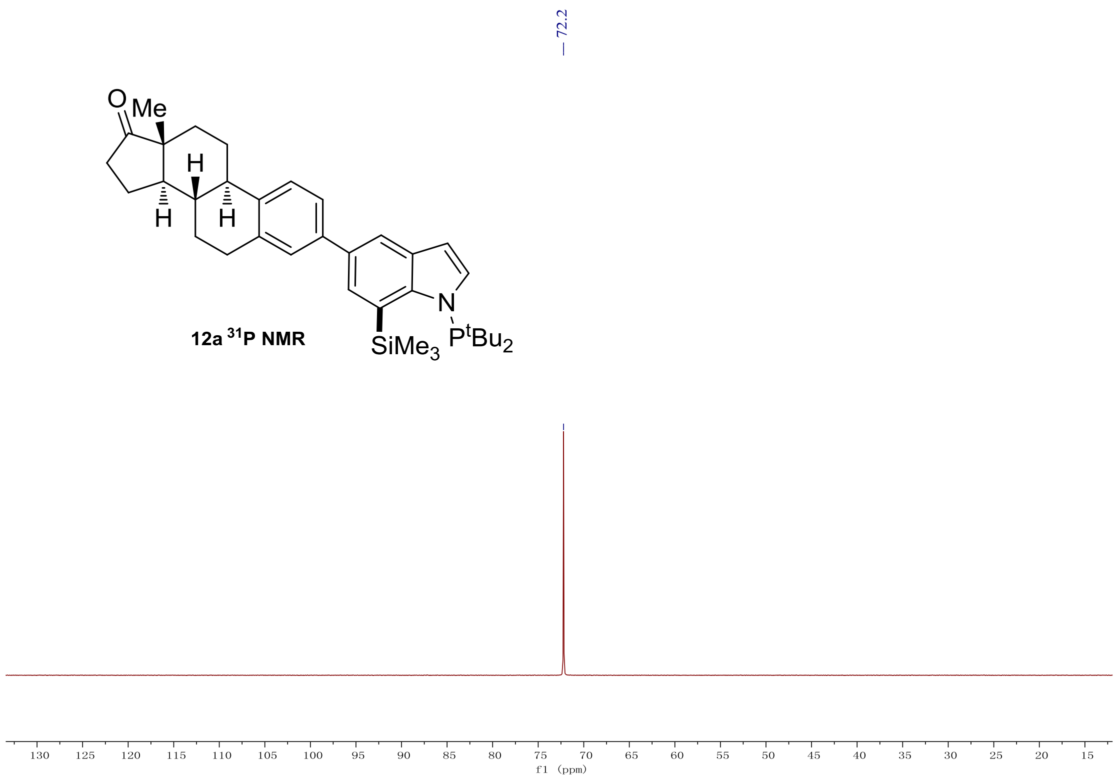

Supplementary Figure 82. ^{31}P NMR of compound 12a.

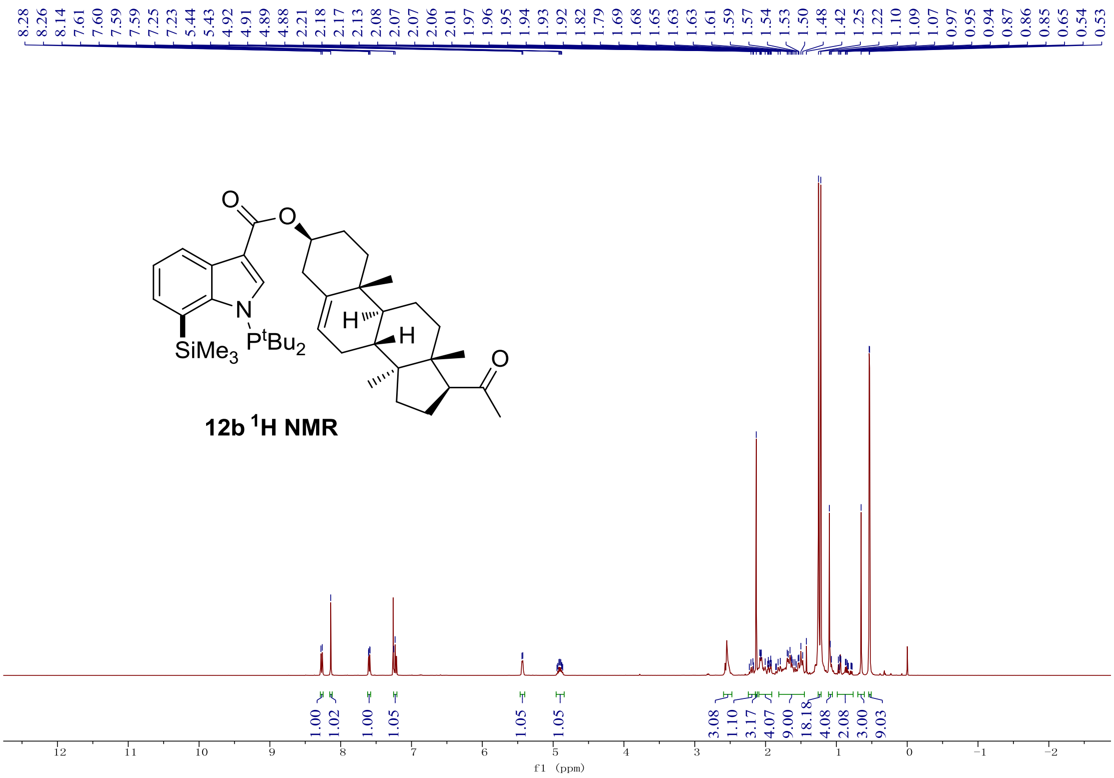

Supplementary Figure 83. ^{1}H NMR of compound 12b.

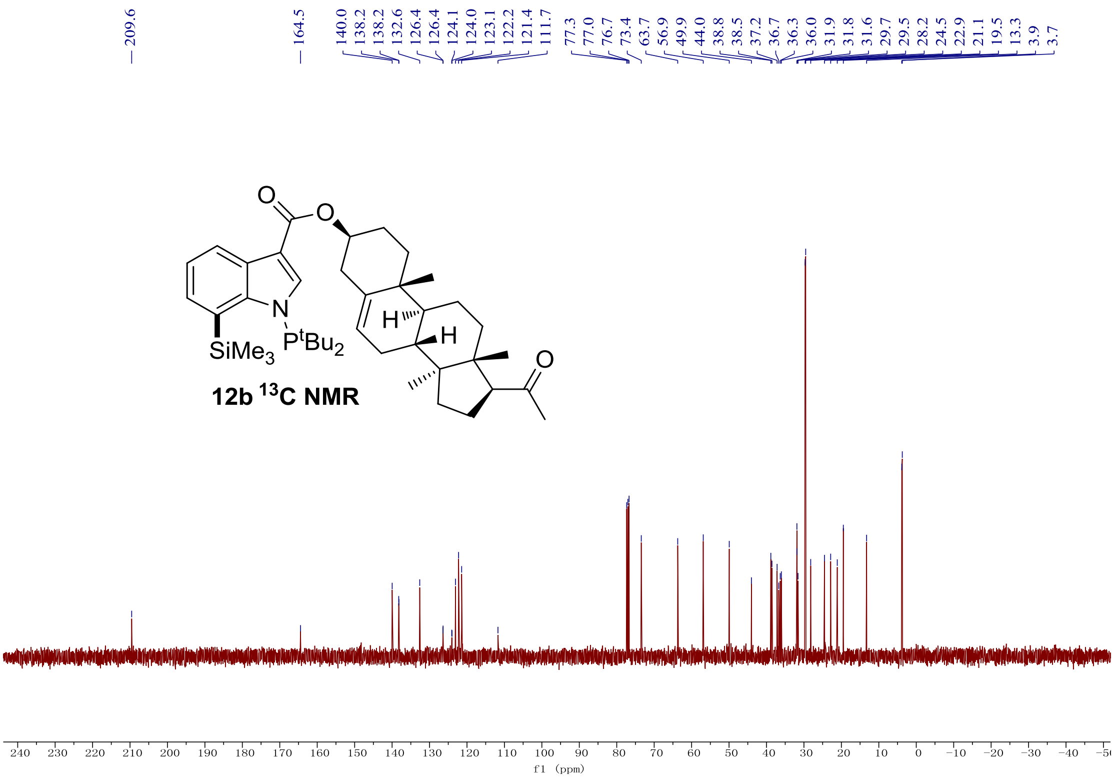

Supplementary Figure 84. ^{13}C NMR of compound 12b.

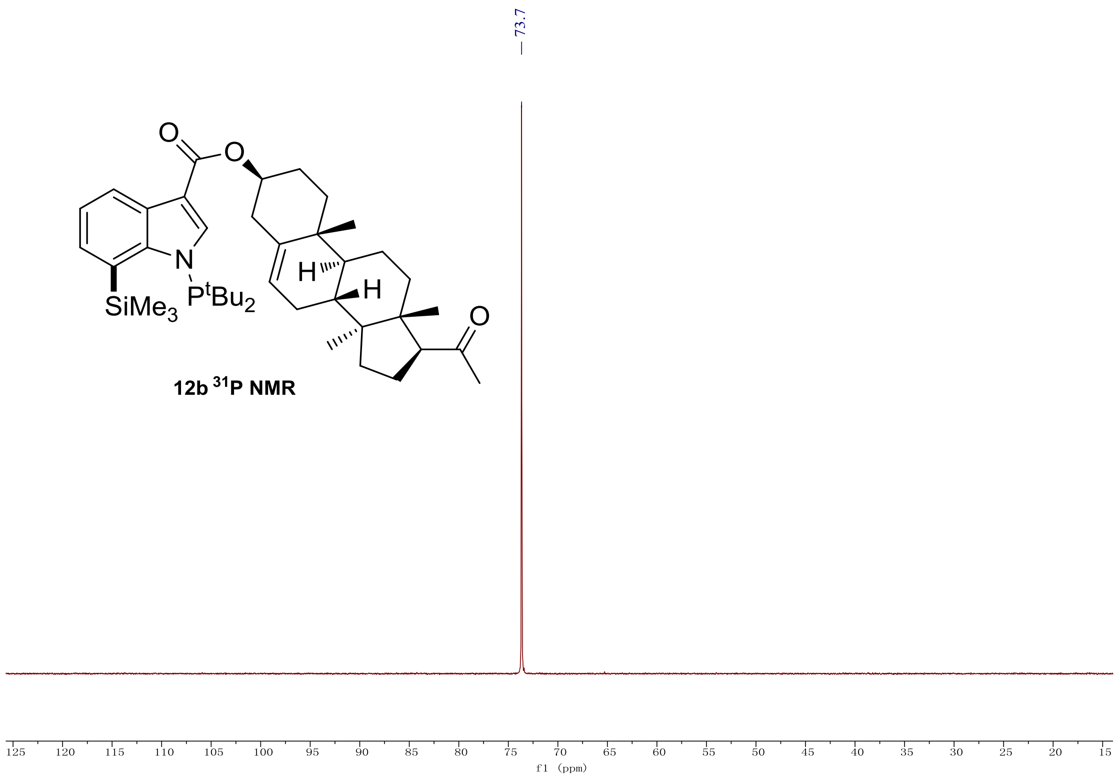

Supplementary Figure 85. ^{31}P NMR of compound 12b.

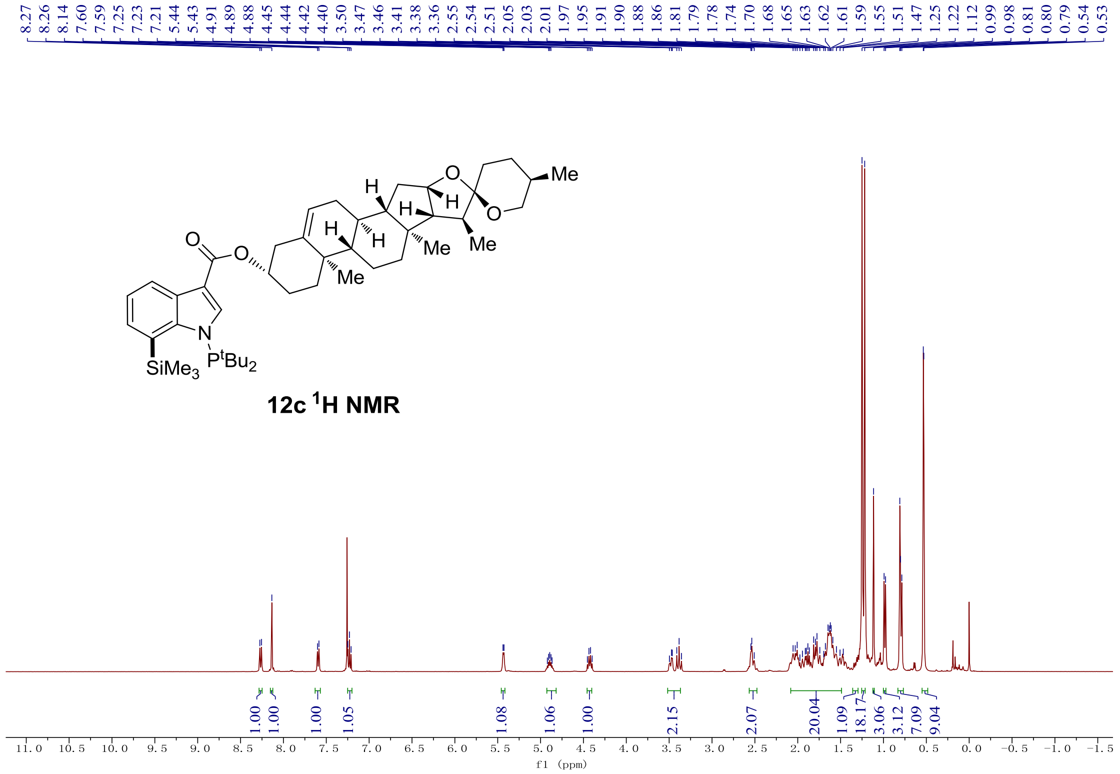

Supplementary Figure 86. ^{1}H NMR of compound 12c.

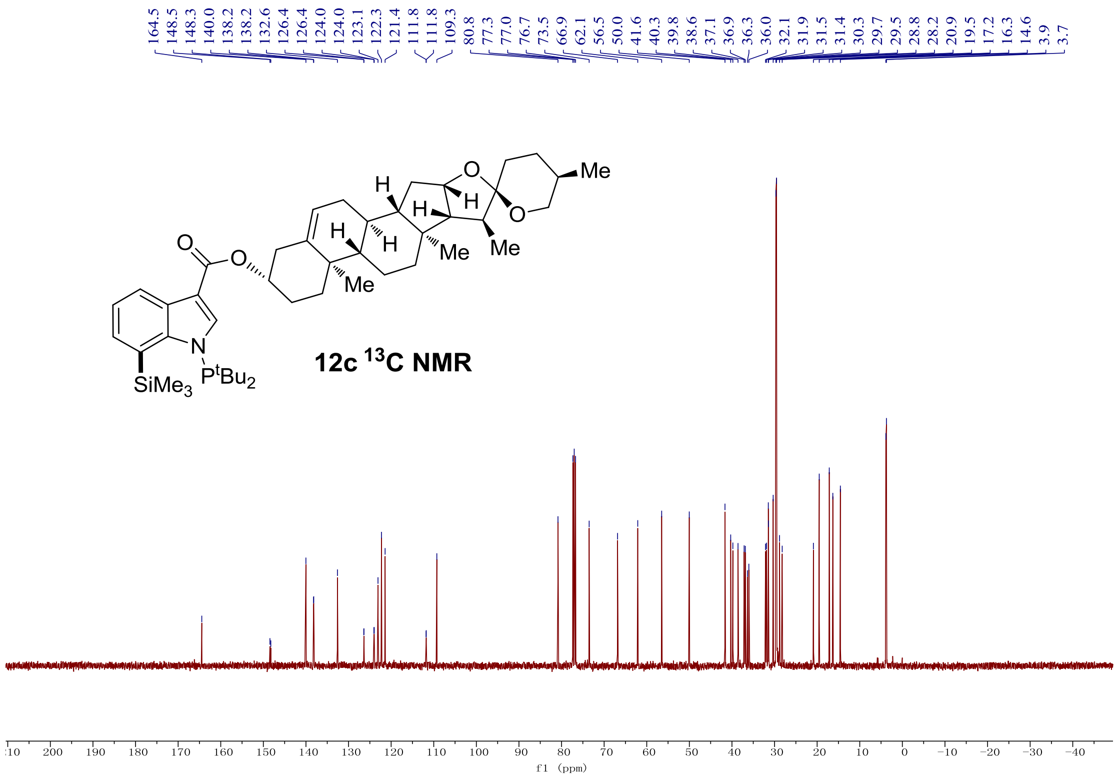

Supplementary Figure 87. ^{13}C NMR of compound 12c.

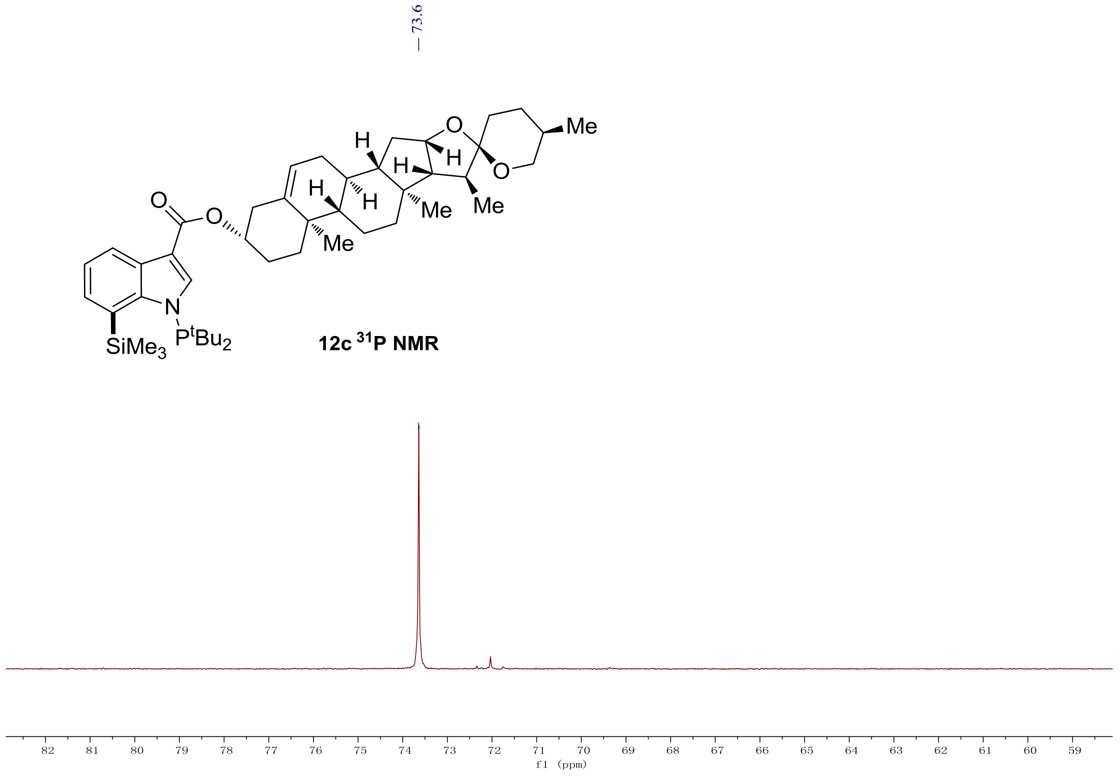

Supplementary Figure 88. ^{31}P NMR of compound 12c.

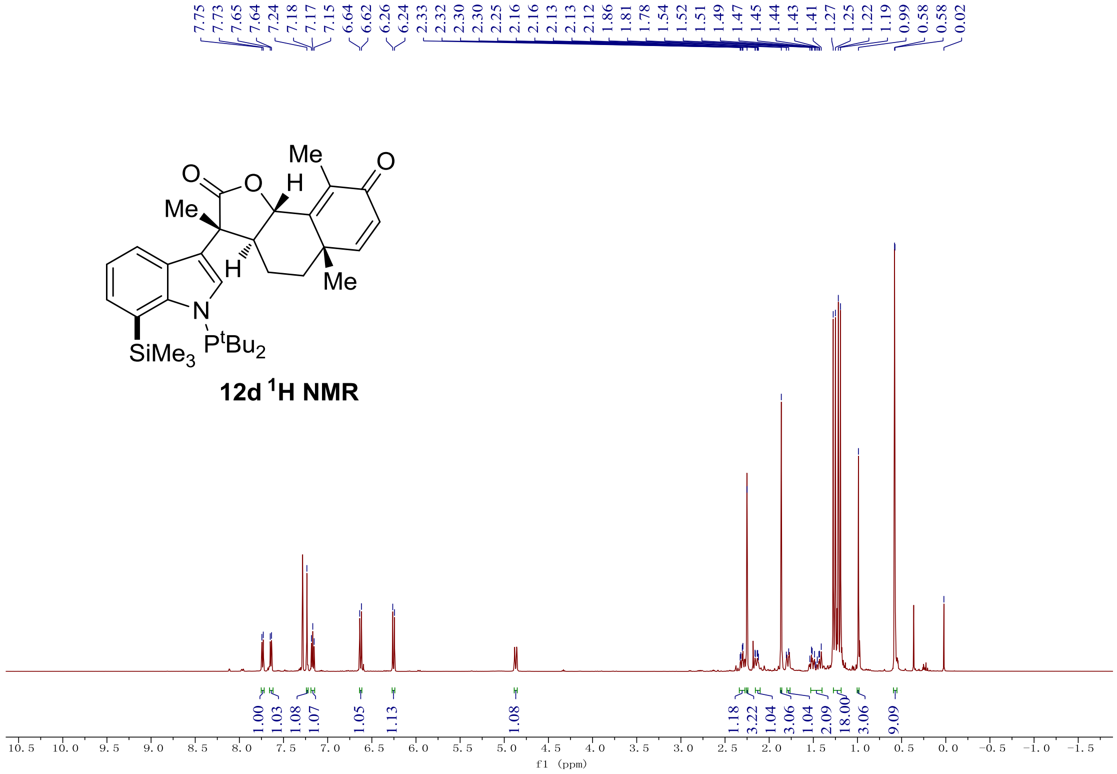

Supplementary Figure 89. ^{1}**H NMR of compound 12d.**

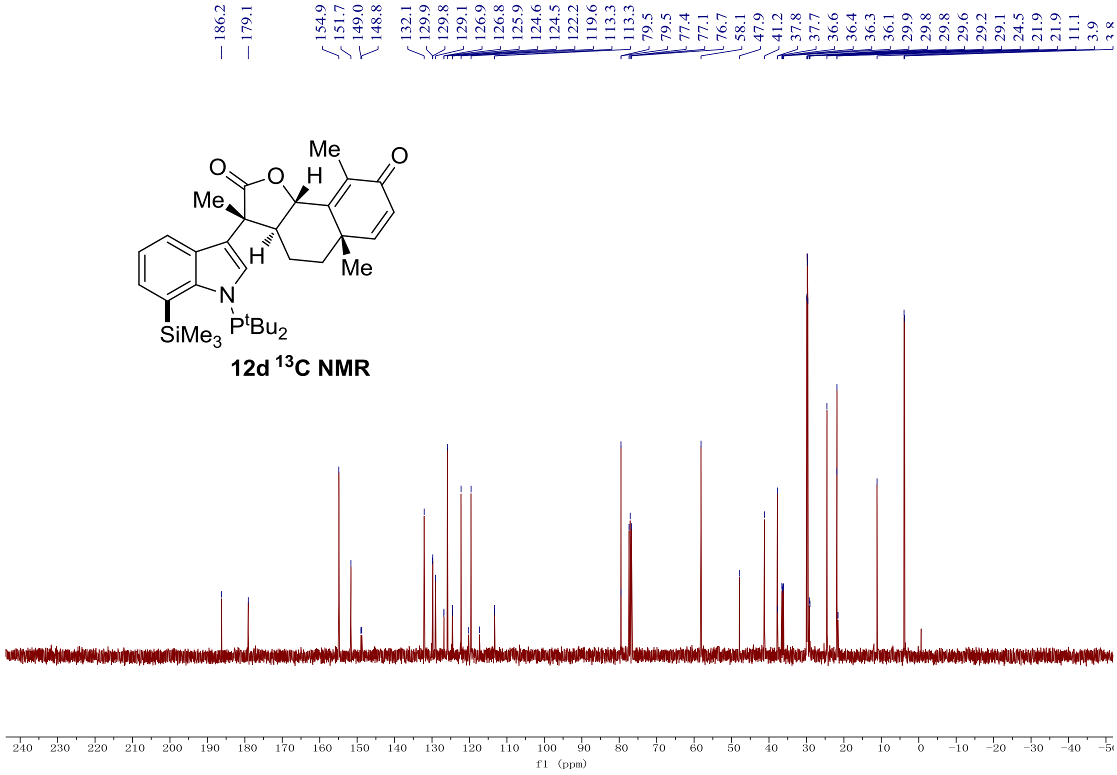

Supplementary Figure 90. ^{13}**C NMR of compound 12d.**

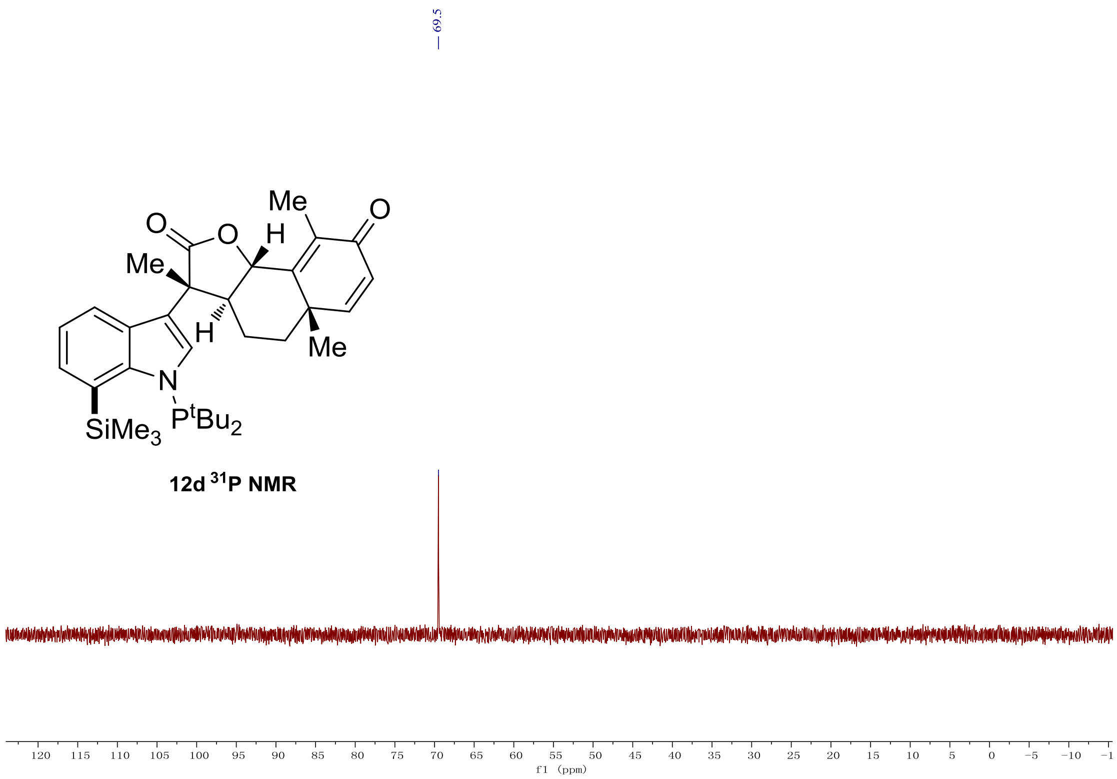

Supplementary Figure 91. ^{31}P NMR of compound 12d.

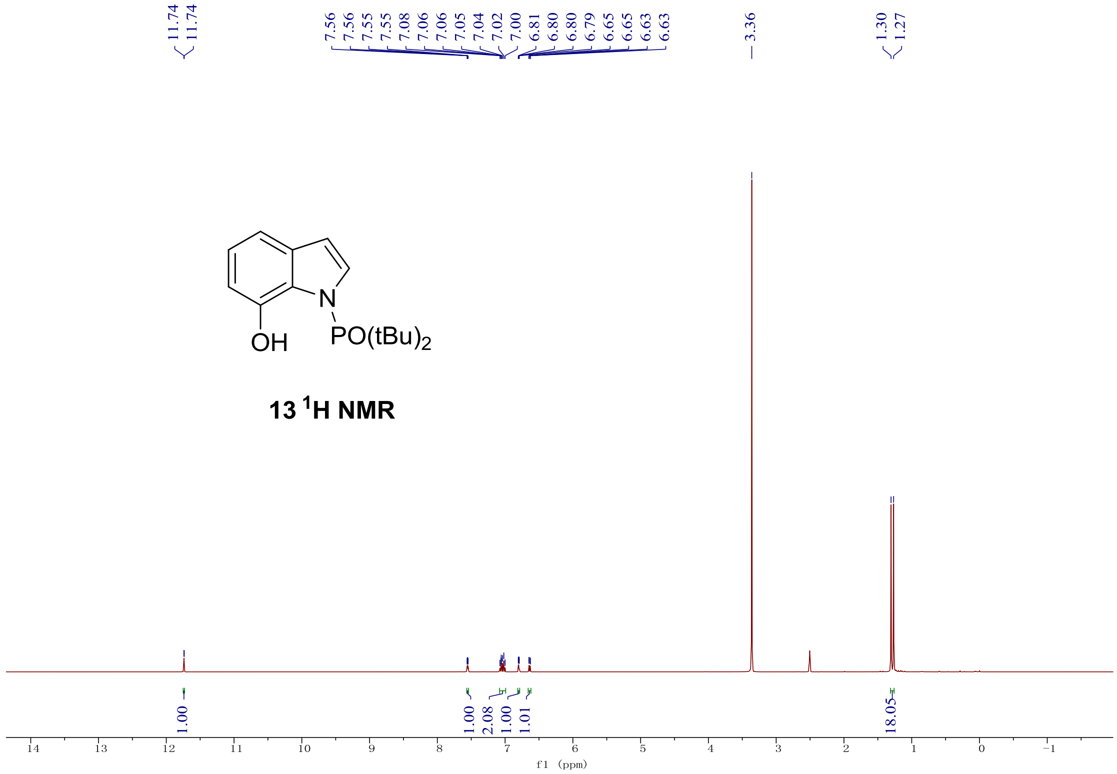

Supplementary Figure 92. ^{1}H NMR of compound 13.

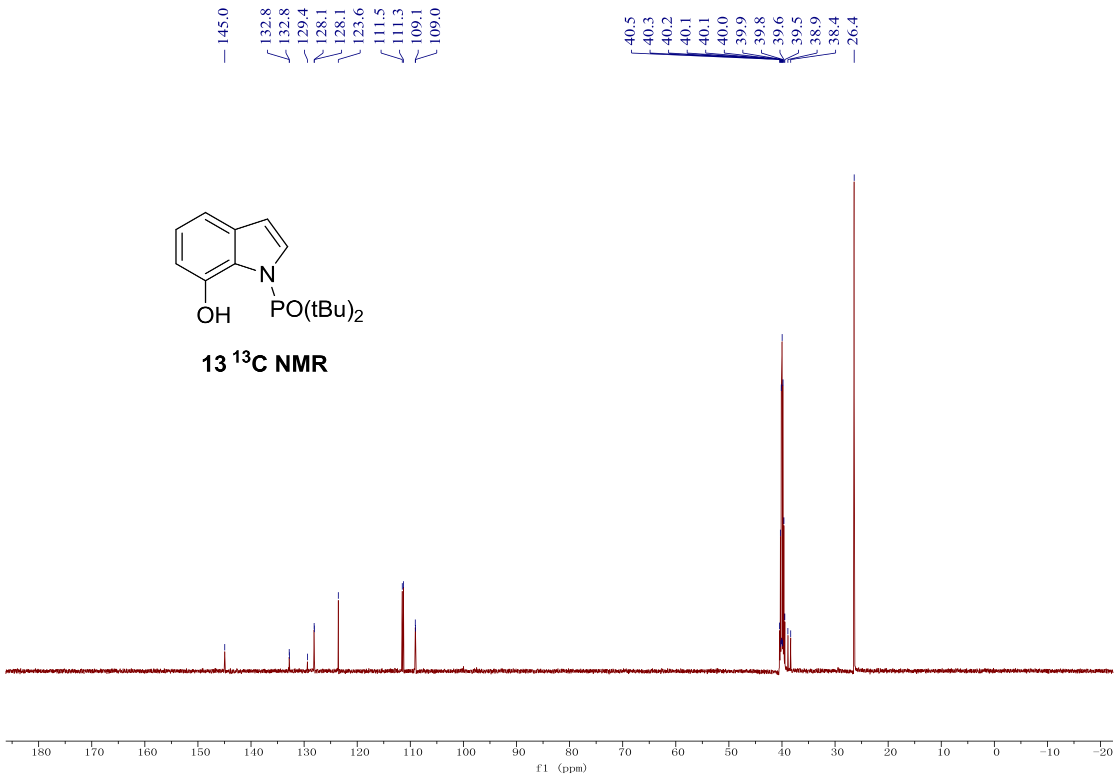

Supplementary Figure 93. ^{13}C NMR of compound 13.

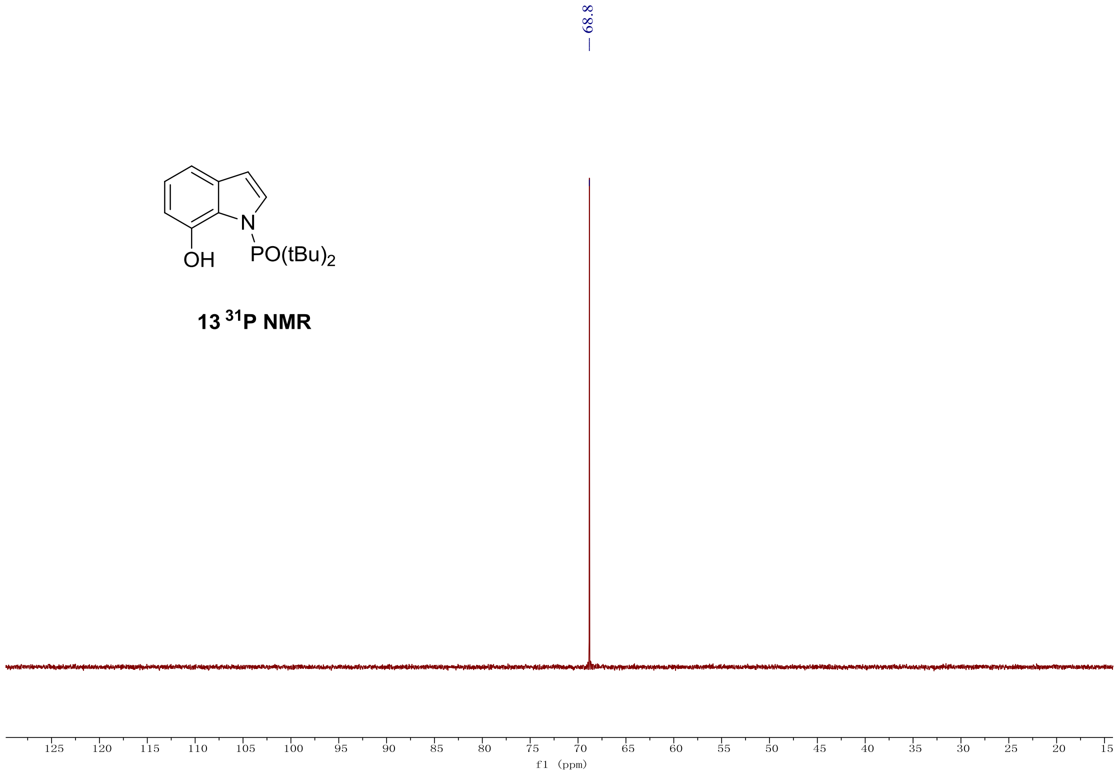

Supplementary Figure 94. ^{31}P NMR of compound 13.

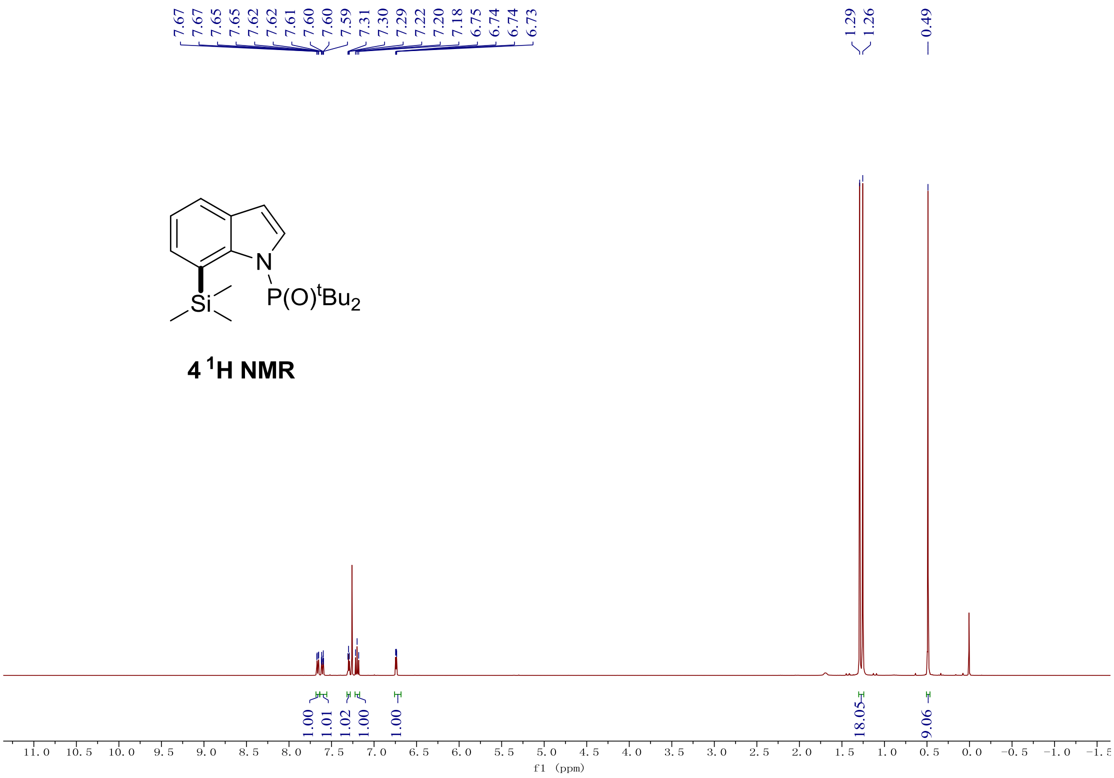

Supplementary Figure 95. ^{1}H NMR of compound 4.

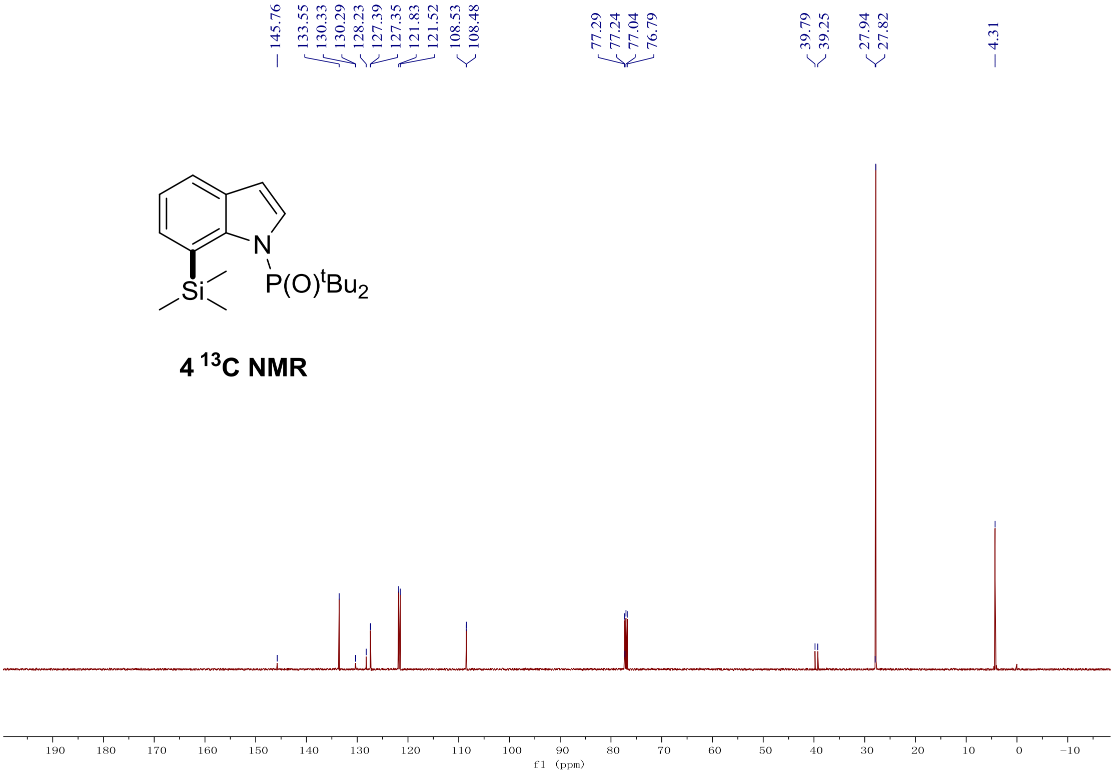

Supplementary Figure 96. ^{13}C NMR of compound 4.

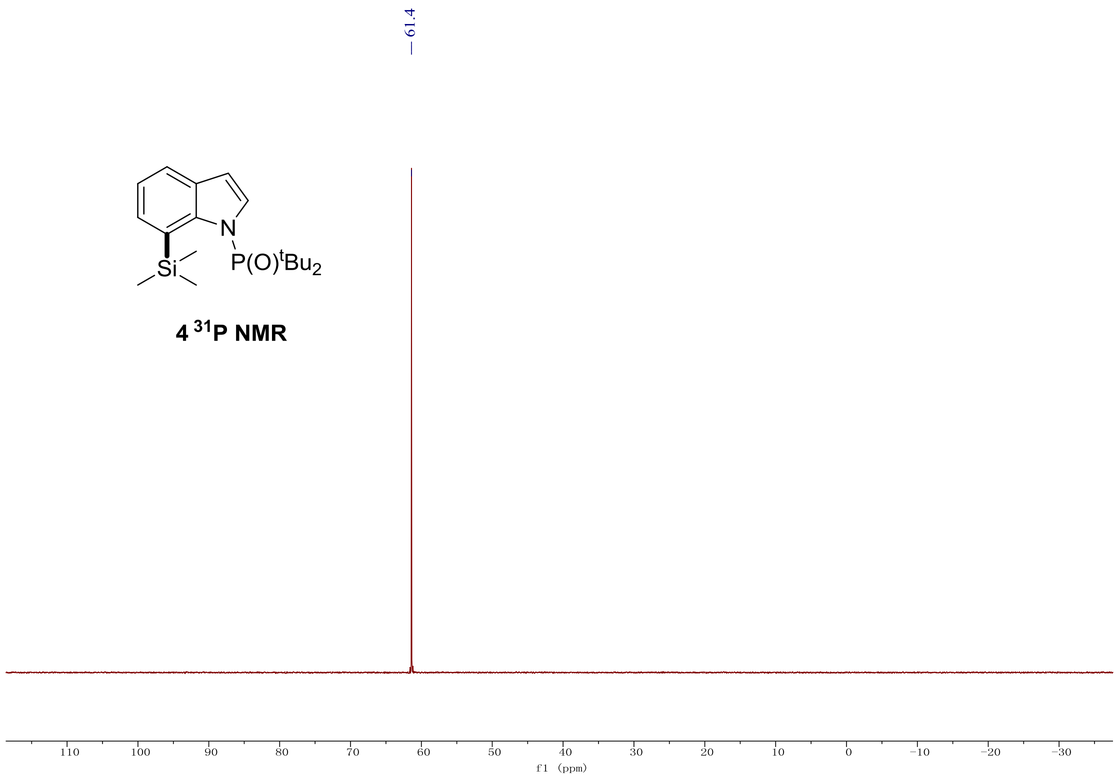

Supplementary Figure 97. ^{31}P NMR of compound 4.

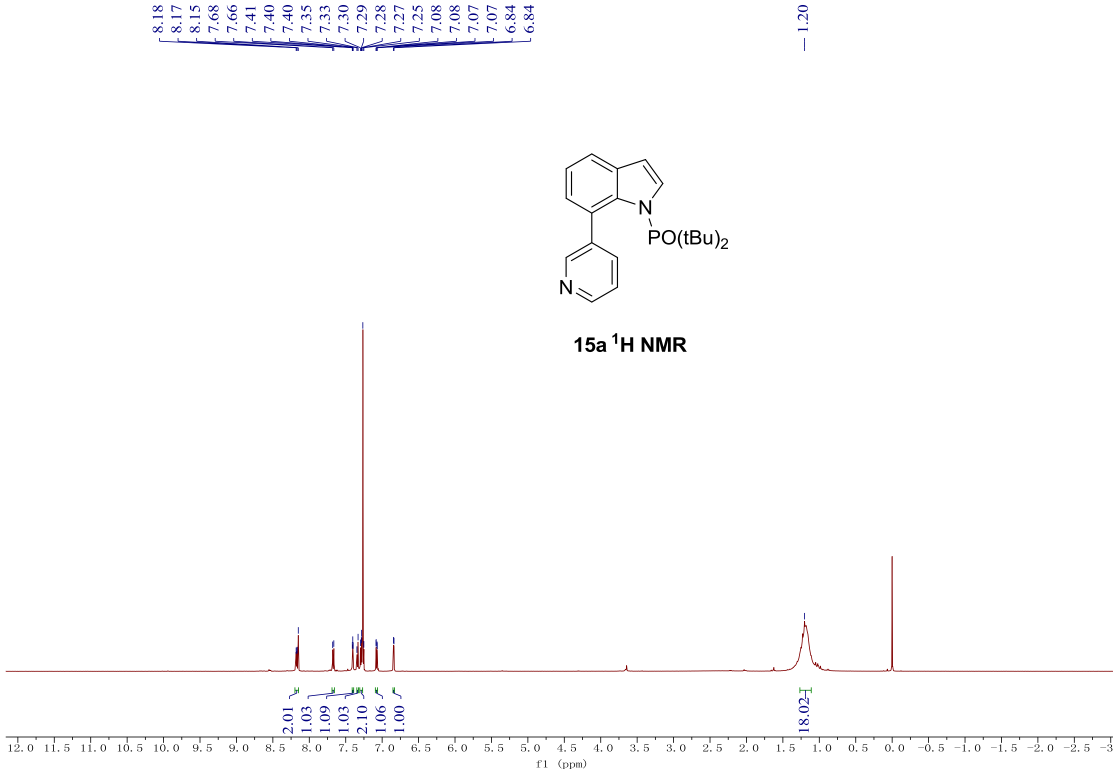

Supplementary Figure 98. ^{1}H NMR of compound 15a.

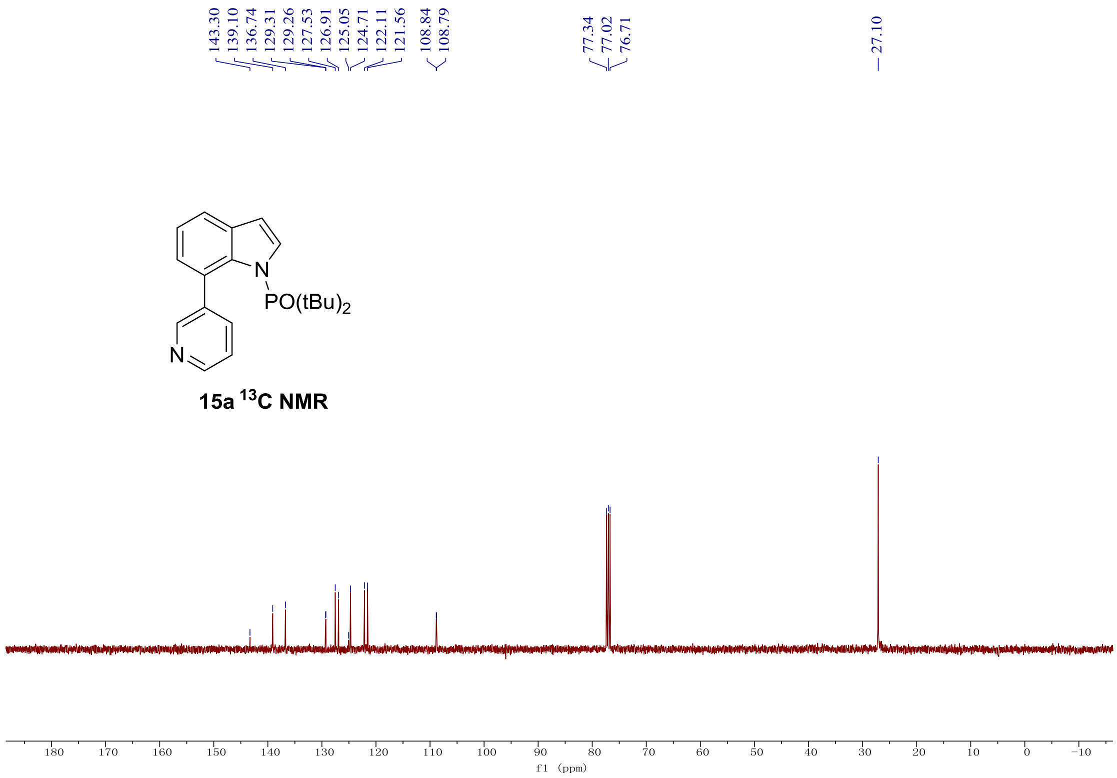

Supplementary Figure 99. ^{13}C NMR of compound 15a.

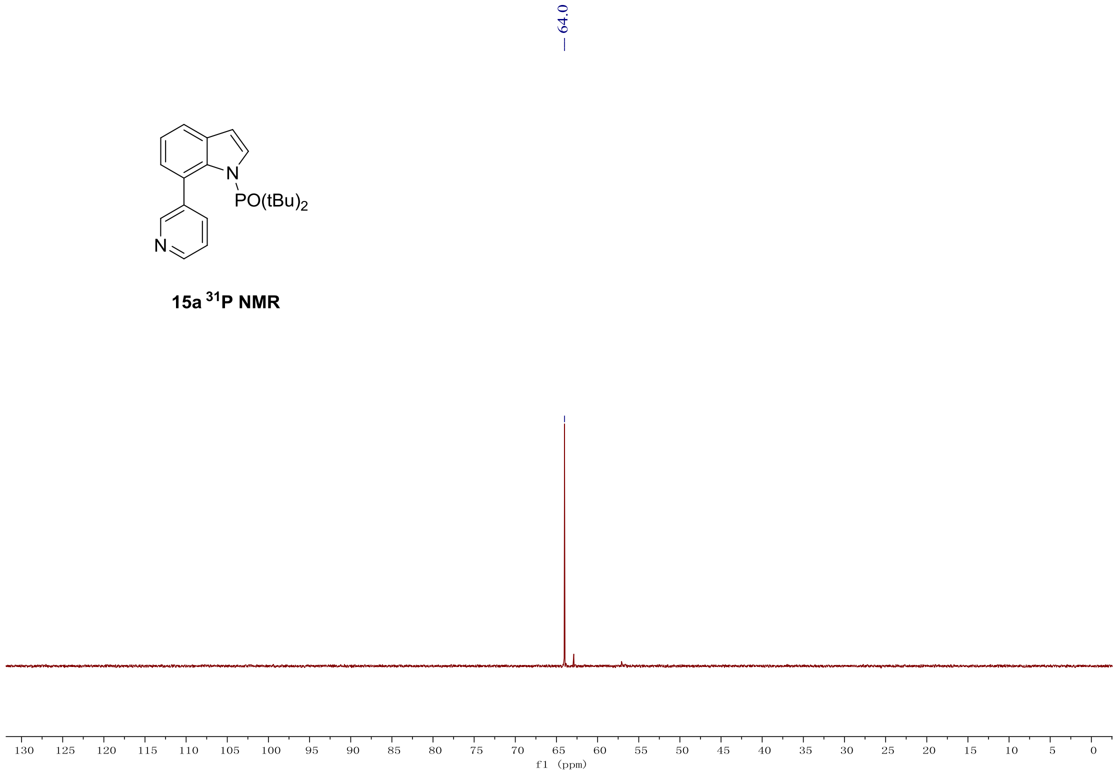

Supplementary Figure 100. ^{31}P NMR of compound 15a.

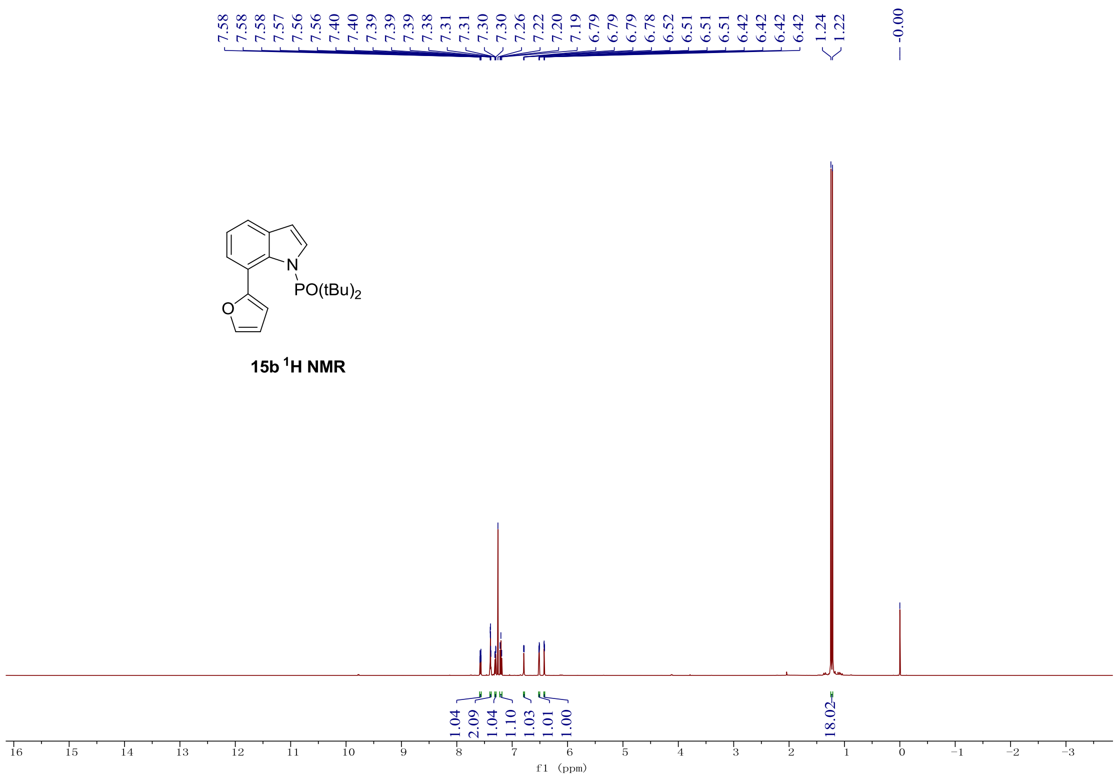

Supplementary Figure 101. ^{1}H NMR of compound 15b.

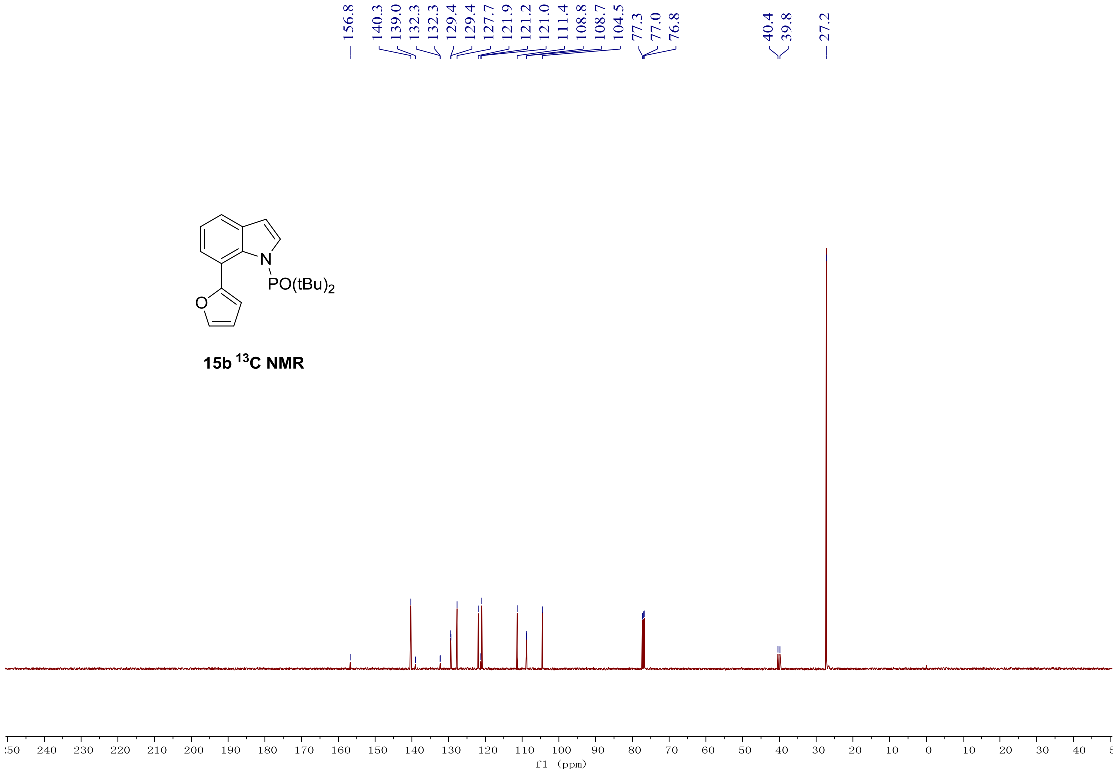

Supplementary Figure 102. ^{13}C NMR of compound 15b.

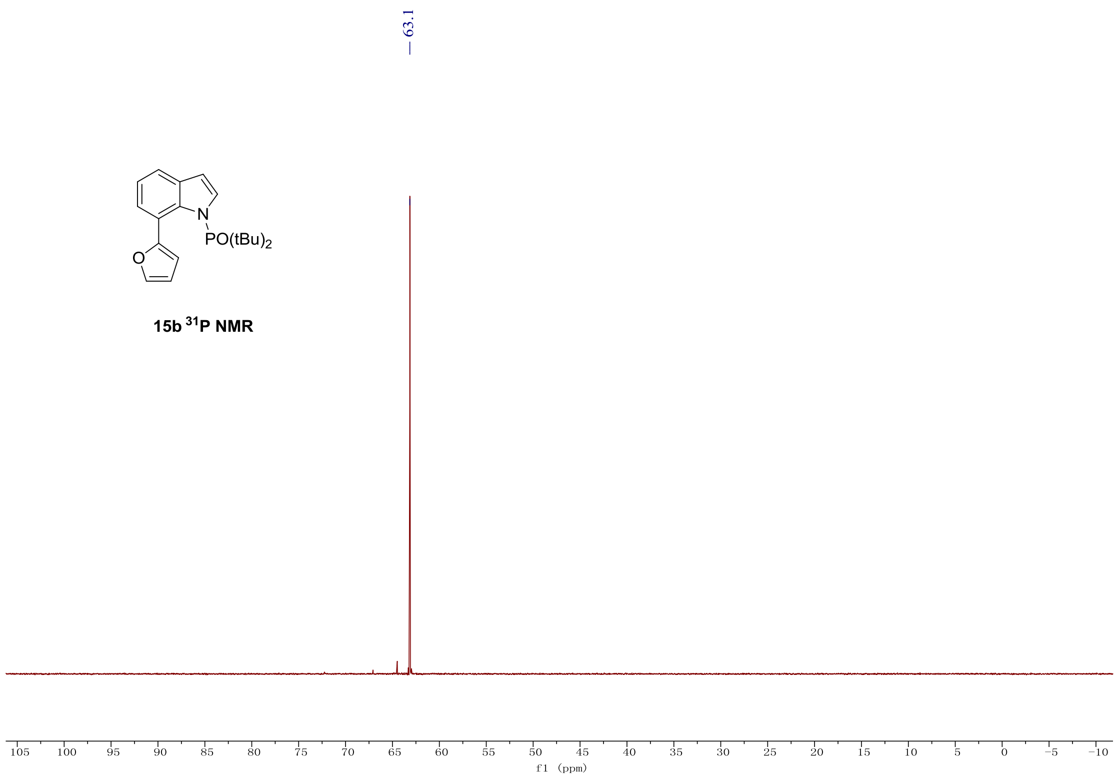

Supplementary Figure 103. ^{31}P NMR of compound 15b.

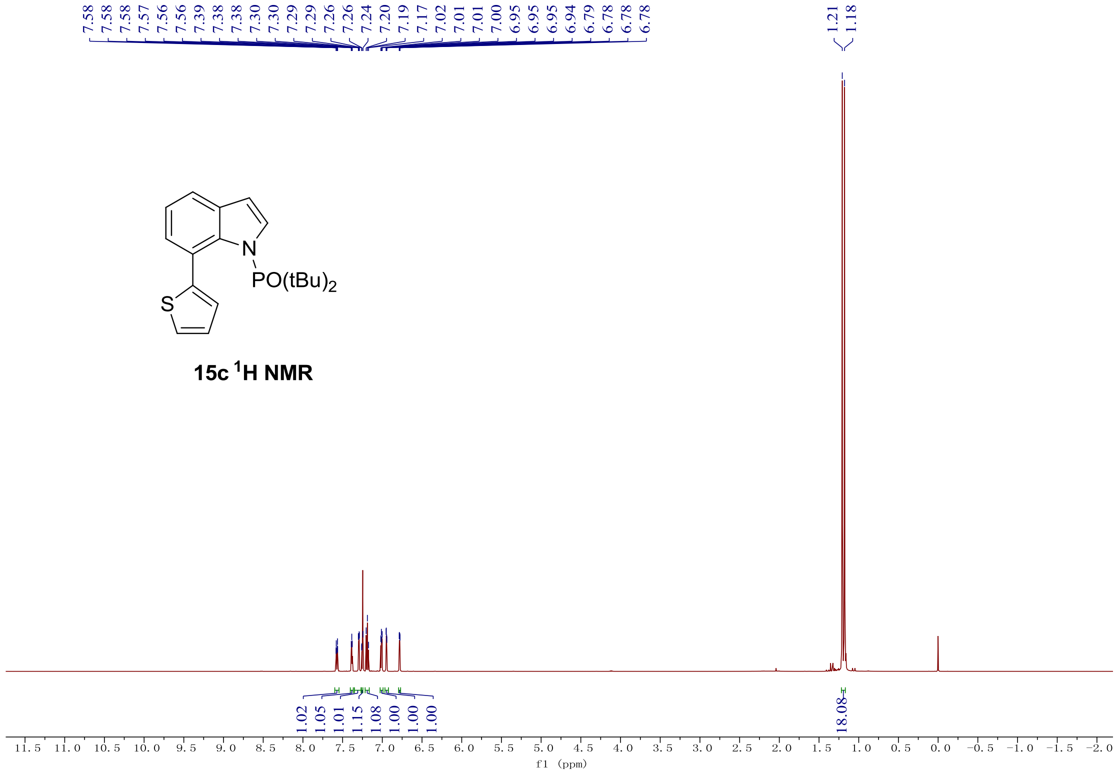

Supplementary Figure 104. ^{1}H NMR of compound 15c.

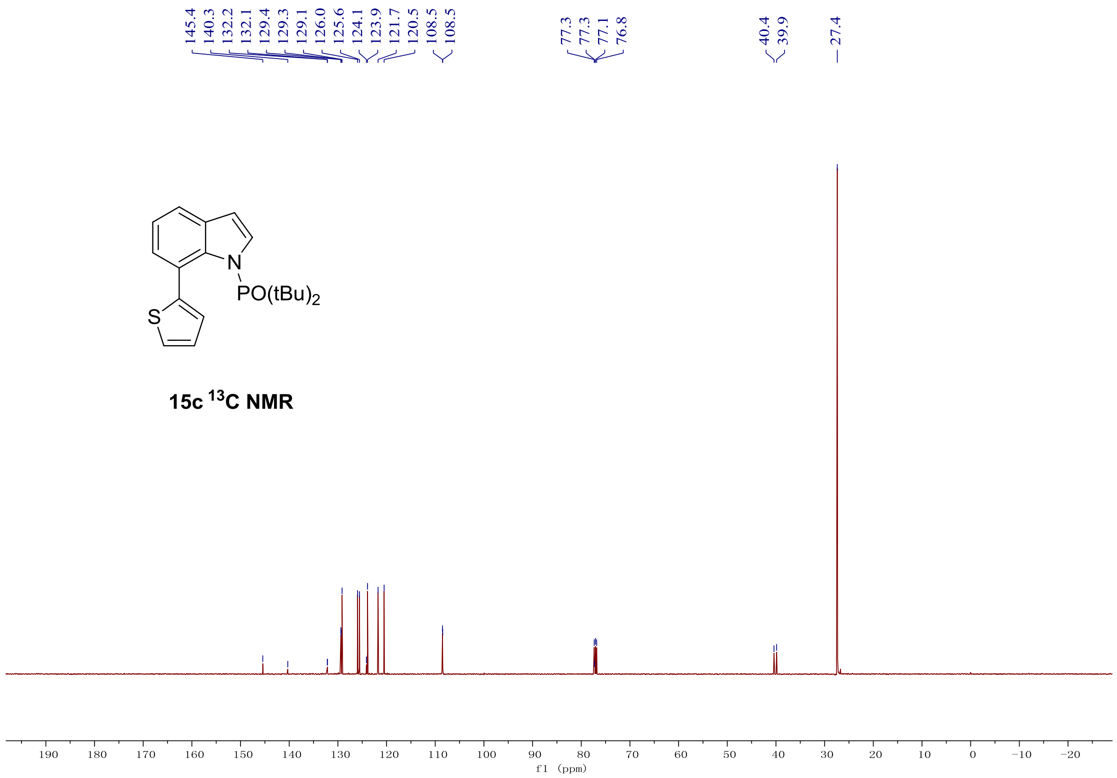

Supplementary Figure 105. ^{13}C NMR of compound 15c.

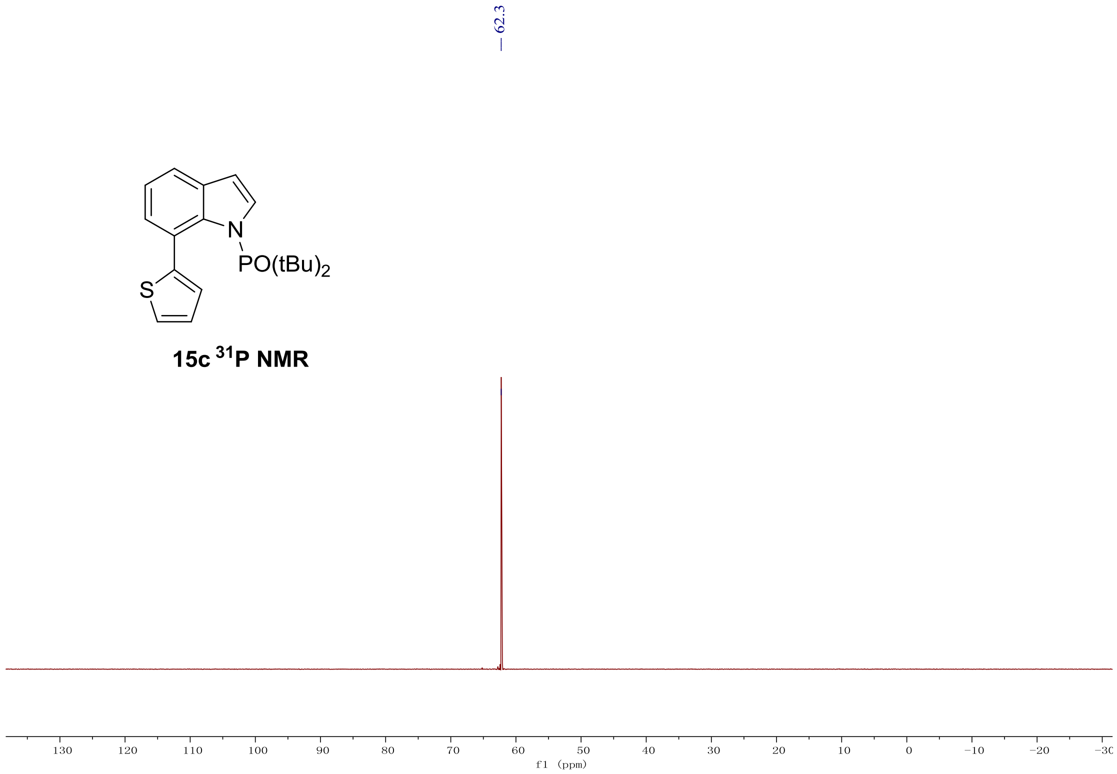

Supplementary Figure 106. ^{31}P NMR of compound 15c.

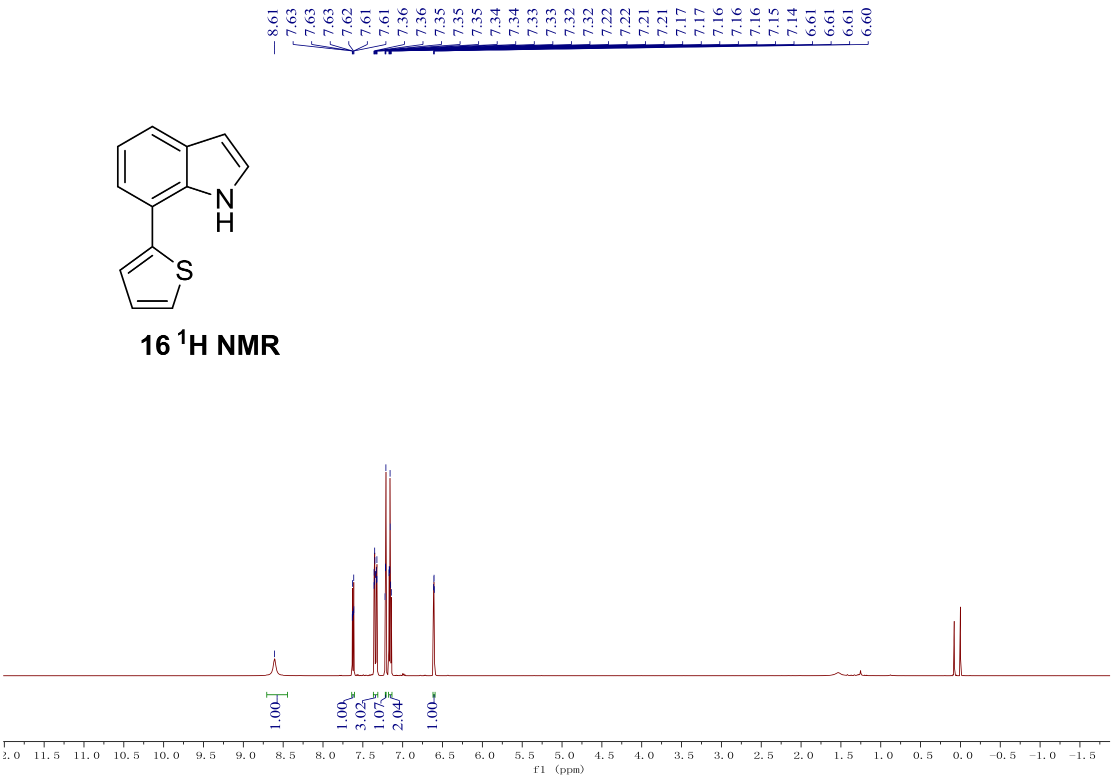

Supplementary Figure 107. ^{1}H NMR of compound 16.

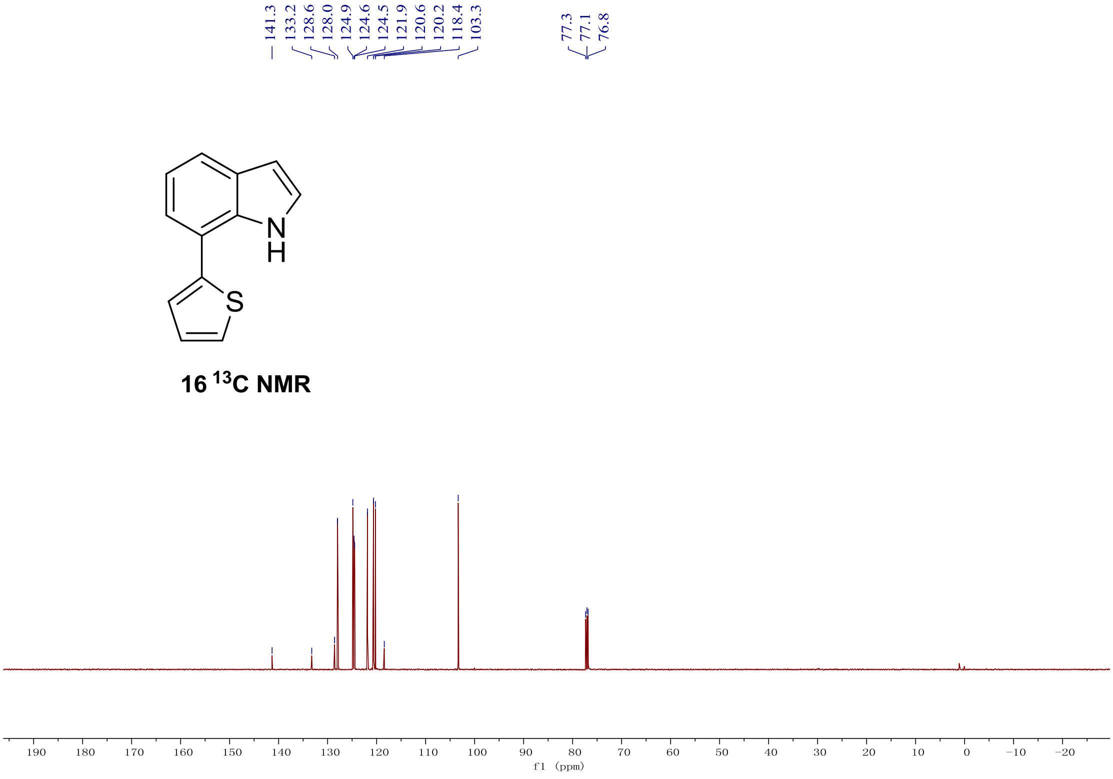

Supplementary Figure 108. ^{13}C NMR of compound 16.

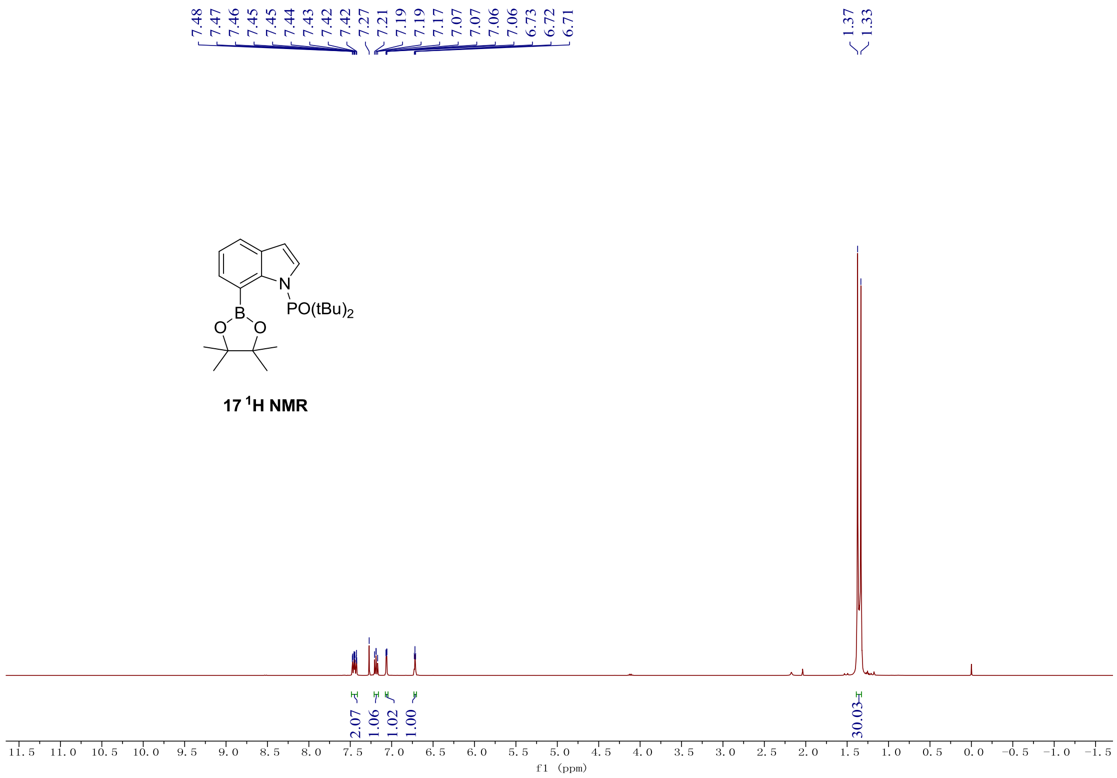

Supplementary Figure 109. ^{1}H NMR of compound 17.

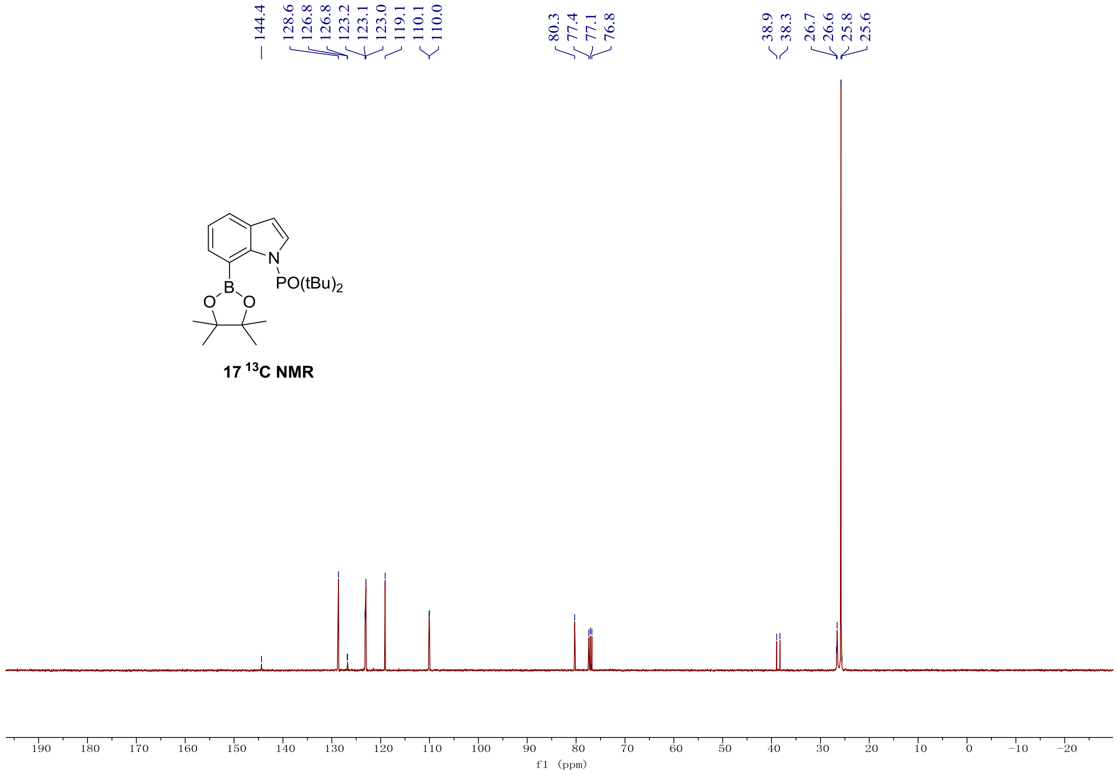

Supplementary Figure 110. ^{13}C NMR of compound 17.

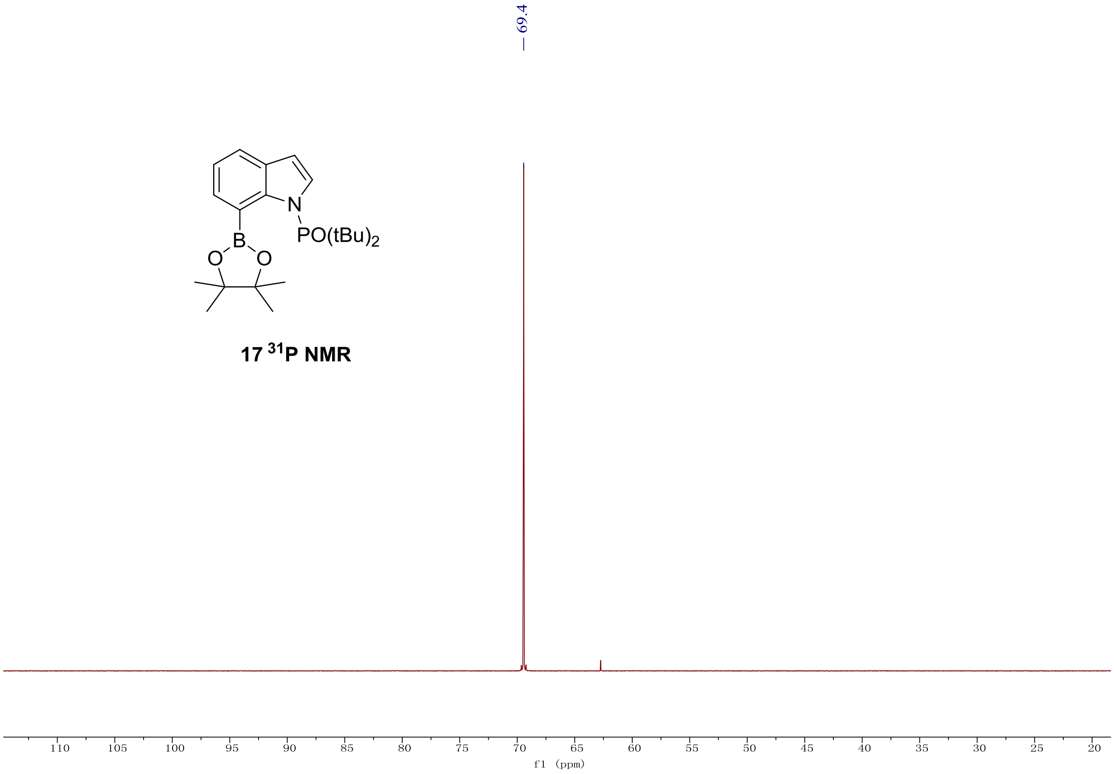

Supplementary Figure 111. ^{31}P NMR of compound 17.

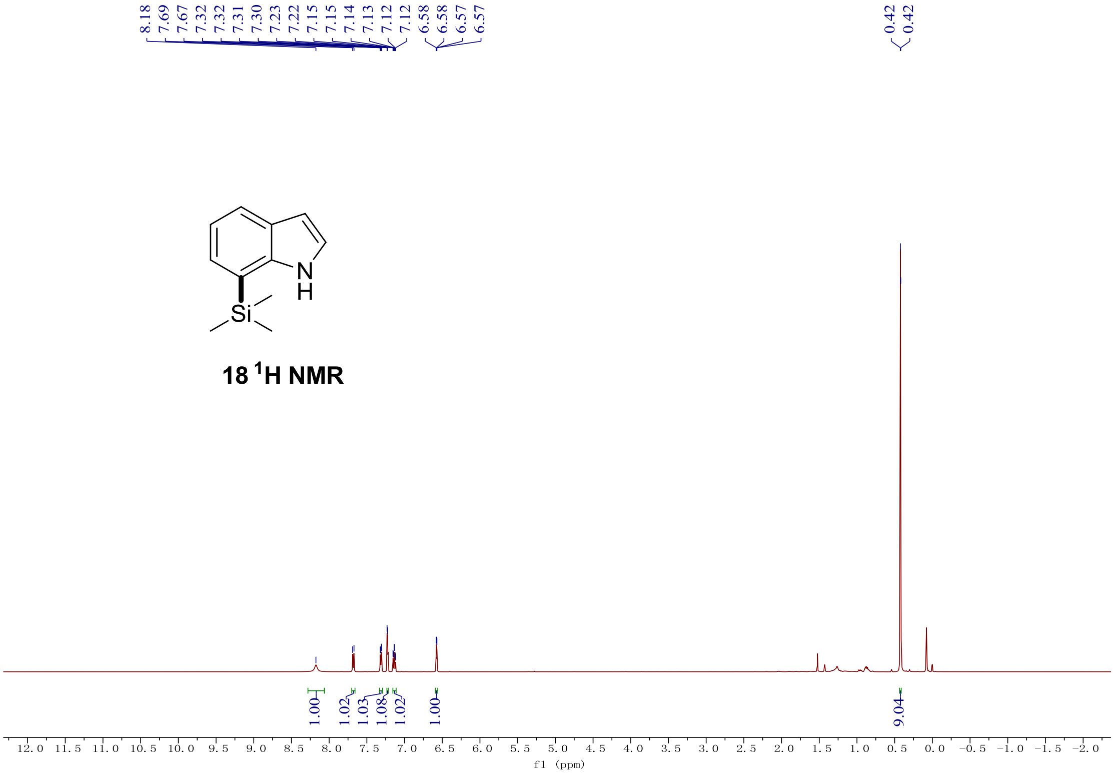

Supplementary Figure 112. ^{1}H NMR of compound 18.

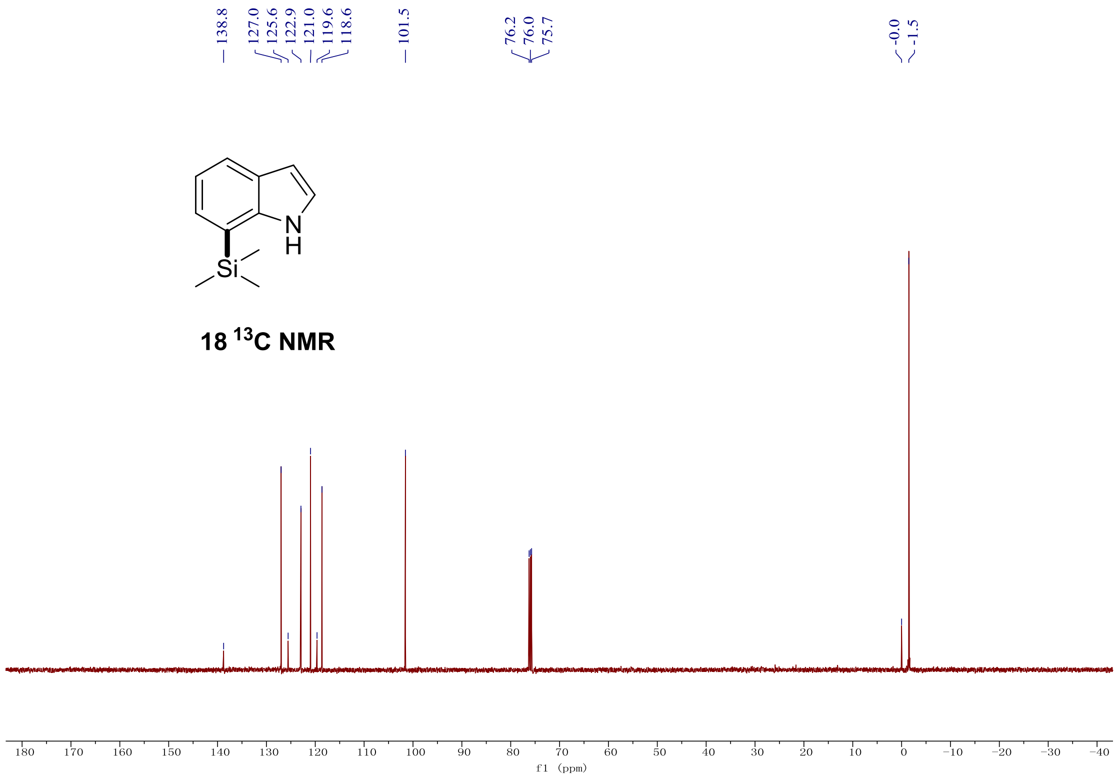

Supplementary Figure 113. ^{13}C NMR of compound 18.

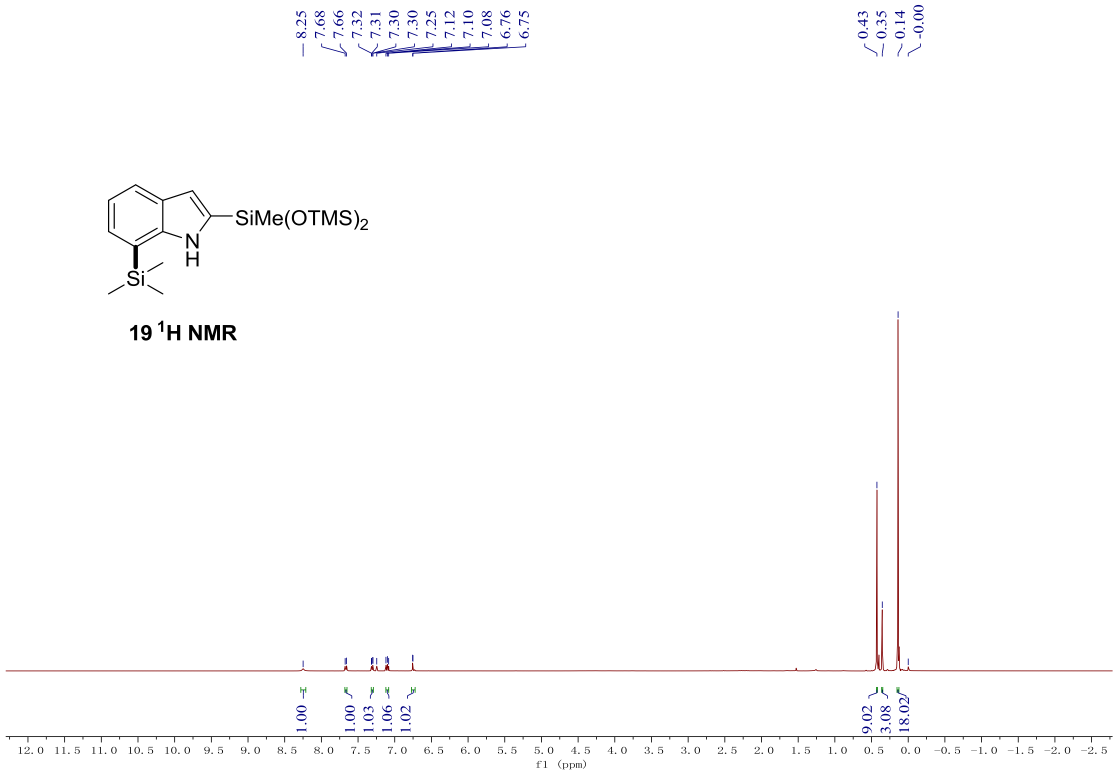

Supplementary Figure 114. ^{1}H NMR of compound 19.

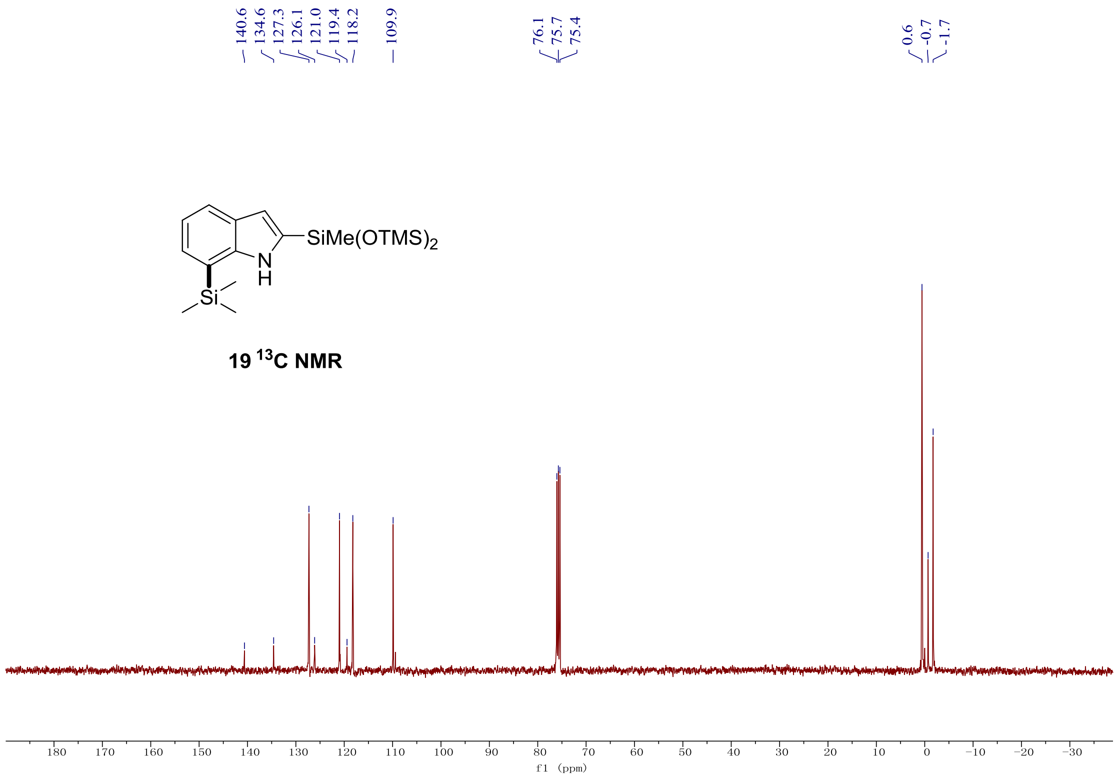

Supplementary Figure 115. ^{13}C NMR of compound 19.

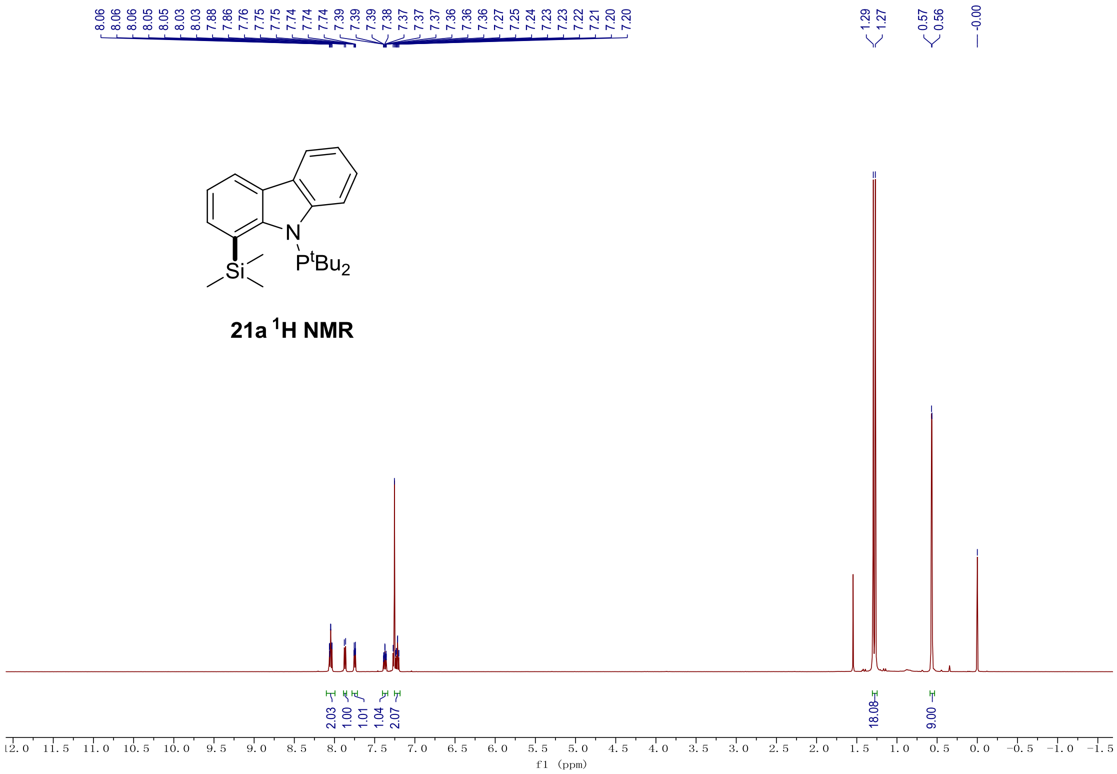

Supplementary Figure 116. ^{1}H NMR of compound 21a.

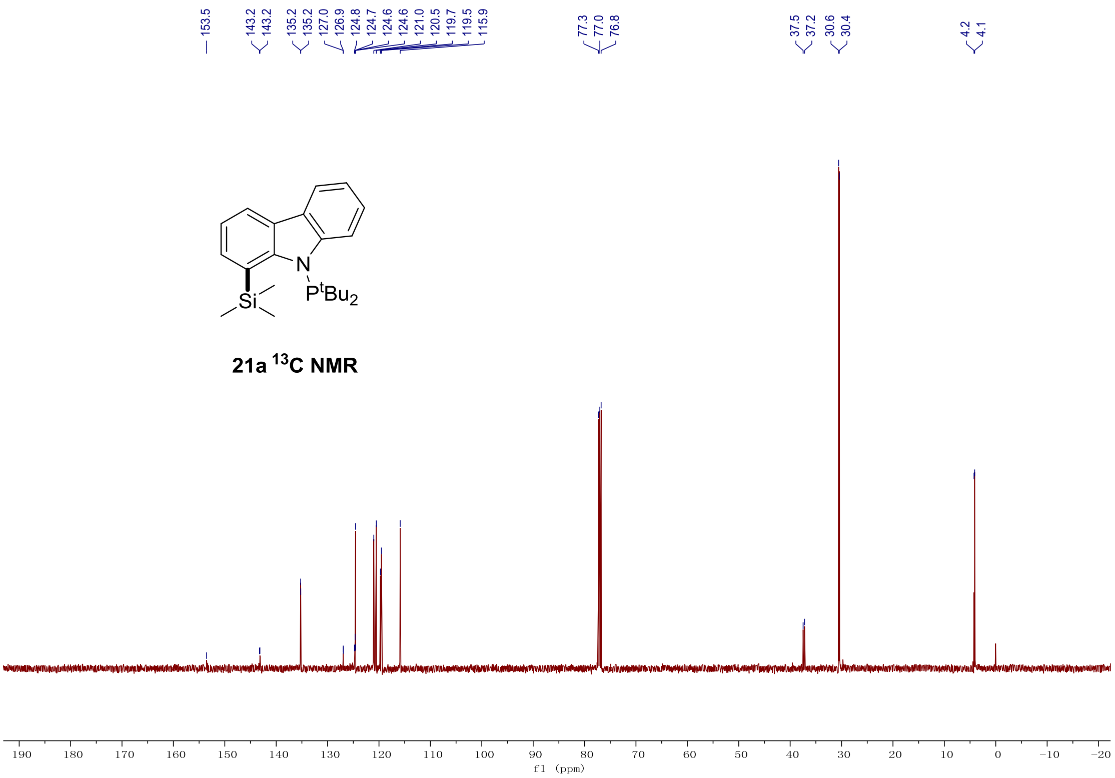

Supplementary Figure 117. ^{13}C NMR of compound 21a.

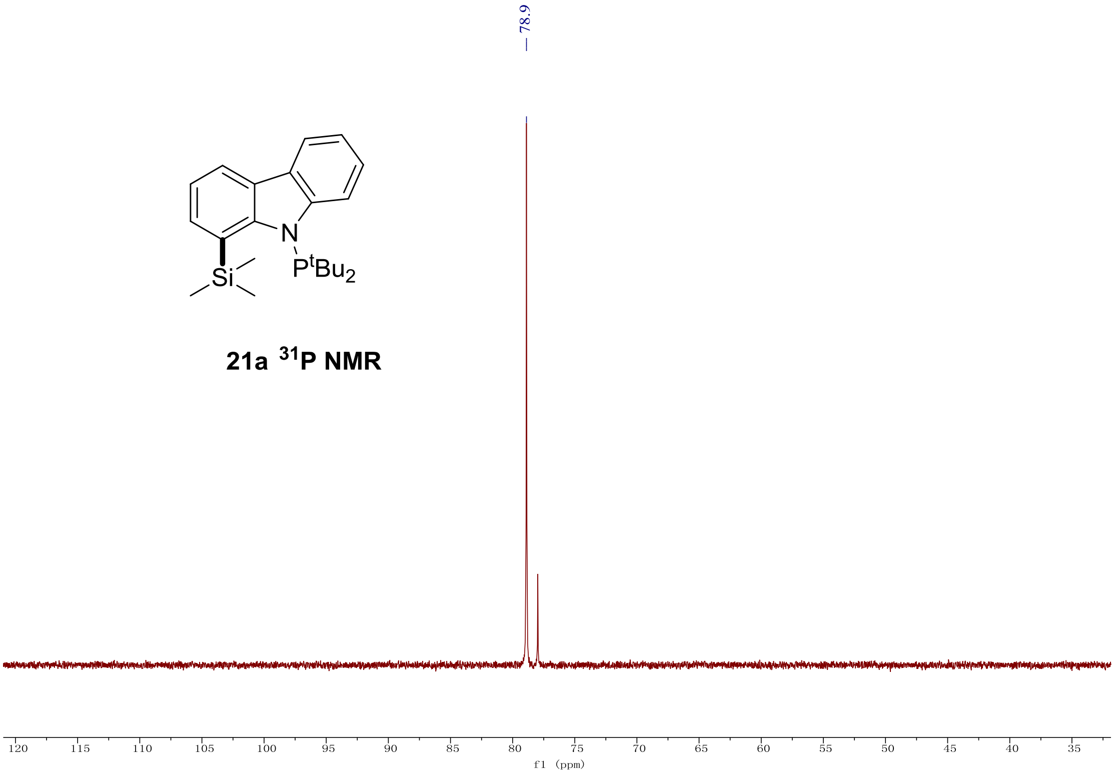

Supplementary Figure 118. ^{31}P NMR of compound 21a.

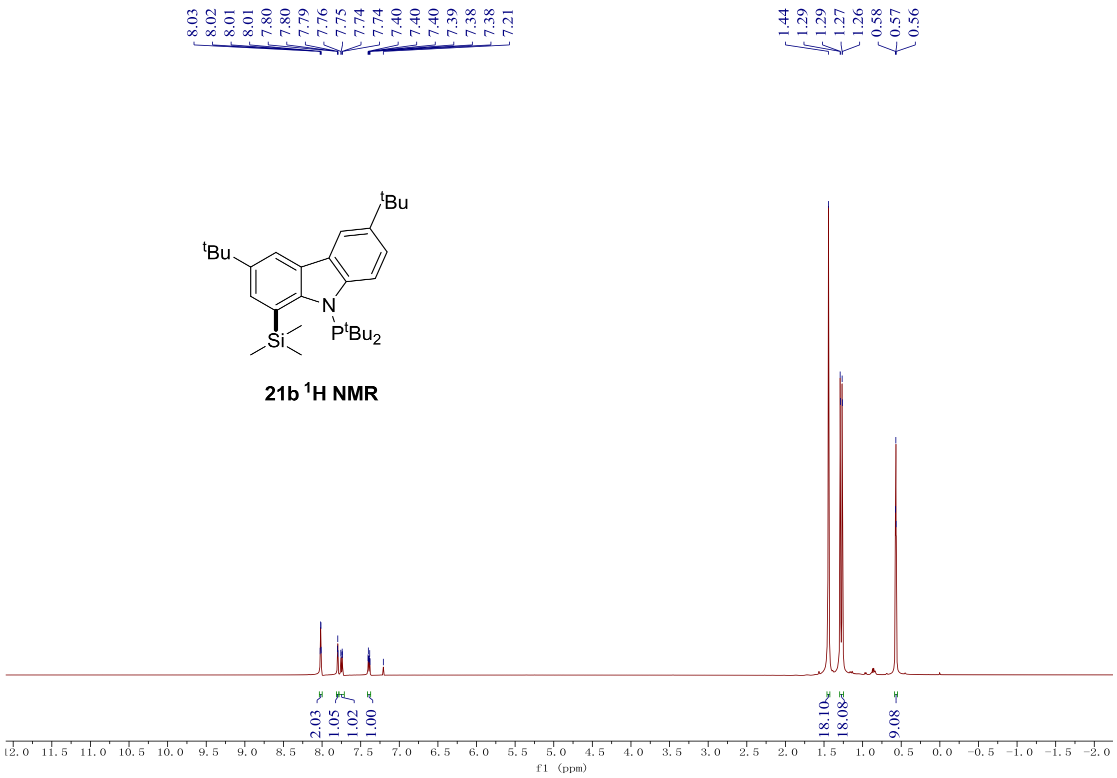

Supplementary Figure 119. ^{1}H NMR of compound 21b.

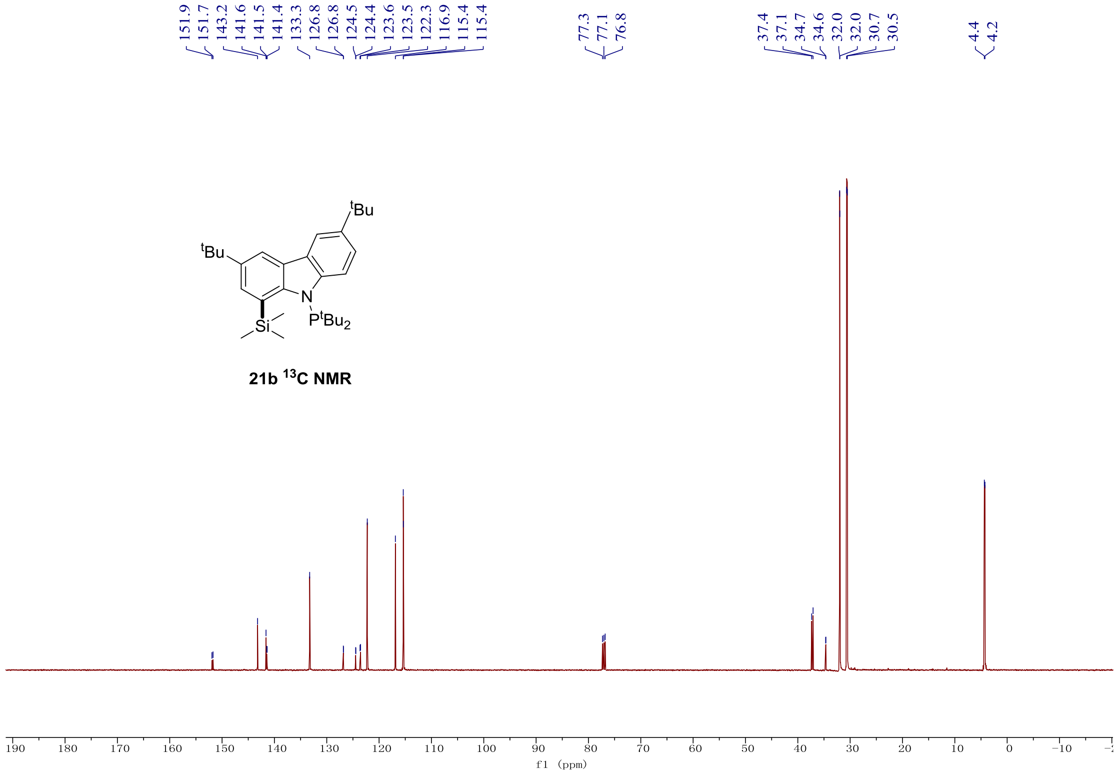

Supplementary Figure 120. ^{13}C NMR of compound 21b.

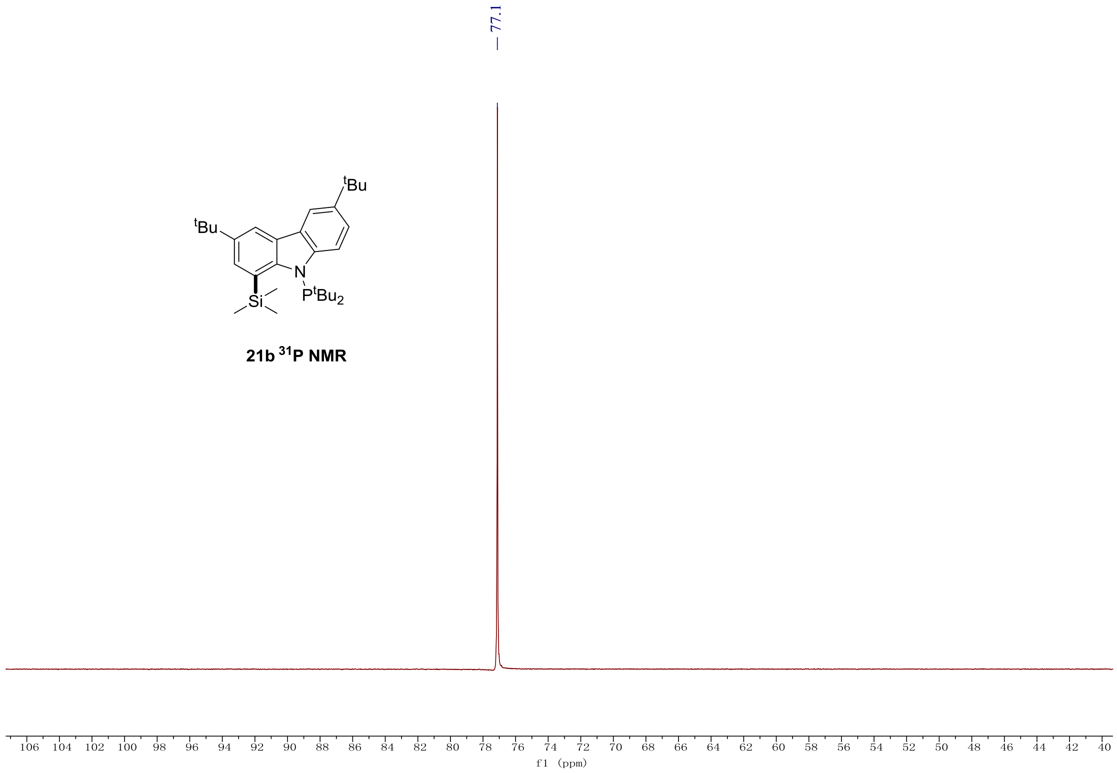

Supplementary Figure 121. ^{31}P NMR of compound 21b.

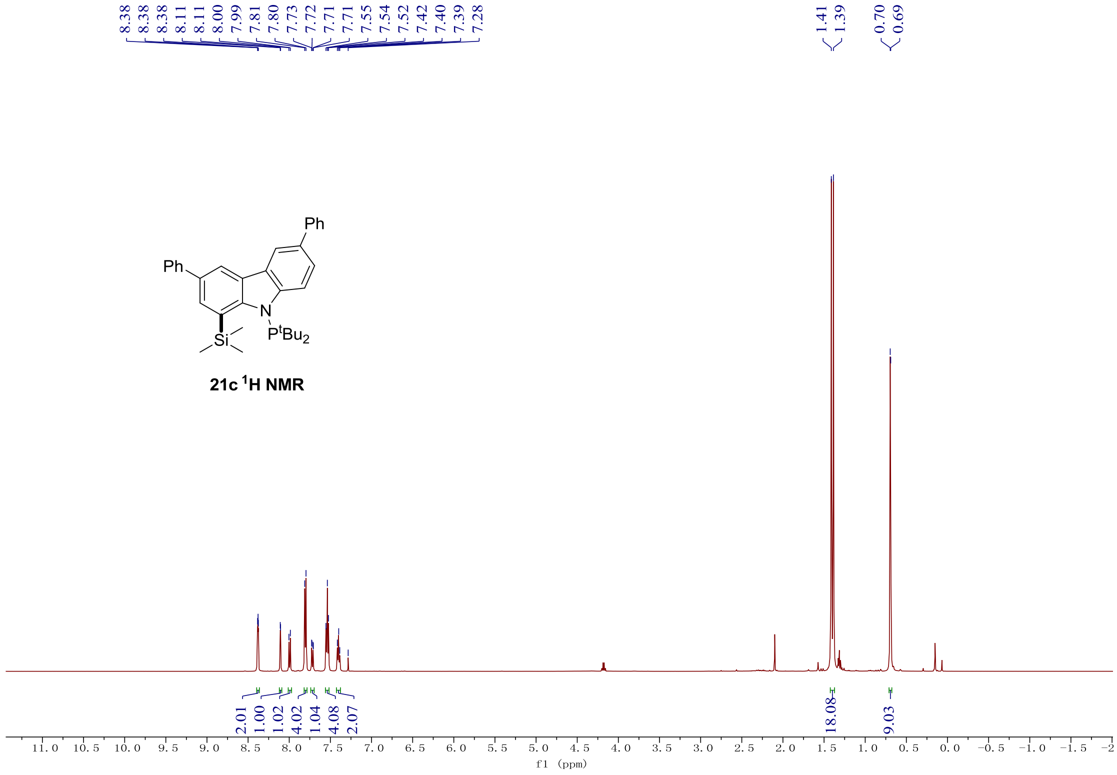

Supplementary Figure 122. ^{1}H NMR of compound 21c.

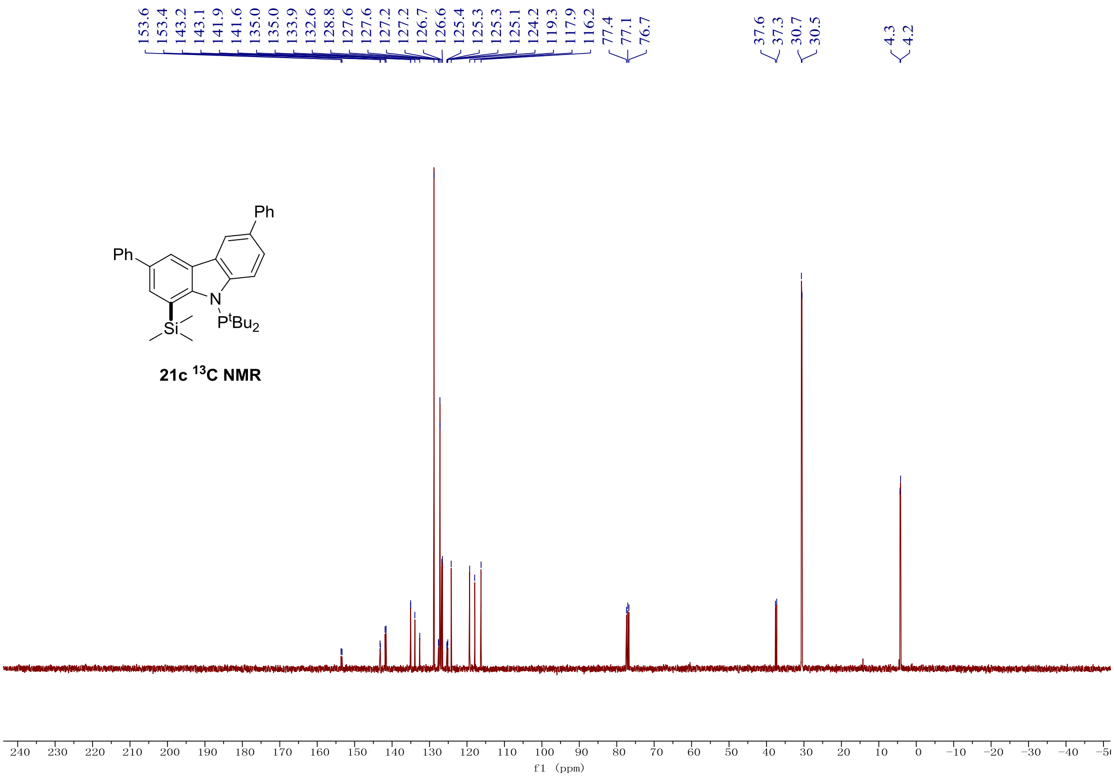

Supplementary Figure 123. ^{13}C NMR of compound 21c.

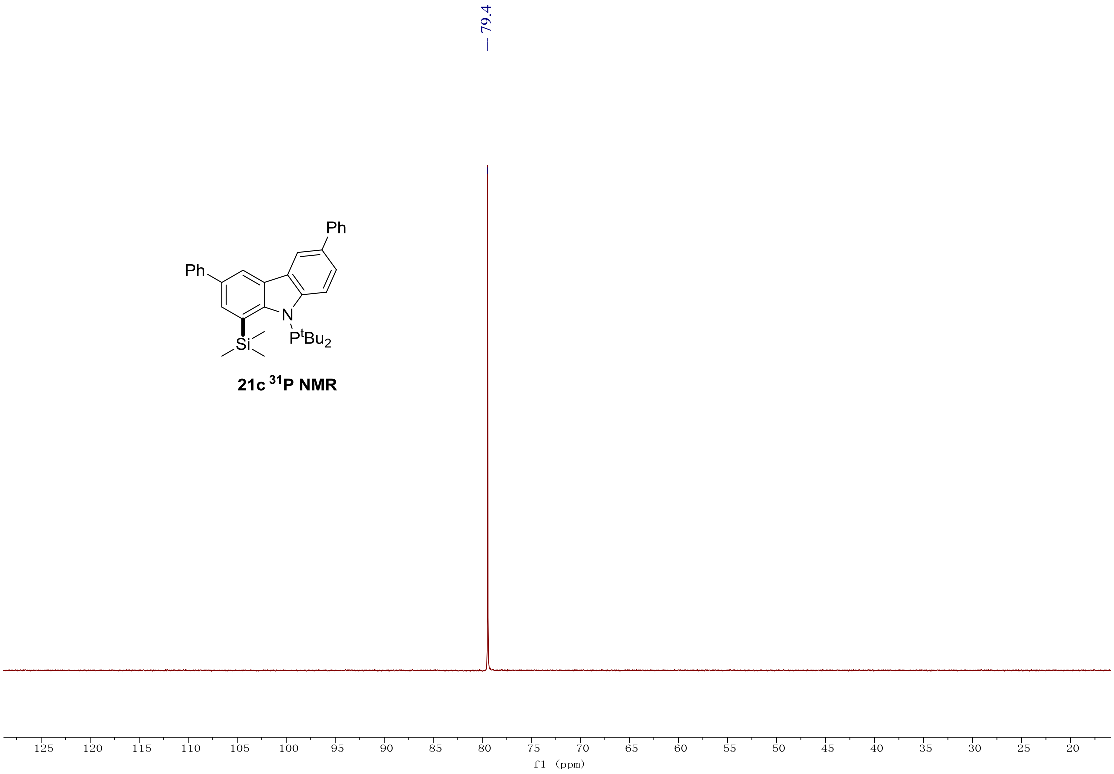

Supplementary Figure 124. ^{31}P NMR of compound 21c.

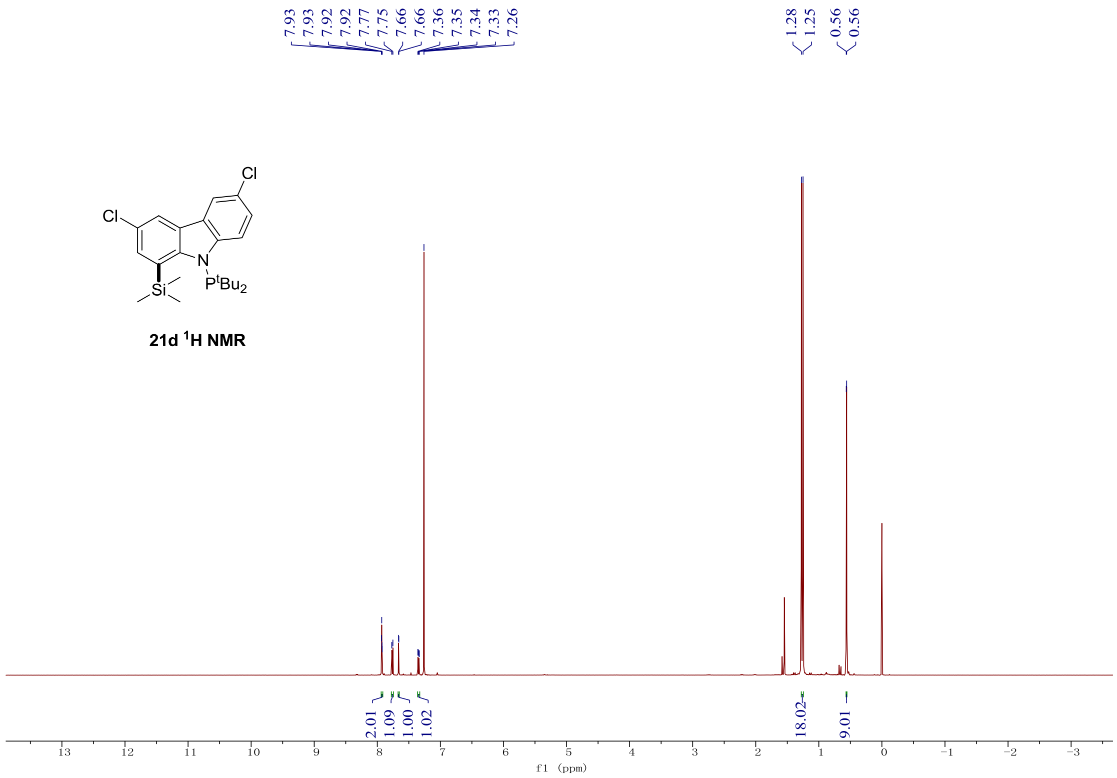

Supplementary Figure 125. ^{1}H NMR of compound 21d.

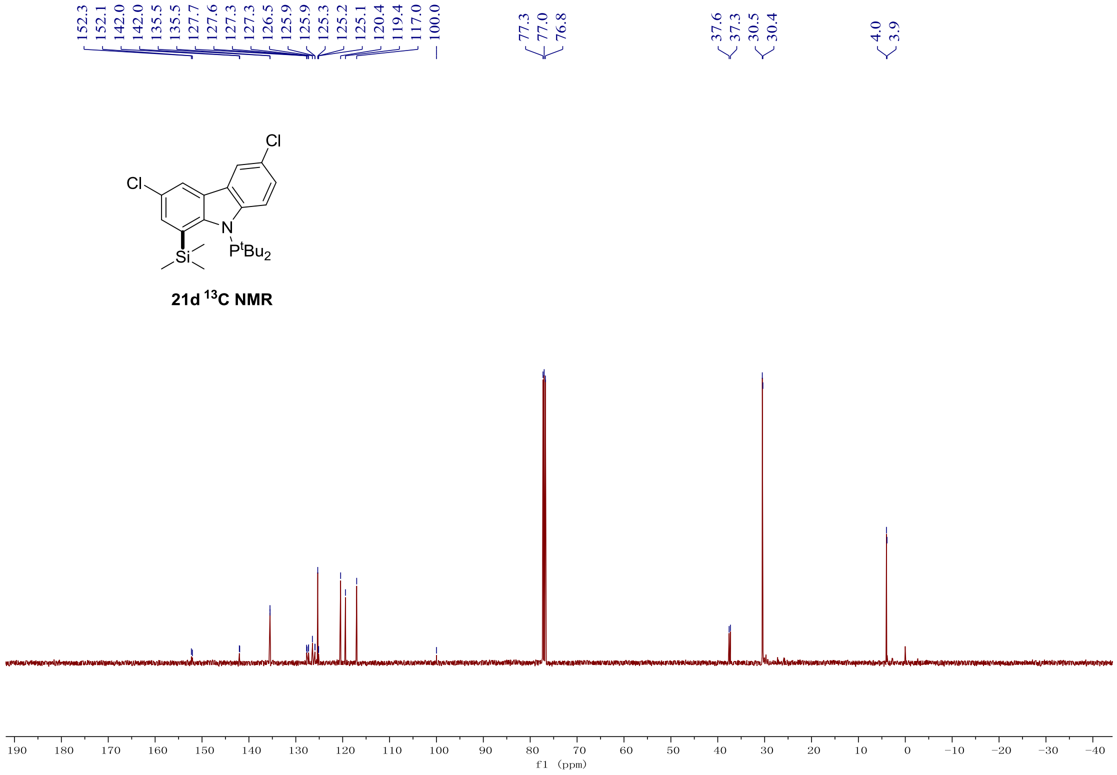

Supplementary Figure 126. ^{13}C NMR of compound 21d.

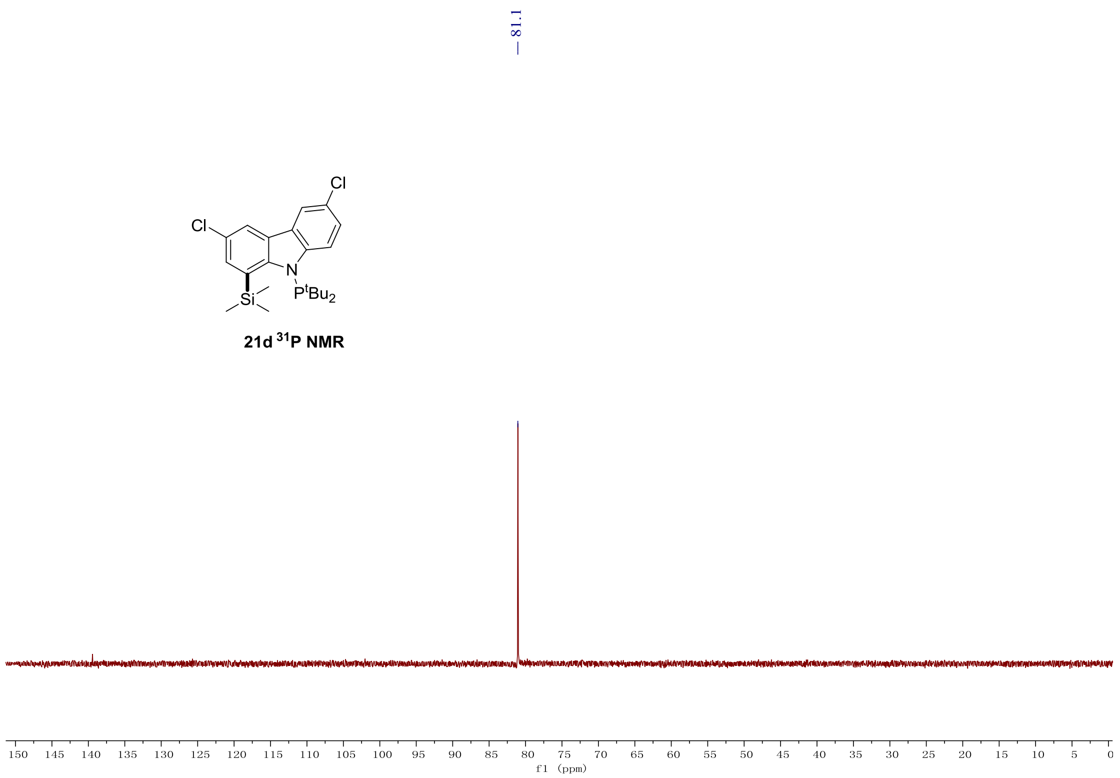

Supplementary Figure 127. ^{31}P NMR of compound 21d.

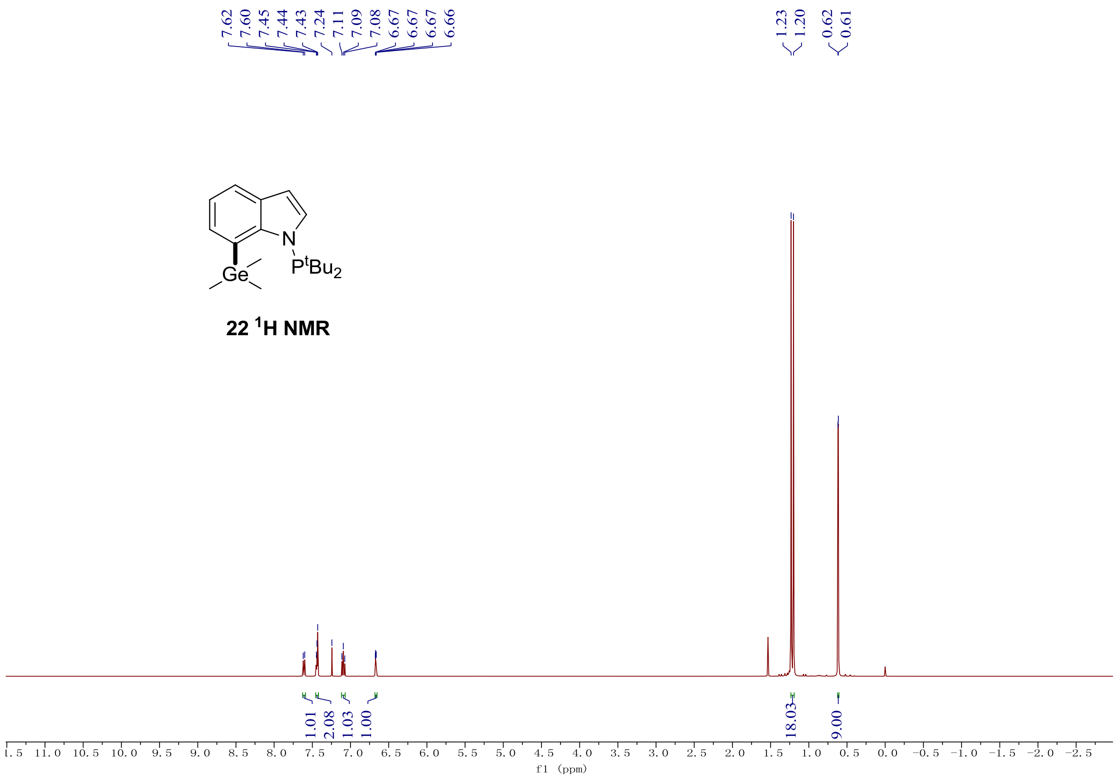

Supplementary Figure 128. ^{1}H NMR of compound 22.

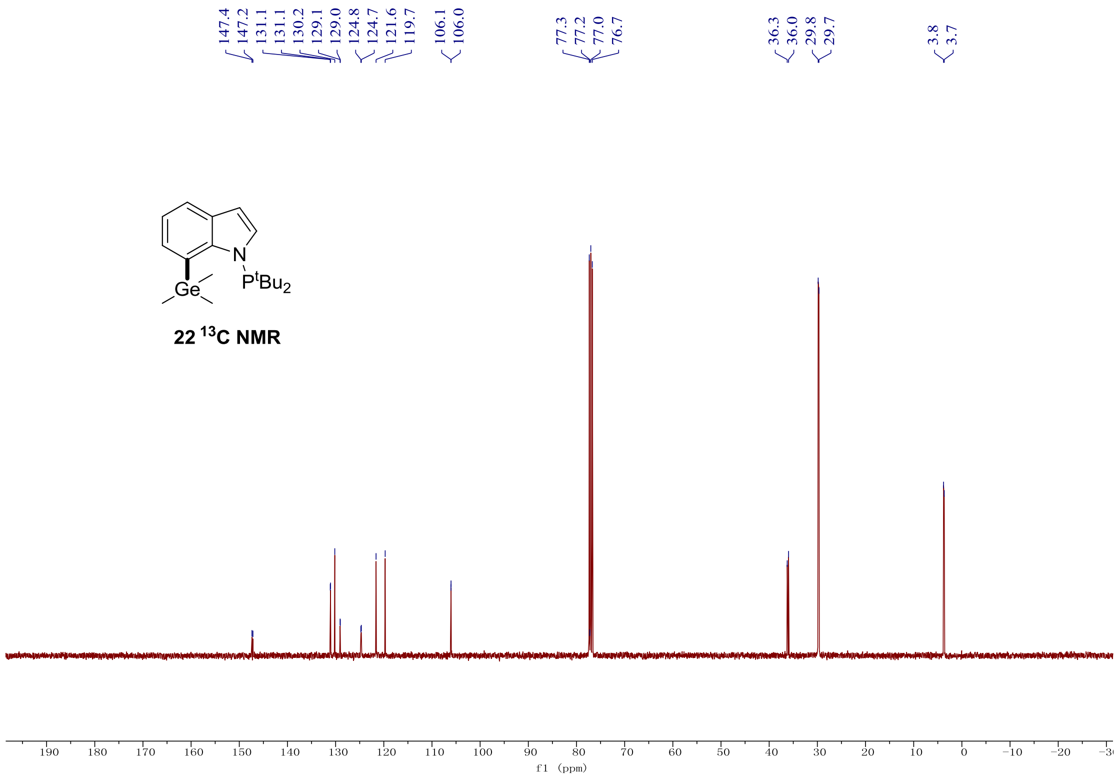

Supplementary Figure 129. ^{13}C NMR of compound 22.

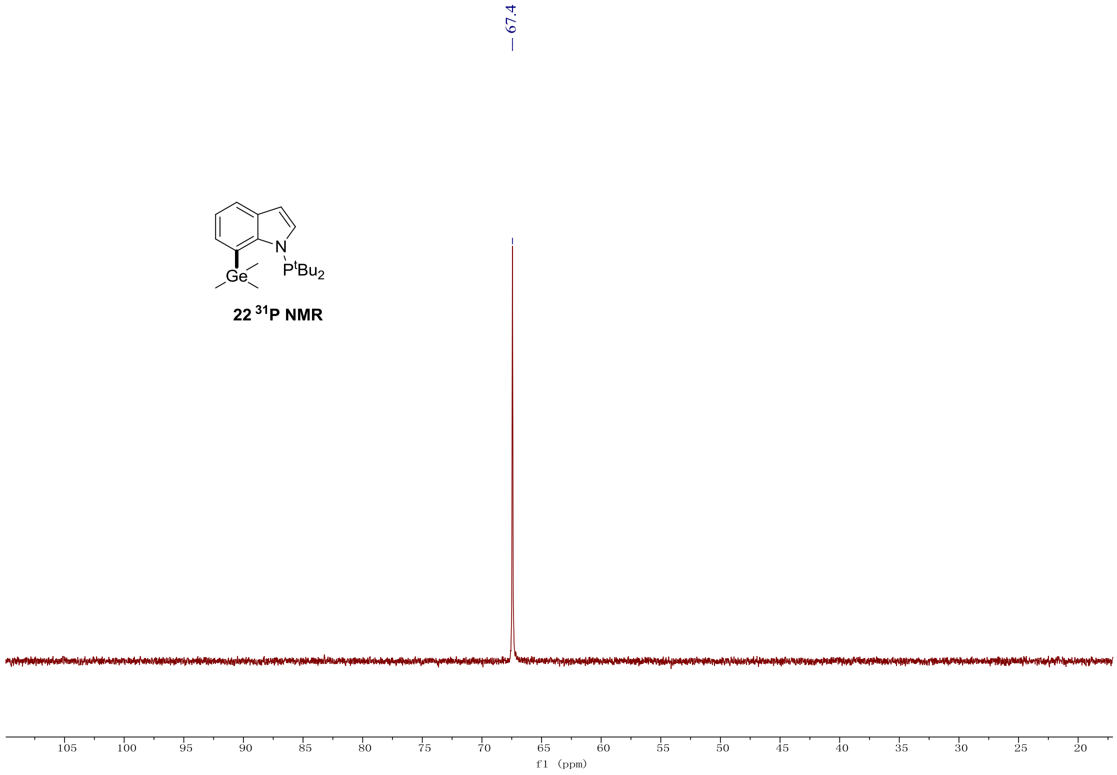

Supplementary Figure 130. ^{31}P NMR of compound 22.

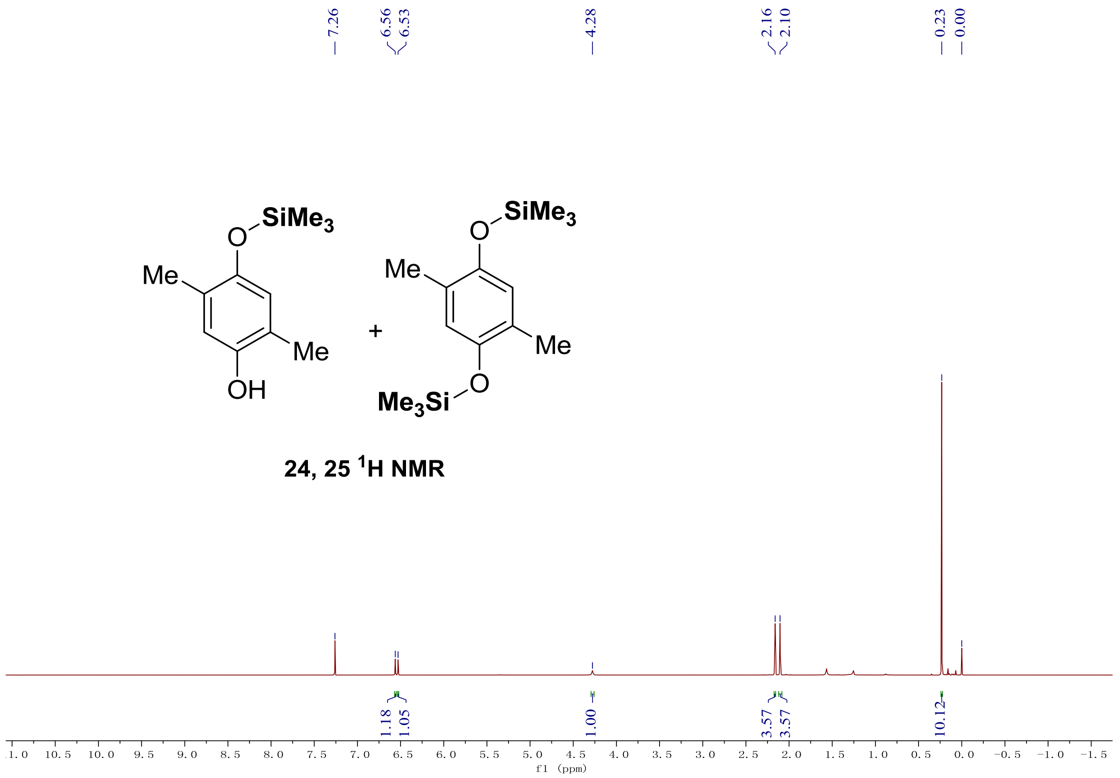

Supplementary Figure 131. ^{1}H NMR of compound 24, 25.

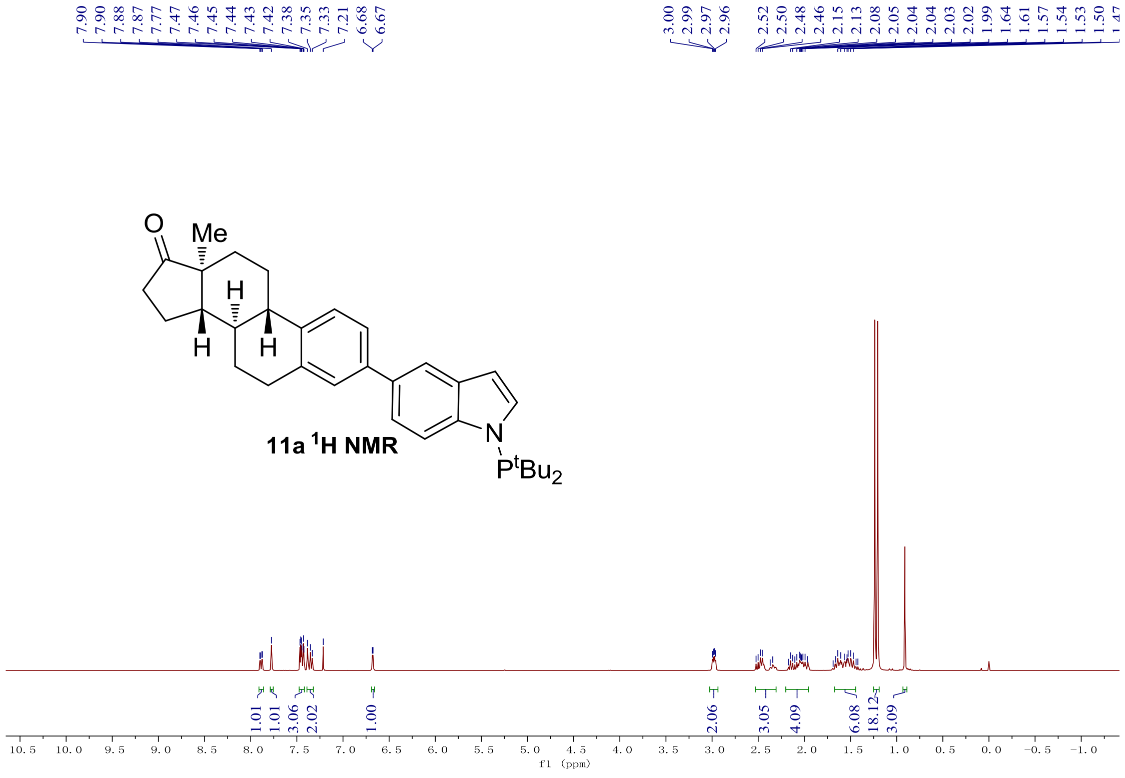

Supplementary Figure 132. ^{1}H NMR of compound 11a.

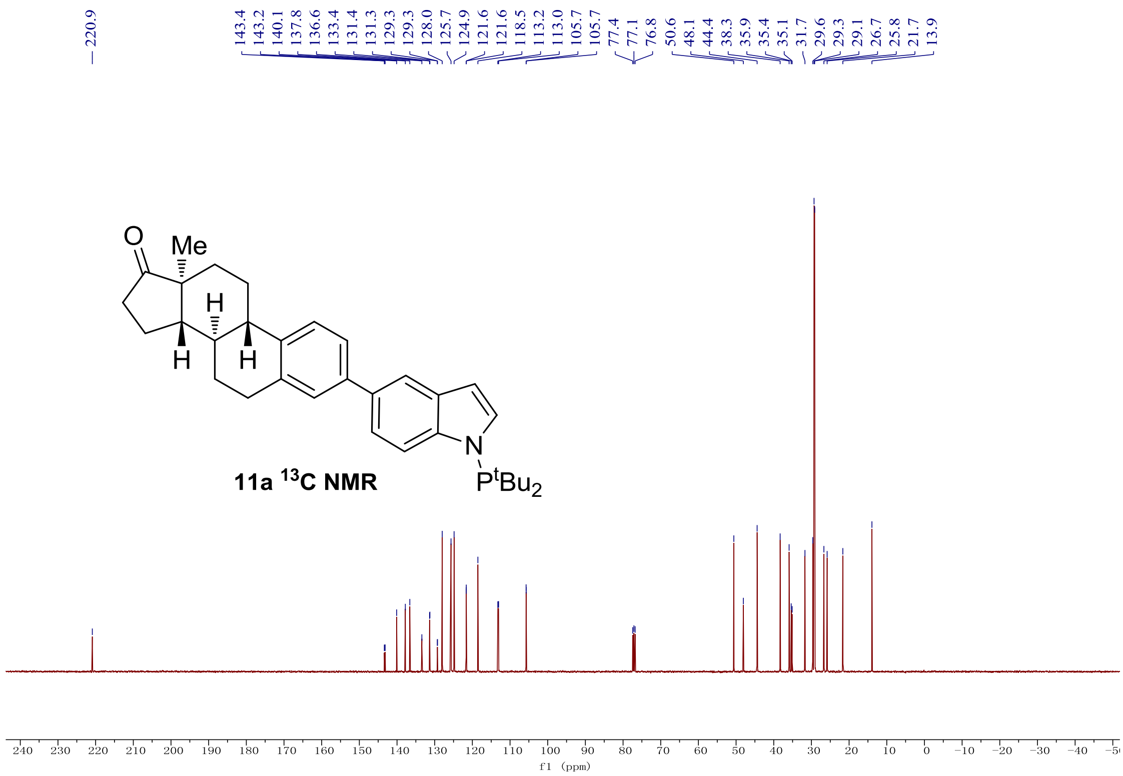

Supplementary Figure 133. ^{13}**C NMR of compound 11a.**

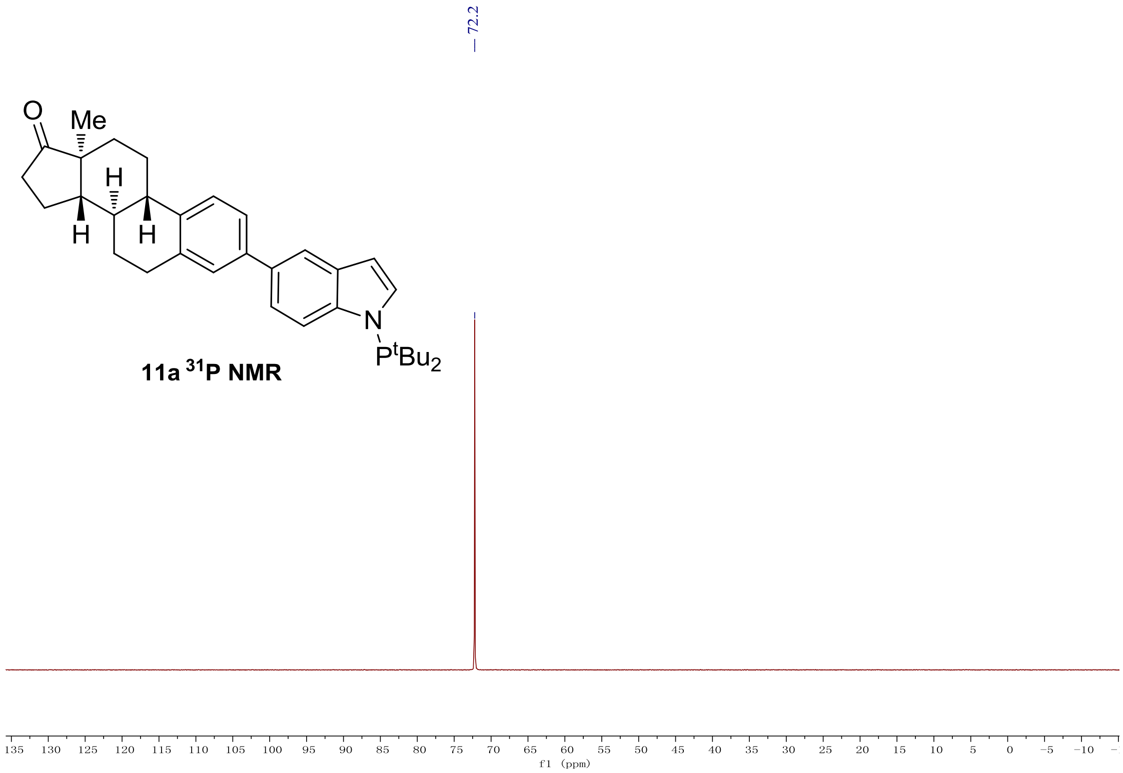

Supplementary Figure 134. ^{31}**P NMR of compound 11a.**

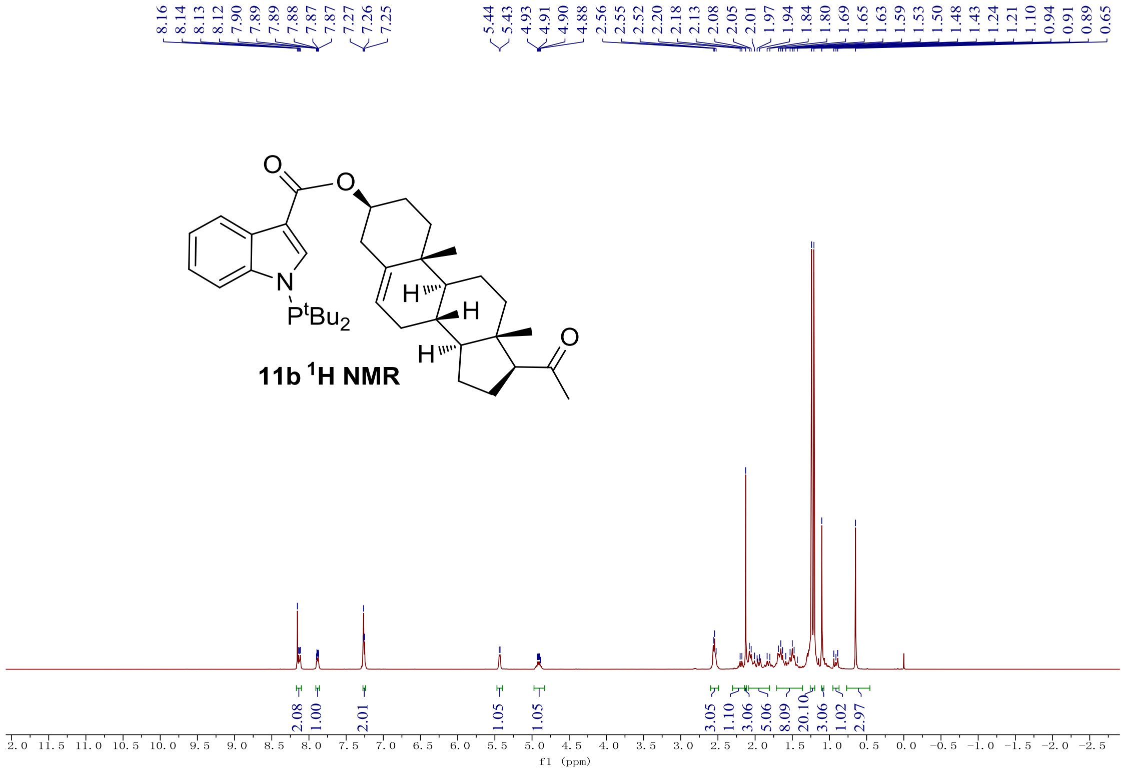

Supplementary Figure 135. ^{1}H NMR of compound 11b.

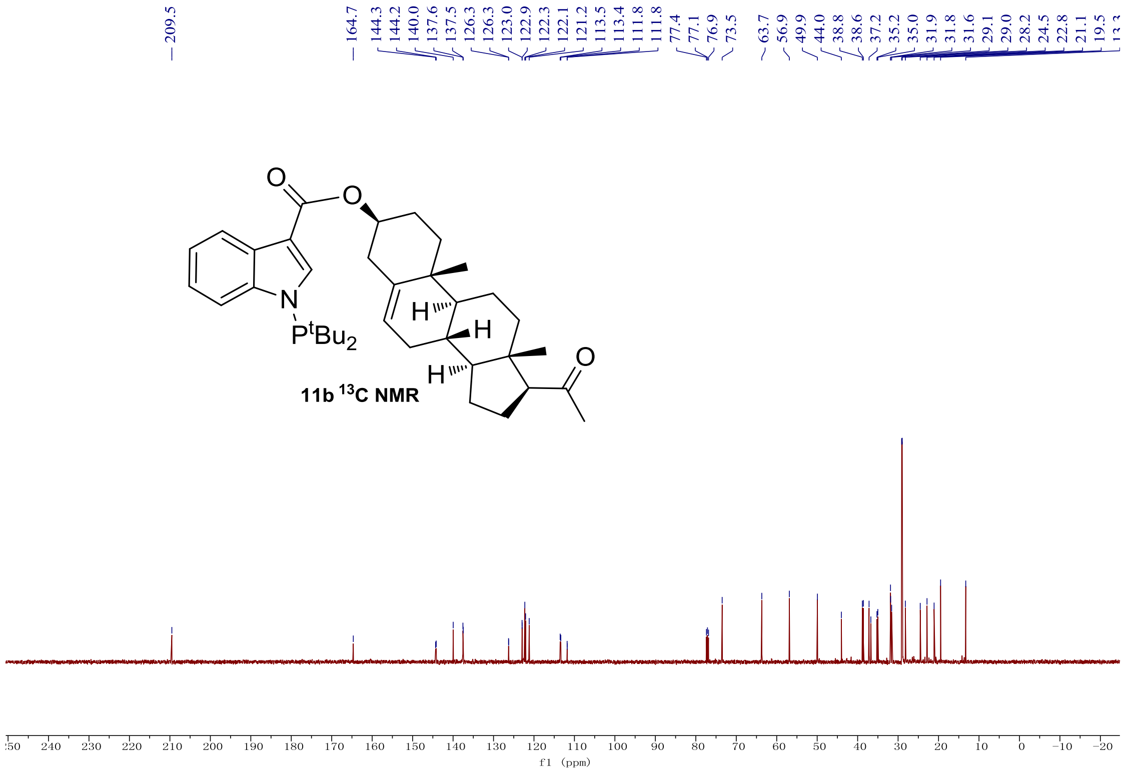

Supplementary Figure 136. ^{13}C NMR of compound 11b.

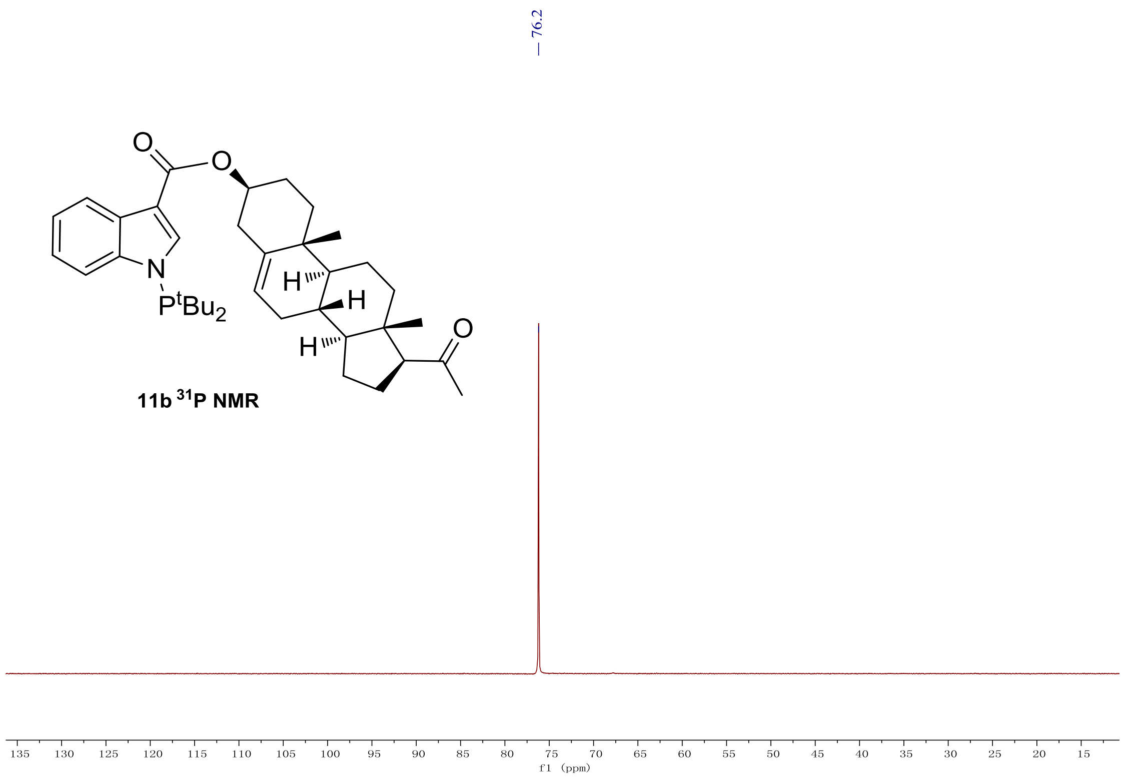

Supplementary Figure 137. ^{31}**P NMR of compound 11b.**

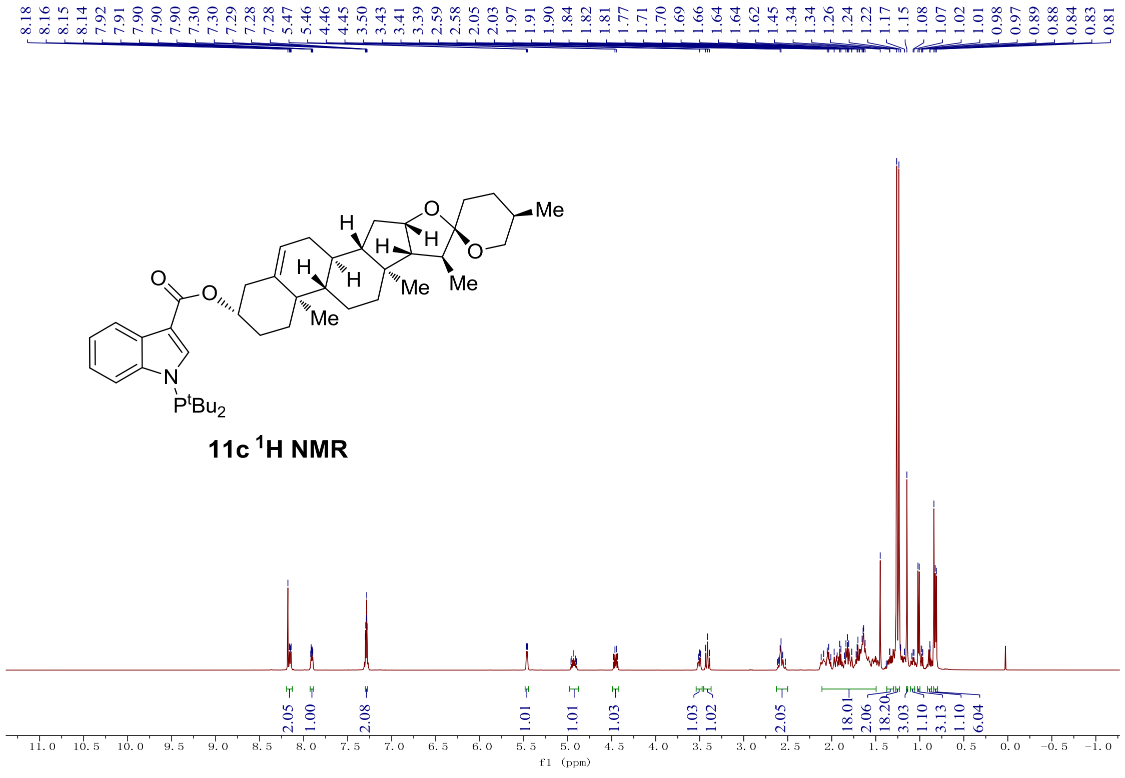

Supplementary Figure 138. ^{1}**H NMR of compound 11c.**

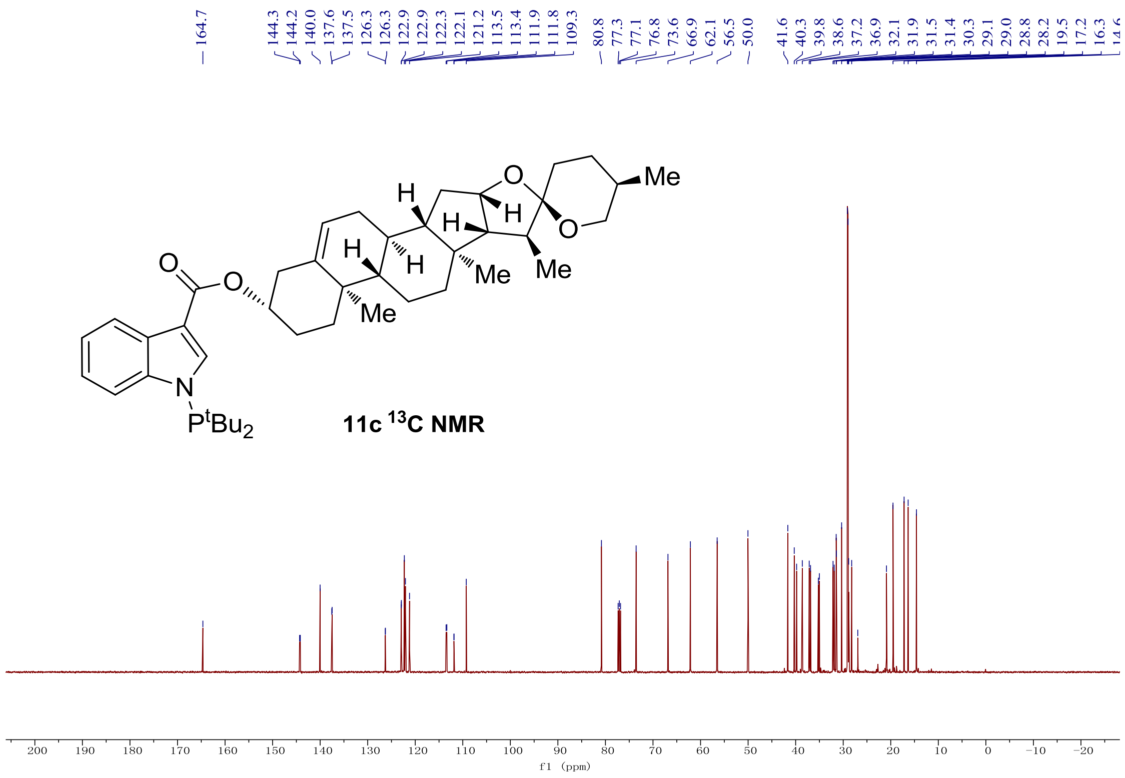

Supplementary Figure 139. ^{13}C NMR of compound 11c.

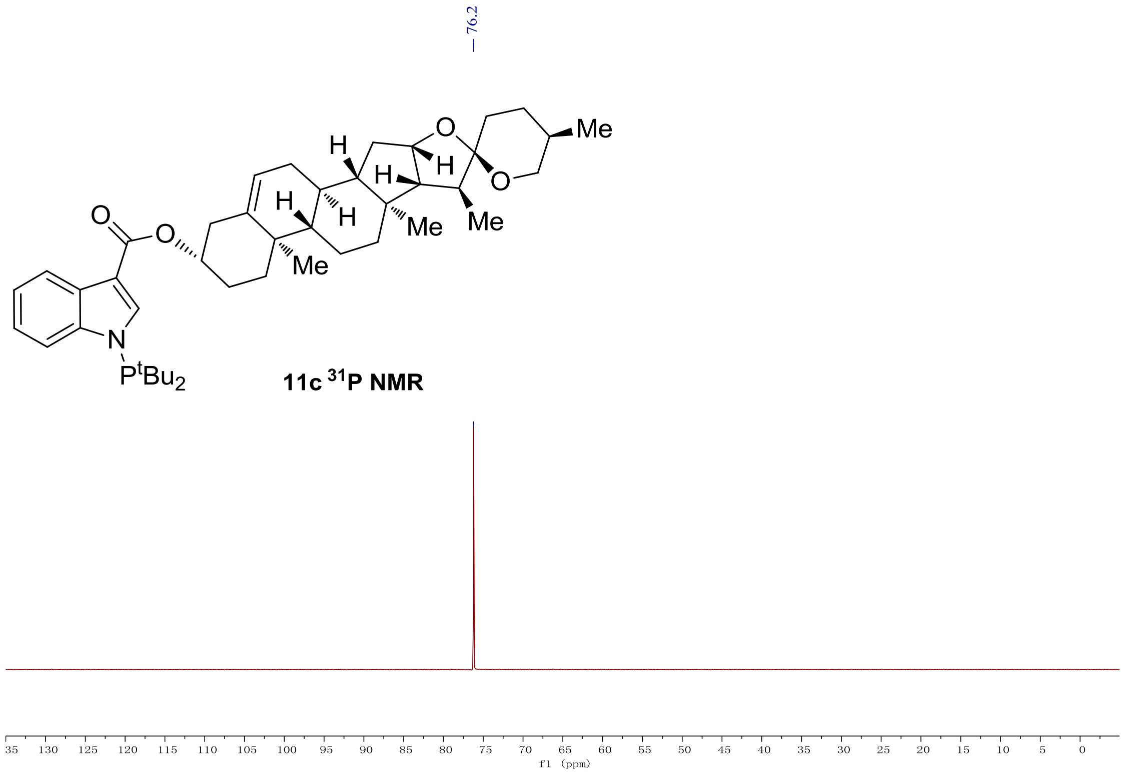

Supplementary Figure 140. ^{31}P NMR of compound 11c.

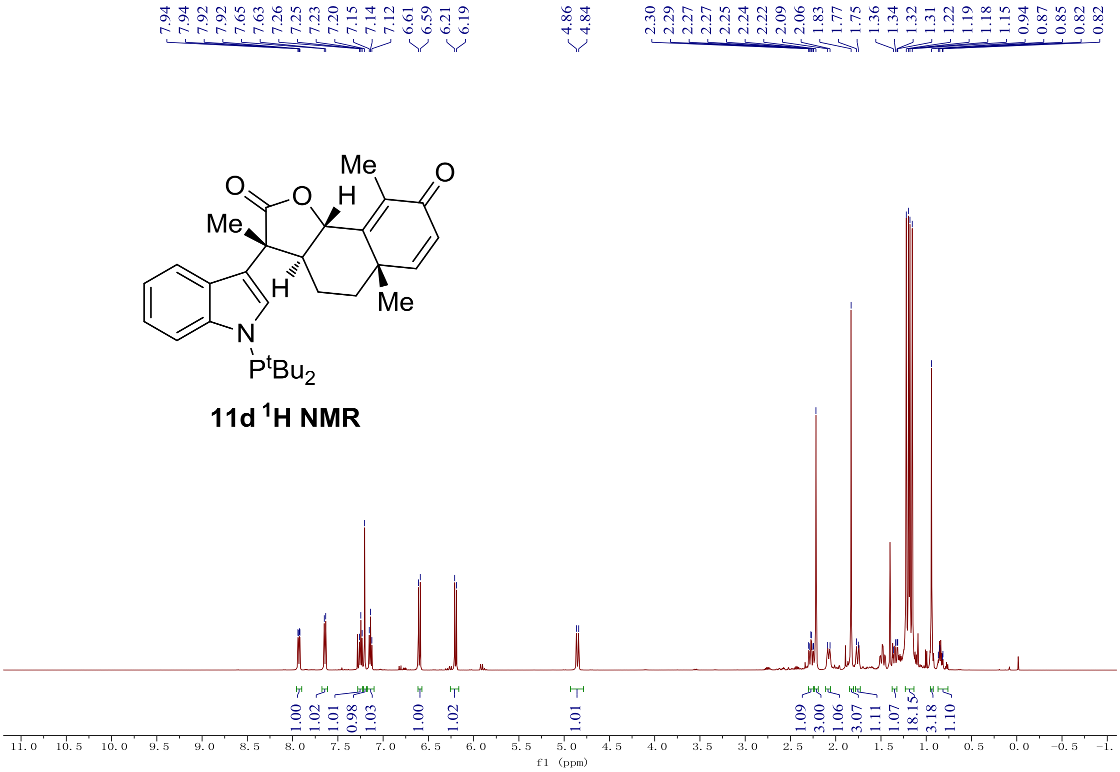

Supplementary Figure 141. ^{1}**H NMR of compound 11d.**

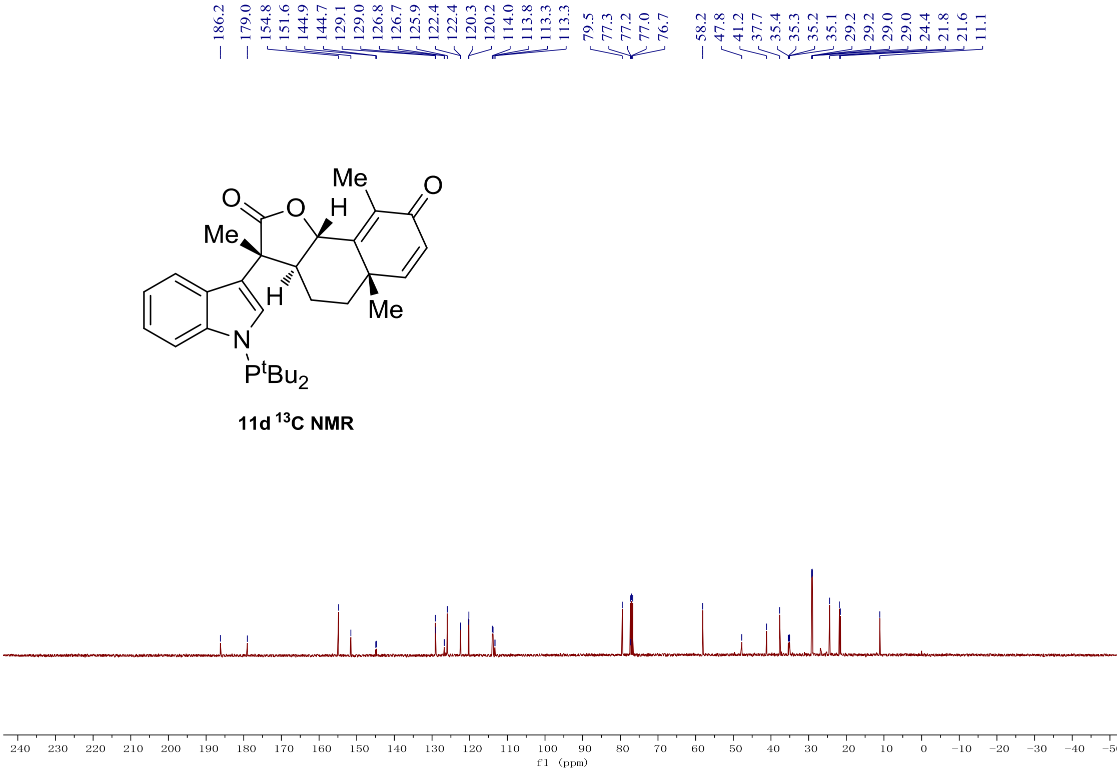

Supplementary Figure 142. ^{13}**C NMR of compound 11d.**

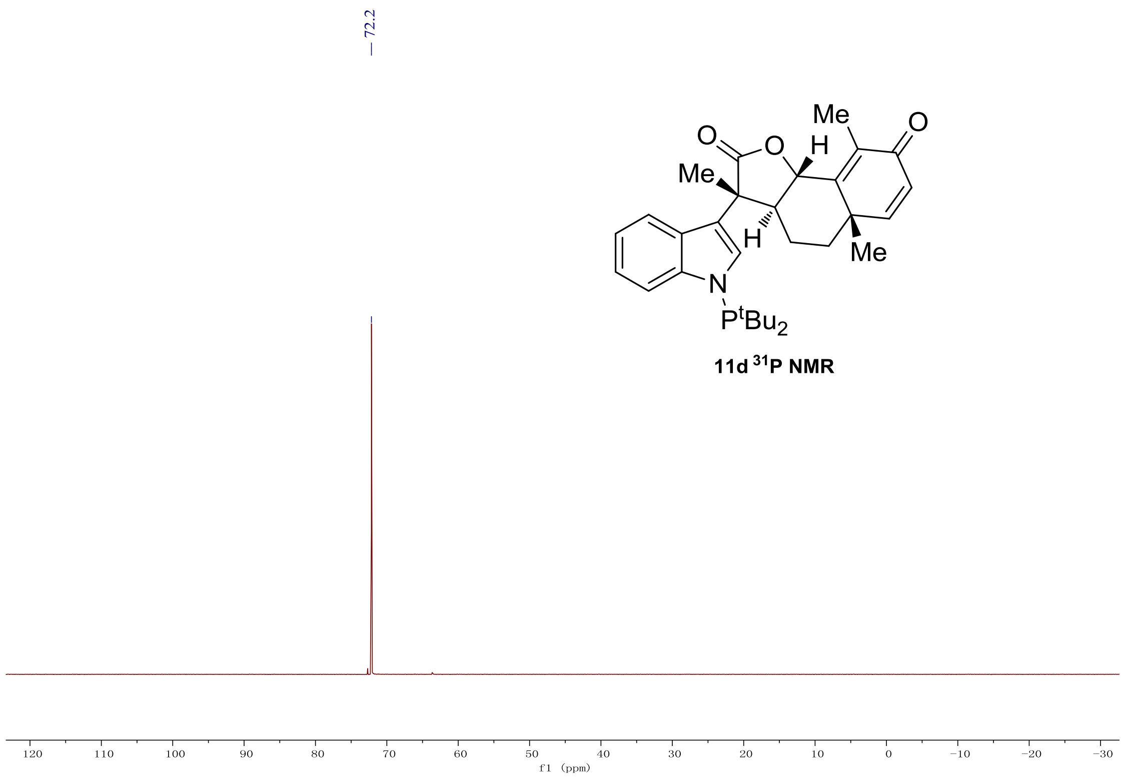

Supplementary Figure 143. ^{31}P NMR of compound 11d.

Supplementary References

1. Frisch, M. J.; Trucks, G. W.; Schlegel, H. B.; Scuseria, G. E.; Robb, M. A.; Cheeseman, J. R.; Scalmani, G.; Barone, V.; Mennucci, B.; Petersson, G. A.; Nakatsuji, H.; Caricato, M.; Li, X.; Hratchian, H. P.; Izmaylov, A. F.; Bloino, J.; Zheng, G.; Sonnenberg, J. L.; Hada, M.; Ehara, M.; Toyota, K.; Fukuda, R.; Hasegawa, J.; Ishida, M.; Nakajima, T.; Honda, Y.; Kitao, O.; Nakai, H.; Vreven, T.; Montgomery Jr., J. A.; Peralta, J. E.; Ogliaro, F.; Bearpark, M. J.; Heyd, J.; Brothers, E. N.; Kudin, K. N.; Staroverov, V. N.; Kobayashi, R.; Normand, J.; Raghavachari, K.; Rendell, A. P.; Burant, J. C.; Iyengar, S. S.; Tomasi, J.; Cossi, M.; Rega, N.; Millam, N. J.; Klene, M.; Knox, J. E.; Cross, J. B.; Bakken, V.; Adamo, C.; Jaramillo, J.; Gomperts, R.; Stratmann, R. E.; Yazyev, O.; Austin, A. J.; Cammi, R.; Pomelli, C.; Ochterski, J. W.; Martin, R. L.; Morokuma, K.; Zakrzewski, V. G.; Voth, G. A.; Salvador, P.; Dannenberg, J. J.; Dapprich, S.; Daniels, A. D.; Farkas, Ö.; Foresman, J. B.; Ortiz, J. V.; Cioslowski, J.; Fox, D. J. *Gaussian 09*, revision C.01; Gaussian, Inc.: Wallingford, CT, USA, 2009.

2. Becke, A. D., Density‐functional thermochemistry. III. The role of exact exchange. *J. Chem. Phys.* **1993,** *98* (7), 5648-5652.

3. Becke, A. D., A new mixing of Hartree–Fock and local density‐functional theories. *J. Chem. Phys.* **1993,** *98* (2), 1372-1377.

4. Stephens, P. J.; Devlin, F. J.; Chabalowski, C. F.; Frisch, M. J., Ab Initio Calculation of Vibrational Absorption and Circular Dichroism Spectra Using Density Functional Force Fields. *J. Phys. Chem.* **1994,** *98* (45), 11623-11627.

5. Grimme, S.; Antony, J.; Ehrlich, S.; Krieg, H. A Consistent and Accurate ab initio Parameterization of Density Functional Dispersion Correction (DFT-D) for the 94 elements H-Pu," *J. Chem. Phys.* **2010**, 132, 154104.

6. Ditchfield, R.; Hehre, W. J.; Pople, J. A., Self‐Consistent Molecular‐Orbital Methods. IX. An Extended Gaussian‐Type Basis for Molecular‐Orbital Studies of Organic Molecules. *J. Chem. Phys.* **1971,** *54* (2), 724-728.

7. Hehre, W. J.; Ditchfield, R.; Pople, J. A., Self—Consistent Molecular Orbital Methods. XII. Further Extensions of Gaussian—Type Basis Sets for Use in Molecular Orbital Studies of Organic Molecules. *J. Chem. Phys.* **1972,** *56* (5), 2257-2261.

8. Hariharan, P. C.; Pople, J. A., The influence of polarization functions on molecular orbital hydrogenation energies. *Theor. Chim. Acta* **1973,** *28* (3), 213-222.

9. Hay, P. J.; Wadt, W. R., Ab initio effective core potentials for molecular calculations. Potentials for K to Au including the outermost core orbitals. *J. Chem. Phys.* **1985,** *82* (1), 299-310.

10. Roy, L. E.; Hay, P. J.; Martin, R. L., Revised Basis Sets for the LANL Effective Core Potentials. *J. Chem. Theory Comput.* **2008,** *4* (7), 1029-31.

11. Zhao, Y.; Truhlar, D. G., The M06 suite of density functionals for main group thermochemistry, thermochemical kinetics, noncovalent interactions, excited states, and transition elements: two new functionals and systematic testing of four M06-class functionals and 12 other functionals. *Theor. Chem. Acc.* **2008,** *120* (1-3), 215-241.

12. Krishnan, R.; Binkley, J. S.; Seeger, R.; Pople, J. A., Self‐consistent molecular orbital methods. XX. A basis set for correlated wave functions. *J. Chem. Phys.* **1980,** *72* (1), 650-654.

13. Dolg, M.; Wedig, U.; Stoll, H.; Preuss, H., Energy‐adjustedabinitiopseudopotentials for the first row transition elements. *J. Chem. Phys.* **1987,** *86* (2), 866-872.

14. Nicklass, A.; Dolg, M.; Stoll, H.; Preuss, H., Ab initioenergy‐adjusted pseudopotentials for the noble gases Ne through Xe: Calculation of atomic dipole and quadrupole polarizabilities. *J. Chem. Phys.* **1995,** *102* (22), 8942-8952.

15. Tomasi, J.; Mennucci, B.; Cammi, R., Quantum Mechanical Continuum Solvation Models. *Chem. Rev.* **2005,** *105* (8), 2999-3094.

16. Marenich, A. V.; Cramer, C. J.; Truhlar, D. G., Universal Solvation Model Based on Solute Electron Density and on a Continuum Model of the Solvent Defined by the Bulk Dielectric Constant and Atomic Surface Tensions. *J. Phys. Chem. B* **2009,** *113* (18), 6378-6396.

17. Grimme, S., Supramolecular binding thermodynamics by dispersion-corrected density functional theory. *Chemistry* **2012,** *18* (32), 9955-64.

18. Li, Y.-P.; Gomes, J.; Mallikarjun Sharada, S.; Bell, A. T.; Head-Gordon, M., Improved Force-Field Parameters for QM/MM Simulations of the Energies of Adsorption for Molecules in Zeolites and a Free Rotor Correction to the Rigid Rotor Harmonic Oscillator Model for Adsorption Enthalpies. *J. Phys. Chem. C* **2015,** *119* (4), 1840-1850.

19. Luchini, G. A.-R. J. V. G., Y.; Funes-Ardoiz, I.; Paton, R. S. (2019). GoodVibes v3.0.1. http://doi.org/10.5281/zenodo.595246.

20. Legault, C. Y. *CYLView, 1.0b*, Université de Sherbrooke, http://www.cylview.org: Canada, 2009.

21. Richter, J.M.; Whitefield, B.W.; Maimone, T.J.; Lin, D.W.; Castroviejo, P.M.; Baran, P.S. Scope and Mechanism of Direct Indole and Pyrrole Couplings Adjacent to Carbonyl Compounds: Total Synthesis of Acremoauxin A and Oxazinin 3. *J.Am.Chem.Soc*. **2007**, *42*, 12863.

22. Toutov1, A.A.; Liu1, W.B.; Betz1, K.N.; Fedorov, A.; Stoltz, B.M.; Grubbs, R.H. Silylation of C–H bonds in aromatic heterocycles by an Earth-abundant metal catalyst. *Nature*.**2015**, *518*, 80.

23. Maji, A.; Guin, S.; Feng, S.; Dahiya, A.; Singh, V.K.; Liu, P.; Maiti, D. Experimental and Computational Exploration of para‐Selective Silylation with a Hydrogen‐Bonded Template. *Angew.Chem.Int.Ed.* **2017**,*56*,14903.

24. Zhao, Z.; Snieckus, V. Directed ortho Metalation-Based Methodology. Halo-, Nitroso-, and Boro-Induced ipso-Desilylation. Link to an in situ Suzuki Reaction. *Org. Lett.* **2005**, *7*, 13

25. Lu, B.; Falck, J. R. Efficient Iridium‐Catalyzed C-H Functionalization/Silylation of Heteroarenes. *Angew.Chem.Int.Ed.* 2008, *47(39)*, 7508-7510.
