# Supplementary material for: Phosphorus(III)-assisted regioselective C–H silylation of heteroarenes
Source: Nat Commun. 2021 Jan 22;12:524. doi: 10.1038/s41467-020-20531-3 (PMC7822902; doi:10.1038/s41467-020-20531-3)
Supplement: Supplementary file 3 — Description of Additional Supplementary Files [file 41467_2020_20531_MOESM3_ESM.pdf]

Description of Additional Supplementary Files

File Name: Supplementary Data 1
Description: Crystallographic Data and Cartesian coordinates

File Name: Supplementary Data 2
Description: Crystallographic Data and Cartesian coordinates
