# Supplementary material for: Phosphorus(III)-assisted regioselective C–H silylation of heteroarenes
Source: Nat Commun. 2021 Jan 22;12:524. doi: 10.1038/s41467-020-20531-3 (PMC7822902; doi:10.1038/s41467-020-20531-3)
Supplement: Supplementary file 4 — Supplementary Data 1 [file 41467_2020_20531_MOESM4_ESM.zip › 256844_2_data_set_5092673_qjvtr8.docx]

**Supplementary Data 1**

**Crystallographic Data**

C(1)-C(3) 1.523(4)

C(1)-C(2) 1.524(4)

C(1)-C(4) 1.531(4)

C(1)-P(1) 1.890(2)

C(2)-H(2A) 0.9800

C(2)-H(2B) 0.9800

C(2)-H(2C) 0.9800

C(3)-H(3A) 0.9800

C(3)-H(3B) 0.9800

C(3)-H(3C) 0.9800

C(4)-H(4A) 0.9800

C(4)-H(4B) 0.9800

C(4)-H(4C) 0.9800

C(5)-C(7) 1.524(3)

C(5)-C(8) 1.539(3)

C(5)-C(6) 1.534(4)

C(5)-P(1) 1.885(2)

C(6)-H(6A) 0.9800

C(6)-H(6B) 0.9800

C(6)-H(6C) 0.9800

C(7)-H(7A) 0.9800

C(7)-H(7B) 0.9800

C(7)-H(7C) 0.9800

C(8)-H(8A) 0.9800

C(8)-H(8B) 0.9800

C(8)-H(8C) 0.9800

C(9)-C(10) 1.335(3)

C(9)-N(1) 1.406(3)

C(9)-H(9) 0.9500

C(10)-C(11) 1.419(3)

C(10)-C(17) 1.502(3)

C(11)-C(16) 1.389(3)

C(11)-C(12) 1.422(3)

C(12)-N(1) 1.410(3)

C(12)-C(13) 1.419(3)

C(13)-C(14) 1.403(3)

C(13)-Si(1) 1.901(2)

C(14)-C(15) 1.398(3)

C(14)-H(14) 0.9500

C(15)-C(16) 1.360(3)

C(15)-H(15) 0.9500

C(16)-H(16) 0.9500

C(17)-H(17A) 0.9800

C(17)-H(17B) 0.9800

C(17)-H(17C) 0.9800

C(18)-Si(1) 1.858(3)

C(18)-H(18A) 0.9800

C(18)-H(18B) 0.9800

C(18)-H(18C) 0.9800

C(19)-Si(1) 1.856(3)

C(19)-H(19A) 0.9800

C(19)-H(19B) 0.9800

C(19)-H(19C) 0.9800

C(20)-Si(1) 1.866(3)

C(20)-H(20A) 0.9800

C(20)-H(20B) 0.9800

C(20)-H(20C) 0.9800

N(1)-P(1) 1.7257(18)

C(3)-C(1)-C(2) 109.7(2)

C(3)-C(1)-C(4) 108.6(2)

C(2)-C(1)-C(4) 107.1(2)

C(3)-C(1)-P(1) 107.48(18)

C(2)-C(1)-P(1) 118.98(18)

C(4)-C(1)-P(1) 104.42(18)

C(1)-C(2)-H(2A) 109.5

C(1)-C(2)-H(2B) 109.5

H(2A)-C(2)-H(2B) 109.5

C(1)-C(2)-H(2C) 109.5

H(2A)-C(2)-H(2C) 109.5

H(2B)-C(2)-H(2C) 109.5

C(1)-C(3)-H(3A) 109.5

C(1)-C(3)-H(3B) 109.5

H(3A)-C(3)-H(3B) 109.5

C(1)-C(3)-H(3C) 109.5

H(3A)-C(3)-H(3C) 109.5

H(3B)-C(3)-H(3C) 109.5

C(1)-C(4)-H(4A) 109.5

C(1)-C(4)-H(4B) 109.5

H(4A)-C(4)-H(4B) 109.5

C(1)-C(4)-H(4C) 109.5

H(4A)-C(4)-H(4C) 109.5

H(4B)-C(4)-H(4C) 109.5

C(7)-C(5)-C(8) 108.6(2)

C(7)-C(5)-C(6) 109.1(2)

C(8)-C(5)-C(6) 107.5(2)

C(7)-C(5)-P(1) 120.18(19)

C(8)-C(5)-P(1) 105.02(15)

C(6)-C(5)-P(1) 105.77(16)

C(5)-C(6)-H(6A) 109.5

C(5)-C(6)-H(6B) 109.5

H(6A)-C(6)-H(6B) 109.5

C(5)-C(6)-H(6C) 109.5

H(6A)-C(6)-H(6C) 109.5

H(6B)-C(6)-H(6C) 109.5

C(5)-C(7)-H(7A) 109.5

C(5)-C(7)-H(7B) 109.5

H(7A)-C(7)-H(7B) 109.5

C(5)-C(7)-H(7C) 109.5

H(7A)-C(7)-H(7C) 109.5

H(7B)-C(7)-H(7C) 109.5

C(5)-C(8)-H(8A) 109.5

C(5)-C(8)-H(8B) 109.5

H(8A)-C(8)-H(8B) 109.5

C(5)-C(8)-H(8C) 109.5

H(8A)-C(8)-H(8C) 109.5

H(8B)-C(8)-H(8C) 109.5

C(10)-C(9)-N(1) 112.1(2)

C(10)-C(9)-H(9) 124.0

N(1)-C(9)-H(9) 124.0

C(9)-C(10)-C(11) 106.48(19)

C(9)-C(10)-C(17) 127.4(2)

C(11)-C(10)-C(17) 126.1(2)

C(16)-C(11)-C(10) 130.6(2)

C(16)-C(11)-C(12) 120.7(2)

C(10)-C(11)-C(12) 108.69(18)

N(1)-C(12)-C(13) 131.59(18)

N(1)-C(12)-C(11) 106.33(18)

C(13)-C(12)-C(11) 122.08(19)

C(14)-C(13)-C(12) 113.32(19)

C(14)-C(13)-Si(1) 113.68(16)

C(12)-C(13)-Si(1) 132.84(16)

C(15)-C(14)-C(13) 124.9(2)

C(15)-C(14)-H(14) 117.6

C(13)-C(14)-H(14) 117.6

C(16)-C(15)-C(14) 120.2(2)

C(16)-C(15)-H(15) 119.9

C(14)-C(15)-H(15) 119.9

C(15)-C(16)-C(11) 118.7(2)

C(15)-C(16)-H(16) 120.7

C(11)-C(16)-H(16) 120.7

C(10)-C(17)-H(17A) 109.5

C(10)-C(17)-H(17B) 109.5

H(17A)-C(17)-H(17B) 109.5

C(10)-C(17)-H(17C) 109.5

H(17A)-C(17)-H(17C) 109.5

H(17B)-C(17)-H(17C) 109.5

Si(1)-C(18)-H(18A) 109.5

Si(1)-C(18)-H(18B) 109.5

H(18A)-C(18)-H(18B) 109.5

Si(1)-C(18)-H(18C) 109.5

H(18A)-C(18)-H(18C) 109.5

H(18B)-C(18)-H(18C) 109.5

Si(1)-C(19)-H(19A) 109.5

Si(1)-C(19)-H(19B) 109.5

H(19A)-C(19)-H(19B) 109.5

Si(1)-C(19)-H(19C) 109.5

H(19A)-C(19)-H(19C) 109.5

H(19B)-C(19)-H(19C) 109.5

Si(1)-C(20)-H(20A) 109.5

Si(1)-C(20)-H(20B) 109.5

H(20A)-C(20)-H(20B) 109.5

Si(1)-C(20)-H(20C) 109.5

H(20A)-C(20)-H(20C) 109.5

H(20B)-C(20)-H(20C) 109.5

C(9)-N(1)-C(12) 106.41(17)

C(9)-N(1)-P(1) 126.93(15)

C(12)-N(1)-P(1) 126.18(14)

N(1)-P(1)-C(5) 102.65(10)

N(1)-P(1)-C(1) 103.26(10)

C(5)-P(1)-C(1) 110.48(11)

C(19)-Si(1)-C(18) 110.93(14)

C(19)-Si(1)-C(20) 104.24(16)

C(18)-Si(1)-C(20) 104.34(17)

C(19)-Si(1)-C(13) 116.14(12)

C(18)-Si(1)-C(13) 112.48(11)

C(20)-Si(1)-C(13) 107.61(11)

C(1)-C(2) 1.523(4)

C(1)-C(4) 1.529(3)

C(1)-C(3) 1.537(3)

C(1)-P(1) 1.840(2)

C(2)-H(2A) 0.9600

C(2)-H(2B) 0.9600

C(2)-H(2C) 0.9600

C(3)-H(3A) 0.9600

C(3)-H(3B) 0.9600

C(3)-H(3C) 0.9600

C(4)-H(4A) 0.9600

C(4)-H(4B) 0.9600

C(4)-H(4C) 0.9600

C(5)-C(6) 1.522(4)

C(5)-C(8) 1.525(5)

C(5)-C(7) 1.547(4)

C(5)-P(1) 1.830(3)

C(6)-H(6A) 0.9600

C(6)-H(6B) 0.9600

C(6)-H(6C) 0.9600

C(7)-H(7A) 0.9600

C(7)-H(7B) 0.9600

C(7)-H(7C) 0.9600

C(8)-H(8A) 0.9600

C(8)-H(8B) 0.9600

C(8)-H(8C) 0.9600

C(9)-C(10) 1.330(3)

C(9)-N(1) 1.407(3)

C(9)-H(9) 0.9300

C(10)-C(11) 1.420(3)

C(10)-H(10) 0.9300

C(11)-C(12) 1.395(3)

C(11)-C(16) 1.401(3)

C(12)-C(13) 1.365(3)

C(12)-H(12) 0.9300

C(13)-C(14) 1.385(3)

C(13)-H(13) 0.9300

C(14)-C(15) 1.379(3)

C(14)-H(14) 0.9300

C(15)-O(1) 1.354(3)

C(15)-C(16) 1.405(3)

C(16)-N(1) 1.420(3)

N(1)-P(1) 1.6985(19)

O(1)-H(1) 0.8200

O(2)-P(1) 1.4849(16)

C(2)-C(1)-C(4) 108.3(2)

C(2)-C(1)-C(3) 110.3(3)

C(4)-C(1)-C(3) 107.6(2)

C(2)-C(1)-P(1) 106.74(19)

C(4)-C(1)-P(1) 107.29(17)

C(3)-C(1)-P(1) 116.41(18)

C(1)-C(2)-H(2A) 109.5

C(1)-C(2)-H(2B) 109.5

H(2A)-C(2)-H(2B) 109.5

C(1)-C(2)-H(2C) 109.5

H(2A)-C(2)-H(2C) 109.5

H(2B)-C(2)-H(2C) 109.5

C(1)-C(3)-H(3A) 109.5

C(1)-C(3)-H(3B) 109.5

H(3A)-C(3)-H(3B) 109.5

C(1)-C(3)-H(3C) 109.5

H(3A)-C(3)-H(3C) 109.5

H(3B)-C(3)-H(3C) 109.5

C(1)-C(4)-H(4A) 109.5

C(1)-C(4)-H(4B) 109.5

H(4A)-C(4)-H(4B) 109.5

C(1)-C(4)-H(4C) 109.5

H(4A)-C(4)-H(4C) 109.5

H(4B)-C(4)-H(4C) 109.5

C(6)-C(5)-C(8) 111.4(3)

C(6)-C(5)-C(7) 108.6(3)

C(8)-C(5)-C(7) 108.0(3)

C(6)-C(5)-P(1) 115.0(2)

C(8)-C(5)-P(1) 109.0(2)

C(7)-C(5)-P(1) 104.5(2)

C(5)-C(6)-H(6A) 109.5

C(5)-C(6)-H(6B) 109.5

H(6A)-C(6)-H(6B) 109.5

C(5)-C(6)-H(6C) 109.5

H(6A)-C(6)-H(6C) 109.5

H(6B)-C(6)-H(6C) 109.5

C(5)-C(7)-H(7A) 109.5

C(5)-C(7)-H(7B) 109.5

H(7A)-C(7)-H(7B) 109.5

C(5)-C(7)-H(7C) 109.5

H(7A)-C(7)-H(7C) 109.5

H(7B)-C(7)-H(7C) 109.5

C(5)-C(8)-H(8A) 109.5

C(5)-C(8)-H(8B) 109.5

H(8A)-C(8)-H(8B) 109.5

C(5)-C(8)-H(8C) 109.5

H(8A)-C(8)-H(8C) 109.5

H(8B)-C(8)-H(8C) 109.5

C(10)-C(9)-N(1) 111.2(2)

C(10)-C(9)-H(9) 124.4

N(1)-C(9)-H(9) 124.4

C(9)-C(10)-C(11) 107.8(2)

C(9)-C(10)-H(10) 126.1

C(11)-C(10)-H(10) 126.1

C(12)-C(11)-C(16) 121.1(2)

C(12)-C(11)-C(10) 131.2(2)

C(16)-C(11)-C(10) 107.7(2)

C(13)-C(12)-C(11) 118.4(2)

C(13)-C(12)-H(12) 120.8

C(11)-C(12)-H(12) 120.8

C(12)-C(13)-C(14) 120.8(2)

C(12)-C(13)-H(13) 119.6

C(14)-C(13)-H(13) 119.6

C(15)-C(14)-C(13) 122.5(2)

C(15)-C(14)-H(14) 118.8

C(13)-C(14)-H(14) 118.8

O(1)-C(15)-C(14) 117.0(2)

O(1)-C(15)-C(16) 125.7(2)

C(14)-C(15)-C(16) 117.2(2)

C(11)-C(16)-C(15) 120.0(2)

C(11)-C(16)-N(1) 107.40(18)

C(15)-C(16)-N(1) 132.59(19)

C(9)-N(1)-C(16) 105.83(17)

C(9)-N(1)-P(1) 123.00(15)

C(16)-N(1)-P(1) 131.16(14)

C(15)-O(1)-H(1) 109.5

O(2)-P(1)-N(1) 109.31(9)

O(2)-P(1)-C(5) 109.39(11)

N(1)-P(1)-C(5) 105.74(13)

O(2)-P(1)-C(1) 108.52(10)

N(1)-P(1)-C(1) 106.58(10)

C(5)-P(1)-C(1) 117.06(13)

C(1)-C(8) 1.383(5)

C(1)-C(2) 1.388(5)

C(1)-Pd(1) 1.989(4)

C(2)-C(3) 1.436(7)

C(2)-H(2) 0.9300

C(3)-C(4) 1.347(8)

C(3)-H(3) 0.9300

C(4)-C(5) 1.397(7)

C(4)-H(4) 0.9300

C(5)-C(8) 1.397(5)

C(5)-C(6) 1.427(7)

C(6)-C(7) 1.348(6)

C(6)-H(6) 0.9300

C(7)-N(1) 1.400(5)

C(7)-H(7) 0.9300

C(8)-N(1) 1.378(5)

C(9)-C(12) 1.523(5)

C(9)-C(10) 1.522(5)

C(9)-C(11) 1.527(5)

C(9)-P(1) 1.867(3)

C(10)-H(10A) 0.9600

C(10)-H(10B) 0.9600

C(10)-H(10C) 0.9600

C(11)-H(11A) 0.9600

C(11)-H(11B) 0.9600

C(11)-H(11C) 0.9600

C(12)-H(12A) 0.9600

C(12)-H(12B) 0.9600

C(12)-H(12C) 0.9600

C(13)-C(15) 1.527(6)

C(13)-C(16) 1.529(5)

C(13)-C(14) 1.534(5)

C(13)-P(1) 1.859(4)

C(14)-H(14A) 0.9600

C(14)-H(14B) 0.9600

C(14)-H(14C) 0.9600

C(15)-H(15A) 0.9600

C(15)-H(15B) 0.9600

C(15)-H(15C) 0.9600

C(16)-H(16A) 0.9600

C(16)-H(16B) 0.9600

C(16)-H(16C) 0.9600

C(17)-C(18) 1.517(5)

C(17)-H(17A) 0.9600

C(17)-H(17B) 0.9600

C(17)-H(17C) 0.9600

C(18)-O(1) 1.247(4)

C(18)-O(2) 1.247(5)

C(19)-C(20) 1.515(6)

C(19)-H(19A) 0.9600

C(19)-H(19B) 0.9600

C(19)-H(19C) 0.9600

C(20)-O(4) 1.238(5)

C(20)-O(3) 1.243(5)

C(21)-C(24) 1.530(5)

C(21)-H(21A) 0.9600

C(21)-H(21B) 0.9600

C(21)-H(21C) 0.9600

C(22)-C(24) 1.535(5)

C(22)-H(22A) 0.9600

C(22)-H(22B) 0.9600

C(22)-H(22C) 0.9600

C(23)-C(24) 1.536(5)

C(23)-H(23A) 0.9600

C(23)-H(23B) 0.9600

C(23)-H(23C) 0.9600

C(24)-P(2) 1.862(3)

C(25)-C(28) 1.526(5)

C(25)-H(25A) 0.9600

C(25)-H(25B) 0.9600

C(25)-H(25C) 0.9600

C(26)-C(28) 1.534(5)

C(26)-H(26A) 0.9600

C(26)-H(26B) 0.9600

C(26)-H(26C) 0.9600

C(27)-C(28) 1.535(5)

C(27)-H(27A) 0.9600

C(27)-H(27B) 0.9600

C(27)-H(27C) 0.9600

C(28)-P(2) 1.862(3)

C(29)-C(30) 1.384(5)

C(29)-C(36) 1.389(5)

C(29)-Pd(2) 1.984(4)

C(30)-C(31) 1.406(6)

C(30)-H(30) 0.9300

C(31)-C(32) 1.354(7)

C(31)-H(31) 0.9300

C(32)-C(33) 1.417(6)

C(32)-H(32) 0.9300

C(33)-C(36) 1.395(5)

C(33)-C(34) 1.425(6)

C(34)-C(35) 1.347(6)

C(34)-H(34) 0.9300

C(35)-N(2) 1.402(4)

C(35)-H(35) 0.9300

C(36)-N(2) 1.380(4)

N(1)-P(1) 1.709(3)

N(2)-P(2) 1.711(3)

O(1)-Pd(1) 2.098(2)

O(2)-Pd(2) 2.114(3)

O(3)-Pd(1) 2.126(3)

O(4)-Pd(2) 2.076(3)

P(1)-Pd(1) 2.2592(10)

P(2)-Pd(2) 2.2453(11)

C(8)-C(1)-C(2) 113.7(4)

C(8)-C(1)-Pd(1) 117.9(3)

C(2)-C(1)-Pd(1) 128.5(3)

C(1)-C(2)-C(3) 119.8(5)

C(1)-C(2)-H(2) 120.1

C(3)-C(2)-H(2) 120.1

C(4)-C(3)-C(2) 123.6(4)

C(4)-C(3)-H(3) 118.2

C(2)-C(3)-H(3) 118.2

C(3)-C(4)-C(5) 118.6(5)

C(3)-C(4)-H(4) 120.7

C(5)-C(4)-H(4) 120.7

C(8)-C(5)-C(4) 116.1(5)

C(8)-C(5)-C(6) 106.0(4)

C(4)-C(5)-C(6) 137.8(5)

C(7)-C(6)-C(5) 108.6(4)

C(7)-C(6)-H(6) 125.7

C(5)-C(6)-H(6) 125.7

C(6)-C(7)-N(1) 108.9(4)

C(6)-C(7)-H(7) 125.5

N(1)-C(7)-H(7) 125.5

N(1)-C(8)-C(1) 123.0(3)

N(1)-C(8)-C(5) 108.8(4)

C(1)-C(8)-C(5) 128.2(4)

C(12)-C(9)-C(10) 109.1(3)

C(12)-C(9)-C(11) 110.2(3)

C(10)-C(9)-C(11) 108.5(3)

C(12)-C(9)-P(1) 108.8(2)

C(10)-C(9)-P(1) 105.4(2)

C(11)-C(9)-P(1) 114.7(3)

C(9)-C(10)-H(10A) 109.5

C(9)-C(10)-H(10B) 109.5

H(10A)-C(10)-H(10B) 109.5

C(9)-C(10)-H(10C) 109.5

H(10A)-C(10)-H(10C) 109.5

H(10B)-C(10)-H(10C) 109.5

C(9)-C(11)-H(11A) 109.5

C(9)-C(11)-H(11B) 109.5

H(11A)-C(11)-H(11B) 109.5

C(9)-C(11)-H(11C) 109.5

H(11A)-C(11)-H(11C) 109.5

H(11B)-C(11)-H(11C) 109.5

C(9)-C(12)-H(12A) 109.5

C(9)-C(12)-H(12B) 109.5

H(12A)-C(12)-H(12B) 109.5

C(9)-C(12)-H(12C) 109.5

H(12A)-C(12)-H(12C) 109.5

H(12B)-C(12)-H(12C) 109.5

C(15)-C(13)-C(16) 109.3(4)

C(15)-C(13)-C(14) 109.3(3)

C(16)-C(13)-C(14) 109.4(4)

C(15)-C(13)-P(1) 108.7(3)

C(16)-C(13)-P(1) 115.5(3)

C(14)-C(13)-P(1) 104.4(3)

C(13)-C(14)-H(14A) 109.5

C(13)-C(14)-H(14B) 109.5

H(14A)-C(14)-H(14B) 109.5

C(13)-C(14)-H(14C) 109.5

H(14A)-C(14)-H(14C) 109.5

H(14B)-C(14)-H(14C) 109.5

C(13)-C(15)-H(15A) 109.5

C(13)-C(15)-H(15B) 109.5

H(15A)-C(15)-H(15B) 109.5

C(13)-C(15)-H(15C) 109.5

H(15A)-C(15)-H(15C) 109.5

H(15B)-C(15)-H(15C) 109.5

C(13)-C(16)-H(16A) 109.5

C(13)-C(16)-H(16B) 109.5

H(16A)-C(16)-H(16B) 109.5

C(13)-C(16)-H(16C) 109.5

H(16A)-C(16)-H(16C) 109.5

H(16B)-C(16)-H(16C) 109.5

C(18)-C(17)-H(17A) 109.5

C(18)-C(17)-H(17B) 109.5

H(17A)-C(17)-H(17B) 109.5

C(18)-C(17)-H(17C) 109.5

H(17A)-C(17)-H(17C) 109.5

H(17B)-C(17)-H(17C) 109.5

O(1)-C(18)-O(2) 127.2(3)

O(1)-C(18)-C(17) 116.6(4)

O(2)-C(18)-C(17) 116.2(3)

C(20)-C(19)-H(19A) 109.5

C(20)-C(19)-H(19B) 109.5

H(19A)-C(19)-H(19B) 109.5

C(20)-C(19)-H(19C) 109.5

H(19A)-C(19)-H(19C) 109.5

H(19B)-C(19)-H(19C) 109.5

O(4)-C(20)-O(3) 126.4(4)

O(4)-C(20)-C(19) 116.3(4)

O(3)-C(20)-C(19) 117.3(4)

C(24)-C(21)-H(21A) 109.5

C(24)-C(21)-H(21B) 109.5

H(21A)-C(21)-H(21B) 109.5

C(24)-C(21)-H(21C) 109.5

H(21A)-C(21)-H(21C) 109.5

H(21B)-C(21)-H(21C) 109.5

C(24)-C(22)-H(22A) 109.5

C(24)-C(22)-H(22B) 109.5

H(22A)-C(22)-H(22B) 109.5

C(24)-C(22)-H(22C) 109.5

H(22A)-C(22)-H(22C) 109.5

H(22B)-C(22)-H(22C) 109.5

C(24)-C(23)-H(23A) 109.5

C(24)-C(23)-H(23B) 109.5

H(23A)-C(23)-H(23B) 109.5

C(24)-C(23)-H(23C) 109.5

H(23A)-C(23)-H(23C) 109.5

H(23B)-C(23)-H(23C) 109.5

C(21)-C(24)-C(22) 108.2(3)

C(21)-C(24)-C(23) 109.1(3)

C(22)-C(24)-C(23) 109.8(3)

C(21)-C(24)-P(2) 109.8(2)

C(22)-C(24)-P(2) 104.8(2)

C(23)-C(24)-P(2) 114.9(2)

C(28)-C(25)-H(25A) 109.5

C(28)-C(25)-H(25B) 109.5

H(25A)-C(25)-H(25B) 109.5

C(28)-C(25)-H(25C) 109.5

H(25A)-C(25)-H(25C) 109.5

H(25B)-C(25)-H(25C) 109.5

C(28)-C(26)-H(26A) 109.5

C(28)-C(26)-H(26B) 109.5

H(26A)-C(26)-H(26B) 109.5

C(28)-C(26)-H(26C) 109.5

H(26A)-C(26)-H(26C) 109.5

H(26B)-C(26)-H(26C) 109.5

C(28)-C(27)-H(27A) 109.5

C(28)-C(27)-H(27B) 109.5

H(27A)-C(27)-H(27B) 109.5

C(28)-C(27)-H(27C) 109.5

H(27A)-C(27)-H(27C) 109.5

H(27B)-C(27)-H(27C) 109.5

C(25)-C(28)-C(26) 111.1(3)

C(25)-C(28)-C(27) 109.0(3)

C(26)-C(28)-C(27) 107.9(3)

C(25)-C(28)-P(2) 109.1(3)

C(26)-C(28)-P(2) 113.8(2)

C(27)-C(28)-P(2) 105.6(2)

C(30)-C(29)-C(36) 113.4(4)

C(30)-C(29)-Pd(2) 129.5(3)

C(36)-C(29)-Pd(2) 117.0(2)

C(29)-C(30)-C(31) 121.3(4)

C(29)-C(30)-H(30) 119.4

C(31)-C(30)-H(30) 119.4

C(32)-C(31)-C(30) 123.5(4)

C(32)-C(31)-H(31) 118.3

C(30)-C(31)-H(31) 118.3

C(31)-C(32)-C(33) 118.1(4)

C(31)-C(32)-H(32) 121.0

C(33)-C(32)-H(32) 121.0

C(36)-C(33)-C(32) 116.0(4)

C(36)-C(33)-C(34) 105.6(3)

C(32)-C(33)-C(34) 138.4(4)

C(35)-C(34)-C(33) 108.6(3)

C(35)-C(34)-H(34) 125.7

C(33)-C(34)-H(34) 125.7

C(34)-C(35)-N(2) 109.5(4)

C(34)-C(35)-H(35) 125.2

N(2)-C(35)-H(35) 125.2

N(2)-C(36)-C(33) 109.7(3)

N(2)-C(36)-C(29) 122.6(3)

C(33)-C(36)-C(29) 127.7(4)

C(8)-N(1)-C(7) 107.7(3)

C(8)-N(1)-P(1) 112.5(2)

C(7)-N(1)-P(1) 139.1(3)

C(36)-N(2)-C(35) 106.6(3)

C(36)-N(2)-P(2) 113.4(2)

C(35)-N(2)-P(2) 140.0(3)

C(18)-O(1)-Pd(1) 139.2(3)

C(18)-O(2)-Pd(2) 126.5(2)

C(20)-O(3)-Pd(1) 133.0(3)

C(20)-O(4)-Pd(2) 131.8(3)

N(1)-P(1)-C(13) 104.93(17)

N(1)-P(1)-C(9) 104.79(16)

C(13)-P(1)-C(9) 114.34(16)

N(1)-P(1)-Pd(1) 102.95(11)

C(13)-P(1)-Pd(1) 116.18(12)

C(9)-P(1)-Pd(1) 111.96(11)

N(2)-P(2)-C(24) 105.79(15)

N(2)-P(2)-C(28) 104.91(15)

C(24)-P(2)-C(28) 113.34(16)

N(2)-P(2)-Pd(2) 102.26(10)

C(24)-P(2)-Pd(2) 116.36(11)

C(28)-P(2)-Pd(2) 112.57(12)

C(1)-Pd(1)-O(1) 90.81(15)

C(1)-Pd(1)-O(3) 173.29(13)

O(1)-Pd(1)-O(3) 86.96(13)

C(1)-Pd(1)-P(1) 82.80(11)

O(1)-Pd(1)-P(1) 169.33(8)

O(3)-Pd(1)-P(1) 98.41(8)

C(29)-Pd(2)-O(4) 92.52(15)

C(29)-Pd(2)-O(2) 176.15(12)

O(4)-Pd(2)-O(2) 85.30(13)

C(29)-Pd(2)-P(2) 84.00(11)

O(4)-Pd(2)-P(2) 170.15(8)

O(2)-Pd(2)-P(2) 97.63(8)
