# Supplementary material for: Phosphorus(III)-assisted regioselective C–H silylation of heteroarenes
Source: Nat Commun. 2021 Jan 22;12:524. doi: 10.1038/s41467-020-20531-3 (PMC7822902; doi:10.1038/s41467-020-20531-3)
Supplement: Supplementary file 5 — Supplementary Data 2 [file 41467_2020_20531_MOESM5_ESM.zip › 256844_2_data_set_5092674_qjvtr8.docx]

**Supplementary Data 2**

**Cartesian coordinates**

**1a** Eopt -1020.317485

C -3.604459 0.350748 -1.679091

H -3.972244 0.579162 -2.675868

C -4.525324 0.094776 -0.643139

H -5.590553 0.130423 -0.855359

C -4.087306 -0.201461 0.640942

H -4.797688 -0.399148 1.439711

C -2.706665 -0.243631 0.896921

C -1.921364 -0.510794 2.069181

H -2.291193 -0.750828 3.057225

C -0.608886 -0.401042 1.709659

H 0.273497 -0.527123 2.315856

C -1.792607 0.019795 -0.158209

C 1.791138 1.615586 0.095425

C 0.916522 2.794763 -0.390383

H -0.108965 2.715228 -0.012660

H 0.873140 2.844458 -1.483882

H 1.336660 3.741204 -0.024535

C 1.896026 1.697132 1.628139

H 2.402857 2.632197 1.904127

H 2.472328 0.875609 2.060514

H 0.908436 1.713220 2.096858

C 3.193992 1.743772 -0.530465

H 3.167737 1.649143 -1.622681

H 3.890607 0.995921 -0.137993

H 3.609563 2.732149 -0.294860

C 1.921122 -1.516679 -0.296889

C 0.898962 -2.670237 -0.390240

H 0.309904 -2.618395 -1.313349

H 0.202376 -2.668598 0.453150

H 1.433832 -3.629071 -0.388584

C 2.925124 -1.660977 -1.464716

H 3.683925 -0.872061 -1.462257

H 2.418661 -1.640995 -2.435638

H 3.450383 -2.621310 -1.378247

C 2.689432 -1.621882 1.030669

H 3.192624 -2.597285 1.082287

H 2.032262 -1.554156 1.901698

H 3.462432 -0.853409 1.120858

N -0.489563 -0.073441 0.354348

P 0.938112 0.085043 -0.664133

C -2.232413 0.318027 -1.453279

H -1.525083 0.514742 -2.251949

**2a** Eopt -1428.998402

C -2.159067 0.155512 0.037873

C -3.454925 0.688907 0.179444

H -4.281054 0.016252 0.378968

C -3.770188 2.053221 0.055592

H -4.800853 2.375187 0.179330

C -2.781591 2.971343 -0.248636

H -3.006718 4.026822 -0.379553

C -1.465452 2.510619 -0.383538

C -0.249925 3.207495 -0.683728

H -0.146267 4.263736 -0.893605

C 0.749132 2.284987 -0.660981

H 1.805767 2.417548 -0.826331

C -1.151312 1.127368 -0.210218

C 2.493940 -0.530141 -1.346373

C 1.741271 -0.283540 -2.671141

H 1.452773 0.764590 -2.788952

H 0.834142 -0.890638 -2.748969

H 2.396057 -0.552439 -3.510629

C 3.744941 0.365462 -1.305187

H 4.372696 0.143182 -2.178927

H 4.353537 0.184028 -0.414932

H 3.506636 1.431872 -1.346092

C 2.956254 -2.006931 -1.313253

H 2.113900 -2.697817 -1.421880

H 3.479248 -2.257787 -0.384432

H 3.651215 -2.192851 -2.142762

C 2.048401 0.183175 1.697900

C 0.892891 0.235783 2.724031

H 0.406188 -0.735635 2.847297

H 0.128420 0.963915 2.431789

H 1.288710 0.540059 3.702228

C 3.036731 -0.926019 2.112700

H 3.929566 -0.943650 1.478630

H 2.574220 -1.919918 2.079340

H 3.372108 -0.752536 3.143546

C 2.757747 1.548979 1.722752

H 3.182796 1.709446 2.723173

H 2.058517 2.367117 1.531185

H 3.578076 1.620364 1.006416

N 0.249034 1.012670 -0.367216

P 1.207997 -0.392890 0.075908

Si -2.098314 -1.755841 0.035161

C -1.293823 -2.447546 -1.537915

H -0.207826 -2.520423 -1.449769

H -1.685539 -3.452974 -1.736893

H -1.526764 -1.822851 -2.408298

C -1.354873 -2.554629 1.586191

H -0.262894 -2.549152 1.577825

H -1.696868 -2.044299 2.494394

H -1.692108 -3.597340 1.647337

C -3.889988 -2.392725 -0.015971

H -4.453876 -2.004569 -0.871642

H -3.856675 -3.485591 -0.110860

H -4.454907 -2.165145 0.895469

**Cat_3Pd** Eopt -1751.367846

Pd 0.024815 1.846282 0.008746

C 2.313023 1.197100 1.860948

O 1.648547 2.076669 1.238465

O 2.323629 -0.053857 1.655503

C 0.002076 -2.582913 -1.891274

O -1.067645 -2.439299 -1.229149

O 1.110795 -1.998841 -1.702509

C -0.062485 -3.568995 -3.039286

H 0.909849 -3.665716 -3.523871

H -0.390576 -4.540442 -2.656608

H -0.812701 -3.227448 -3.759399

C 3.226490 1.706157 2.957055

H 2.738721 2.516556 3.503412

H 3.505358 0.893760 3.630235

H 4.134424 2.106617 2.491507

Pd -1.621704 -0.907024 0.013392

C -0.079433 -2.597163 1.871223

O 0.994165 -2.481977 1.209702

O -1.169625 -1.977779 1.687428

C 2.241288 1.324192 -1.870826

O 1.176391 1.982083 -1.667848

O 2.649043 0.302206 -1.244449

C 3.148697 1.840418 -2.968556

H 3.634093 1.005352 -3.478103

H 2.582855 2.452628 -3.673058

H 3.926067 2.462529 -2.509917

C -0.045002 -3.592932 3.012171

H 0.712113 -3.276808 3.736690

H -1.020918 -3.666372 3.493628

H 0.257739 -4.570209 2.623573

Pd 1.592716 -0.955979 -0.019643

C -2.274814 1.280104 -1.853186

O -1.591179 2.136094 -1.217667

O -2.326558 0.029523 -1.654439

C -3.139114 1.827094 -2.970732

H -2.519707 2.419137 -3.650551

H -3.634032 1.017842 -3.509181

H -3.890549 2.496396 -2.538622

C -2.205159 1.373237 1.884899

O -1.122801 2.003148 1.686044

O -2.641420 0.367876 1.250787

C -3.097105 1.903709 2.988454

H -3.872185 2.531838 2.534268

H -3.587385 1.075317 3.504405

H -2.518699 2.511346 3.686567

**DMBQ** Eopt -460.110113

C -1.240673 0.722232 0.000000

C 0.060660 1.426416 0.000000

C 1.240673 0.777326 0.000000

C 1.240673 -0.722232 0.000000

C -0.060660 -1.426416 0.000000

C -1.240673 -0.777326 0.000000

H -0.002413 2.511676 0.000000

H 0.002413 -2.511676 0.000000

O -2.296245 1.347101 0.000000

O 2.296245 -1.347101 0.000000

C -2.580182 -1.449488 0.000000

H -3.162422 -1.140779 0.875691

H -3.162422 -1.140779 -0.875691

H -2.480277 -2.538117 0.000000

C 2.580182 1.449488 0.000000

H 3.162422 1.140779 0.875691

H 3.162422 1.140779 -0.875691

H 2.480277 2.538117 0.000000

**24** Eopt -870.060151

C -2.419301 1.673433 0.000000

C -1.268502 2.463283 0.000000

C 0.008486 1.896125 0.000000

C 0.109713 0.495729 0.000000

C -1.046742 -0.291300 0.000000

C -2.323962 0.275380 0.000000

H -1.364634 3.548742 0.000000

H -0.961705 -1.373440 0.000000

C -3.574223 -0.566146 0.000000

H -3.333443 -1.633939 0.000000

H -4.195030 -0.351723 0.878244

H -4.195030 -0.351723 -0.878244

C 1.256603 2.739913 0.000000

H 1.877641 2.524808 0.877696

H 1.877641 2.524808 -0.877696

H 1.015121 3.807586 0.000000

O -3.686699 2.211393 0.000000

H -3.613823 3.177789 0.000000

O 1.377699 -0.026012 0.000000

Si 1.948300 -1.612125 0.000000

C 1.377699 -2.509725 1.555489

H 0.288296 -2.611189 1.605008

H 1.809513 -3.517442 1.600751

H 1.700545 -1.967700 2.452056

C 1.377699 -2.509725 -1.555489

H 0.288296 -2.611189 -1.605008

H 1.700545 -1.967700 -2.452056

H 1.809513 -3.517442 -1.600751

C 3.814990 -1.413528 0.000000

H 4.151006 -0.862352 0.885531

H 4.315601 -2.389475 0.000000

H 4.151006 -0.862352 -0.885531

**HOAC** Eopt -229.084787

C 0.093105 0.127087 -0.000398

C -1.396586 -0.109451 -0.000042

H -1.681533 -0.685683 0.886374

H -1.916822 0.848495 -0.006811

H -1.680731 -0.698603 -0.878078

O 0.645914 1.203399 0.000080

O 0.776229 -1.048228 -0.000023

H 1.722830 -0.811399 0.000699

**INT1_A** Eopt -1604.100753

C 2.645207 1.981929 1.942890

H 2.277310 2.847157 2.486858

C 4.031860 1.756722 1.846257

H 4.721418 2.456991 2.309681

C 4.523040 0.641697 1.180177

H 5.591458 0.452893 1.117530

C 3.614396 -0.252042 0.590644

C 3.781301 -1.488218 -0.124125

H 4.715677 -1.981038 -0.356663

C 2.541057 -1.940150 -0.452794

H 2.262462 -2.838633 -0.977413

C 2.221541 0.004181 0.674054

C -0.685567 -2.218093 1.313177

C -0.433441 -1.323635 2.548037

H 0.632310 -1.114872 2.679945

H -0.960708 -0.369679 2.506415

H -0.777836 -1.865667 3.437870

C 0.165767 -3.494060 1.475209

H -0.105904 -3.963484 2.429049

H -0.010150 -4.231505 0.690380

H 1.235528 -3.268480 1.513046

C -2.181427 -2.571974 1.212691

H -2.791765 -1.681999 1.039883

H -2.379837 -3.300667 0.419787

H -2.495870 -3.030233 2.158858

C -0.473161 -2.091367 -1.863474

C 0.437972 -1.450043 -2.935000

H 0.360367 -0.360000 -2.941617

H 1.490875 -1.708044 -2.801244

H 0.121848 -1.824384 -3.916856

C -1.946812 -1.793339 -2.241705

H -2.659029 -2.138819 -1.488164

H -2.116296 -0.723618 -2.396134

H -2.172754 -2.312969 -3.181196

C -0.282469 -3.620642 -1.852705

H -0.433795 -3.990532 -2.874408

H 0.715551 -3.934836 -1.538471

H -1.015229 -4.120968 -1.214172

C 0.794050 3.873158 -2.479316

H 1.263668 3.469094 -3.380645

H 0.135790 4.707996 -2.727707

H 1.591899 4.227790 -1.816492

C 0.023695 2.795543 -1.770575

C -4.164746 1.184954 2.284122

H -4.459508 0.150014 2.490832

H -4.169664 1.762493 3.210210

H -4.893827 1.598457 1.580114

C -2.774743 1.212155 1.666295

N 1.553363 -1.046758 0.001203

O -1.106067 3.005884 -1.248684

O -2.712713 0.546231 0.534421

O -1.825217 1.783171 2.200632

P -0.161163 -1.202190 -0.201025

Pd -0.987805 0.903944 -0.474442

O 0.555717 1.621894 -1.664611

C 1.721827 1.111875 1.370322

H 0.659077 1.296918 1.492844

**INT2_A** Eopt -1374.997929

C 0.271086 1.870719 0.075731

C 0.116493 3.251606 0.026743

H -0.876706 3.691386 -0.009064

C 1.257330 4.096667 0.015133

H 1.101588 5.172225 -0.020645

C 2.559758 3.604105 0.037079

H 3.407302 4.283792 0.012248

C 2.748370 2.207771 0.081298

C 3.853909 1.277232 0.073045

H 4.908588 1.520577 0.051096

C 3.340004 0.004971 0.103293

H 3.852838 -0.945336 0.110400

C 1.588751 1.415173 0.118246

C 0.739424 -1.853876 -1.651664

C 1.090769 -0.746553 -2.670912

H 2.073493 -0.306374 -2.476382

H 0.347937 0.057679 -2.665668

H 1.106838 -1.188514 -3.674767

C 1.828488 -2.939704 -1.677243

H 1.927249 -3.320829 -2.701563

H 1.577569 -3.789655 -1.035823

H 2.806505 -2.548790 -1.377330

C -0.630273 -2.462942 -2.024043

H -1.430824 -1.717192 -1.996861

H -0.916786 -3.284386 -1.362425

H -0.568766 -2.864610 -3.043295

C 0.625657 -2.098119 1.523580

C 0.313205 -1.178477 2.727043

H -0.696432 -0.760703 2.663977

H 1.022135 -0.346573 2.796844

H 0.385255 -1.767008 3.650391

C -0.484017 -3.158382 1.390095

H -0.243673 -3.909123 0.630729

H -1.451702 -2.705884 1.144425

H -0.590958 -3.682846 2.347637

C 1.998044 -2.758468 1.753869

H 1.941993 -3.374503 2.660267

H 2.778796 -2.009224 1.915001

H 2.302354 -3.411431 0.933078

C -5.231069 0.526894 -0.029392

H -5.584475 0.614269 1.005009

H -5.633074 -0.394531 -0.457006

H -5.589786 1.398170 -0.583257

C -3.721989 0.492314 -0.030226

N 1.942042 0.069293 0.152588

O -3.109095 -0.626074 -0.034069

O -3.069484 1.588832 0.000442

P 0.571548 -0.971876 0.012131

Pd -1.179995 0.492931 0.031563

**INT2_B** Eopt -1374.979873

C 2.539116 -0.769595 -0.122628

C 2.455974 -2.194490 -0.092547

C 3.651283 -2.929846 -0.033526

C 4.867758 -2.254106 0.009779

C 4.921766 -0.847945 -0.008643

C 3.755533 -0.086868 -0.078348

C 1.059820 -2.572742 -0.117501

H 3.621917 -4.016100 -0.015527

H 5.793742 -2.820552 0.061012

H 5.884635 -0.346327 0.031424

H 3.796004 0.997951 -0.099355

H 0.669256 -3.580147 -0.083701

C 0.328408 -1.417301 -0.168189

N 1.223328 -0.320191 -0.202907

C 0.303396 2.109681 -1.500273

C -0.225366 1.302255 -2.706339

H -0.090312 1.898481 -3.617560

H 0.318886 0.360662 -2.829689

H -1.289819 1.069317 -2.602082

C -0.565483 3.365853 -1.304629

H -0.181523 4.020696 -0.516510

H -0.569753 3.943082 -2.237383

H -1.604554 3.108506 -1.069694

C 1.775667 2.482627 -1.759986

H 2.385953 1.591437 -1.934017

H 1.827679 3.102468 -2.664100

H 2.220307 3.056497 -0.943439

C 0.405931 1.792804 1.667142

C 0.713644 0.643472 2.651843

H 1.659621 0.146991 2.415831

H 0.787758 1.061842 3.663130

H -0.077813 -0.112828 2.653932

C 1.566096 2.803629 1.685353

H 2.514138 2.341211 1.392134

H 1.380761 3.665880 1.038519

H 1.687593 3.182394 2.708060

C -0.918553 2.472495 2.078356

H -1.753215 1.763471 2.066367

H -0.814325 2.861689 3.098790

H -1.178324 3.313979 1.429726

P 0.123878 0.974135 -0.008943

Pd -1.508240 -0.623029 -0.054321

O -3.120321 -2.118899 -0.052900

C -3.981896 -1.183893 0.037147

O -3.603684 0.034129 0.089699

C -5.452707 -1.519840 0.048251

H -5.621200 -2.456063 0.586646

H -6.025996 -0.706717 0.499372

H -5.794084 -1.658174 -0.984566

**INT2_dimmer_A** Eopt -2750.070608

C 1.920443 -2.026019 -0.065884

C 1.611468 -3.355686 0.206025

H 1.108695 -3.615631 1.131327

C 1.939894 -4.381999 -0.716305

H 1.662509 -5.404854 -0.471368

C 2.611334 -4.130341 -1.909252

H 2.862924 -4.941773 -2.587090

C 2.983447 -2.801861 -2.195954

C 3.736938 -2.112332 -3.215742

H 4.185188 -2.547730 -4.099792

C 3.792602 -0.785007 -2.873928

H 4.273172 0.034942 -3.385161

C 2.601930 -1.814242 -1.267833

C 4.647727 0.890024 0.238029

C 5.152013 -0.532988 0.569276

H 5.278364 -1.143708 -0.330293

H 4.470924 -1.058756 1.245565

H 6.128009 -0.449942 1.063556

C 5.644499 1.565782 -0.719779

H 6.646458 1.521156 -0.274195

H 5.408173 2.621056 -0.879715

H 5.696438 1.063501 -1.691373

C 4.540733 1.705249 1.544194

H 3.823655 1.269962 2.244562

H 4.238051 2.739897 1.360983

H 5.528506 1.727514 2.022160

C 2.217129 2.166064 -1.390775

C 0.832722 1.721020 -1.899791

H 0.198608 1.374582 -1.085022

H 0.919004 0.923266 -2.643673

H 0.324393 2.574741 -2.362605

C 2.057293 3.309154 -0.368214

H 3.021434 3.763865 -0.116025

H 1.578218 2.964796 0.552102

H 1.420932 4.086772 -0.805709

C 3.042564 2.641815 -2.601326

H 2.515504 3.490677 -3.055354

H 3.123188 1.867693 -3.369779

H 4.044585 2.983528 -2.337546

C -0.747780 -2.874390 4.023052

H 0.186038 -3.370234 4.294158

H -1.508800 -3.612244 3.752365

H -1.123443 -2.315401 4.887764

C -0.538663 -1.903640 2.869929

C 0.622470 2.599398 4.201482

H -0.305241 2.950051 4.656662

H 1.239546 3.451308 3.897836

H 1.198517 2.023344 4.933945

C 0.345857 1.719933 2.990998

C -1.670935 -3.106762 -0.586601

H -1.045564 -3.822573 -1.131720

H -2.514225 -3.651462 -0.154728

H -1.070412 -2.699124 0.225642

C -0.871715 -1.374645 -2.214514

H -0.217419 -0.934538 -1.460485

H -1.132050 -0.606063 -2.948900

H -0.298195 -2.160664 -2.719876

C -3.018847 -2.618845 -2.655475

H -2.422143 -3.339491 -3.228873

H -3.397516 -1.874509 -3.362710

H -3.866958 -3.163959 -2.231212

C -2.122010 -2.004494 -1.566662

C -4.685253 -2.273333 0.842735

H -3.889442 -2.259115 1.595781

H -4.566655 -3.167126 0.221916

H -5.647295 -2.362121 1.363106

C -5.706693 -1.094713 -1.160979

H -5.486673 -1.917160 -1.844707

H -5.759529 -0.165639 -1.736558

H -6.702739 -1.278266 -0.738264

C -5.105485 0.209175 0.888218

H -6.146044 0.068793 1.208017

H -5.040175 1.160698 0.350112

H -4.477760 0.282185 1.781563

C -4.695617 -0.985463 -0.004059

C -1.930049 2.041436 0.059908

C -1.482711 3.324857 0.362136

H -0.990004 3.519607 1.308217

C -1.646733 4.387686 -0.562748

H -1.273778 5.372302 -0.289299

C -2.263544 4.217945 -1.799497

H -2.369904 5.053566 -2.486244

C -2.752798 2.938224 -2.129819

C -3.430757 2.318296 -3.242915

H -3.739120 2.794913 -4.164691

C -3.624335 0.995275 -2.934613

H -4.101939 0.219050 -3.512924

C -2.565279 1.917007 -1.178584

N 3.074217 -0.574543 -1.691894

N -3.095773 0.727491 -1.668578

O 0.659403 -1.606663 2.590830

O -1.588098 -1.482177 2.300834

O 1.359814 1.370570 2.318029

O -0.863145 1.427718 2.763465

P 2.907933 0.660755 -0.486668

P -2.930674 -0.610941 -0.579233

Pd 1.546618 -0.402815 1.055518

Pd -1.692073 0.348802 1.116802

**INT3_A** Eopt -2193.567506

C 0.387878 1.752059 -0.529335

C 1.243054 2.786691 -0.919794

H 2.289086 2.581282 -1.090966

C 0.806055 4.123443 -1.062685

H 1.527236 4.878605 -1.365609

C -0.505773 4.490910 -0.800500

H -0.827559 5.525421 -0.886142

C -1.411440 3.487234 -0.416783

C -2.804888 3.472277 -0.060412

H -3.471028 4.323931 -0.014784

C -3.139968 2.177804 0.223440

H -4.094407 1.765028 0.509967

C -0.947042 2.152379 -0.309018

C -2.702602 -1.079691 1.520116

C -2.249427 -0.246090 2.734844

H -2.594614 0.790649 2.677315

H -1.160029 -0.241448 2.832995

H -2.663293 -0.692606 3.648067

C -4.241020 -1.082663 1.457704

H -4.633806 -1.516456 2.386770

H -4.624893 -1.681667 0.629305

H -4.655507 -0.073820 1.372115

C -2.177965 -2.521886 1.705067

H -1.092788 -2.526120 1.840963

H -2.416261 -3.169691 0.858204

H -2.638661 -2.956248 2.601682

C -2.694323 -0.847855 -1.631835

C -1.656787 -0.524749 -2.731981

H -0.776384 -1.164757 -2.630226

H -1.341777 0.524508 -2.700361

H -2.107294 -0.710255 -3.716080

C -3.029791 -2.351704 -1.690201

H -3.831815 -2.627339 -0.998544

H -2.151352 -2.969388 -1.496646

H -3.386247 -2.581777 -2.703000

C -3.970573 -0.024580 -1.890517

H -4.410105 -0.358426 -2.839382

H -3.759496 1.044003 -1.981631

H -4.728442 -0.161985 -1.113572

N -2.024178 1.348939 0.084969

P -1.808543 -0.382422 -0.000560

Si 1.825375 0.848492 1.801252

Si 2.943482 -0.484842 -1.126160

C 2.304964 -1.358289 -2.684017

H 1.699171 -0.669441 -3.283833

H 3.171655 -1.660409 -3.289440

H 1.697082 -2.237311 -2.459951

C 4.077388 -1.619942 -0.123687

H 4.896200 -1.973523 -0.764722

H 4.523493 -1.107789 0.735395

H 3.513623 -2.477783 0.248535

C 3.998023 0.958406 -1.763370

H 4.357580 1.648879 -0.996536

H 4.882206 0.501672 -2.230890

H 3.477485 1.530602 -2.537560

C 3.490426 1.734980 1.655253

H 3.743083 2.093582 2.662941

H 4.309525 1.095804 1.316774

H 3.428111 2.609052 1.000936

C 0.647636 2.123798 2.559686

H -0.403368 1.838068 2.497233

H 0.908674 2.253891 3.619192

H 0.758672 3.085202 2.048852

C 2.021717 -0.630397 2.963743

H 2.675333 -1.389066 2.528042

H 2.448105 -0.280377 3.914165

H 1.062068 -1.114766 3.165078

Pd 0.786168 -0.208489 -0.077007

C 0.848466 -4.562997 0.475390

C 0.598516 -3.163119 -0.074339

O 1.140044 -2.217258 0.618088

O -0.061266 -2.997353 -1.112942

H 0.719181 -4.579661 1.561801

H 1.886068 -4.849718 0.265209

H 0.179788 -5.284422 0.001201

**INT3_B** Eopt -2193.559583

Si -2.131474 -1.283541 1.781373

Si -2.261618 -1.337269 -1.576090

C -1.178718 -1.177176 -3.116885

H -0.199706 -1.638768 -2.961960

H -1.682690 -1.685358 -3.951210

H -1.035061 -0.130083 -3.393568

C -3.898194 -0.448585 -1.882019

H -4.444353 -0.965908 -2.683034

H -4.532685 -0.411794 -0.994414

H -3.690945 0.575809 -2.203599

C -2.559793 -3.184621 -1.281745

H -3.020036 -3.592210 -2.192498

H -1.623145 -3.723874 -1.116993

H -3.233906 -3.397808 -0.447744

C -4.013779 -1.306723 1.625685

H -4.441236 -1.597677 2.594991

H -4.350326 -0.293503 1.387530

H -4.397958 -1.994834 0.868204

C -1.433728 -2.997812 2.144793

H -0.347316 -2.955595 2.260183

H -1.874245 -3.362551 3.083081

H -1.663779 -3.724661 1.361048

C -1.737859 -0.100186 3.207311

H -2.078852 0.906224 2.946861

H -2.289862 -0.435783 4.096699

H -0.676827 -0.061740 3.464670

C -3.527929 3.473239 -0.080535

C -2.590219 2.318886 -0.407552

O -2.583879 1.372095 0.475399

O -1.914328 2.315615 -1.448862

H -3.269802 3.898930 0.895218

H -4.555586 3.100013 -0.009319

H -3.469463 4.244426 -0.850937

C 2.852931 -1.179812 -0.205480

C 2.422330 -2.536021 -0.287093

C 3.387552 -3.554973 -0.353988

C 4.735965 -3.217038 -0.321321

C 5.142423 -1.872621 -0.217307

C 4.208639 -0.840697 -0.155913

C 0.983663 -2.537562 -0.249312

H 3.078534 -4.595006 -0.421887

H 5.488851 -3.999181 -0.368851

H 6.201718 -1.633554 -0.180961

H 4.529587 0.191080 -0.070514

H 0.354427 -3.415065 -0.270007

C 0.557197 -1.236365 -0.149936

N 1.702333 -0.396073 -0.143572

C 1.830526 2.233391 -1.415033

C 1.088786 1.602367 -2.612329

H 1.365806 2.144015 -3.526373

H 1.367150 0.551933 -2.747100

H 0.007043 1.675319 -2.483905

C 1.335340 3.683822 -1.231939

H 1.843416 4.200268 -0.410122

H 1.543141 4.248057 -2.149952

H 0.253253 3.711536 -1.064032

C 3.341140 2.195890 -1.703585

H 3.681718 1.176110 -1.901730

H 3.539489 2.788873 -2.606478

H 3.946568 2.617562 -0.899188

C 1.996237 1.842545 1.736946

C 2.114209 0.598357 2.640191

H 2.931117 -0.055222 2.321515

H 2.312575 0.919362 3.670858

H 1.196761 0.003393 2.642810

C 3.362814 2.544322 1.673822

H 4.144913 1.889180 1.280128

H 3.335617 3.456774 1.071753

H 3.660258 2.833068 2.690883

C 0.948941 2.813733 2.331269

H -0.026893 2.330689 2.443380

H 1.282151 3.151876 3.321030

H 0.812890 3.702405 1.704788

P 1.227763 1.261811 0.093053

Pd -1.065804 -0.050214 0.047727

**INT4_A** Eopt -2193.589556

C 1.271566 -1.824382 -0.441194

C 1.257185 -3.176878 -0.802672

H 0.420043 -3.804724 -0.525821

C 2.304694 -3.799544 -1.520889

H 2.213933 -4.856994 -1.760011

C 3.438072 -3.103113 -1.911617

H 4.245856 -3.592174 -2.450121

C 3.511890 -1.739072 -1.582303

C 4.496862 -0.708874 -1.783223

H 5.453114 -0.816362 -2.279346

C 4.008505 0.443662 -1.233597

H 4.457558 1.423621 -1.182767

C 2.430436 -1.146779 -0.884005

C 2.592153 1.697610 1.766931

C 3.291121 0.413423 2.267161

H 4.087400 0.092197 1.588978

H 2.581562 -0.414937 2.372182

H 3.736091 0.609749 3.251246

C 3.634832 2.810595 1.575655

H 4.168705 2.973506 2.521551

H 3.174616 3.763056 1.294262

H 4.382167 2.549099 0.819964

C 1.550831 2.130572 2.823089

H 0.801545 1.350818 2.991125

H 1.024710 3.047336 2.539735

H 2.062693 2.326261 3.774530

C 1.184810 2.611912 -0.918888

C 0.302584 1.969412 -2.013909

H -0.581823 1.492089 -1.584294

H 0.853501 1.206618 -2.574706

H -0.022471 2.744381 -2.722126

C 0.338638 3.619802 -0.116427

H 0.944800 4.191650 0.593434

H -0.463955 3.125742 0.442031

H -0.122719 4.338368 -0.806725

C 2.357633 3.336554 -1.603063

H 1.961522 4.146876 -2.230422

H 2.912210 2.658856 -2.257415

H 3.056201 3.784936 -0.892421

N 2.740021 0.212430 -0.694737

P 1.627026 1.162474 0.222926

Si -1.465060 -2.480856 1.032919

Si -3.812381 0.847714 -1.434527

C -4.302738 2.623819 -1.814259

H -3.414682 3.236786 -2.009262

H -4.932996 2.660193 -2.711549

H -4.866816 3.088335 -0.997504

C -5.260302 -0.170584 -0.808930

H -6.009400 -0.289691 -1.601718

H -4.929759 -1.170374 -0.507698

H -5.761528 0.293348 0.048322

C -2.917118 0.032245 -2.858451

H -2.182366 0.709724 -3.306721

H -2.376548 -0.850654 -2.500251

H -3.614842 -0.282613 -3.643487

C -2.305823 -3.265423 -0.496880

H -2.889563 -4.152146 -0.212692

H -2.999255 -2.546053 -0.950419

H -1.592644 -3.561159 -1.272615

C -0.657328 -3.859135 2.077028

H -0.411135 -3.465958 3.071533

H -1.344072 -4.706435 2.212956

H 0.270611 -4.233372 1.634888

C -2.940458 -1.891383 2.112197

H -3.608977 -1.216039 1.564796

H -3.538053 -2.757742 2.427658

H -2.597936 -1.367829 3.011661

Pd -0.041305 -0.643690 0.585110

C -3.518554 2.051706 1.717383

C -2.459075 1.198565 1.065321

O -1.469696 0.812747 1.677110

O -2.611606 0.877643 -0.209476

H -4.470603 1.509648 1.727566

H -3.664351 2.976095 1.149191

H -3.225777 2.284201 2.741392

**INT5_A** Eopt -2193.592389

C 1.086642 2.346333 0.054563

C 0.820442 3.506331 0.811476

H 0.024164 4.170017 0.480646

C 1.577494 3.918364 1.918846

H 1.315134 4.835164 2.440227

C 2.704393 3.198446 2.284882

H 3.364828 3.540847 3.077392

C 2.970080 1.997735 1.615122

C 4.014270 1.029132 1.794989

H 4.861204 1.108271 2.463940

C 3.734014 -0.012404 0.971373

H 4.275818 -0.933463 0.860372

C 2.107910 1.519592 0.583482

C 2.396099 -2.397741 -1.109726

C 2.397150 -1.817865 -2.538212

H 2.900630 -0.847891 -2.576793

H 1.381152 -1.695382 -2.922009

H 2.935419 -2.504929 -3.204993

C 3.860601 -2.723973 -0.752029

H 4.207514 -3.528972 -1.413484

H 3.994953 -3.071625 0.274199

H 4.517082 -1.867098 -0.927397

C 1.581965 -3.709746 -1.123163

H 0.521743 -3.519737 -1.325083

H 1.666154 -4.260993 -0.181887

H 1.966717 -4.360624 -1.919093

C 1.193047 -1.881465 1.768501

C 0.919793 -0.676443 2.693732

H 0.147515 -0.020280 2.282293

H 1.816840 -0.080725 2.871408

H 0.566277 -1.047860 3.665031

C -0.076899 -2.764188 1.768427

H 0.005508 -3.627737 1.103439

H -0.957137 -2.187492 1.467518

H -0.243810 -3.142089 2.786292

C 2.356506 -2.706423 2.347003

H 2.101095 -3.007756 3.372034

H 3.285559 -2.135498 2.404971

H 2.539631 -3.623837 1.779882

N 2.557589 0.208403 0.224684

P 1.409472 -1.156016 -0.017481

Si 0.375681 2.534080 -1.706613

Si -4.076036 0.396504 1.068556

C -5.234707 0.075205 2.511596

H -4.763228 -0.577828 3.254928

H -5.502891 1.013935 3.011859

H -6.162050 -0.405315 2.179322

C -4.930117 1.374520 -0.285235

H -5.298724 2.328314 0.113259

H -4.248708 1.585731 -1.112786

H -5.791531 0.824102 -0.681801

C -2.462548 1.154580 1.643778

H -2.109464 0.661176 2.556072

H -1.675244 1.059376 0.886814

H -2.588759 2.221628 1.865002

C -1.516311 2.617030 -1.693422

H -1.871765 3.074966 -2.625595

H -1.969838 1.625540 -1.611737

H -1.884521 3.227340 -0.860135

C 1.003764 4.238773 -2.275533

H 2.099242 4.280442 -2.242207

H 0.691996 4.428764 -3.310889

H 0.625185 5.058554 -1.655523

C 0.998358 1.342036 -3.026127

H 0.532442 0.355272 -2.955159

H 0.764894 1.764554 -4.012168

H 2.084211 1.216081 -2.963297

Pd -0.603581 -0.693655 -0.867468

C -3.063943 -3.031420 -0.848672

C -3.137077 -1.541276 -0.618434

O -2.765262 -0.692897 -1.445387

O -3.763977 -1.221382 0.512440

H -4.082650 -3.428960 -0.937029

H -2.589620 -3.519990 0.006398

H -2.500091 -3.239187 -1.758207

**Si_2_Me_6_** Eopt -818.553451

Si -1.179021 0.000330 0.001076

Si 1.179021 -0.000329 -0.001076

C -1.840040 -0.215139 -1.769553

H -1.504190 -1.162045 -2.209055

H -2.937407 -0.213430 -1.780840

H -1.498886 0.595155 -2.425039

C -1.836112 -1.427083 1.072799

H -1.497698 -1.335803 2.111850

H -2.933458 -1.440164 1.079581

H -1.493889 -2.398345 0.695780

C -1.835716 1.642904 0.700147

H -2.932980 1.653457 0.713365

H -1.488844 1.807601 1.727314

H -1.501638 2.494758 0.095740

C 1.835712 -1.642911 -0.700130

H 2.932976 -1.653468 -0.713346

H 1.501630 -2.494759 -0.095715

H 1.488842 -1.807617 -1.727295

C 1.836113 1.427071 -1.072815

H 1.493890 2.398337 -0.695809

H 2.933460 1.440151 -1.079597

H 1.497700 1.335777 -2.111865

C 1.840042 0.215159 1.769551

H 1.498883 -0.595125 2.425048

H 2.937409 0.213443 1.780837

H 1.504198 1.162072 2.209040

**TSI_A** Eopt -1604.069840

C 0.141032 3.184053 -0.858415

H 1.171517 3.445036 -1.080462

C -0.886161 4.080009 -1.197074

H -0.636590 5.000321 -1.717448

C -2.214968 3.827559 -0.851960

H -2.994464 4.541720 -1.104165

C -2.530863 2.626331 -0.198224

C -3.747452 1.956361 0.193934

H -4.753428 2.351063 0.137587

C -3.403117 0.717062 0.652402

H -4.033035 -0.074047 1.027439

C -1.474207 1.744233 0.110951

C -1.047543 -1.886547 1.834363

C -0.106597 -1.164459 2.826232

H -0.403102 -0.121841 2.983960

H 0.928819 -1.181774 2.475151

H -0.152885 -1.680049 3.793956

C -2.453549 -1.980684 2.456861

H -2.394937 -2.612221 3.352075

H -3.189216 -2.431719 1.785651

H -2.817433 -0.998830 2.773650

C -0.485011 -3.290181 1.535895

H 0.487573 -3.226448 1.039502

H -1.167429 -3.887734 0.923591

H -0.350134 -3.819629 2.487464

C -1.813963 -1.663689 -1.255658

C -2.060427 -0.520623 -2.266657

H -1.139923 0.033834 -2.476228

H -2.824218 0.183263 -1.923151

H -2.407751 -0.962750 -3.208335

C -0.801508 -2.651326 -1.874560

H -0.537955 -3.466914 -1.196638

H 0.117998 -2.150904 -2.187351

H -1.265204 -3.098685 -2.763111

C -3.136663 -2.389795 -0.951750

H -3.550873 -2.766761 -1.895244

H -3.887415 -1.728657 -0.509921

H -2.993200 -3.251363 -0.292834

C 4.332423 2.512564 1.164832

H 4.263406 3.459130 1.703957

H 4.922049 2.625474 0.251909

H 4.838620 1.777783 1.801612

C 2.950827 1.984520 0.831231

C 3.477960 -3.235539 -0.937109

H 4.294354 -2.965940 -0.258902

H 3.883879 -3.525162 -1.908184

H 2.946504 -4.081985 -0.487968

C 2.532858 -2.050401 -1.100327

N -2.004251 0.556380 0.612091

O 2.874617 1.132638 -0.111278

O 1.972842 -1.688403 0.027104

O 2.327921 -1.525230 -2.193950

P -1.016995 -0.850199 0.258633

Pd 1.000764 0.133302 -0.134284

O 1.963702 2.403050 1.511146

C -0.109285 1.995547 -0.141448

H 0.798629 1.995823 0.780077

**TSI_B** Eopt -1604.058421

C -2.800144 0.493080 0.091500

C -2.916872 1.730337 -0.605572

C -4.189529 2.191024 -0.995057

C -5.301911 1.421337 -0.691986

C -5.170836 0.206189 0.016150

C -3.928774 -0.267800 0.420487

C -1.603639 2.286704 -0.712123

H -4.290602 3.136682 -1.519935

H -6.290941 1.760393 -0.985961

H -6.062010 -0.363649 0.263578

H -3.838733 -1.190316 0.983449

H -1.347786 3.258974 -1.112291

C -0.717252 1.447956 -0.063488

H 0.080073 2.154422 0.629509

N -1.458156 0.310684 0.416096

C -0.847910 -2.200478 -1.074393

C -0.694668 -1.390647 -2.382529

H -1.037391 -2.016137 -3.216716

H -1.307515 -0.482006 -2.372121

H 0.350655 -1.122853 -2.556408

C 0.138294 -3.387714 -1.082314

H -0.006381 -4.043287 -0.217606

H -0.060325 -3.982846 -1.982167

H 1.177102 -3.055029 -1.136871

C -2.290139 -2.733992 -0.973062

H -3.028096 -1.952653 -1.157257

H -2.418418 -3.497691 -1.750375

H -2.511349 -3.206605 -0.013011

C -0.147337 -1.764680 2.022427

C -0.123768 -0.574458 3.006203

H -1.072737 -0.030714 3.013627

H 0.060963 -0.961312 4.015759

H 0.674974 0.135339 2.765403

C -1.294639 -2.719977 2.392049

H -2.273155 -2.231168 2.327068

H -1.307579 -3.616112 1.764604

H -1.161442 -3.048648 3.430475

C 1.213753 -2.494653 2.087056

H 2.036359 -1.839709 1.783728

H 1.386589 -2.816050 3.121781

H 1.239335 -3.388150 1.457404

P -0.313628 -1.016965 0.300643

Pd 1.251608 0.572190 -0.176431

O 3.010620 -0.466023 0.006047

C 3.333912 -1.260068 -0.974838

O 2.628693 -1.529328 -1.950991

C 4.720818 -1.871812 -0.799569

H 4.940289 -2.545582 -1.629967

H 5.471253 -1.075742 -0.757539

H 4.772504 -2.417576 0.148856

O 2.364886 2.363560 -0.355825

C 1.947081 3.298908 0.400283

O 0.876454 3.272135 1.080485

C 2.830088 4.528462 0.506413

H 2.246436 5.393318 0.827730

H 3.607566 4.331370 1.253749

H 3.323525 4.723226 -0.448549

**TSII_A** Eopt -2193.543064

C -0.091276 1.775047 -0.585990

C 0.682228 2.812061 -1.105285

H 1.691466 2.628361 -1.457581

C 0.185822 4.138984 -1.166523

H 0.835310 4.914998 -1.564685

C -1.088926 4.479529 -0.726845

H -1.438919 5.506899 -0.778271

C -1.914573 3.453801 -0.228803

C -3.260448 3.336200 0.276435

H -3.977734 4.135826 0.410056

C -3.485629 2.016078 0.569577

H -4.378290 1.541622 0.946064

C -1.374892 2.153132 -0.191164

C -2.303220 -1.172296 1.832736

C -1.646186 -0.268500 2.898273

H -2.056562 0.745823 2.888823

H -0.563861 -0.202598 2.759367

H -1.831026 -0.702794 3.888682

C -3.817908 -1.256662 2.101021

H -3.971749 -1.679164 3.102154

H -4.331105 -1.908413 1.389289

H -4.300072 -0.274807 2.088166

C -1.679541 -2.581871 1.918506

H -0.608315 -2.565514 1.705983

H -2.150697 -3.286507 1.228708

H -1.830676 -2.964485 2.935806

C -2.931521 -1.063739 -1.286801

C -2.197255 -0.609503 -2.568647

H -1.231408 -1.112445 -2.659506

H -2.041517 0.474786 -2.584998

H -2.813179 -0.876164 -3.437256

C -3.010441 -2.601803 -1.263342

H -3.601977 -2.974846 -0.421304

H -2.018873 -3.058709 -1.256098

H -3.512323 -2.929107 -2.182711

C -4.353441 -0.465746 -1.284854

H -4.908976 -0.910870 -2.119700

H -4.341753 0.616715 -1.435177

H -4.911469 -0.683720 -0.369822

N -2.333957 1.269562 0.295755

P -1.881171 -0.401739 0.155530

Si 2.621413 0.573174 1.508908

Si 3.166839 0.045269 -0.887523

C 2.184726 -0.228645 -2.510641

H 1.643826 0.661089 -2.846417

H 2.972669 -0.435596 -3.250795

H 1.503740 -1.085280 -2.513591

C 4.163930 -1.542630 -0.604807

H 4.939680 -1.428909 0.158939

H 3.481223 -2.336036 -0.288203

H 4.647357 -1.850353 -1.540520

C 4.327682 1.500825 -1.310060

H 5.265908 1.434781 -0.751642

H 4.564922 1.464713 -2.380557

H 3.886341 2.481271 -1.100374

C 4.407381 1.141448 1.907259

H 4.469260 1.231909 3.000236

H 5.185019 0.440845 1.588001

H 4.631201 2.123493 1.478991

C 1.577609 2.027223 2.140669

H 0.507925 1.811626 2.178662

H 1.924246 2.275893 3.153229

H 1.704016 2.909743 1.506040

C 2.367833 -0.971439 2.576375

H 3.066227 -1.759130 2.276086

H 2.572995 -0.709293 3.622978

H 1.364795 -1.392458 2.501766

Pd 0.406680 -0.164030 -0.244093

C 1.157304 -4.500424 -0.330085

C 0.728170 -3.090461 -0.725976

O 0.986359 -2.195750 0.176018

O 0.168204 -2.878972 -1.811221

H 0.529637 -4.848795 0.499181

H 2.191982 -4.499753 0.027718

H 1.052109 -5.184236 -1.174607

**TSII_B** Eopt -2193.531692

C 2.956774 -1.543486 -0.230562

C 2.303981 -2.804659 -0.387325

C 3.081175 -3.974571 -0.388360

C 4.459849 -3.879117 -0.229325

C 5.084929 -2.627890 -0.079961

C 4.343445 -1.447545 -0.085540

C 0.885555 -2.570286 -0.511861

H 2.603745 -4.943485 -0.509824

H 5.064728 -4.781901 -0.224055

H 6.163795 -2.576140 0.037411

H 4.838959 -0.488658 0.009458

H 0.119436 -3.326259 -0.615574

C 0.691476 -1.216299 -0.437464

N 1.943560 -0.581207 -0.294022

C 2.286139 2.179561 -1.173386

C 1.403082 2.147679 -2.438270

H 1.886604 2.741796 -3.224144

H 1.273388 1.127462 -2.816191

H 0.418989 2.575440 -2.231464

C 2.411988 3.630270 -0.670907

H 3.087109 3.715373 0.185812

H 2.844720 4.230040 -1.482096

H 1.441057 4.049254 -0.407919

C 3.684270 1.624703 -1.511423

H 3.642492 0.607962 -1.909848

H 4.132098 2.268854 -2.278333

H 4.354539 1.635026 -0.646813

C 1.895576 1.305813 1.890329

C 1.426542 0.047330 2.652057

H 1.945846 -0.855240 2.316963

H 1.642386 0.187992 3.718281

H 0.352032 -0.118897 2.544580

C 3.421401 1.447819 2.061197

H 3.955992 0.561725 1.710401

H 3.821544 2.328997 1.554057

H 3.639557 1.559930 3.130868

C 1.175392 2.545040 2.453045

H 0.092989 2.470748 2.337647

H 1.414579 2.625075 3.521337

H 1.486689 3.468848 1.962123

P 1.426429 1.033366 0.076438

Pd -0.677462 0.232890 -0.391638

O -2.057717 1.825176 -0.492178

C -1.660202 3.024152 -0.235306

O -0.501974 3.347944 0.077978

C -2.758051 4.078122 -0.327481

H -2.347200 5.078910 -0.182229

H -3.257235 4.013974 -1.299893

H -3.515583 3.878479 0.439119

Si -3.172524 -1.294904 -1.032203

Si -2.827528 -1.179787 1.371377

C -1.623245 -2.474756 2.055172

H -0.582991 -2.264971 1.796898

H -1.713819 -2.519251 3.148503

H -1.867911 -3.469850 1.663717

C -4.574910 -1.760220 1.894822

H -4.574720 -1.883204 2.986196

H -5.340201 -1.016961 1.646841

H -4.872852 -2.716999 1.454393

C -2.646279 0.494976 2.249086

H -3.020538 0.395187 3.276607

H -1.619459 0.865628 2.291633

H -3.236863 1.254193 1.728540

C -3.517769 -3.155422 -1.258573

H -3.857332 -3.343817 -2.285069

H -2.602561 -3.739070 -1.099918

H -4.281791 -3.536124 -0.574139

C -2.049916 -0.907049 -2.544429

H -2.705402 -1.156491 -3.392702

H -1.765596 0.142946 -2.675172

H -1.158760 -1.536455 -2.619787

C -4.716015 -0.229599 -1.308247

H -5.064635 -0.322365 -2.344591

H -5.543840 -0.496475 -0.643724

H -4.444947 0.817135 -1.129525

**TSIII_A** Eopt -2193.549613

C -0.276863 1.867267 -0.203052

C 0.257402 3.155707 -0.122321

H 1.304989 3.284659 0.106982

C -0.504569 4.322941 -0.349185

H -0.011303 5.289246 -0.276439

C -1.856088 4.262029 -0.657064

H -2.439102 5.164462 -0.820662

C -2.454980 2.994533 -0.731457

C -3.802164 2.531863 -0.942908

H -4.670833 3.144860 -1.146262

C -3.792042 1.169212 -0.847887

H -4.607084 0.468411 -0.940679

C -1.652832 1.844939 -0.520942

C -2.651609 -0.961713 1.716241

C -2.603502 0.435387 2.374855

H -3.252752 1.152546 1.863561

H -1.590724 0.847498 2.389525

H -2.946176 0.346937 3.413633

C -4.108596 -1.453093 1.689106

H -4.500792 -1.469594 2.714643

H -4.198153 -2.467549 1.290168

H -4.752535 -0.788506 1.104101

C -1.778627 -1.929562 2.545006

H -0.731808 -1.613301 2.566420

H -1.805615 -2.952531 2.160411

H -2.155514 -1.952517 3.575800

C -2.301910 -2.114759 -1.208574

C -1.361222 -1.898156 -2.411384

H -0.316556 -2.036383 -2.124847

H -1.471270 -0.894260 -2.836196

H -1.598552 -2.626467 -3.197763

C -1.971479 -3.475864 -0.562212

H -2.687616 -3.747790 0.219229

H -0.964264 -3.480480 -0.132270

H -2.016582 -4.255343 -1.333497

C -3.757485 -2.091276 -1.708390

H -3.915013 -2.946625 -2.378578

H -3.964160 -1.184659 -2.284048

H -4.489221 -2.168993 -0.900844

N -2.486965 0.716390 -0.619733

P -1.832344 -0.752745 0.013486

Si 2.257117 0.894534 1.625462

Si 2.583576 0.019872 -1.574828

C 1.968078 -0.917431 -3.111317

H 0.996560 -0.534877 -3.439149

H 2.685738 -0.695730 -3.914326

H 1.914187 -2.001506 -2.995060

C 4.477287 -0.009881 -1.358854

H 4.930834 0.901238 -1.765017

H 4.787728 -0.113868 -0.315511

H 4.881274 -0.877663 -1.891626

C 2.322899 1.817792 -2.197482

H 1.285766 2.047145 -2.448039

H 2.686886 2.582323 -1.507273

H 2.929889 1.883429 -3.114703

C 3.523759 2.260233 1.241301

H 4.152428 2.368085 2.136429

H 4.181308 2.025334 0.401554

H 3.062543 3.233825 1.052132

C 1.154637 1.556287 3.028557

H 0.497136 0.773420 3.422484

H 1.786581 1.911647 3.854540

H 0.532597 2.385881 2.680047

C 3.232537 -0.572283 2.337457

H 3.886953 -1.031453 1.591753

H 3.850400 -0.223300 3.175355

H 2.547313 -1.340936 2.708277

Pd 0.562522 0.024519 0.144856

C 2.457079 -3.917493 0.550754

C 2.045126 -2.518144 0.138807

O 0.987541 -2.040351 0.645542

O 2.803549 -1.891881 -0.650586

H 3.133741 -3.834612 1.409582

H 2.994609 -4.415410 -0.259127

H 1.586621 -4.503054 0.854878

**TSIV_A** Eopt -2193.574745

C -0.072329 1.749507 -0.472630

C 0.717343 2.727364 -1.101022

H 1.700700 2.953327 -0.701910

C 0.263034 3.480147 -2.207230

H 0.917949 4.237412 -2.631590

C -1.009896 3.300144 -2.730827

H -1.370375 3.914835 -3.551577

C -1.816235 2.281255 -2.189858

C -3.116433 1.751841 -2.508764

H -3.805952 2.128802 -3.253121

C -3.325230 0.673104 -1.695791

H -4.174122 0.008489 -1.648326

C -1.307593 1.505627 -1.122257

C -3.038843 -1.367001 1.225854

C -3.455953 -0.034060 1.877467

H -3.952282 0.630520 1.164529

H -2.598772 0.498967 2.293686

H -4.153633 -0.239576 2.699865

C -4.296268 -2.056986 0.669035

H -5.015649 -2.206385 1.485472

H -4.081677 -3.041018 0.243416

H -4.795990 -1.452524 -0.094310

C -2.400270 -2.264200 2.310578

H -1.520039 -1.784942 2.752259

H -2.092675 -3.238570 1.917899

H -3.129735 -2.445582 3.110826

C -1.593591 -2.286391 -1.422992

C -0.349932 -1.897832 -2.251909

H 0.563119 -1.949633 -1.651342

H -0.431792 -0.882675 -2.654959

H -0.243053 -2.590867 -3.097491

C -1.333929 -3.653545 -0.760349

H -2.223011 -4.051657 -0.261636

H -0.523788 -3.596387 -0.024575

H -1.036221 -4.379015 -1.528443

C -2.794427 -2.386811 -2.380489

H -2.613563 -3.206934 -3.088839

H -2.914789 -1.471998 -2.965733

H -3.736597 -2.599928 -1.871119

N -2.230262 0.480321 -0.846374

P -1.649639 -0.975265 -0.025947

Si 0.068091 2.149872 1.751369

Si 3.914109 -0.661668 -0.976138

C 5.424986 -1.616394 -1.559577

H 5.133705 -2.538489 -2.075616

H 6.019372 -1.013892 -2.257546

H 6.071654 -1.890417 -0.717977

C 4.380618 0.871374 -0.003359

H 5.010250 1.526335 -0.619659

H 3.477295 1.416428 0.282061

H 4.926800 0.629355 0.913651

C 2.781204 -0.226909 -2.403825

H 2.477324 -1.113164 -2.971080

H 1.881202 0.283534 -2.045630

H 3.298859 0.453605 -3.092413

C 1.564283 3.304393 1.943048

H 1.544063 3.723688 2.957524

H 2.509534 2.761622 1.832454

H 1.554428 4.139040 1.236166

C -1.499802 3.220328 1.696048

H -2.385604 2.636740 1.428094

H -1.679653 3.689258 2.672317

H -1.390898 4.009898 0.944575

C 0.018493 1.206745 3.430102

H 0.950860 0.657313 3.597206

H -0.085530 1.961745 4.223677

H -0.813233 0.502892 3.532096

Pd 0.395835 0.003856 0.675948

C 1.974379 -2.940319 1.786866

C 2.478434 -1.649468 1.182568

O 2.453980 -0.565650 1.770562

O 3.091715 -1.832705 0.004460

H 2.825560 -3.465402 2.240166

H 1.550152 -3.592929 1.021410

H 1.235184 -2.721946 2.558218
